# Lewis acid mediated, mild C–H aminoalkylation of azoles *via* three component coupling†

**DOI:** 10.1039/d0sc06868c

**Published:** 2021-02-05

**Authors:** Marion H. Emmert, Cyndi Qixin He, Akshay A. Shah, Stephanie Felten

**Affiliations:** Process Research & Development, MRL, Merck & Co. Inc. 770 Sumneytown Pike, West Point PA 19486 USA marion.emmert@merck.com; Computational and Structural Chemistry, MRL, Merck & Co. Inc. 126 E Lincoln Ave Rahway NJ 07065 USA cyndi.he@merck.com; Discovery Chemistry, MRL, Merck & Co. Inc. 770 Sumneytown Pike, West Point PA 19486 USA

## Abstract

This manuscript reports the development of a mild, highly functional group tolerant and metal-free C–H aminoalkylation of azoles *via* a three-component coupling approach. This method enables the C–H functionalization of diverse azole substrates, such as oxazoles, benzoxazoles, thiazoles, benzothiazoles, imidazoles, and benzimidazoles. DFT calculations identify a key deprotonation equilibrium in the mechanism of the reaction. Using DFT as a predictive tool, the C–H aminoalkylation of initially unreactive substrates (imidazoles/benzimidazoles) can be enabled through an *in situ* protecting/activating group strategy. The DFT-supported mechanistic pathway proposes key interactions between the azole substrate and the Lewis acid/base pair TBSOTf/EtN^i^Pr_2_ that lead to azole activation by deprotonation, followed by C–C bond formation between a carbene intermediate and an iminium electrophile. Two diverse approaches are demonstrated to explore the amine substrate scope: (i) a DFT-guided predictive analysis of amine components that relates reactivity to distortion of the iminium intermediates in the computed transition state structures; and (ii) a parallel medicinal chemistry workflow enabling synthesis and isolation of several diversified products at the same time. Overall, the presented work enables a metal-free approach to azole C–H functionalization *via* Lewis acid mediated azole C–H deprotonation, demonstrating the potential of a readily available, Si-based Lewis acid to mediate new C–C bond formations.

## Abstract 2

Lewis acid mediated activation enables mild, metal-free, and highly functional group tolerant C–H aminoalkylation of diverse azoles *via* three-component coupling.

## Graphical abstract

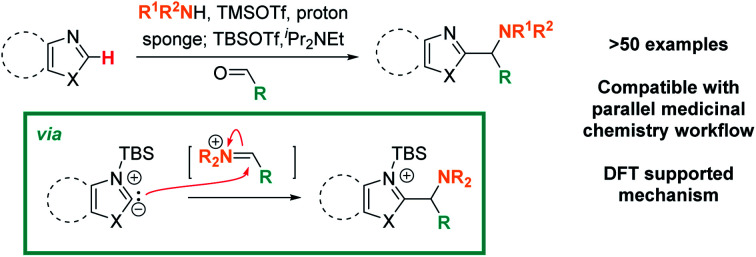

## Introduction

**Scheme 1:**
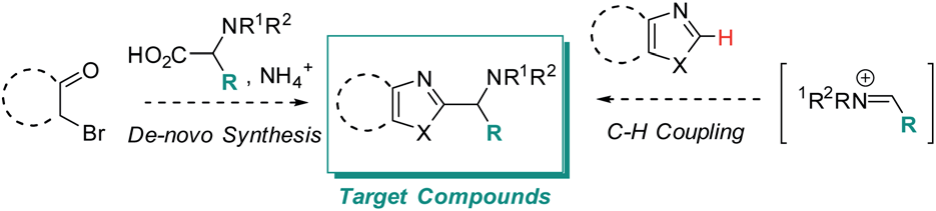
*De novo* synthesis of alpha-amino azoles requiring pre-functionalized building blocks (left) and target transformation (right) to allow rapid diversification *via* C–H functionalization and *in situ* iminium formation.

Functionalized azole scaffolds are common target molecules in drug discovery. However, efficient diversifying routes towards these building blocks are underdeveloped. Typical approaches to access such chemical matter include multi-step, *de novo* syntheses of the azole core through condensation approaches.^1^ Under the framework of a recent drug discovery program, azoles with pendant amino groups in alpha-position became an important structural class (Scheme 1). However, the rapid diversification of the chemical matter was limited by the lengthy *de novo* syntheses as well as by substrate-dependent synthetic success of assembling different azole cores from alpha-bromo ketones, NH_3_ (or a surrogate), and complex α-amino acids (Scheme 1, left side).^1^ Therefore, we envisioned a different disconnection: direct C–H functionalization of the azole core with iminium electrophiles, thereby opening the door to one-step access to the desired structures and 3-component couplings, if the iminium electrophile can be accessed *in situ* from aldehyde and amine precursors (Scheme 1, right side).^2^

This strategy would also contribute to establishing more general and synthetically useful methods for azole C–H alkylation, which are generally challenging transformations. In fact, most azole C–H alkylation methods are promoted by transition metals and require forcing conditions, such as high temperatures, stoichiometric amounts of strong bases, and high catalyst loadings.^3–5^ Although various metal-free azole C–H alkylations are reported in the literature,^2*b*,3,6^ no examples of direct C–H aminoalkylations of azoles under mild reaction conditions have been described.

**Scheme 2:**
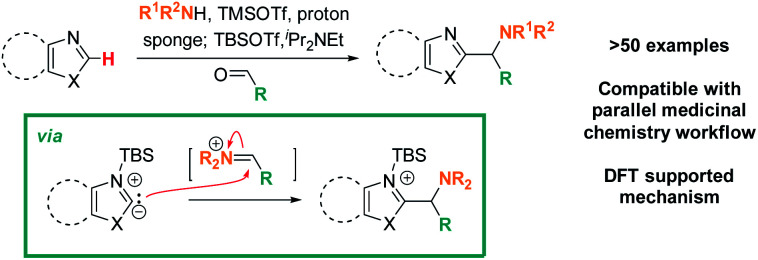
Lewis-acid promoted, redox-neutral, one-pot, three-component azole C–H aminoalkylation & proposed mechanism.

Addressing these limitations, this manuscript describes a Lewis acid promoted direct C–H functionalization of azole substrates, which proceeds under mild conditions (Scheme 2). Trapping the *in situ* generated iminium electrophile with an azole nucleophile produces a diversity of heterocyclic structures in a three-component coupling approach.

The mildness of the protocol is showcased by its ability to proceed in the presence of reactive functional groups such as amides, heterocycles, alkynes, and even enolizable ketones. Furthermore, its compatibility with a parallel medicinal chemistry workflow has been demonstrated, allowing access to compound arrays in parallel. Data from reaction optimization and DFT calculations support the need for Lewis acid and amine base to act in concert for successful C–H aminoalkylation, supporting the intermediacy of a silylated carbene as the key reactive species in these reactions.

## Results and discussion

### Reaction design and optimizations

**Scheme 3:**
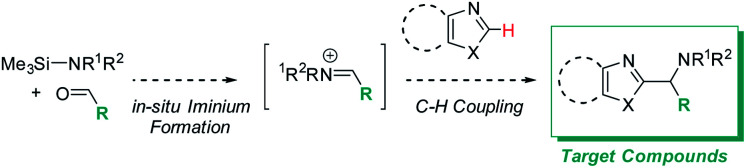
Desired target compounds and reaction design for C–H aminoalkylation.

**Scheme 4:**
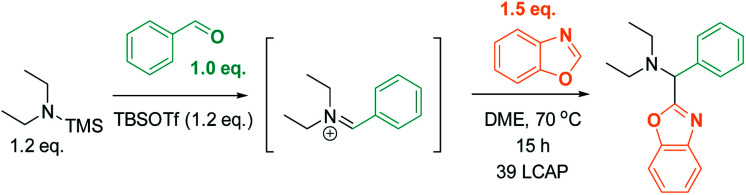
Metal-free formation of desired C–H aminoalkylation product. LCAP = LC area percent.

We initially reasoned that synthesis of the highly functionalized target compounds could be achieved by direct C–H functionalization of azoles^4–6^ with an *in situ* formed iminium electrophile (Scheme 3); the procedure for forming the iminium intermediates was inspired by conditions used in Doyle's reductive three-component coupling.^7^ A reaction optimization screen (solvents, temperature, metal catalyst precursors, ligands, bases, acids; for details see ESI†) *via* high throughput experimentation led to the discovery that C–H aminoalkylation of benzoxazole at the 2-position was successful (39 LCAP; LCAP = LC area percent) when the azole was reacted with the *in situ* formed iminium electrophile in DME – even in the absence of any metal catalyst (Scheme 4).

**Scheme 5:**
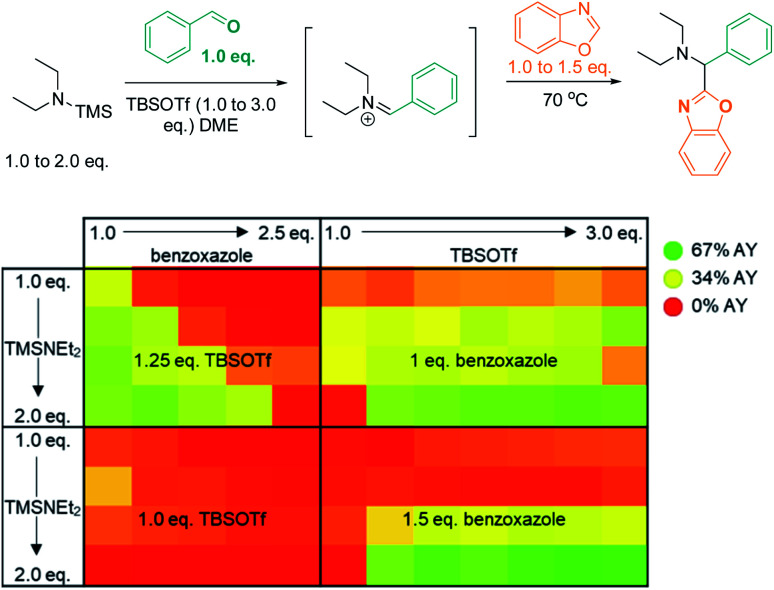
Optimization *via* high-throughput screening: influence of 3 variables (benzoxazole, TBSOTf, and Et_2_NTMS loading) on product yield. AY = assay yield, as determined by calibrated UPLC analysis (210 nm), using biphenyl as internal standard.

A multidimensional optimization of reactant loadings (Scheme 5; 1.0 to 3.0 eq. TBSOTf, 1.0 to 2.5 eq. benzoxazole, 1.0 to 2.0 eq. TMS–NEt_2_) revealed that all varied reaction components showed a significant influence on the reaction outcome. The highest assay yields (65–66% AY; determined by calibrated UPLC analysis) were obtained under conditions with an excess of both basic and acidic reactants (2.5 or 3.0 eq. TBSOTf, 2.0 eq. TMS–NEt_2_, 1.5 eq. benzoxazole). These findings imply that high concentrations of acidic reagent (TBSOTf) and basic reagents promote high yields. As both basic components of the reaction mixture (TMS–NEt_2_, benzoxazole) contribute to the skeletal atoms of the desired product, reducing the required stoichiometry (ideally to 1 : 1) is highly desirable.

**Scheme 6:**
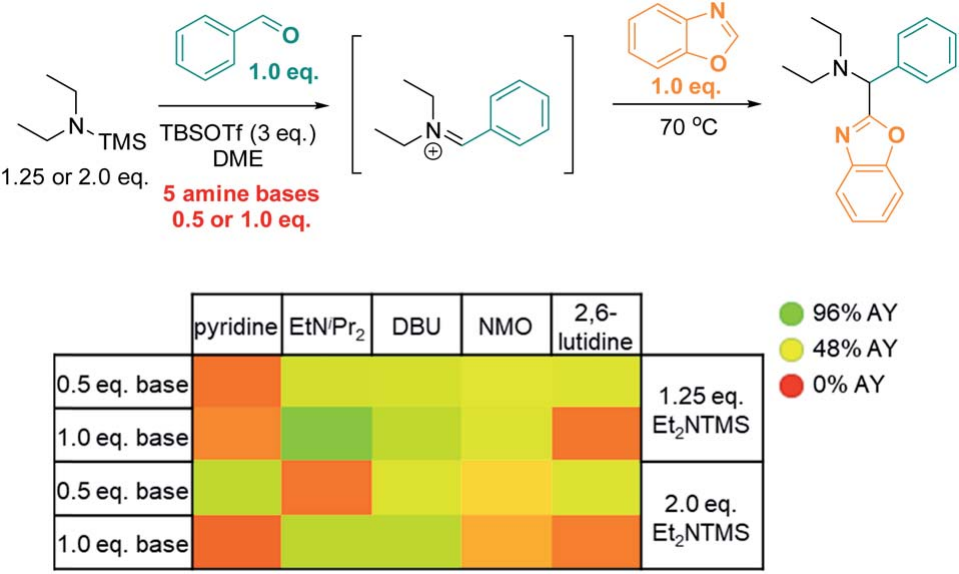
High throughput screen: base optimization. AY = assay yield, as determined by calibrated UPLC analysis (210 nm), using biphenyl as internal standard.

We reasoned that substituting the excess of basic reactants with an exogenous base may result in even better conversion and higher efficiency. To test this hypothesis, we surveyed the effects of five base additives (pyridine, EtN^i^Pr_2_, DBU, NMO, 2,6-lutidine; Scheme 6) at two different loadings (0.5 eq. and 1.0 eq.) in the presence of two concentrations of TMS–NEt_2_ (1.25 eq. and 2.0 eq.). In this series, most of the tested conditions afforded yields close to 50% AY. The highest yield was obtained with the sterically bulky base EtN^i^Pr_2_ (1.0 eq.) in combination with 1.25 eq. TMS–NEt_2_ (96% AY), while other common bases (pyridine, 2,6-lutidine) were detrimental to the reactivity under analogous conditions (22% and 17% AY, respectively; 1.0 eq. base, 1.25 eq. TMS–NEt_2_).

### Mechanistic hypothesis

Based on the data presented above, both TBSOTf and bases promote the reaction. Among the tested bases, EtN^i^Pr_2_ is the most bulky base, but not the most basic (p*K*_a_'s of the corresponding protonated ammonium acids: 5.2 – pyridine;^8^ 6.6 – 2,6-lutidine;^8^ 9.2 – NMO;^9^ 10.5 – EtN^i^Pr_2_;^10^ 12 – DBU^11^). This suggests that steric hindrance around the base lone pair is beneficial for the overall reaction outcome. The influence of steric bulk on chemical reactivity of acids and bases has been documented particularly in investigations of Frustrated Lewis Pairs (FLPs);^12^ the results from the amine screen described in the last paragraph thus led us to hypothesize that steric factors between EtN^i^Pr_2_ and TBSOTf may weaken the interaction between this Lewis base and acid pair and in turn enable efficient reactivity.

**Scheme 7:**
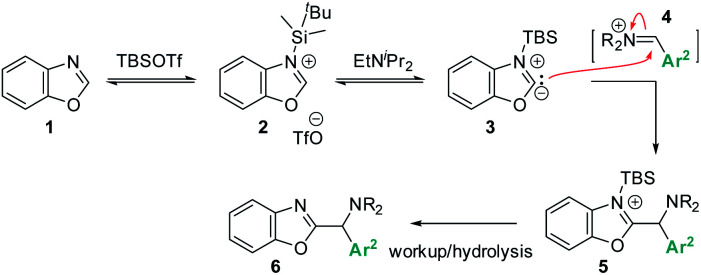
Proposed reaction mechanism *via* Lewis acid mediated azole deprotonation.

Based on this reasoning, our mechanistic hypothesis (Scheme 7) assigns important roles to EtN^i^Pr_2_ and TBSOTf: we propose that the TBSOTf first reacts with the azole substrate **1** to form silylated intermediate **2**, which then undergoes deprotonation. In a subsequent reaction step inspired by the mechanism of the Stetter reaction,^13^ the formed carbene **3** undergoes C–C bond formation with the pre-formed iminium electrophile **4**. Hydrolysis of **5** during workup and/or isolation leads to the final product **6**.

Preliminary experimental studies that react **1** with TBSOTf, ^i^Pr_2_NEt, and TfOH–D_1_ indeed detect mass spectroscopic evidence for **2** as well as deuterium incorporation in **1**. These data (see ESI† for details) support the accessibility of a deprotonated species such as **3** under the tested conditions.

### DFT support for deprotonation pathway

**Scheme 8:**
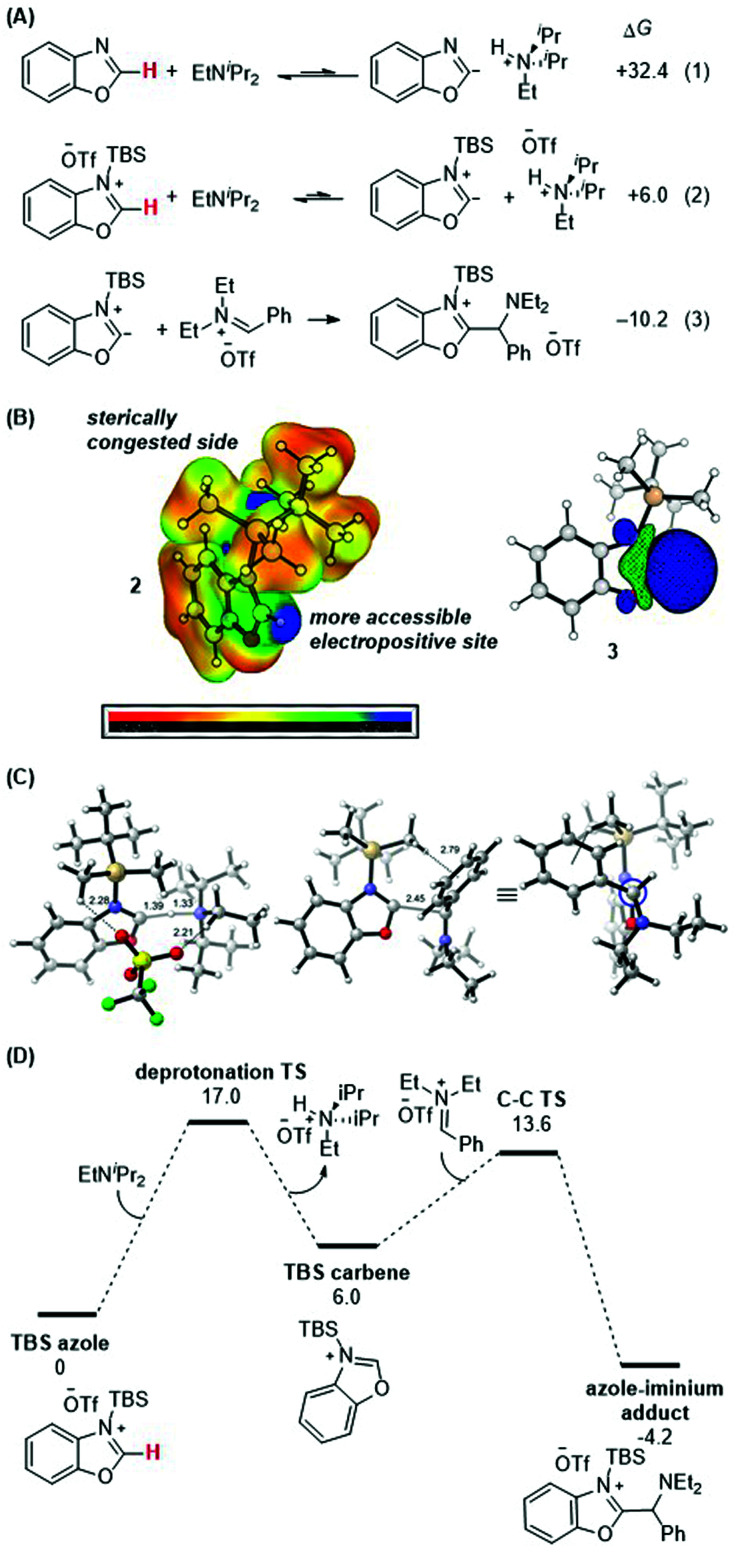
(A) Calculated thermodynamics for deprotonation equilibria and C–C bond formation with benzoxazole and silylated benzoxazole. (B) HOMO of silylated benzoxazole. (C) Lowest-energy transition state of C–C bond formation. (D) Energy profile. See ESI† for details of computational methods. M06-2X/def2TZVPP/SMD(ether)//M06-2X/6-31G(d)/SMD(ether).

DFT calculations were performed to gain further insight into the thermodynamic and kinetic features of the proposed mechanistic pathway. Scheme 8A details the obtained thermodynamics of deprotonation and C–C bond formation. Without silylation at the N-atom of benzoxazole, deprotonation of the C–H bond at the 2-position is highly endergonic (eqn (1); Δ*G* = +32.4 kcal mol^−1^) and thus unlikely to occur. Silylation with TBS (eqn (2)) activates this position and drastically shifts the deprotonation equilibrium by over 26 kcal mol^−1^ (Δ*G* = +6.0 kcal mol^−1^). Finally, the energetics of the C–C bond formation step (eqn (3); Δ*G* = −10.2 kcal mol^−1^) are exergonic, resulting in an overall thermodynamically favorable deprotonation/C–C bond formation sequence.

Scheme 8B shows the electrostatic potential surface of the silylated azole **2** and the pictorial representation of the HOMO for the proposed carbene intermediate **3**. While there are two electropositive regions in **2**, the azole 2-position is spatially more accessible. Upon deprotonation, the large blue orbital lobe localized at the azole 2-position in **3** shows that this is the most nucleophilic site in the molecule.

Finally, the lowest energy transition states for the deprotonation and the C–C bond formation steps were computed (Scheme 8C and D). The proton transfer transition state was located with an activation barrier of 17.0 kcal mol^−1^ and is the rate-determining step of the reaction. The deprotonation barrier excluding the triflate counterion in the model is 2.1 kcal mol^−1^ lower. Upon formation of the carbene, the C–C bond forming TS between the silylated carbene intermediate (with TBS as silyl group and benzoxazole as azole) and the iminium electrophile [PhCH=NEt_2_]^+^ is 13.6 kcal mol^−1^. The TS structure features a forming C–C bond distance of 2.5 Å and a favorable electrostatic interaction between the α-C–H of the TBS group and the phenyl group of the iminium reactant at 2.8 Å. Higher energy conformers are reported in the ESI.†

### Scope and limitations: azole scope

**Scheme 9:**
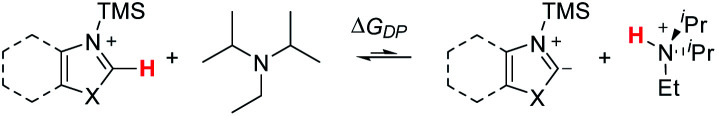
Deprotonation (DP) equilibrium calculated for each azole substrate (shown in Scheme 10).

With the optimized experimental conditions in hand and a reasonable mechanistic proposal supported by DFT calculations, we next set out to test the generality of the established C–H aminoalkylation protocol. With regard to demonstrating functional group tolerance, we reasoned that differently substituted azoles would be good indicators of compatibility with typical complex molecules encountered in drug discovery and development. In addition to experimentally subjecting azole substrates to the optimized conditions, the free energy of deprotonation (Δ*G*_DP_; Scheme 9) was determined for each substrate with the expectation that these values may be able to predict the ability of an azole substrate to undergo C–H aminoalkylation.

**Scheme 10:**
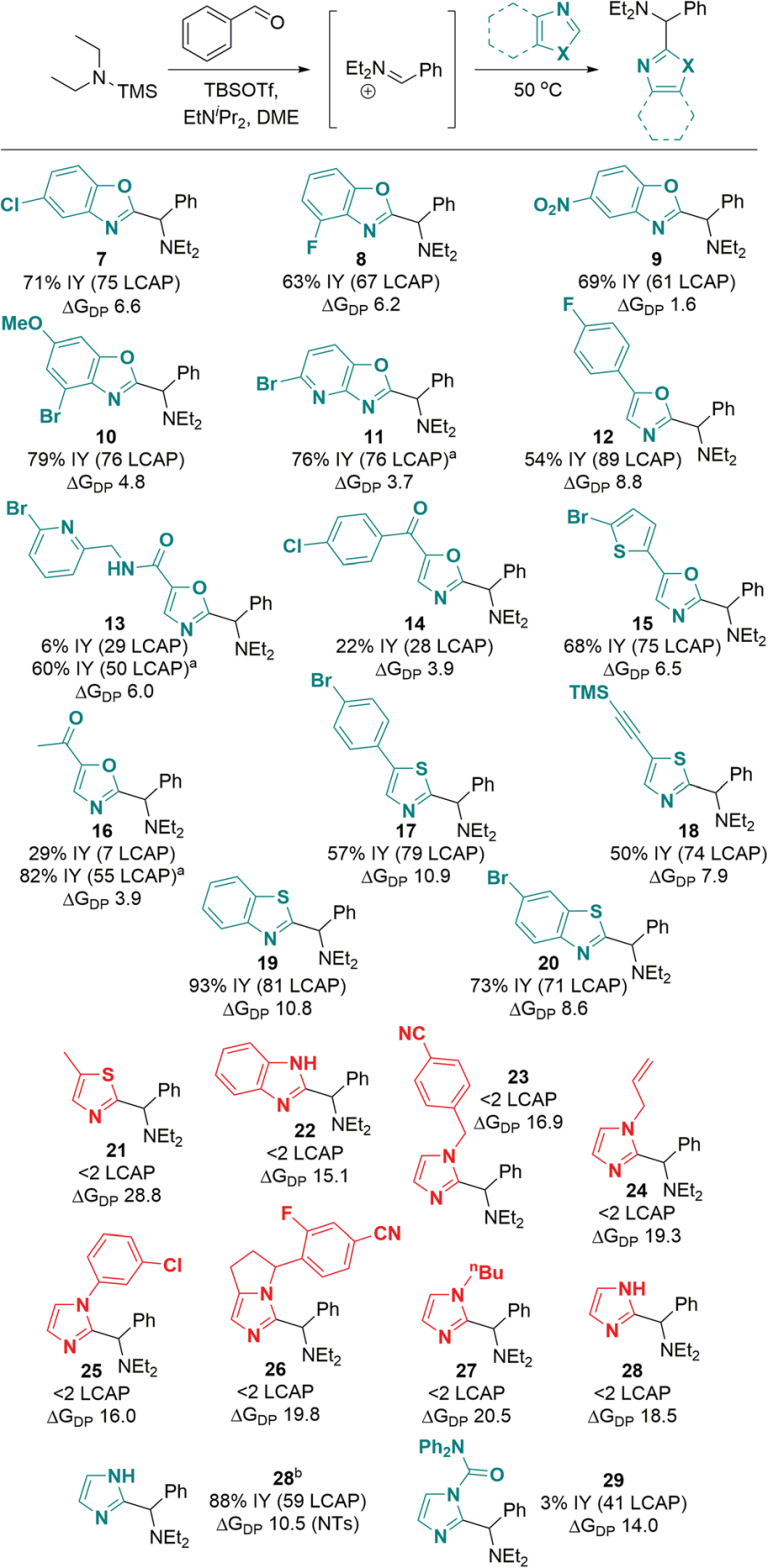
Azole substrate scope. Successful substrates are shown in cyan (Δ*G*_DP_ < 14 kcal mol^−1^); unsuccessful substrates are shown in red (Δ*G*_DP_ > 14 kcal mol^−1^). Δ*G*_DP_ = free energy of deprotonation (in kcal mol^−1^). Conditions: azole (1.0 eq.), TBSOTf (0.416 mL, 1.813 mmol, 3.0 eq.), TMS–NEt_2_ (1.25 eq.), PhCHO (1.0 eq.), EtN^i^Pr_2_ (1.0 eq.), N_2_, 50 °C, 18 h. ^a^ Modification for substrates with acidic C–H or N–H bonds: TBSOTf (4.0 eq.), EtN^i^Pr_2_ (3.0 eq.); workup for desilylation: 8 eq. KF, pyridine. ^b^ From *N*-tosyl imidazole; tosyl hydrolysis after reaction: pyridine (2.0 mL), water (0.5 mL), 50 °C, 3 h. See ESI† for details of computational methods. M06-2X/def2TZVPP/SMD(ether)//M06-2X/6-31G(d)/SMD(ether).

Excitingly, a broad variety of azole substrates successfully underwent C–H aminoalkylation (Scheme 10), including benzoxazoles with electron-withdrawing and electron-donating substituents (**7** to **10**), oxazoles with various functionalized substituents (**11** to **16**), thiazoles (**17**, **18**), and benzothiazoles (**19**, **20**). Interestingly, imidazoles and benzimidazoles (**22** to **28**) afforded only traces of products, regardless of the substitution pattern at the N atom (H, alkyl, aryl, benzyl, allyl).

When considering the calculated free energies of deprotonation Δ*G*_DP_ for the respective silylated substrates in combination with the experimental findings, a clear pattern emerges: all azoles that show very low conversion (<2 LCAP; products **21** to **28**) exhibit values for Δ*G*_DP_ that are larger than 14 kcal mol^−1^. Furthermore, the deprotonation barrier of the least activated silylated 5-methylthiazole (product **21**) is 4.1 kcal mol^−1^ higher than that of TBS–benzoxazole. This suggests that imidazoles and benzimidazoles as well as aryl- or alkyl-protected imidazoles are not reactive under the optimized conditions due to unfavorable deprotonation kinetics and thermodynamic equilibrium.

To circumvent this significant limitation in the azole scope, we considered activating protecting groups for imidazoles and benzimidazoles that may be able to shift the deprotonation equilibrium below the threshold energy of 14 kcal mol^−1^. Indeed, we discovered that urea derivative **29** (Scheme 10; Δ*G*_DP_ 14.0 kcal mol^−1^) afforded reasonable crude yields of the desired coupling product (41 LCAP); however, isolation by column chromatography on silica led to decomposition of the product, resulting in a low isolated yield (3%). This observation suggests that electron-withdrawing protecting groups (such as acyl groups) can activate imidazole or benzimidazole substrates for C–H deprotonation and subsequent aminoalkylation.

**Scheme 11:**
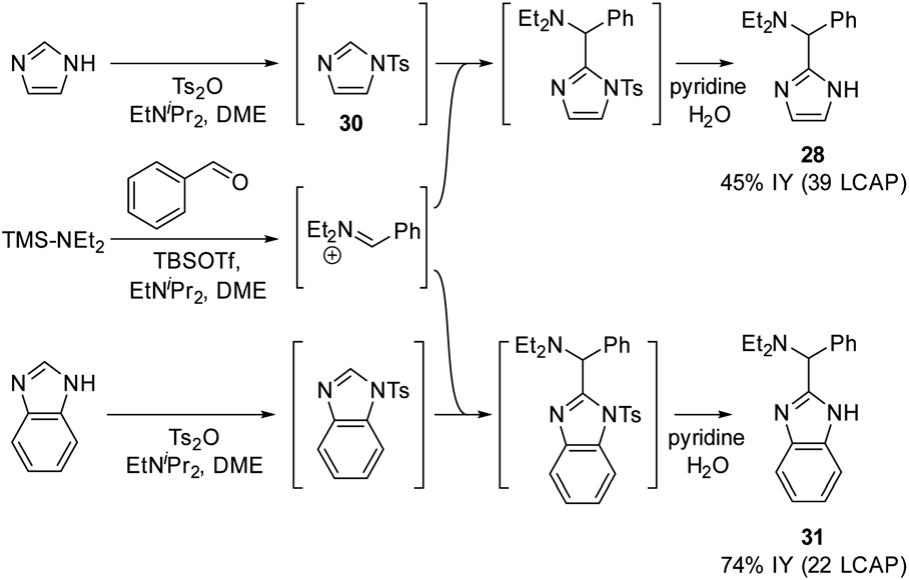
*In situ* tosylation strategy.

In an extension of the activating protecting group strategy, subjecting *N*-tosyl imidazole (Δ*G*_DP_ 10.5 kcal mol^−1^) to the standard conditions afforded an 88% isolated yield of the desired imidazole product **28**. To enable analogous reactivity with more diverse imidazoles and benzimidazoles that might not be as easily available as tosylated precursors, we further developed a procedure (Scheme 11) combining *in situ* tosylation, C–H aminoalkylation, and detosylation in one pot. With this sequence, imidazole and benzimidazole coupling products **28** and **31** were synthesized, starting from unprotected imidazole and benzimidazole substrates in moderate to good yields (45% and 74%, respectively) without the need to isolate the *N*-tosyl intermediates. These results demonstrate that the use of electron-withdrawing protecting groups can be implemented successfully to enable the C–H aminoalkylation of less activated azoles.

### Predicting amine reactivity *via* distortion analysis of the transition state

Having explored different azole substrates in C–H aminoalkylation, we set out to elucidate how structural features of the amine component may affect the reactivity. Given that the deprotonation barrier of a given silylated azole can be overcome, and the resulting silylated carbene is sufficiently stabilized, such as in the case of benzoxazole, comparing the activation energies of the C–C bond forming step across various electophiles should enable predictions of reactivity patterns.

**Scheme 12:**
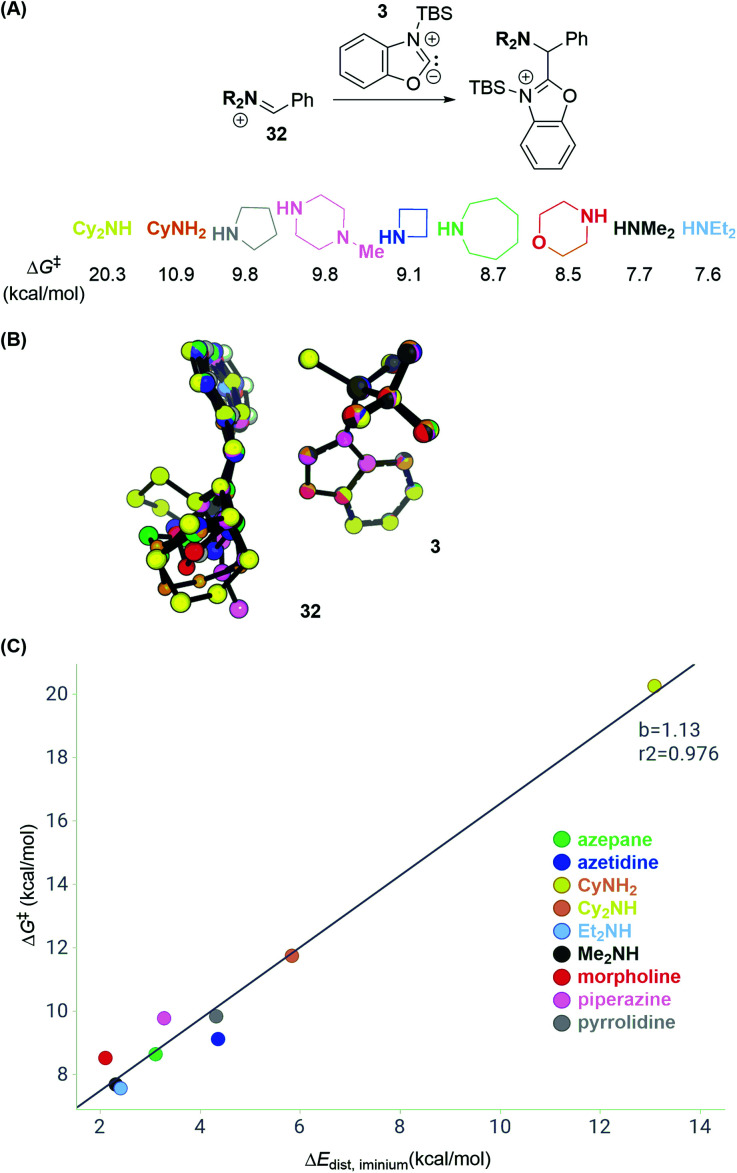
Distortion–interaction analysis of reactions with iminium electrophiles. (A) Transition state energies Δ*G*^‡^ for series of iminium electrophiles. (B) Overlay of transition state structures. (C) Distortion energies Δ*E*_dist_*versus* Δ*G*^‡^ of C–C bond formation. See ESI† for details of computational methods. M06-2X/def2TZVPP/SMD(ether)//M06-2X/6-31G(d)/SMD(ether).

We first calculated transition state energies for reactions between a series of differently substituted iminium electrophiles (**32**; Scheme 12A) and carbene intermediate **3** (Table S2†). The substitution patterns for **32** were chosen to reflect common amine cores in pharmaceutical chemistry and to vary between low and high steric demand. DFT calculations predict, accordingly, activation energies that span from 7.6 to 20.3 kcal mol^−1^ (with respect to the silylated carbene and iminium). Interestingly, the C–C bond forming step for the dicyclohexyl iminium electrophile is 12.6 kcal mol^−1^ higher than that for diethyl iminium, which is higher than the deprotonation barrier in the reaction pathway (Scheme 8D).

A distortion–interaction analysis^14^ (Scheme 12B and C) reveals that the total distortion energies Δ*E*_dist_ for the components in each reaction are unique and correlate with the activation free energies Δ*G*^‡^ of C–C bond formation. In contrast, the values of the interaction energy term between **3** and each of the iminium intermediates **32** lie within 0.5 kcal mol^−1^. Scheme 12B depicts an overlay of all computed transition state structures. It shows that carbene intermediate **3** remains in a very similar geometry in all computed transition state structures (Δ*E*_dist,_**3** = 0.1–0.7 kcal mol^−1^). In contrast, the iminium electrophiles distort away from their preferred planar geometry to avoid potential steric clashes with **3** going from reactant to the transition state (see Fig. S11† for a breakdown of the total distortion energies). These data suggest that sterically congested iminium electrophiles such as the electrophile obtained by condensation of PhCHO and Cy_2_NH may not undergo C–C bond formation due to the high distortion energy necessary to achieve the transition state.

### Scope and limitations: amine scope I

**Scheme 13:**
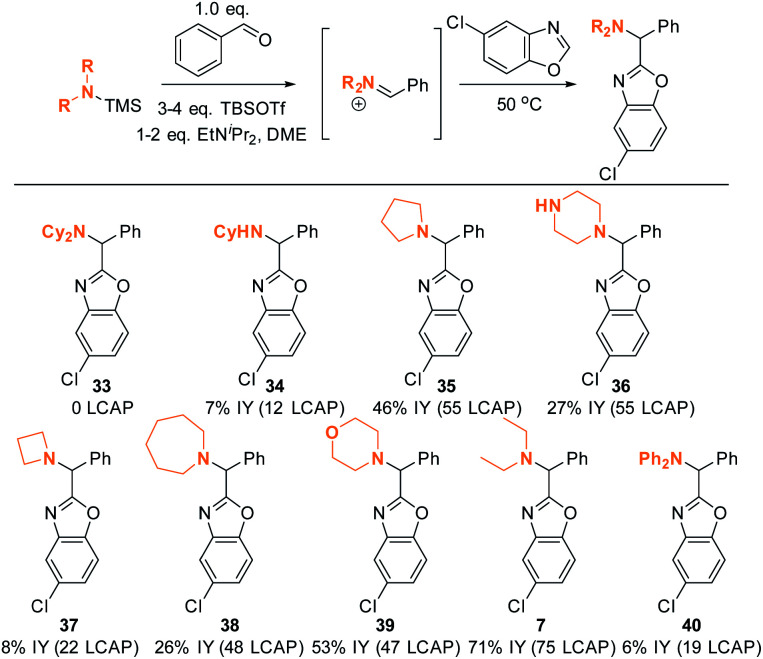
Amine scope I. Conditions: see ESI.†

The DFT analysis discussed in the previous paragraph predicts that the steric environment of the amine coupling partner will majorly influence the success of C–H aminoalkylation. We tested this hypothesis experimentally by employing different amine substrates under the optimized conditions (Scheme 13). As predicted, Cy_2_NH is the least reactive substrate: not even traces of C–H aminoalkylation product **33** were observed in agreement with the DFT analysis above (Δ*G*^‡^ > 20 kcal mol^−1^). Generally, lower-yielding product formation (**34**, **36**, **37**, **38**, **40**; 12 to 55 LCAP; 6 to 27% IY) was observed in reactions in which the silylated amine was formed *in situ* from the amine precursor (see ESI† for details). In contrast, products were formed in moderate to good yields in reactions employing preformed, isolated silylated amines (**35**, **39**, **7**; 47 to 75 LCAP; 45% to 75% IY).

### Scope and limitations: amine scope II *via* parallel medicinal chemistry workflow

Having shown that a broad range of amines can be used as reactants, we decided to pressure-test the robustness of the reaction protocol by employing it in a standard parallel medicinal chemistry (PMC) workflow. Success in this workflow would demonstrate the ability to synthesize a large variety of compounds in parallel, and allow broad and rapid interrogation of chemical space in order to improve absorption, distribution, metabolism, and excretion (ADME) properties. A standard PMC workflow encompasses (1) reaction setup; (2) reaction quench/minimal workup; and (3) automated, mass-selective purification. All these steps are typically performed in parallel.

**Scheme 14:**
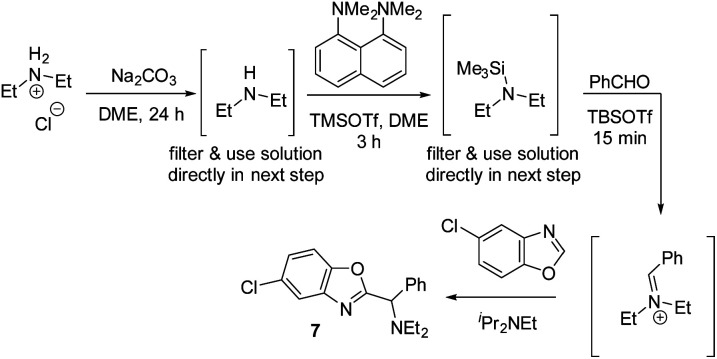
Non-aqueous free-basing conditions allowing use of amine HCl salts in azole C–H aminoalkylation.

This presented an additional challenge for reaction development: the majority of complex amines are only commerically available as their corresponding HCl salts and direct use of these salts did not afford any product under standard conditions. We hypothesized that chloride ions are detrimental to the reactivity, as chloride might react with TBSOTf and form less reactive TBSCl. Therefore, we developed a protocol that first removes chloride under non-aqueous conditions (Scheme 14, first step). After filtration, the resulting solution of the free amine can then be directly used for silylation and iminium ion formation. Using Et_2_NH_2_Cl as the amine with this protocol, 59% of the desired C–H aminoalkylation product **7** was isolated, demonstrating that employing HCl salts directly is feasible with this modified protocol.

**Scheme 15:**
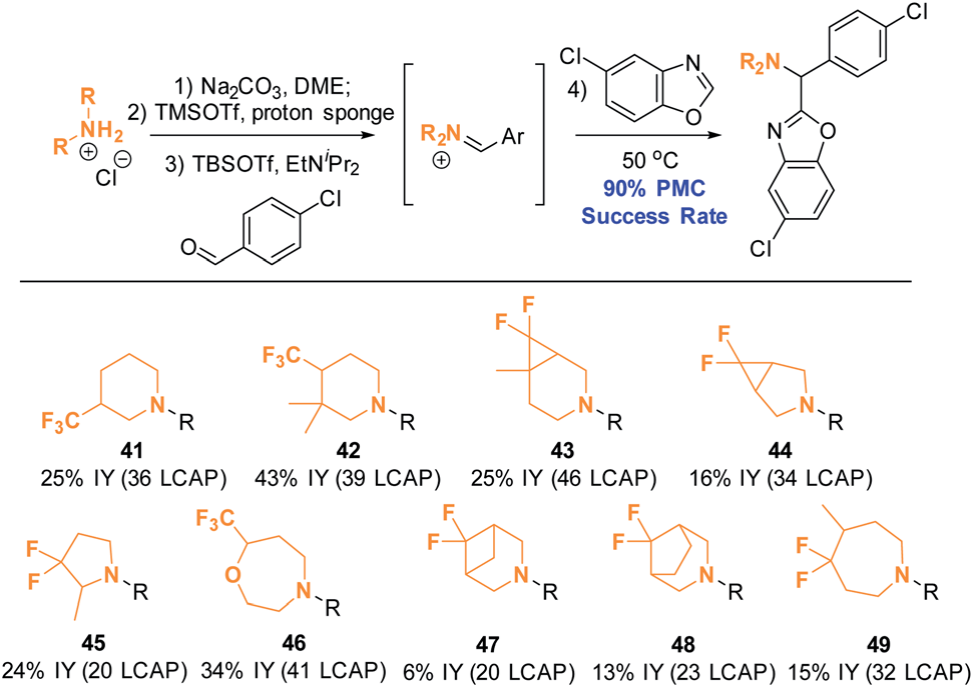
Amine scope II: azole C–H aminoalkylation in parallel. Conditions: see ESI.† All reactions were conducted on a 0.18 mmol scale. Products were isolated as TFA salts after automated preparative HPLC purification.

Ten complex amines that are commercially available as HCl salts were thus subjected to the PMC workflow under the modified conditions (Scheme 15). The amine structures depicted were chosen to reflect structures of interest for medicinal chemistry, as they are highly saturated^15^ and incorporate fluorinated moiteties.^16^ All employed monomers successfully formed product; nine out of ten of these products were isolated in ≥95% purity (suitable for primary assay screenings) by mass-guided, automated preparative HPLC. This is equivalent to an excellent success rate of 90%, as calculated by the number of amines yielding the intended product divided by the total number of amines employed. This result suggests that the established conditions will be applicable for synthesizing compound arrays of interest to the medicinal chemistry community.

### Scope and limitations: aldehyde scope

**Scheme 16:**
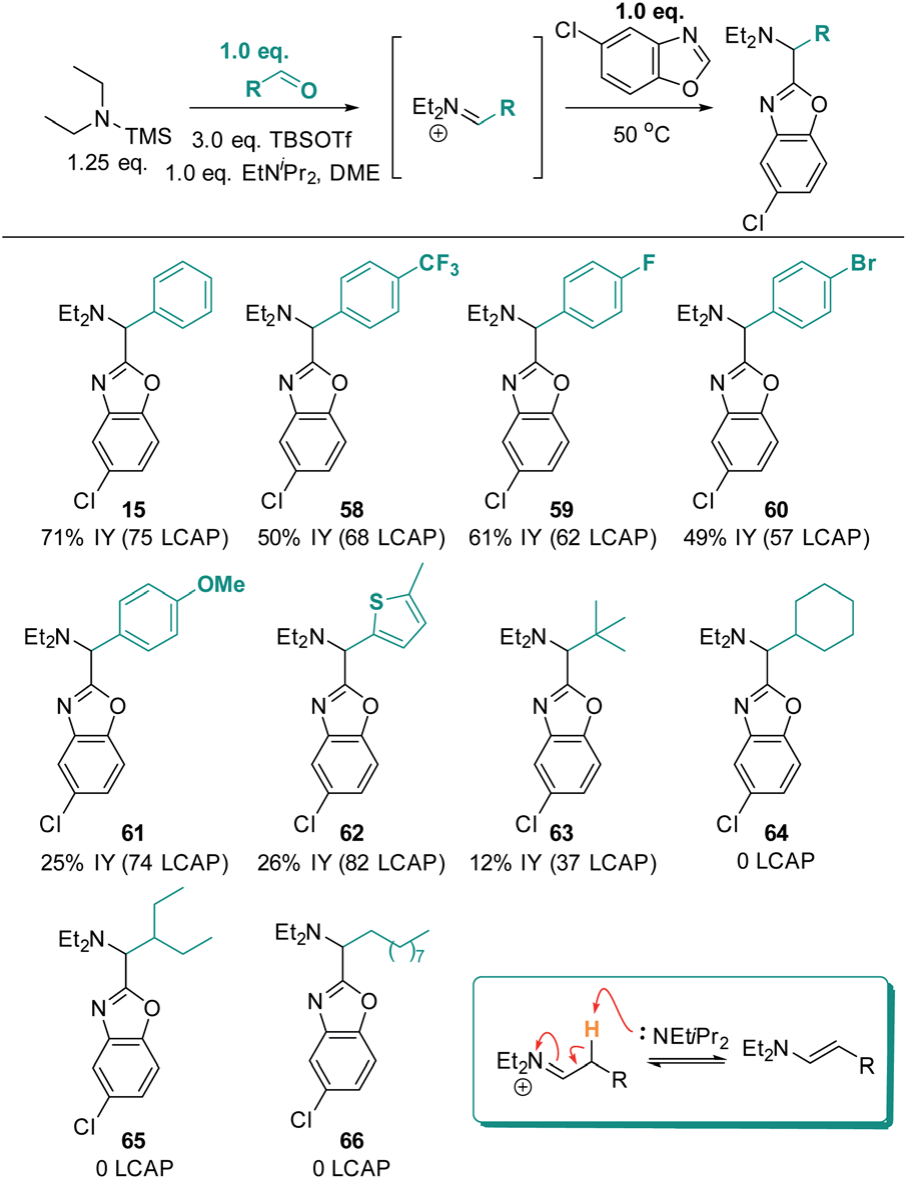
Aldehyde scope. Conditions: azole (1.0 eq.), TBSOTf (0.416 mL, 1.813 mmol, 3.0 eq.), TMS–NEt_2_ (1.25 eq.), PhCHO (1.0 equiv.), EtNiPr_2_ (1.0 equiv.), N_2_, 50 °C, 18 h.

Finally, the robustness of the C–H aminoalkylation protocol was tested with different aldehyde coupling partners (Scheme 16). All tested aromatic aldehydes, ranging from electron-poor to electron-neutral to electron-rich, afforded the desired products **15** and **58** to **61**. Generally, all LCAPs for these products were >55, indicating efficient reactivity, and good isolated yields were obtained. In the case of the MeO-substituted product **61**, low isolated yields were obtained despite high LCAP (74), indicating high crude yields.

Excitingly, a thiophene-derived aldehyde was also employed successfully, forming product **62** in 82 LCAP (26% IY). Furthermore, pivaldehyde was successfully reacted to afford **63**, showcasing that aliphatic aldehydes are generally also reactive under the established standard conditions. In contrast, aliphatic aldehydes with acidic C–H bonds in β-position to the *in situ* formed iminium intermediate do not form any product (**64** to **66**). This can be rationalized by the ability of the iminium intermediates to undergo deprotonation to the enamine products (shown in insert in Scheme 16). This would remove both the electrophile and the reactive base from the reaction solution and is thus expected to be detrimental for C–H aminoalkylation. Indeed, analysis of a crude reaction mixture (for details see ESI†) detects evidence for the formation of enamine side products.

## Summary and conclusions

In summary, this manuscript details the high-throughput optimization of a metal-free, mild C–H aminoalkylation method for azoles. Mechanistically-driven, DFT-supported expansion of the substrate scope provides a novel approach to directly functionalize heterocycles that are of tremendous importance to the synthesis of biologically active compounds. Our computational studies support the concerted action of a Lewis acid/base pair (TBSOTf/EtN^i^Pr_2_) to enable the key bond-forming step; both components have experimentally been established to be necessary for efficient reactivity. Combined computational and experimental explorations of the azole scope provide insights into suitable azole acidity ranges that predict successful reactivity. Based on this understanding, a strategy to overcome low acidities in imidazole and benzimidazole substrates was devised (*via in situ* tosylation/detosylation). DFT calculations further suggest rationales for substrate limitations: bulky iminium electrophiles lead to an increase in the activation barrier due to the required distortion of the iminium electrophile to achieve the transition state geometry; unactivated imidazoles and benzoxazoles render the azole deprotonation equilibrium unfavorable. Finally, the suitability of the method for parallel medicinal chemistry workflows has been demonstrated with a 90% success rate, providing confidence that the established conditions are robust and can be transferred between chemists without loss of reactivity.

Overall, this manuscript outlines a new strategy to diversify azoles *via* Lewis-acid mediated C–H functionalization under much milder conditions than those typically employed with transition metal catalysts.^3–5^ Moreover, the protocol employs both acidic and basic components, which is another distinguishing feature from more common, base-promoted azole C–H functionalization approaches.^17^ Importantly, this leads to broad functional group tolerance, which is typically inaccessible through other direct azole functionalizations approaches proceeding through catalytic or stoichiometric metalation.^3–6^

## Experimental

### General procedure for C–H aminoalkylation

*N*-Tosyl-1*H*-imidazole (1.0 equiv.) was weighed into an oven-dried vial and introduced into the glovebox. *tert*-Butyldimethylsilyl trifluoromethanesulfonate (0.416 mL, 1.81 mmol, 3.0 equiv.), *N*,*N*-diethyl-1,1,1-trimethylsilanamine (1.0 equiv.), and benzaldehyde (1.0 equiv.) were mixed in a separate oven-dried vial in the glovebox in 1,2-dimethoxyethane (1.0 mL) and stirred for 15 min. Then, the resulting solution was added to the vial containing *N*-tosyl imidazole. EtN^i^Pr_2_ (1.0 equiv.) was added. The vial was sealed, removed from the glovebox, and heated on a hotplate to 50 °C for 18 h. To hydrolyze the tosyl protecting group (only for tosyl-protected azole substrates), pyridine (2.0 mL) and water (0.50 mL) were added and the reaction was stirred at 50 °C for 3 h. All volatiles were removed, and the residue was purified by silica gel or reverse phase chromatography.

## Author contributions

Conceptualization (MHE, CQH), data curation (all authors), formal analysis (all authors), investigation (all authors), methodology (MHE, CQH), software (CQH), supervision (MHE), validation (all authors), vizualization (MHE, CQH, AAS), writing – original draft (MHE, CQH), writing – review & editing (all authors).

## Conflicts of interest

There are no conflicts to declare.

## Supplementary Material

SC-012-D0SC06868C-s001

## References

[cit1] (a) Attenni B., Ferrigno F., Jones P., Ingenito R., Kinzel O., Llauger Bufi L., Ontoria J. M., Pescatore G., Rowley M., Scarpelli R. and Schultz C., WO2006061638, 2006

[cit2] Erkkilä A., Majander I., Pihko P. M. (2007). Chem. Rev..

[cit3] Lewis J. C., Bergman R. G., Ellman J. A. (2008). Acc. Chem. Res..

[cit4] Do H.-Q., Daugulis O. (2007). J. Am. Chem. Soc..

[cit5] Amaike K., Muto K., Yamaguchi J., Itami K. (2012). J. Am. Chem. Soc..

[cit6] He T., Li H., Li P., Wang L. (2011). Chem. Commun..

[cit7] Heinz C., Lutz J. P., Simmons E. M., Miller M. M., Ewing W. R., Doyle A. G. (2018). J. Am. Chem. Soc..

[cit8] Jia Z., Ramstad T., Zhong M. (2001). Electrophoresis.

[cit9] Crampton M. R., Robotham I. A. (1997). J. Chem. Res., Synop..

[cit10] Kricheldorf H. R. (1974). Makromol. Chem..

[cit11] Khurana J. M., Nand B., Saluja P. (2010). Tetrahedron.

[cit12] Stephan D. W. (2016). Science.

[cit13] Yetra S. R., Patra A., Biju A. T. (2015). Synthesis.

[cit14] Green A. G., Liu P., Merlic C. A., Houk K. N. (2014). J. Am. Chem. Soc..

[cit15] Lovering F., Bikker J., Humblet C. (2009). J. Med. Chem..

[cit16] Gillis E. P., Eastman K. J., Hill M. D., Donnelly D. J., Meanwell N. A. (2015). J. Med. Chem..

[cit17] Patel U. N., Jain S., Pandey D. K., Gonnade R. G., Vanka K., Punji B. (2018). Organometallics.

