# Supplementary material for: Lewis acid mediated, mild C–H aminoalkylation of azoles via three component coupling
Source: Chem Sci. 2021 Feb 5;12(11):3890–7. doi: 10.1039/d0sc06868c (PMC8179430; doi:10.1039/d0sc06868c)
Supplement: SC-012-D0SC06868C-s001 [file SC-012-D0SC06868C-s001.pdf]

Electronic Supplementary Material (ESI) for Chemical Science.
This journal is © The Royal Society of Chemistry 2021

Lewis Acid Mediated, Mild C-H Aminoalkylation of Azoles via Three Component Coupling

Marion H. Emmert,*‡a Cyndi Qixin He,*‡b Akshay A. Shah‡c, and Stephanie Felten‡ a

[a]*Process Research & Development, MRL, Merck & Co. Inc, 770 Sumneytown Pike, West Point, PA 19486.*

[b]*Computational and Structural Chemistry, MRL, Merck & Co. Inc, 126 E Lincoln Ave, Rahway, NJ 07065.*

[c]*Discovery Chemistry, MRL, Merck & Co. Inc, 770 Sumneytown Pike, West Point, PA 19486.*

Table of Contents

Materials and Methods 5

Reaction Optimization: Ni/Ligand/Solvent Screen 6

Reaction Time Course in the Absence of Ni Catalysts 8

Reaction Optimization: TBSOTf, Et_2N-TMS, and benzoxazole loading 9

Reaction Optimization: Influence of Added Base 11

General Procedure 1: Amine scope 13

General Procedure 2: Amine, Aldehyde, and Azole Substrate Scope 14

Modifications for Substrates with Acidic NH or CH bonds 14

Amine Scope 1 15

5-Chloro-2-(morpholino(phenyl)methyl)benzo[d]oxazole 15

5-Chloro-2-(phenyl(pyrrolidin-1-yl)methyl)benzo[d]oxazole 17

5-Chloro-2-((4-methylpiperazin-1-yl)(phenyl)methyl)benzo[d]oxazole 19

2-(Azepan-1-yl(phenyl)methyl)-5-chlorobenzo[d]oxazole 21

2-(Azetidin-1-yl(phenyl)methyl)-5-chlorobenzo[d]oxazole 23

N-((5-Chlorobenzo[d]oxazol-2-yl)(phenyl)methyl)cyclohexanamine 25

N-((5-Chlorobenzo[d]oxazol-2-yl)(phenyl)methyl)-N-phenylaniline 27

Amine with low reactivity (<2 LCAP) under conditions of General Procedure 1 29

Modified Conditions for Amine-HCl Salts as Substrates 30

Amine Scope II Via Parallel Medicinal Chemistry 31

General Procedure 3 31

5-Chloro-2-((4-chlorophenyl)(3-(trifluoromethyl)piperidin-1-yl)methyl)benzo[d]oxazole 32

5-Chloro-2-((4-chlorophenyl)(3,3-dimethyl-4-(trifluoromethyl)piperidin-1-yl)methyl)benzo[d]oxazole 35

5-Chloro-2-((4-chlorophenyl)(7,7-difluoro-6-methyl-3-azabicyclo[4.1.0]heptan-3-yl)methyl)benzo[d]oxazole 38

5-Chloro-2-((4-chlorophenyl)(6,6-difluoro-3-azabicyclo[3.1.0]hexan-3-yl)methyl)benzo[d]oxazole 42

5-Chloro-2-((4-chlorophenyl)(3,3-difluoro-2-methylpyrrolidin-1-yl)methyl)benzo[d]oxazole 45

5-Chloro-2-((4-chlorophenyl)(7-(trifluoromethyl)-1,4-oxazepan-4-yl)methyl)benzo[d]oxazole 48

5-Chloro-2-((4-chlorophenyl)(6,6-difluoro-3-azabicyclo[3.1.1]heptan-3-yl)methyl)benzo[d]oxazole 51

5-Chloro-2-((4-chlorophenyl)(8,8-difluoro-3-azabicyclo[3.2.1]octan-3-yl)methyl)benzo[d]oxazole 54

5-Chloro-2-((4-chlorophenyl)(4,4-difluoro-5-methylazepan-1-yl)methyl)benzo[d]oxazole 57

Azole Scope 60
N-((5-Chlorobenzo[d]oxazol-2-yl)(phenyl)methyl)-*N*-ethylethanamine 60
N-Ethyl-*N*-((4-fluorobenzo[d]oxazol-2-yl)(phenyl)methyl)ethanamine 62
N-Ethyl-*N*-((5-nitrobenzo[d]oxazol-2-yl)(phenyl)methyl)ethanamine 65
N-ethyl-*N*-((5-(4-fluorophenyl)oxazol-2-yl)(phenyl)methyl)ethanamine 67
N-((1H-imidazol-2-yl)(phenyl)methyl)-*N*-ethylethanamine 70
2-((diethylamino)(phenyl)methyl)-*N*,*N*-diphenyl-1H-imidazole-1-carboxamide 72
N-ethyl-*N*-(phenyl(5-((trimethylsilyl)ethynyl)thiazol-2-yl)methyl)ethanamine 75
N-((6-bromopyridin-2-yl)methyl)-2-((diethylamino)(phenyl)methyl)oxazole-5-carboxamide 78
N-((5-(5-bromothiophen-2-yl)oxazol-2-yl)(phenyl)methyl)-*N*-ethylethanamine 81
(4-chlorophenyl)(2-((diethylamino)(phenyl)methyl)oxazol-5-yl)methanone 83
1-(2-((diethylamino)(phenyl)methyl)oxazol-5-yl)ethan-1-one 85
Synthesis according to General Procedure 2 85
Crude UPLC/MS before hydrolysis 85
Crude UPLC/MS after hydrolysis 86
N-((5-(4-bromophenyl)thiazol-2-yl)(phenyl)methyl)-*N*-ethylethanamine 90
N-((6-Bromobenzo[d]thiazol-2-yl)(phenyl)methyl)-*N*-ethylethanamine 92
N-(benzo[d]thiazol-2-yl(phenyl)methyl)-*N*-ethylethanamine 94
N-((5-Bromooxazolo[4,5-b]pyridin-2-yl)(phenyl)methyl)-*N*-ethylethanamine 96
C-H aminoalkylation of imidazole and benzimidazole via *in-situ* tosylation 100
Imidazole: *N*-((1H-imidazol-2-yl)(phenyl)methyl)-N-ethylethanamine 100
Benzimidazole: *N*-((1H-benzo[d]imidazol-2-yl)(phenyl)methyl)-N-ethylethanamine 100
Azoles With Low Reactivity (<2 LCAP) 104
Aldehyde Scope 105
N-((5-Chlorobenzo[d]oxazol-2-yl)(4-fluorophenyl)methyl)-*N*-ethylethanamine 105
N-((5-Chlorobenzo[d]oxazol-2-yl)(4-methoxyphenyl)methyl)-*N*-ethylethanamine 110
N-((4-Bromophenyl)(5-chlorobenzo[d]oxazol-2-yl)methyl)-*N*-ethylethanamine 112
N-((5-Chlorobenzo[d]oxazol-2-yl)(4-(trifluoromethyl)phenyl)methyl)-*N*-ethylethanamine 114
N-((5-Chlorobenzo[d]oxazol-2-yl)(5-methylthiophen-2-yl)methyl)-*N*-ethylethanamine 116
1-(5-Chlorobenzo[d]oxazol-2-yl)-*N,N*-diethyl-2,2-dimethylpropan-1-amine 118
Aldehydes showing no product formation under conditions of General Procedure 2 120
Experimental data supporting enamine formation for aliphatic aldehydes with acidic β-C-H bonds.....121
Experiments in support of proposed mechanism: Observation of azole-TBS adduct and H/D exchange124

DFT Results..128

Proposed Mechanism..128

Computational Methods..129

Calculated Transition States for C-C Bond Formation Step..130

Insights into Azole Reactivity: Calculated Deprotonation Equilibria with Successful and Unsuccessful Substrates...132

Cartesian Coordinates for Transition State Structures in the Amine Scope...................................133

Materials and Methods

Reaction optimization was performed via high-throughput experimentation in 1000 uL glass vials. Analysis of crude reaction mixtures was performed by reverse-phase UPLCMS. Yields in reaction optimizations are provided in LCAP (Liquid Chromatography Area Percent) at 215 nm or are shown as calibrated yields using biphenyl as internal standard. Data obtained from parallel synthesis experiments and optimization were analyzed using Virscidian to quantify and export data.

Reaction Optimization: Ni/Ligand/Solvent Screen

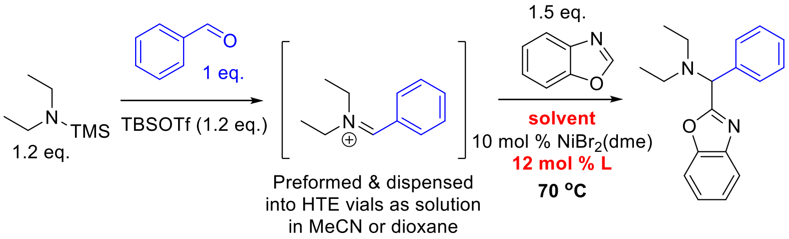

The reaction setup was prepared in analogy to Heinz, C.; Lutz, J. P.; Simmons, E. M.; Miller, M. M.; Ewing, W. R.; Doyle, A. G., Ni-Catalyzed Carbon-Carbon Bond-Forming Reductive Amination. *J. Am. Chem. Soc.* **2018**, *140* (6), 2292-2300.

A stock solution of $NiBr_2$(diglyme) (67.9 mg in a 22.0 mL of a 1:1 mixture of THF and MeOH) was prepared. 100 uL of this solution (corresponding to 0.309 mg, 1.00 µmol, 0.10 equiv.) was dispensed into each well of a parallel synthesis plate (array of 4 x 8 1000 uL vials) containing 1.2 µmol (0.12 equiv.) of various bidentate ligands. The solvent was evaporated.

Mixtures of reactants (181.9 uL Et_2N-$SiMe_3$, 81.3 uL benzaldehyde, 220.5 uL $Me_2{}^tBuSiOTf$) were prepared in 4 different solvents (MeCN, toluene, DMA, 1,2-dimethoxyethane; 2.37 mL each). Then, 1 mL of a stock solution of 286 mg of benzoxazole in each solvent was added to the respective reactant mixture. 50 uL of each reactant mix [corresponding to 1.744 mg *N*,*N*-diethyl-1,1,1-trimethylsilanamine (12.00 µmol, 1.20 equiv.), 1.061 mg benzaldehyde (10 µmol, 1.00 equiv.), 3.17 mg *tert*-butyldimethylsilyl trifluoromethanesulfonate (12.00 µmol, 1.2 equiv.), and 1.787 mg (15 µmol, 1.50 equiv.) benzoxazole were added to the reaction vials containing Ni/ligand complexes. The plates were sealed and heated to 70 °C in the glovebox and stirred on a tumble stirrer for 18 h.

Upon completion of the reaction time, the plates were taken off the stirrer and allowed to cool to room temperature. Each well was quenched with 500 uL (equivalent to 0.300 µmol standard) of 0.6 mM solution of di-*tert*-butylbiphenyl in MeCN (159.9 mg in 1.00 L MeCN). The plates were resealed, put on a shaker and then left to sit for 5 min to allow any solids to settle. Then, 150 uL of the supernatant solution were added to 550 uL MeCN in UPLC-ready trays. The solutions were analyzed by UPLC/MS.

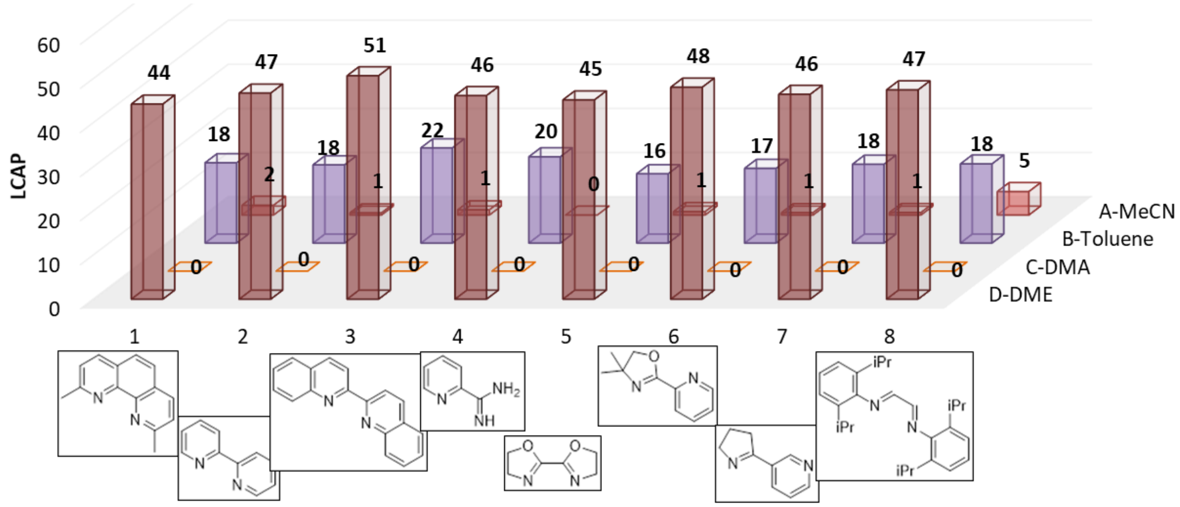

Scheme 1. LC Area Percent (LCAP) of product obtained in different solvent with 12 mol % N,N bidentate ligands. Wells in column 7 serve as control, as the base contained in these wells cannot ligate to a metal center in a bidentate fashion.

Reaction Time Course in the Absence of Ni Catalysts

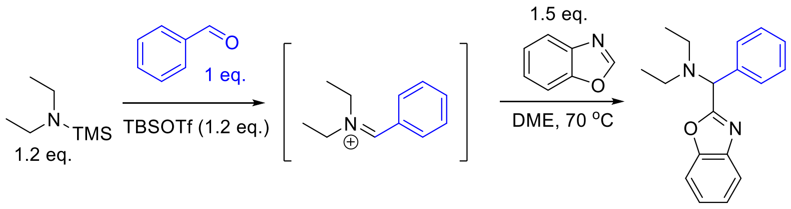

N,*N*-diethyl-1,1,1-trimethylsilanamine (214 µL, 1.129 mmol), *tert*-butyldimethylsilyl trifluoromethanesulfonate (259 µL, 1.129 mmol), and benzaldehyde (96 µL, 0.941 mmol) were mixed in 1,2-dimethoxyethane (2.5 ml) in the glovebox and stirred at room temperature for 5 minutes. The solution was added to a vial containing benzoxazole (168 mg, 1.411 mmol) and the mixture was sampled after stirring vigorously for 10 sec. Then, the mixture was placed on a hotplate and heated to 70 °C. The mixture was sampled at 15 min and 15 h.

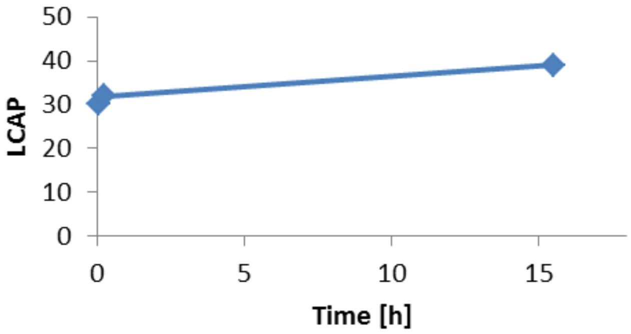

Scheme 2. LC Area Percent vs. Time in the absence of metal catalyst and ligands.

Reaction Optimization: TBSOTf, Et_2N-TMS, and benzoxazole loading

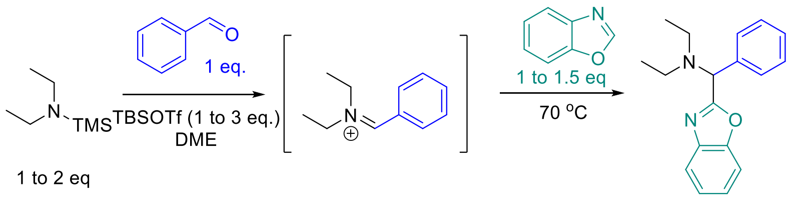

A stock solution of 297.1 mg benzaldehyde and 215.9 mg biphenyl (as internal standard) in 6.499 mL was prepared. 50 uL of this solution (corresponding to 1.00 equiv. benzaldehyde and 0.50 equiv. biphenyl) was added to each well of a 96-well plate.

A stock solution of benzoxazole (595.6 mg in 4.40 mL DME) was prepared.

3.8 uL (1.00 equiv.), 4.7 uL (1.25 equiv.), 5.7 uL (1.50 equiv.), or 7.6 uL (2.00 equiv.) Et_2N-$SiMe_3$ were added to the respective vials (see design in Figure 1). Then, 4.6 uL (1.00 equiv.), 5.7 uL (1.25 equiv.), 6.9 uL (1.50 equiv.), 8.0 uL (1.75 equiv.), 9.2 uL (2.00 equiv.), 11.5 uL (2.50 equiv.), or 13.8 uL (3.00 equiv.) TBS-OTf were added to the respective vials (see design in Figure 1). Then, 20 uL (1.00 equiv.), 25 uL (1.25 equiv.), 30 uL (1.50 equiv.), 40 uL (2.00 equiv.), or 50 uL (2.50 equiv.) of the benzoxazole stock solution were added to the respective vials.

The plate was sealed in the glovebox, removed, and placed on a tumblestirrer outside the glovebox. The plate was heated to 70 °C for 18 h. After the reaction time was complete, the plate was taken off the hotplate and allowed to cool to room temperature. 700 uL MeCN was added to each well; 10 uL of the corresponding solution was added to 800 uL of MeCN. The solutions were analyzed by UPLC/MS. Data analysis and quantification was performed using Virscidian.

benzoxazole		1 eq	1.25 eq	1.5 eq	2 eq	2.5 eq	1 eq	1.25 eq	1.5 eq	1.75 eq	2 eq	2.5 eq	3 eq	TBSOTf
Et2NTMS		1	2	3	4	5	6	7	8	9	10	11	12	
1 eq	A	1.25 eq. TBSOTf					1 eq. benzoxazole							
1.25 eq	B													
1.5 eq	C													
2 eq	D													
1 eq	E	1.0 eq. TBSOTf					1.5 eq. benzoxazole							
1.25 eq	F													
1.5 eq	G													
2 eq	H													

Figure 1. Design of high throughput optimization.

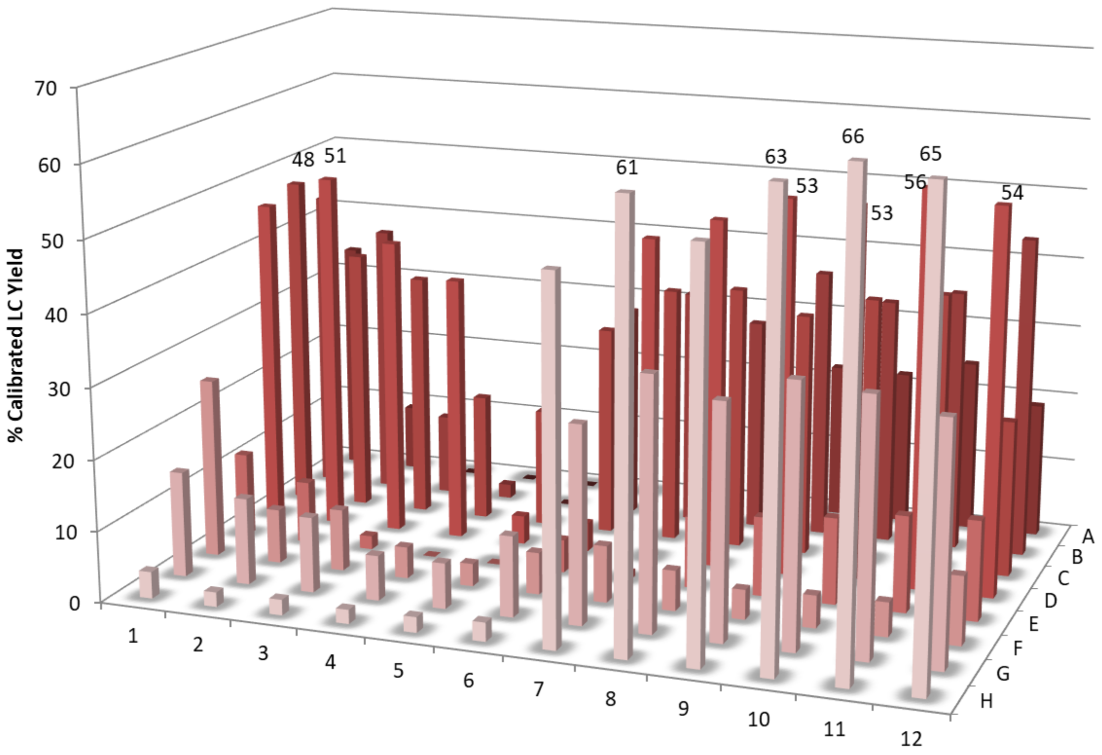

Figure 2. Calibrated LC yields for high-throughput optimization.

Reaction Optimization: Influence of Added Base

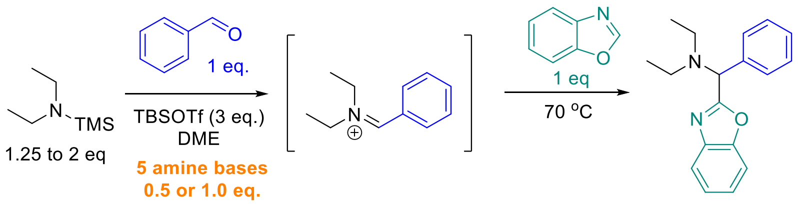

A stock solution of benzaldehyde (102.0 uL), biphenyl (77.1 mg), and TBS-OTf (689 uL) in DME (1.63 mL) was prepared. 50 uL of this solution (corresponding to 1.00 equiv. benzaldehyde, 0.50 equiv. biphenyl, and 3.00 equiv. TBSOTf) was added to each of the 24 wells (1000 uL vials) in a high-throughput plate.

4.8 uL (1.25 equiv.) or 7.6 uL (2.00 equiv.) $Et_2N\text{-}SiMe_3$ were added to the respective vials (see design in Figure 3).

A stock solution of benzoxazole (178.7 mg) in DME (1.32 mL) was prepared. 20 uL of this solution was added to each well (corresponding to 1.00 equiv. benzoxazole).

Then, 0.8 uL (0.50 equiv.) or 1.6 uL (1.0 equiv.) pyridine, 1.7 uL (0.50 equiv.) or 3.5 uL (1.00 equiv.) $^{i}PrNEt_2$, 1.5 uL (0.50 equiv.) or 3.0 uL (1.00 equiv.) DBU, 1.1 uL (0.50 equiv.) or 2.2 uL (1.00 equiv.) *N*-methyl morpholine, or 1.2 uL (0.50 equiv.) or 2.3 uL (1.00 equiv.) 2,6-lutidine were added to the respective vials (see design in Figure 3).

The plate was sealed in the glovebox, removed, and placed on a hotplate outside the glovebox. The plate was heated to 70 °C for 18 h. After the reaction time was complete, the reaction block was taken off the hotplate and allowed to cool to room temperature. 700 uL MeCN was added to each well; 10 uL of the corresponding solution was added to 800 uL of ACN. The solutions were analyzed by UPLC/MS. Data analysis and quantification was performed using Virscidian.

	no base	pyridine	EtNiPr2	DBU	N-Me morpholine	2,6-lutidine	
	1-no base	2-pyr	3-iPr2NEt	4-DBU	5-NMO	6-lutidine	
A-1.25 Et2NTMS, 0.5 base	34	16	57	56	52	52	1.25 eq.
B-1.25 Et2NTMS, 1.0 base	49	22	96	64	53	17	Et2NTMS
C-2.0 Et2NTMS, 0.5 base	0	64	16	53	39	53	2.0 eq.
D-2.0 Et2NTMS, 1.0 base	52	13	65	66	30	19	Et2NTMS

Figure 3. Base Screen Design and Calibrated LC Yields.

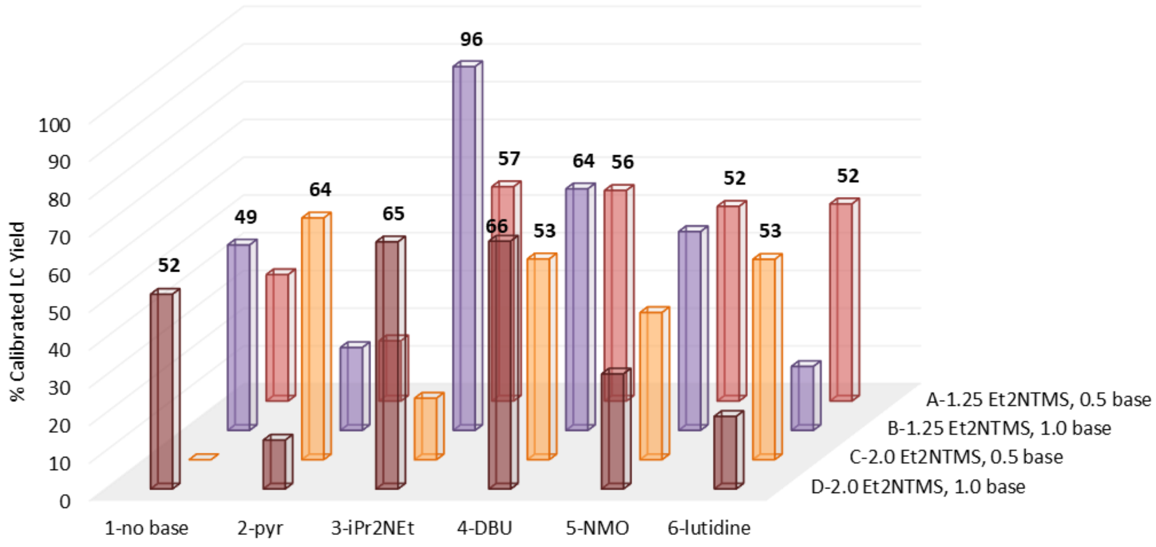

Figure 4. Calibrated LC yields obtained from base screening.

General Procedure 1: Amine scope

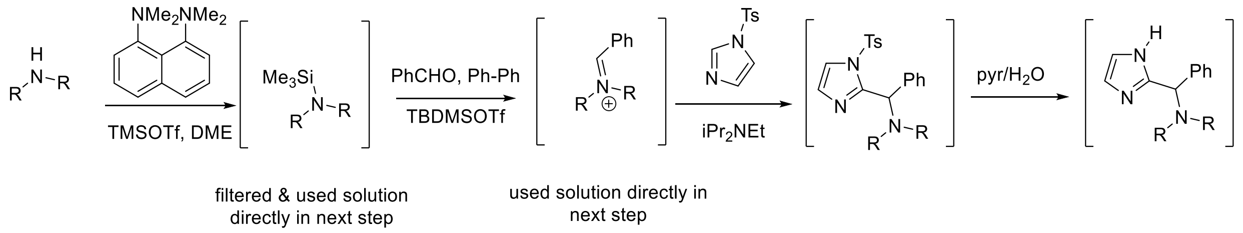

In the glovebox, an oven-dried 1-dram vial equipped with a stir bar was charged with *N*1,*N*1,*N*8,*N*8-tetramethylnaphthalene-1,8-diamine (72.7 mg, 0.339 mmol, 1.20 equiv.) and amine (1.25 equiv.). 1,2-Dimethoxyethane (1000 µl) was added and the mixture was stirred for 5 min. Trimethylsilyl trifluoromethanesulfonate (0.064 mL, 0.353 mmol, 1.25 equiv.) was added and the vial was capped and allowed to stir vigorously at room temperature for 2h.

The suspension was filtered through a kimwipe-clogged pipette-tip into another 1-dram vial. 1,2-dimethoxyethane (0.50 mL) was used to rinse the original reaction vial and the filtercake. Benzaldehyde (0.029 mL, 0.283 mmol, 1.00 equiv.) and *tert*-Butyldimethylsilyl trifluoromethanesulfonate (0.195 mL, 0.848 mmol) were added to the filtrate and the reaction mixture was stirred for 10 min, before adding *N*-ethyl-*N*-isopropylpropan-2-amine (1.00 or 3.00 equiv.). Then, the mixture was added to a vial containing the azole (1.00 equiv.). The vial was sealed, removed from the glovebox, and heated to 50 °C overnight.

The residue was cooled to room temperature, diluted with 10 mL water, and extracted with 10 mL ethyl acetate. The organic phase was purified by preparative HPLC.

General Procedure 2: Amine, Aldehyde, and Azole Substrate Scope

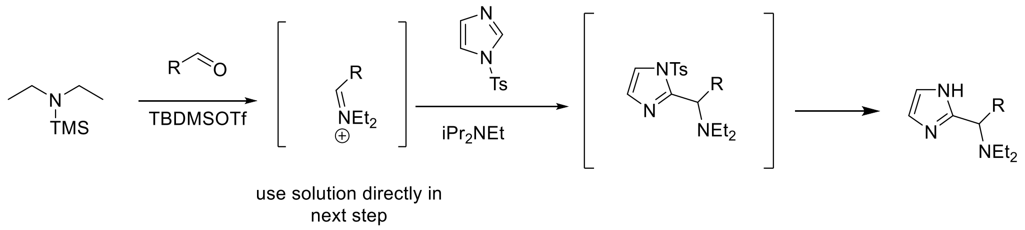

1-Tosyl-1H-imidazole (1.00 or 1.20 equiv.) was weighed into an oven-dried vial and brought into the glovebox. *tert*-Butyldimethylsilyl trifluoromethanesulfonate (0.416 mL, 1.813 mmol, 3.00 equiv.), *N*,*N*-diethyl-1,1,1-trimethylsilanamine (1.00 or 1.25 equiv.), and aldehyde (1.00 equiv.) were mixed in a separate oven-dried vial in the glovebox in 1,2-dimethoxyethane (1.0 mL) and stirred for 15 min. Then, this solution was added to the vial containing *N*-tosyl imidazole. *N*-ethyl-*N*-isopropylpropan-2-amine (1.00 equiv.) was added. The resulting solution was removed from the glovebox and heated on a hotplate to 50 °C overnight.

To hydrolyze the tosyl protecting group (only for tosyl-protected imidazole substrate), pyridine (2.0 mL) and water (0.50 mL) were added and the reaction was stirred at 50 °C for 3 h.

Modifications for Substrates with Acidic NH or CH bonds

Compounds were prepared according to the general procedures, but with several modifications:

1) 4.00 equiv. of TBSOTf were used
2) 2.00 equiv. or 3.00 equiv. of $^{i}Pr_2NEt$ were used
3) $^{i}Pr_2NEt$ was added to the reaction vial containing the azole before addition of the iminium/TBSOTf mixture
4) Workup was performed by pouring the mixture onto 4 to 8 equiv. solid KF, adding 0.5 mL pyridine, and stirring until all silylated products had disappeared (UPLC detection).
5) Workup was performed by diluting with 10 mL water, extracting with ethyl acetate, and preparative HPLC purification.

Amine Scope 1

5-Chloro-2-(morpholino(phenyl)methyl)benzo[d]oxazole

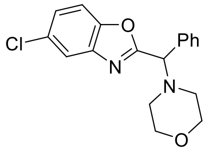

Chemical Formula: $C_{18}H_{17}ClN_2O_2$
Exact Mass: 328.10
Molecular Weight: 328.80

The title compound was prepared according to General Procedure 2 (reaction time 4 h).

Yield: 175 mg (53%); 47 LCAP before workup/column chromatography

^{1}H NMR (500 MHz, Methanol-d_4, ppm) δ 7.70 (s, 1H), 7.63 – 7.51 (m, 3H), 7.42 – 7.26 (m, 4H), 4.82 (s, 1H), 3.71 (s, 4H), 2.59 – 2.52 (m, 2H), 2.50 – 2.36 (m, 2H).

^{13}C NMR (126 MHz, Methanol-d_4, ppm) δ 168.4, 150.7, 142.9, 137.2, 131.3, 130.0, 129.9, 129.8, 126.9, 120.7, 113.0, 70.8, 67.8, 53.0.

HRMS: Calc. $C_{18}H_{18}ClN_2O_2^+$ [M+H$^+$] 329.1052; found 329.1046.

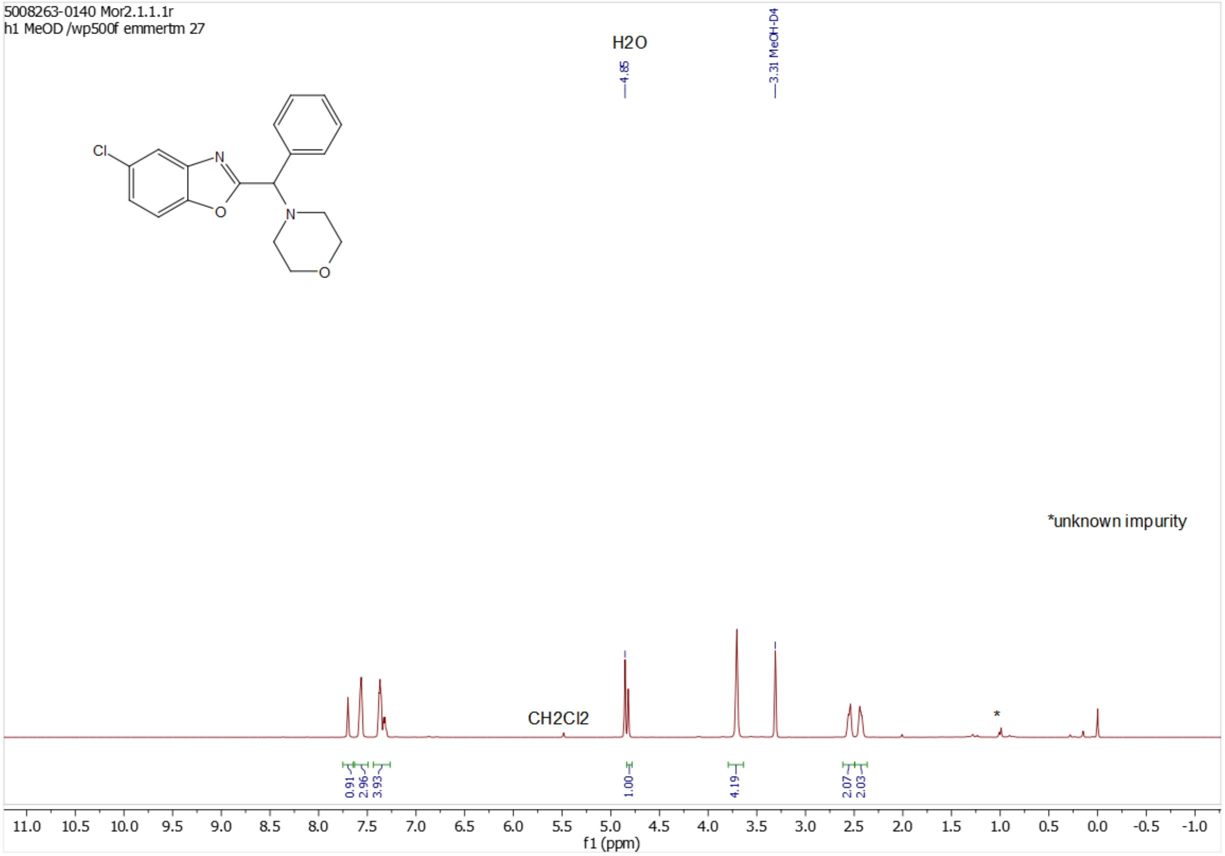

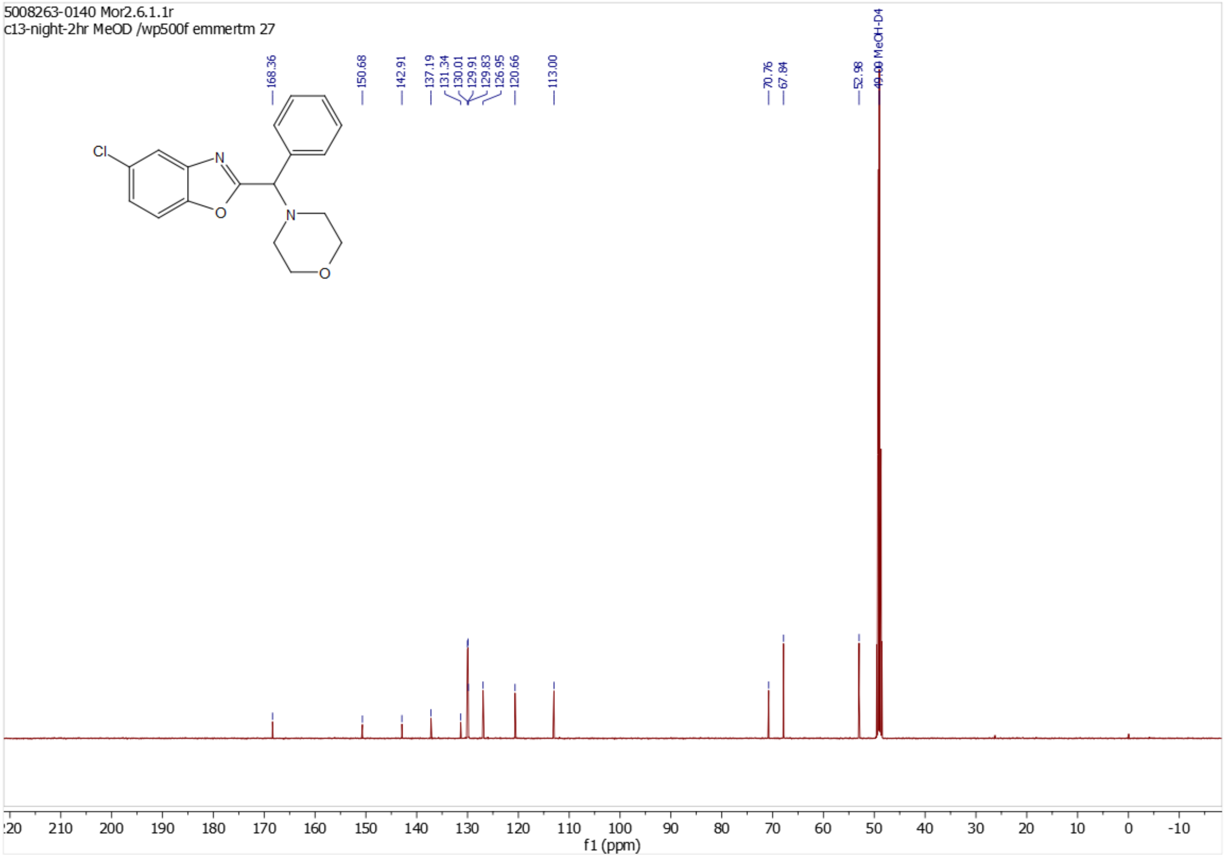

5008263-0140 Mor2.6.1.1r
c13-night-2hr MeOD /wp500f emmertm 27
168.36
150.68
142.91
137.19
131.34
130.01
129.91
129.83
126.95
120.66
113.00
70.76
67.84
52.98
49.00 MeOH-D4
Cl
N
O
N
O
f1 (ppm)

5-Chloro-2-(phenyl(pyrrolidin-1-yl)methyl)benzo[d]oxazole

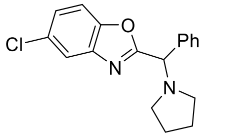

Chemical Formula: $C_{18}H_{17}ClN_2O$
Exact Mass: 312.10
Molecular Weight: 312.80

The title compound was prepared according to General Procedure 2 (reaction time 4 h).

Yield: 145 mg (46%); 55 LCAP before workup/column chromatography

^{1}H NMR (500 MHz, Methanol-d_4, ppm) δ 7.70 (d, J = 2.0 Hz, 1H), 7.61 – 7.55 (m, 3H), 7.36 (m, 4H), 4.81 (s, 1H), 2.62 (d, J = 16.4 Hz, 4H), 1.85 (t, J = 6.2 Hz, 4H).

^{13}C NMR (126 MHz, Methanol-d_4, ppm) δ 168.7, 150.6, 142.9, 138.4, 131.3, 129.9, 129.9, 129.6, 126.9, 120.7, 112.9, 69.8, 54.0, 24.2.

HRMS: Calc. $C_{18}H_{18}ClN_2O^+$ [M+H$^+$] 313.1102; found 313.1103.

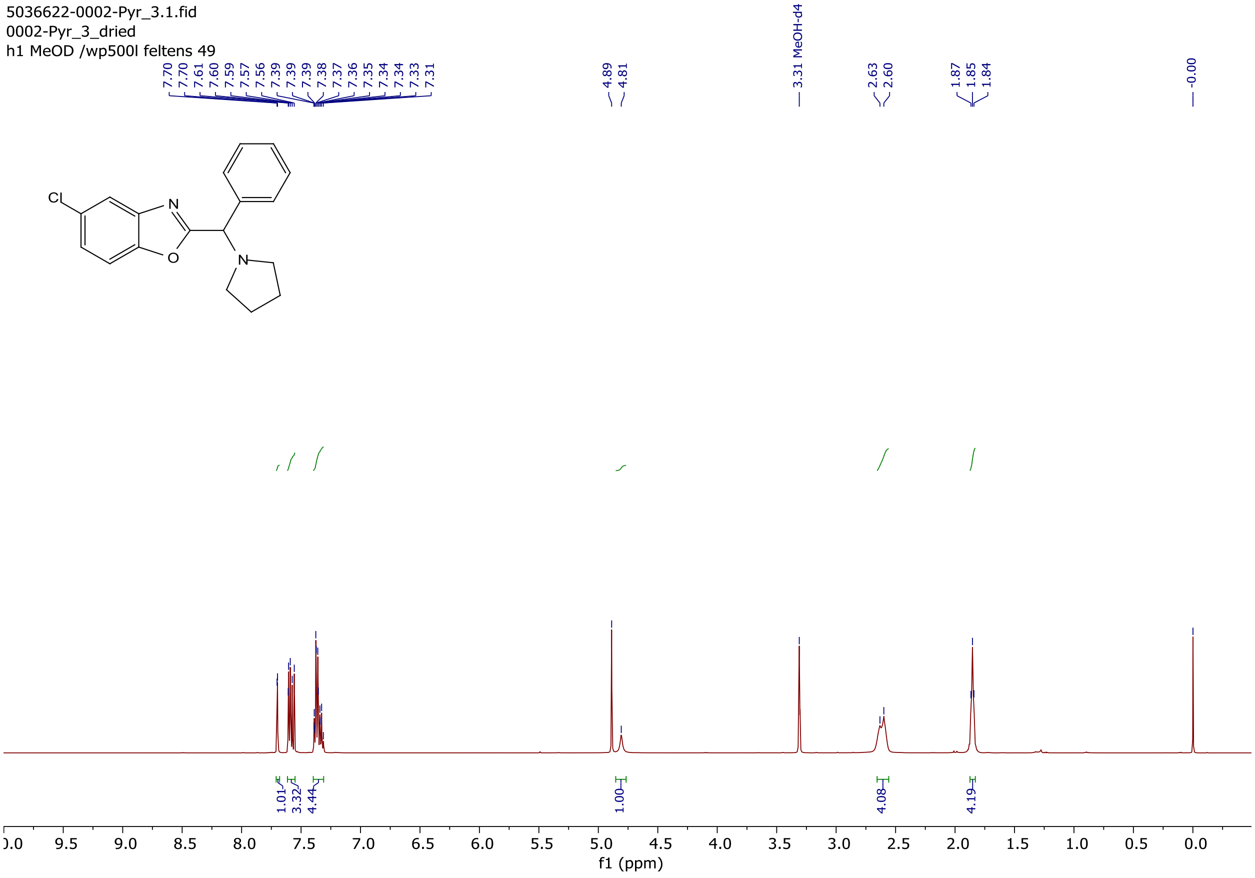

5036622-0002-Pyr-13C.1.fid
0002-Pyr-3-13C
c13-night-2hr MeOD /wp500l feltens 30

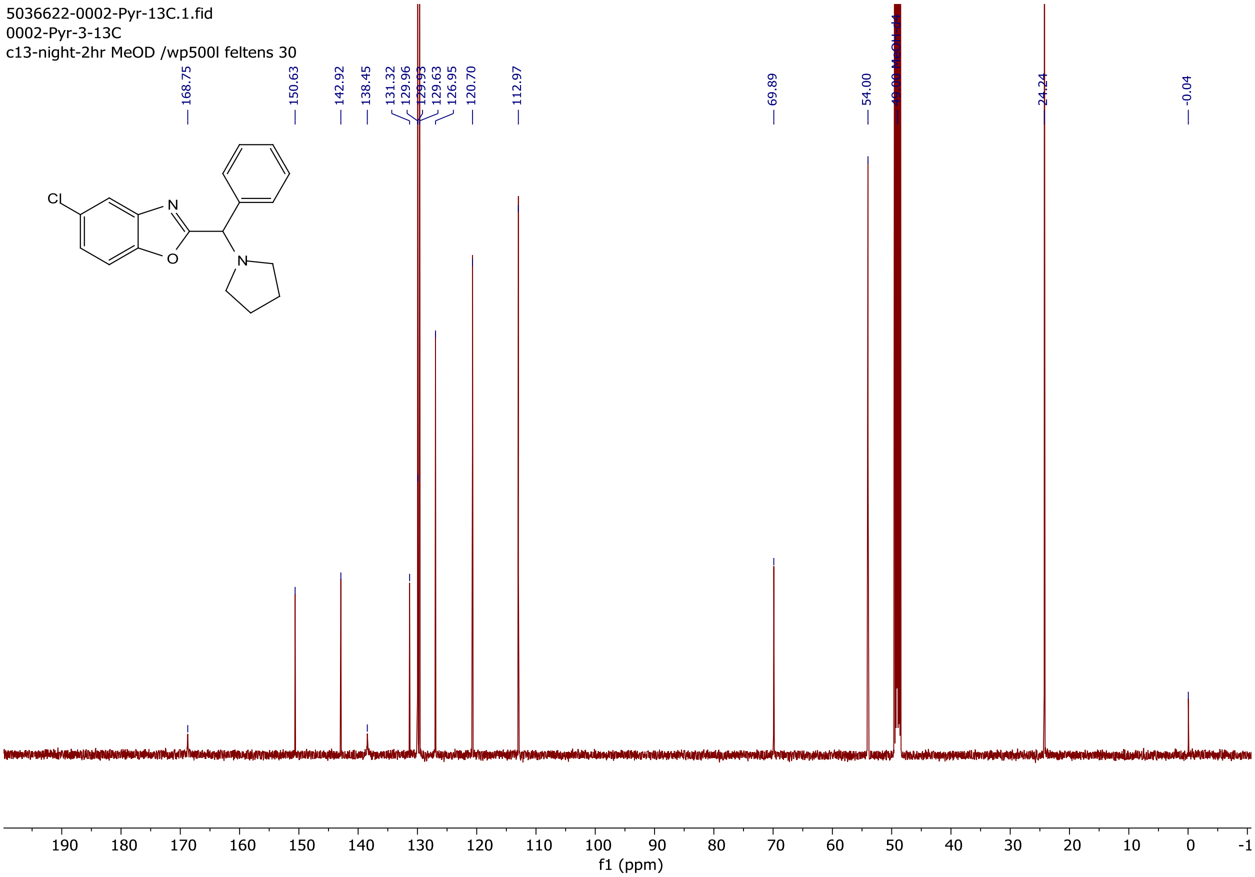

5-Chloro-2-((4-methylpiperazin-1-yl)(phenyl)methyl)benzo[d]oxazole

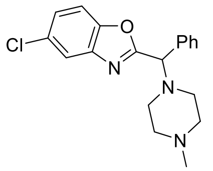

Chemical Formula: $C_{19}H_{20}ClN_3O$
Exact Mass: 341.1295
Molecular Weight: 341.8390

The title compound was prepared according to General Procedure 1 (reaction time 20 h).

Yield: 150 mg (27%); 55 LCAP before workup/ Prep-HPLC

^{1}H NMR (300 MHz, Chloroform-d, ppm) δ 7.68 (d, *J* = 2.1 Hz, 1H), 7.59 – 7.53 (m, 2H), 7.42 (d, *J* = 8.6 Hz, 1H), 7.37 – 7.31 (m, 2H), 7.31 – 7.25 (m, 2H), 4.78 (s, 1H), 2.72 – 2.40 (m, 8H), 2.29 (s, 3H).

^{13}C NMR (75 MHz, Chloroform-d, ppm) δ 166.7, 149.4, 142.0, 136.4, 129.9, 128.8, 128.8, 128.5, 125.4, 120.2, 111.6, 69.5, 54.9, 51.3, 45.8.

HRMS: Calc. $C_{19}H_{20}ClN_3O^+$ [M+H$^+$] 342.1368; found 342.1360.

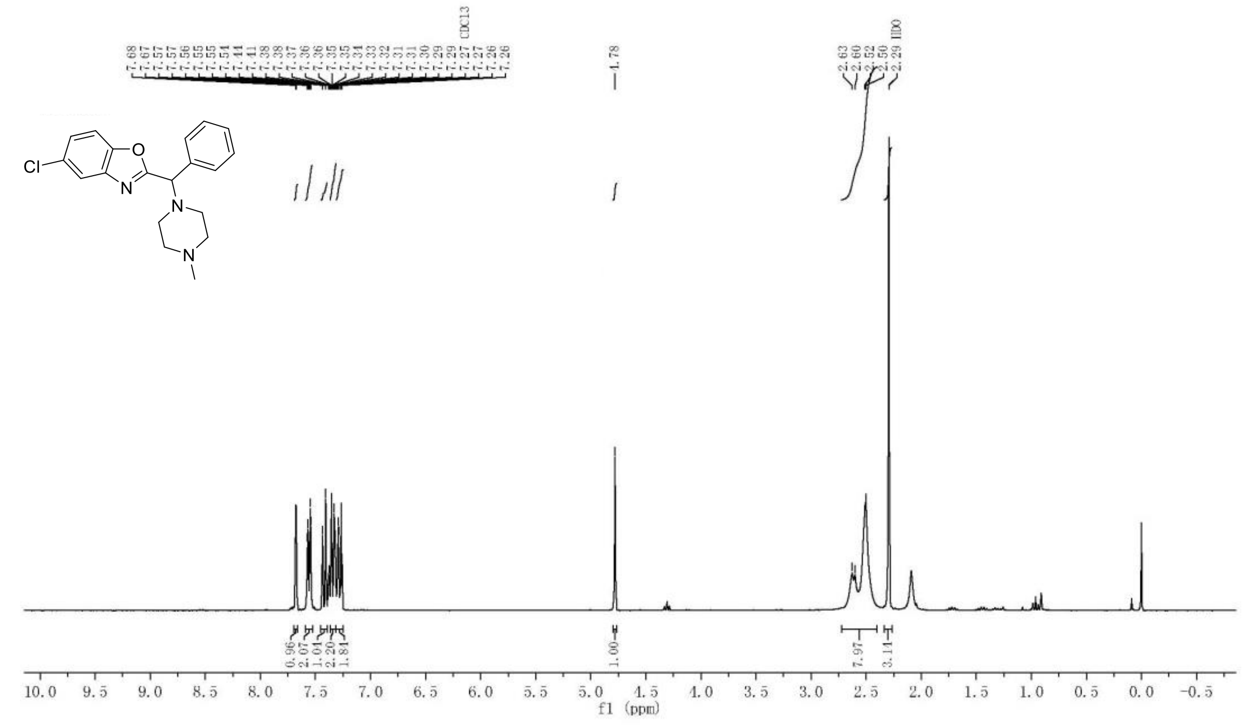

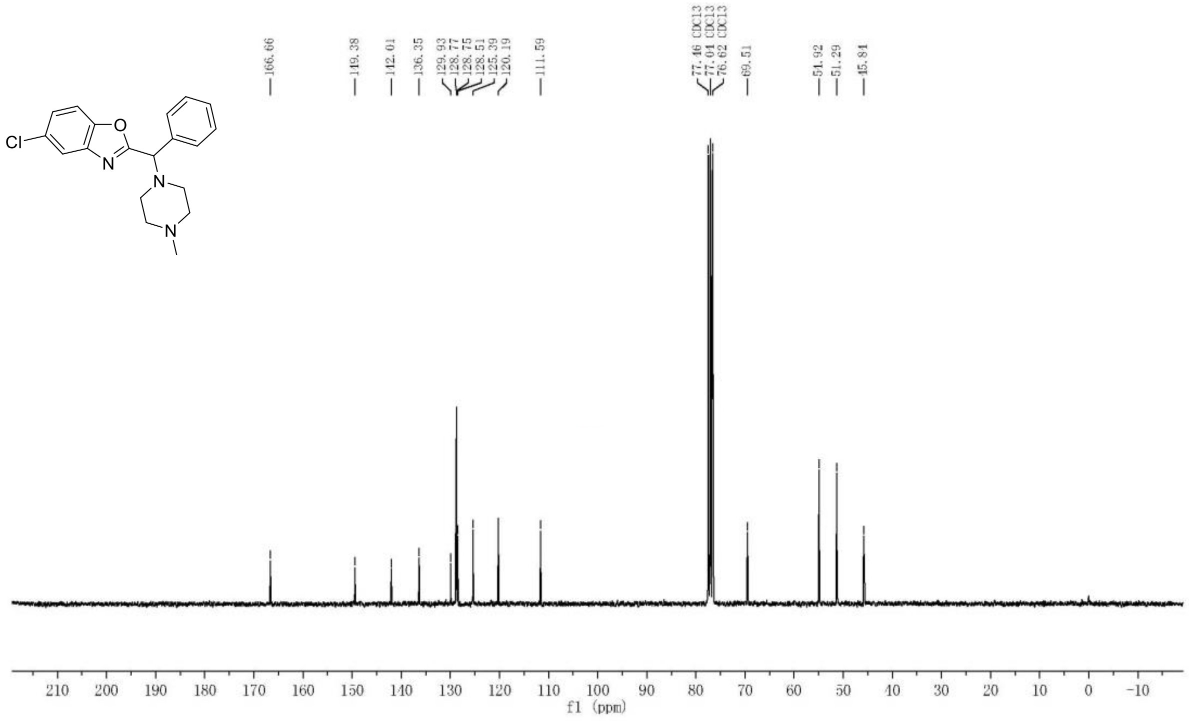

166.66
149.38
142.01
136.35
129.93
128.77
128.75
128.51
125.39
120.19
111.59
77.46 CDCl3
77.04 CDCl3
76.62 CDCl3
69.51
54.92
51.29
45.84
Cl
O
N
N
N
f1 (ppm)

2-(Azepan-1-yl(phenyl)methyl)-5-chlorobenzo[d]oxazole

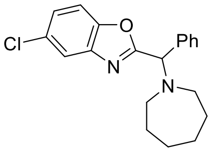

Chemical Formula: $C_{20}H_{21}ClN_2O$
Exact Mass: 340.1342
Molecular Weight: 340.8510

The title compound was prepared according to General Procedure 1 (reaction time: 20 h).

Yield: 147 mg (26%); 48 LCAP before workup/Prep-HPLC

^{1}H NMR (300 MHz, Methanol-d_4, ppm) δ 7.85 (d, J = 1.9 Hz, 1H), 7.71 – 7.66 (m, 2H), 7.60 (d, J = 8.8 Hz, 1H), 7.53 (dd, J = 5.3, 1.6 Hz, 3H), 7.44 (dd, J = 8.8, 2.1 Hz, 1H), 6.19 (s, 1H), 3.54 – 3.42 (m, 4H), 1.96 (s, 4H), 1.73 (s, 4H).

^{13}C NMR (126 MHz, Methanol-d_4, ppm) δ 161.1, 149.4, 141.2, 130.9, 130.5, 129.9, 129.5, 129.3, 126.6, 120.0, 111.9, 66.7, 54.2, 26.1, 23.1.

HRMS: Calc. $C_{20}H_{21}ClN_2O^+$ [M+H$^+$] 341.1415; found 341.1404.

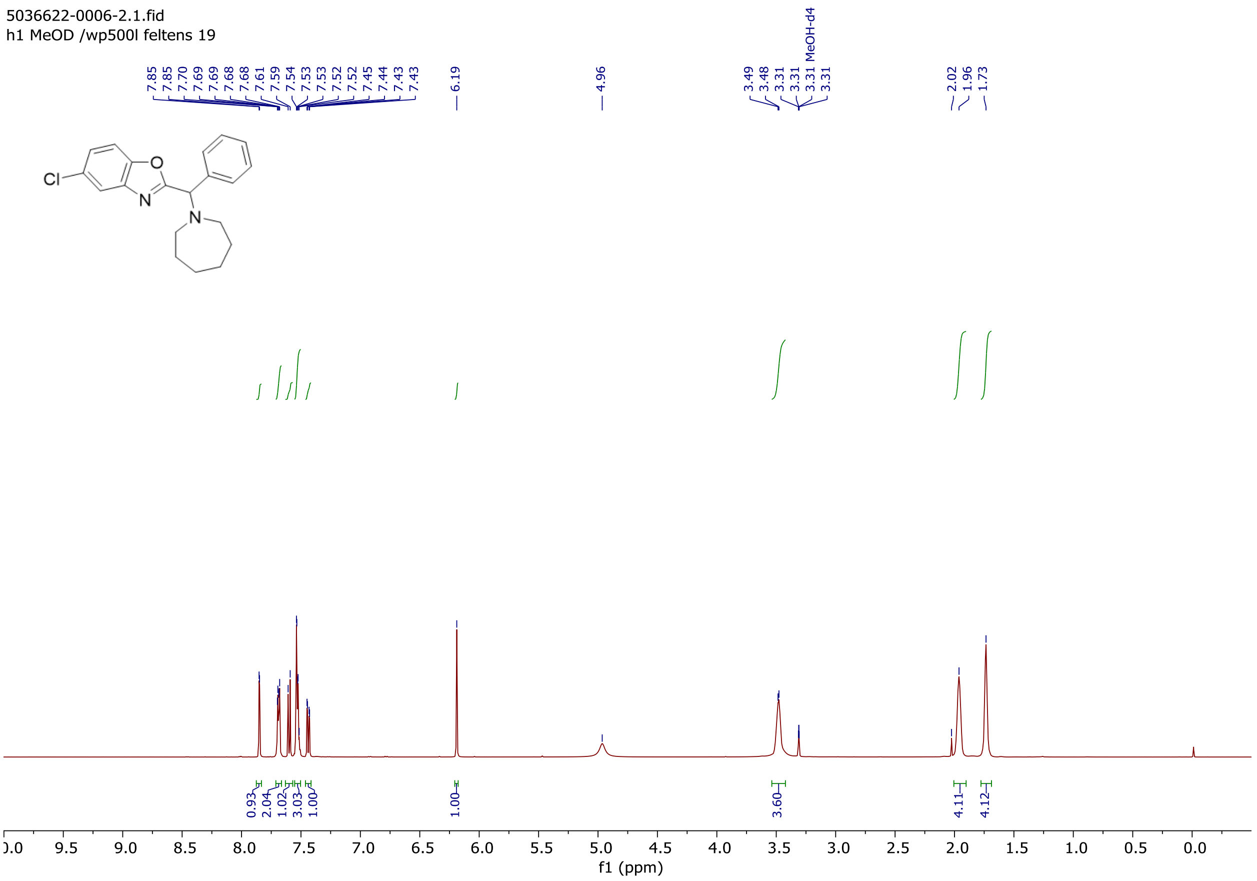

5036622-0006-2-13C.1.fid
c13-night-8hr MeOD /wp500l feltens 52

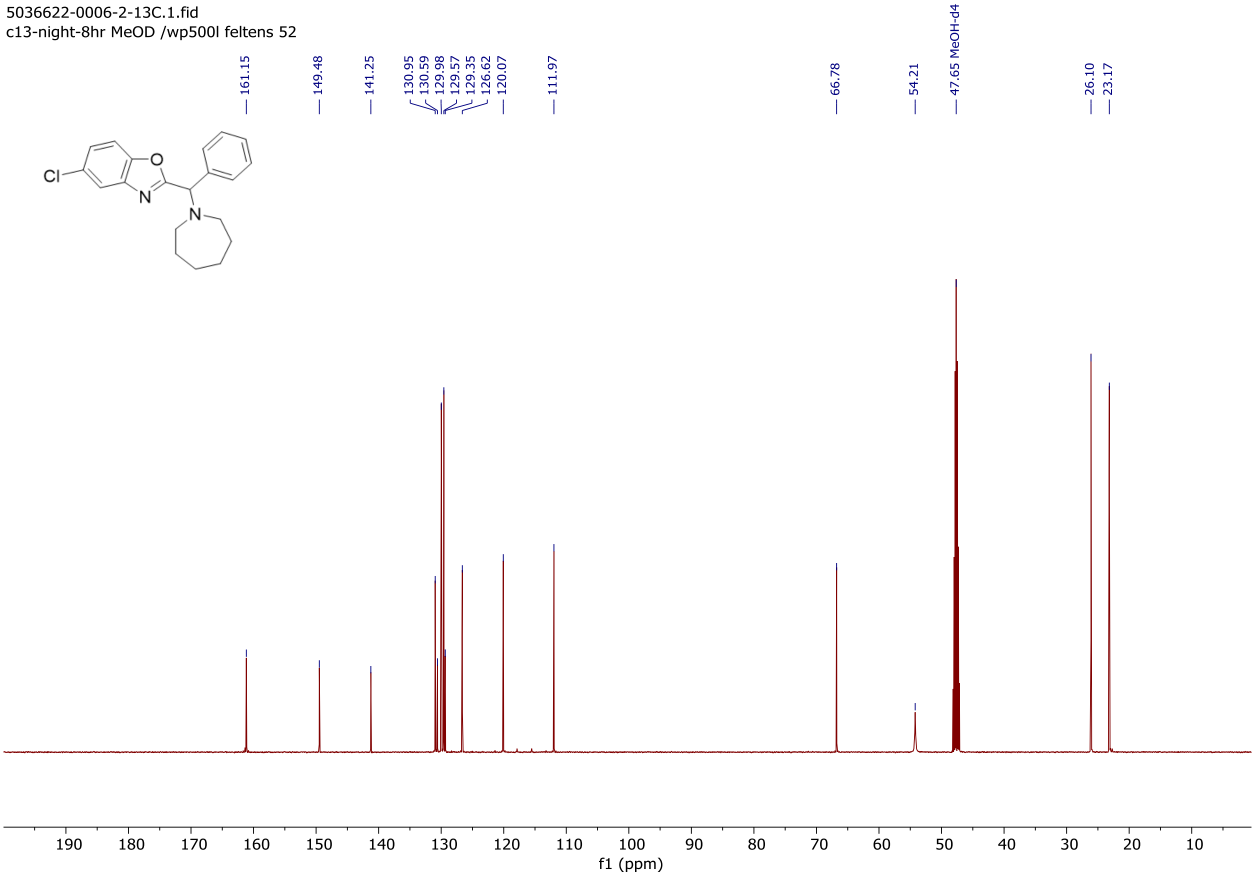

2-(Azetidin-1-yl(phenyl)methyl)-5-chlorobenzo[d]oxazole

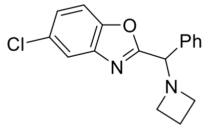

Chemical Formula: $C_{17}H_{15}ClN_2O$
Exact Mass: 298.0873
Molecular Weight: 298.7700

2-(zetidin-1-yl(phenyl)methyl)-5-chlorobenzo[d]oxazole

The title compound was prepared according to General Procedure 1 (reaction time: 20 h).

Yield: 71 mg (8%); 22 LCAP before workup/column chromatography

^{1}H NMR (300 MHz, Chloroform-d, ppm) δ 7.67 (d, *J* = 2.1 Hz, 1H), 7.58 – 7.51 (m, 2H), 7.42 – 7.29 (m, 4H), 7.29 – 7.23 (m, 1H), 4.73 (s, 1H), 3.31 (m, 4H), 2.16 (m, 2H).

^{13}C NMR (75 MHz, Chloroform-d, ppm) δ 166.6, 149.5, 142.1, 136.2, 129.8, 128.8, 128.6, 128.4, 125.3, 120.2, 111.5, 71.3, 54.3, 17.3.

HRMS: Calc. $C_{17}H_{15}ClN_2O^+$ [M+H$^+$] 299.0946; found 299.0940.

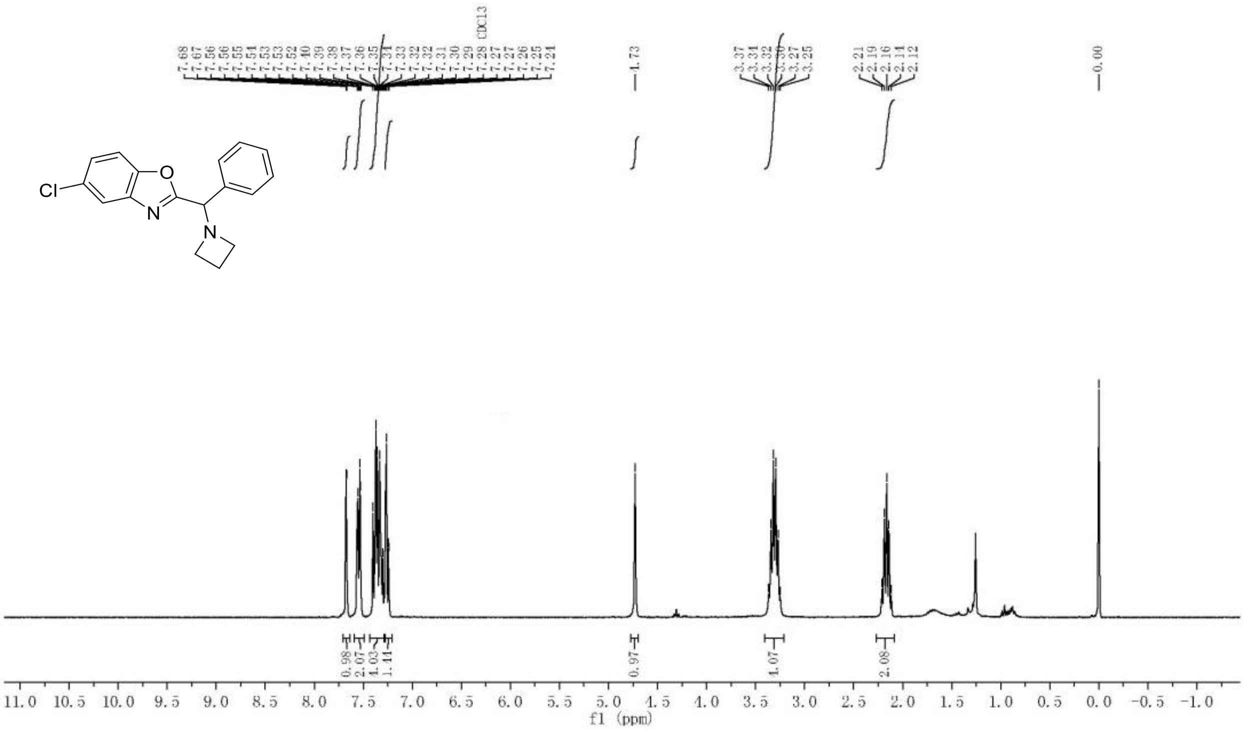

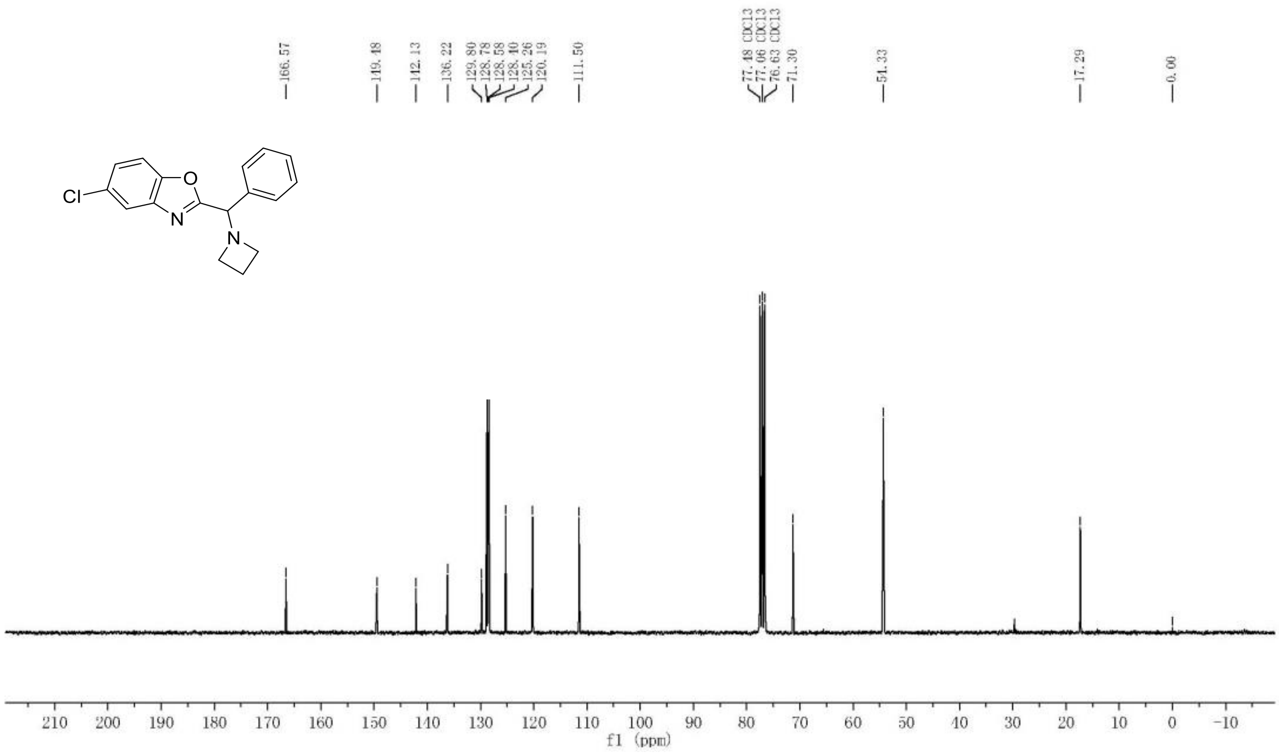

166.57
149.48
142.13
136.22
129.80
128.78
128.58
128.40
125.26
120.19
111.50
77.48 CDCl3
77.06 CDCl3
76.63 CDCl3
71.30
54.33
17.29
0.00
Cl
O
N
N
210 200 190 180 170 160 150 140 130 120 110 100 90 80 70 60 50 40 30 20 10 0 -10
f1 (ppm)

N-((5-Chlorobenzo[d]oxazol-2-yl)(phenyl)methyl)cyclohexanamine

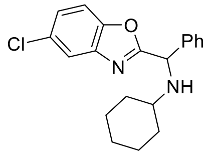

Chemical Formula: $C_{20}H_{21}ClN_2O$
Exact Mass: 340.1342
Molecular Weight: 340.8510

The title compound was prepared according to General Procedure 1 (reaction time: about 20 h).

Yield: 53 mg (7%); 12 LCAP before workup/column chromatography

^{1}H NMR (300 MHz, Methanol-d_4, ppm) δ 7.85 (d, *J* = 2.0 Hz, 1H), 7.67 – 7.61 (m, 2H), 7.59 (d, *J* = 8.8 Hz, 1H), 7.55 – 7.50 (m, 3H), 7.44 (dd, *J* = 8.8, 2.1 Hz, 1H), 6.16 (s, 1H), 4.92 (br. s, 1H), 3.21 – 3.13 (m, 1H), 2.31 (d, *J* = 12.2 Hz, 1H), 2.24 (d, *J* = 12.0 Hz, 1H), 1.87 (t, *J* = 11.1 Hz, 2H), 1.68 (d, *J* = 10.5 Hz, 1H), 1.49 (dt, *J* = 12.1, 3.9 Hz, 2H), 1.30 – 1.20 (m, 3H).

^{13}C NMR (126 MHz, Methanol-d_4, ppm) δ 163.4, 151.1, 142.5, 132.3, 131.8, 131.8, 130.9, 130.0, 127.7, 121.3, 113.2, 58.0, 57.3, 30.2, 30.2, 25.9, 25.5, 25.5.

HRMS: Calc. $C_{20}H_{21}ClN_2O^+$ [M+H$^+$] 341.1415; found 341.1408.

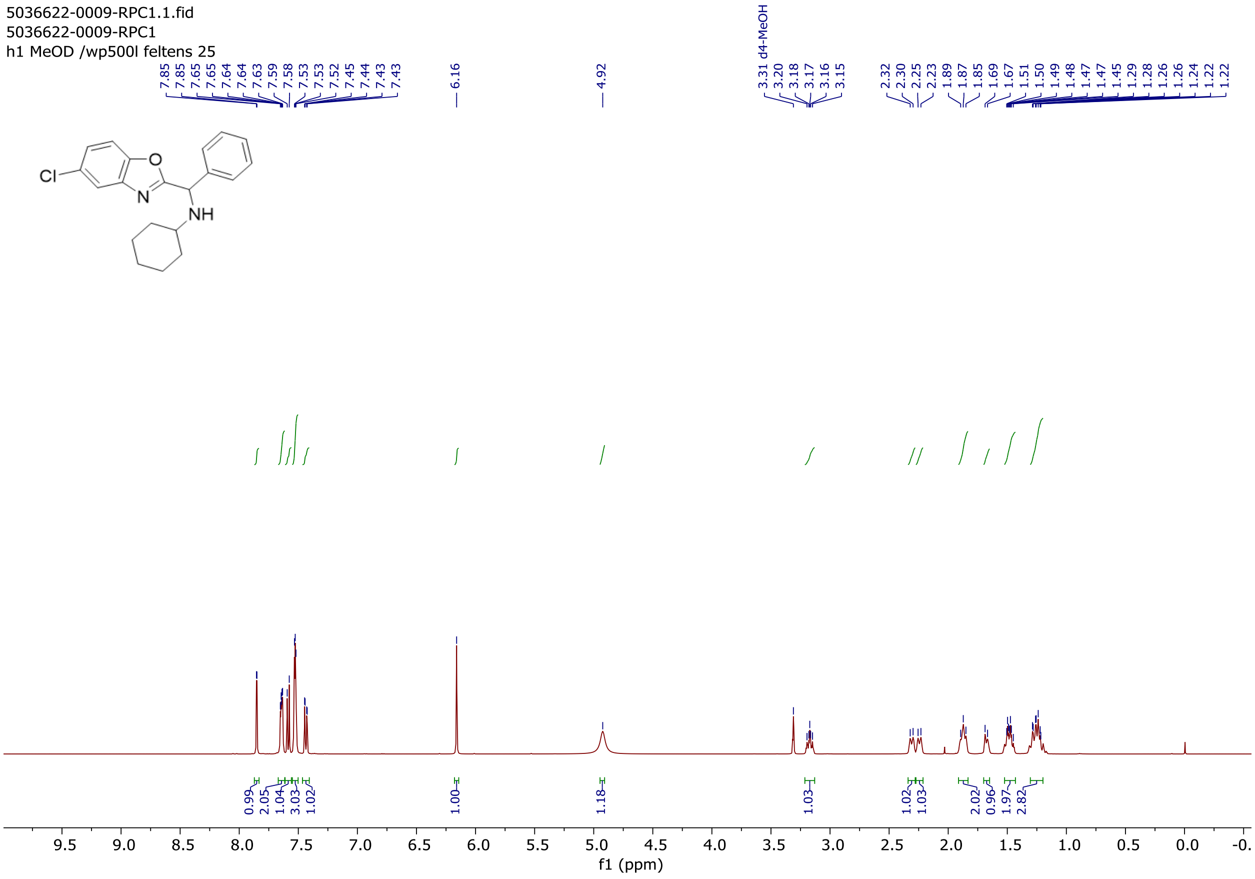

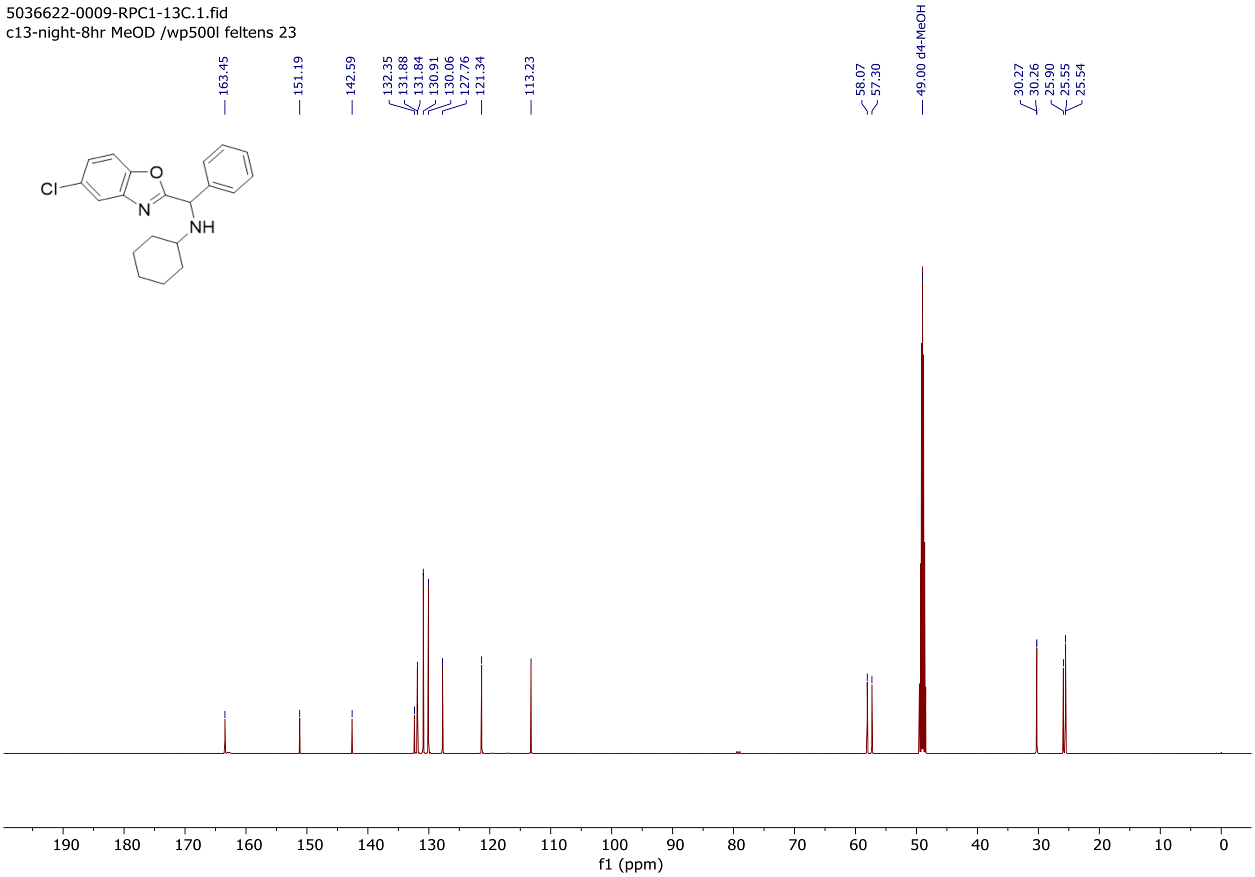
5036622-0009-RPC1-13C.1.fid
c13-night-8hr MeOD /wp500l feltens 23
163.45
151.19
142.59
132.35
131.88
131.84
130.91
130.06
127.76
121.34
113.23
58.07
57.30
49.00 d4-MeOH
30.27
30.26
25.90
25.55
25.54
Cl
O
N
NH
190 180 170 160 150 140 130 120 110 100 90 80 70 60 50 40 30 20 10 0
f1 (ppm)

N-((5-Chlorobenzo[d]oxazol-2-yl)(phenyl)methyl)-N-phenylaniline

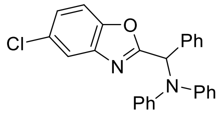

Chemical Formula: $C_{26}H_{19}ClN_2O$
Exact Mass: 410.1186
Molecular Weight: 410.9010

The title compound was prepared according to General Procedure 1 (reaction time: about 20 h).

Yield: 30 mg (6%); 19 LCAP before workup/column chromatography

^{1}H NMR (300 MHz, Chloroform-d, ppm) δ 7.69 (d, *J* = 2.0 Hz, 1H), 7.37 – 7.23 (m, 7H), 7.22 – 7.11 (m, 4H), 7.05 – 6.89 (m, 6H), 6.61 (s, 1H).

^{13}C NMR (75 MHz, Chloroform-d, ppm) δ 166.6, 149.4, 146.4, 142.1, 135.9, 129.9, 129.1, 128.5, 128.2, 125.4, 123.0, 122.6, 120.4, 111.4, 63.2.

HRMS: Calc. $C_{26}H_{19}ClN_2O^+$ [M+H$^+$] 411.1259; found 411.1255.

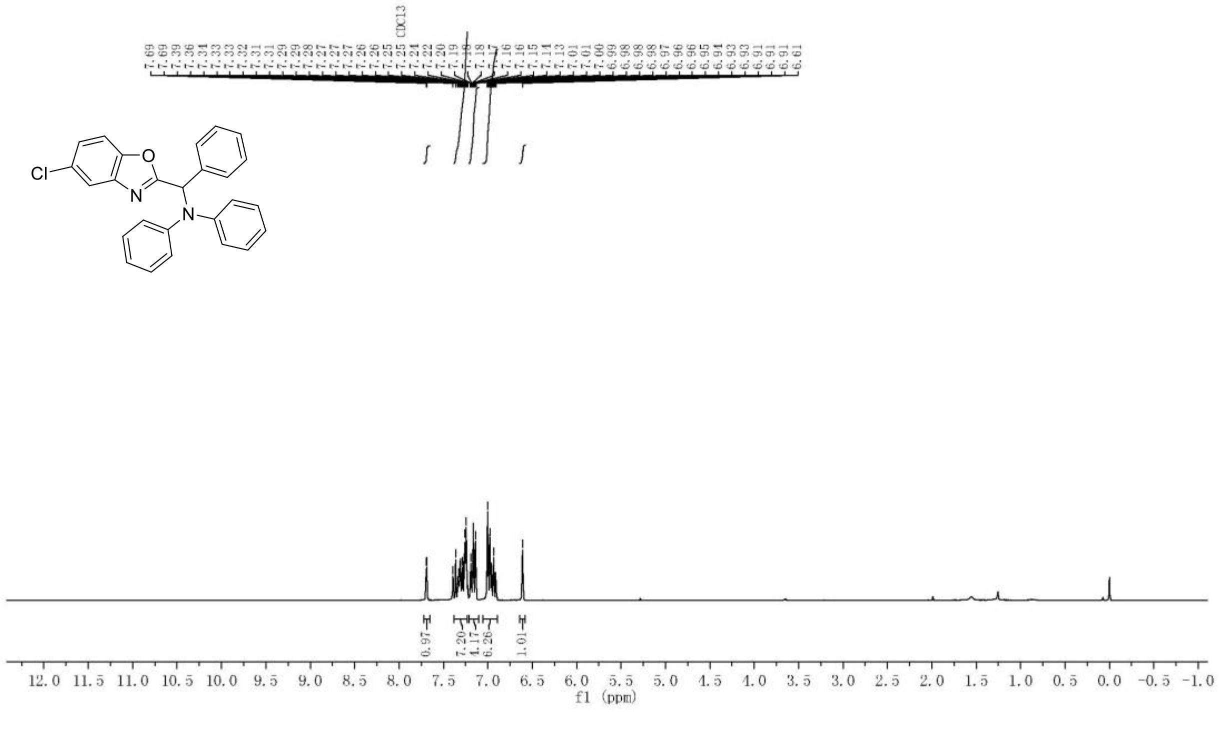

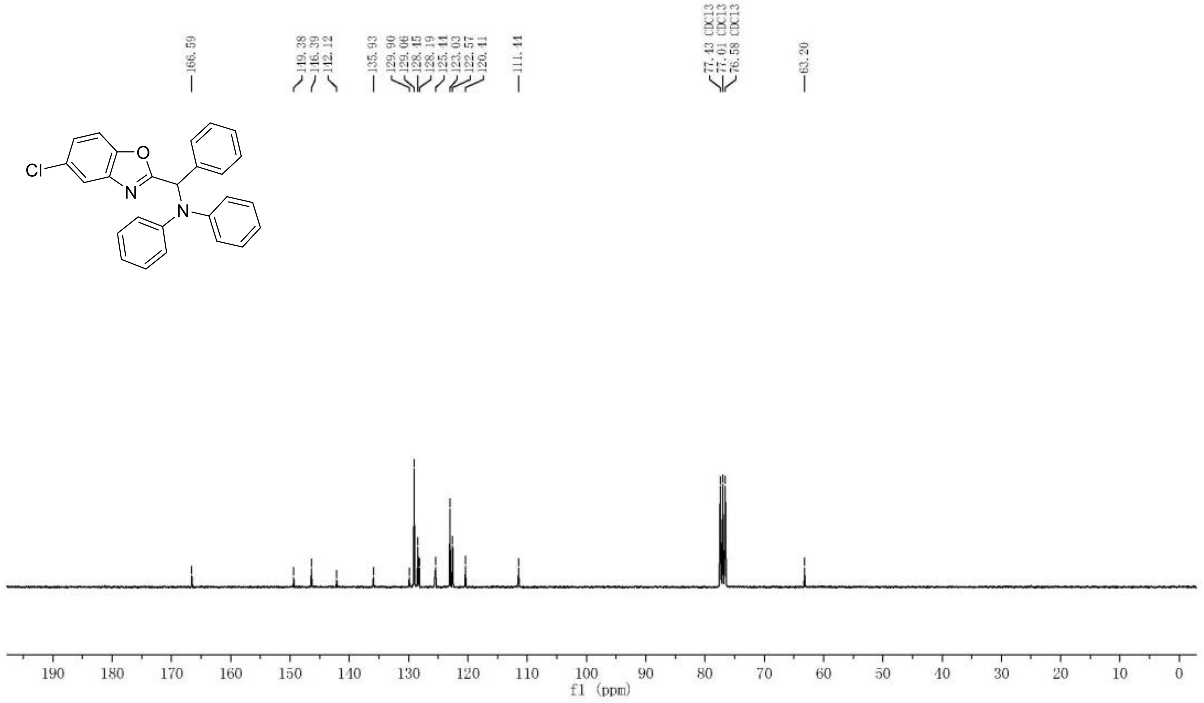
166.59
149.38
146.39
142.12
135.93
129.90
129.06
128.45
128.19
125.41
123.03
122.57
120.41
111.44
77.43 CDCl3
77.01 CDCl3
76.58 CDCl3
63.20
Cl
O
N
N
f1 (ppm)

Amine with low reactivity (<2 LCAP) under conditions of General Procedure 1

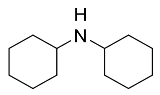

Modified Conditions for Amine-HCl Salts as Substrates

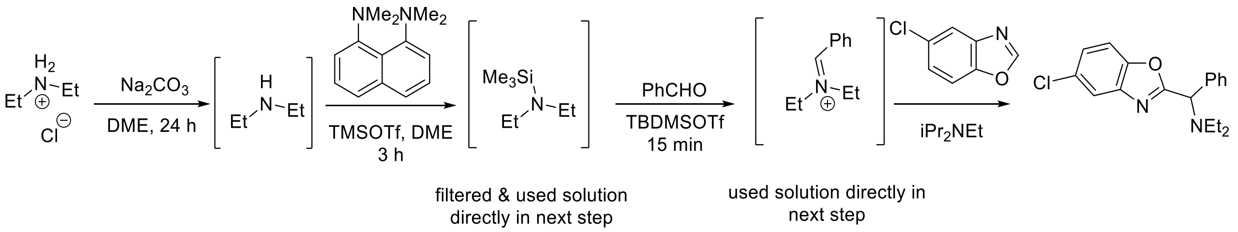

Na_2CO_3 (223 mg, 2.100 mmol) was weighed into a vial containing a stirbar and diethylamine HCl adduct (164 mg, 1.500 mmol). The vial was introduced into the glovebox, where 1,2-dimethoxyethane (1.0 mL) was added. The mixture was stirred vigorously at room temperature in the glovebox for 18 h.

The suspension was filtered through a syringe filter into a vial containing *N*1,*N*1,*N*8,*N*8-tetramethylnaphthalene-1,8-diamine (300 mg, 1.400 mmol; Proton-Sponge®). The filter cake was rinsed with 1,2-dimethoxyethane (0.8 mL). Trimethylsilyl trifluoromethanesulfonate (0.271 mL, 1.500 mmol) was added to the combined filtrates and the resulting suspension was stirred for 3 h in the glovebox. Then, the mixture was filtered through a kimwipe-clogged pipette tip and 1,2-dimethoxyethane (0.50 mL) was used to rinse the reaction vial and the filtercake. To the combined filtrates, benzaldehyde (0.102 mL, 1.0 mmol) and *tert*-butyldimethylsilyl trifluoromethanesulfonate (0.919 mL, 4.00 mmol) were added and the mixture was stirred for 15 min at room temperature in the glovebox.

N-Ethyl-*N*-isopropylpropan-2-amine (0.523 mL, 3.00 mmol) was added to another vial containing 5-chlorobenzo[d]oxazole (154 mg, 1.00 mmol). The contents of both vials were combined, and the reaction mixture was removed from the glovebox and heated to 50 °C for 18 h.

To quench any remaining silyl triflate reagent, the mixture was poured into a mixture of potassium fluoride (465 mg, 8.00 mmol) and pyridine (0.5 mL, 6.21 mmol). The resulting suspension was evaporated to dryness (rotavap). The residue was suspended in 2 mL DCM and 2 mL water and the DCM phase was purified by silica gel chromatography to afford 187 mg (59%) of *N*-((5-chlorobenzo[d]oxazol-2-yl)(phenyl)methyl)-*N*-ethylethanamine as a colorless oil.

^{1}H NMR (500 MHz, Methanol-d_4, ppm) δ 7.69 (s, 1H), 7.56 (d, J = 8.6 Hz, 1H), 7.50 (d, J = 7.4 Hz, 2H), 7.42 – 7.31 (m, 3H), 7.31 – 7.25 (m, 1H), 5.26 (s, 1H), 2.69 (dq, J = 13.5, 6.9 Hz, 2H), 2.58 (dq, J = 13.5, 6.6 Hz, 2H), 1.03 (t, J = 6.9 Hz, 6H).

For full characterization, see page 60.

Amine Scope II Via Parallel Medicinal Chemistry

General Procedure 3

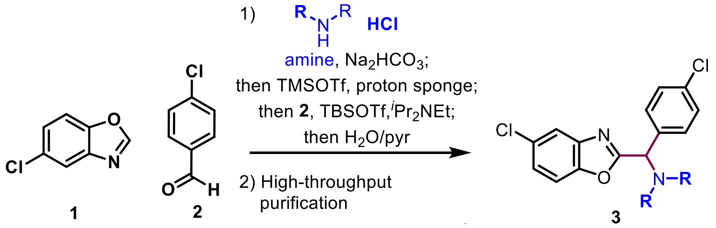

Na_2CO_3 (39.6 mg, 0.373 mmol) was weighed into a vial containing amine hydrochloride (0.267 mmol), which was then charged with a stirbar and introduced into the glovebox. Then, 1,2-dimethoxyethane (0.8 mL) was added and the mixture was stirred vigorously at room temperature in the glovebox for 20 h. The mixture was filtered through a syringe filter into a vial containing *N*1,*N*1,*N*8,*N*8-tetramethylnaphthalene-1,8-diamine (53.4 mg, 0.249 mmol). The filter cake was rinsed with 1,2-dimethoxyethane (0.4 mL). Trimethylsilyl trifluoromethanesulfonate (0.048 mL, 0.267 mmol) was added to the combined filtrates and the resulting suspension was stirred for 4 h. The mixture was filtered through a kimwipe-clogged pipette tip directly into a vial containing 4-chlorobenzaldehyde (25 mg, 0.178 mmol); 1,2-dimethoxyethane (0.50 mL) was used to rinse the reaction vial and the filtercake. *tert*-Butyldimethylsilyl trifluoromethanesulfonate (0.163 mL, 0.711 mmol) was added to the combined filtrates and the mixture was stirred for 30 min. *N*-Ethyl-*N*-isopropylpropan-2-amine (0.093 mL, 0.534 mmol) was added into another vial containing 5-chlorobenzo[d]oxazole (27.3 mg, 0.178 mmol); the mixture in both vials were combined and the resulting reaction mixture was removed from the glovebox and heated to 50 °C for 18 h. The mixture was sampled by UPLC/MS. The crude reaction mixture was evaporated and was purified by reverse-phase preparative HPLC. All compounds were isolated a TFA salts.

5-Chloro-2-((4-chlorophenyl)(3-(trifluoromethyl)piperidin-1-yl)methyl)benzo[d]oxazole

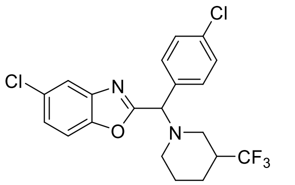

Chemical Formula: $C_{20}H_{17}Cl_2F_3N_2O$
Exact Mass: 428.0670
Molecular Weight: 429.2642

The title compound was prepared according to General Procedure 3 (PMC). Isolated as $C_{20}H_{17}Cl_2F_3N_2O(F_3CCO_2H)_{1.5}$ (M_w 429.2642 + 114.0232*1.5 = 600.299).

Yield: 26.3 mg (25%); 36 LCAP before workup/column chromatography; 1:1 mix of 2 diastereomers.

^{1}H NMR (500 MHz, Methanol-d_4, ppm) δ 7.79 (dd, *J* = 8.5, 1.9 Hz, 1H), 7.63 – 7.51 (m, 3H), 7.48 – 7.41 (m, 3H), 5.49 (s, 0.5H; diastereomer 1), 5.43 (s, 0.5H – diastereomer 2), 3.33 (d, *J* = 7.9 Hz, 0.5H – diastereomer 1), 3.27 (d, *J* = 11.2 Hz, 0.5H – diastereomer 2), 3.15 (t, *J* = 11.0 Hz, 1H), 2.69 – 2.50 (m, 2H), 2.48 – 2.40 (m, 1H), 2.01 (d, *J* = 16.2 Hz, 1H), 1.88 (d, *J* = 13.6 Hz, 1H), 1.77 (q, *J* = 12.7 Hz, 1H), 1.42 (qd, *J* = 12.7, 4.6 Hz, 1H).

^{19}F NMR (470 MHz, Methanol-d_4, ppm) δ -73.91 (ds 1), -74.00 (ds 2), -77.58 (TFA).

^{13}C NMR (126 MHz, Methanol-d_4, ppm) δ 165.9 (ds1), 165.7 (ds 2), 160.7 (q, *J* = 39.0 Hz; TFA), 150.9, 142.9 (ds1), 142.9 (ds2), 136.8 (ds1), 136.8 (ds2), 133.9 (ds1), 133.7 (ds2), 132.1 (ds1), 132.0 (ds2), 131.9 (ds1), 131.8 (ds2), 130.5, 127.9 (q, *J* = 289 Hz; ds1), 127.9 (q, *J* = 289 Hz; ds1), 127.7 (ds1), 127.6 (ds2), 121.2 (ds1), 121.2 (ds2), 117.1 (q, *J* = 290 Hz; TFA), 113.6 (ds1), 113.3 (ds2), 68.7 (ds1), 68.5 (ds2), 52.6 (ds1), 52.6 (ds2), 51.1 (q, *J* = 3.4 Hz; ds1), 50.9 (q, *J* = 3.1 Hz; ds2), 41.7 (q, *J* = 26.7 Hz; ds1), 41.6 (q, *J* = 26.9 Hz; ds2), 24.4 (ds1), 24.2 (ds2), 23.8 (m).

HRMS: Calc. $C_{20}H_{18}Cl_2F_3N_2O^+$ [M+H$^+$] 429.0743; found 429.0747.

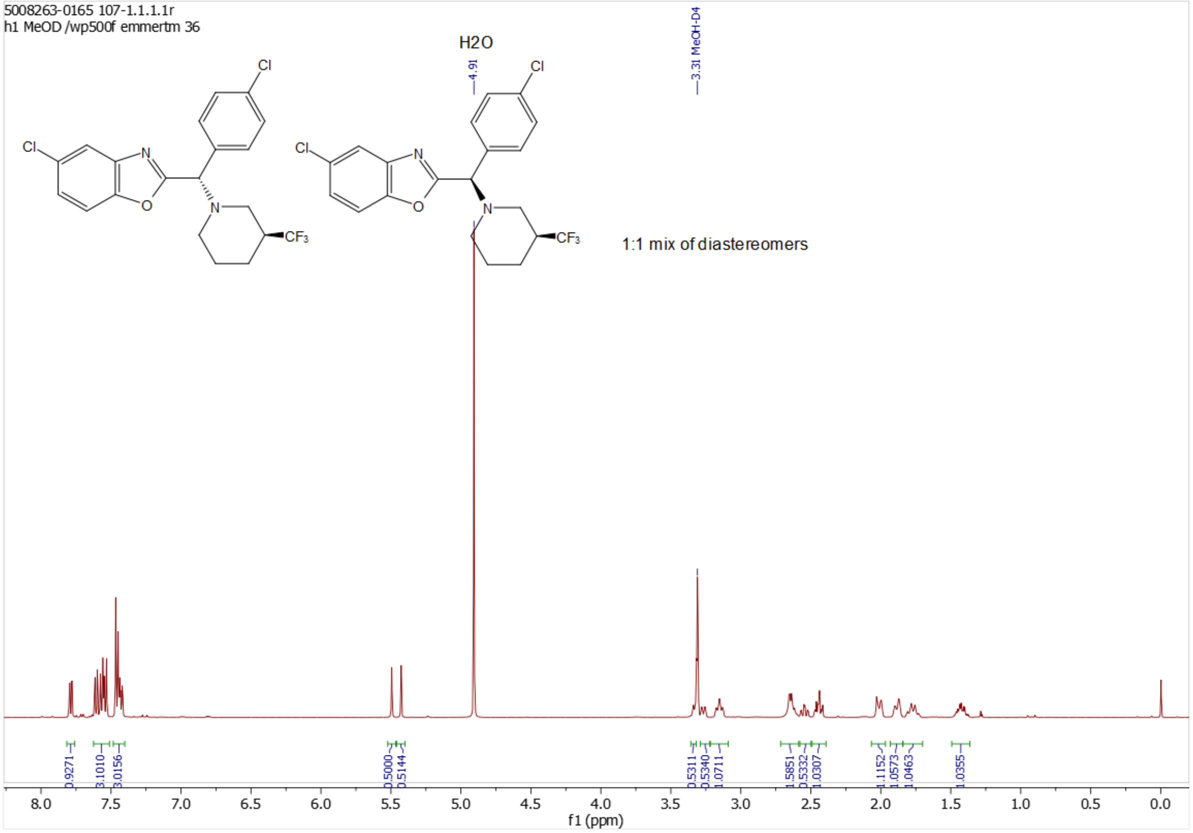
5008263-0165 107-1.1.1.1r
h1 MeOD /wp500f emmertm 36
H2O
4.91
3.31 MeOH-D4
1:1 mix of diastereomers
f1 (ppm)

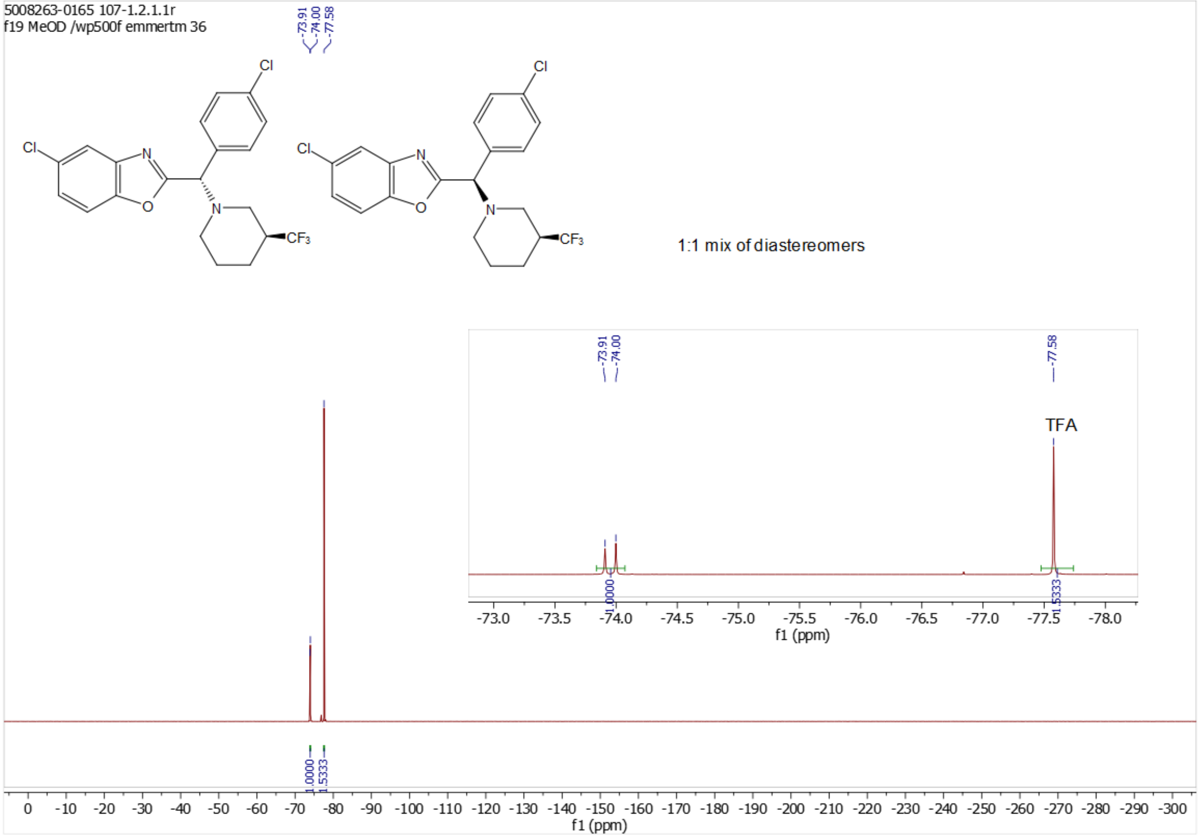
5008263-0165 107-1.2.1.1r
f19 MeOD /wp500f emmertm 36
-73.91
-74.00
-77.58
1:1 mix of diastereomers
TFA
f1 (ppm)

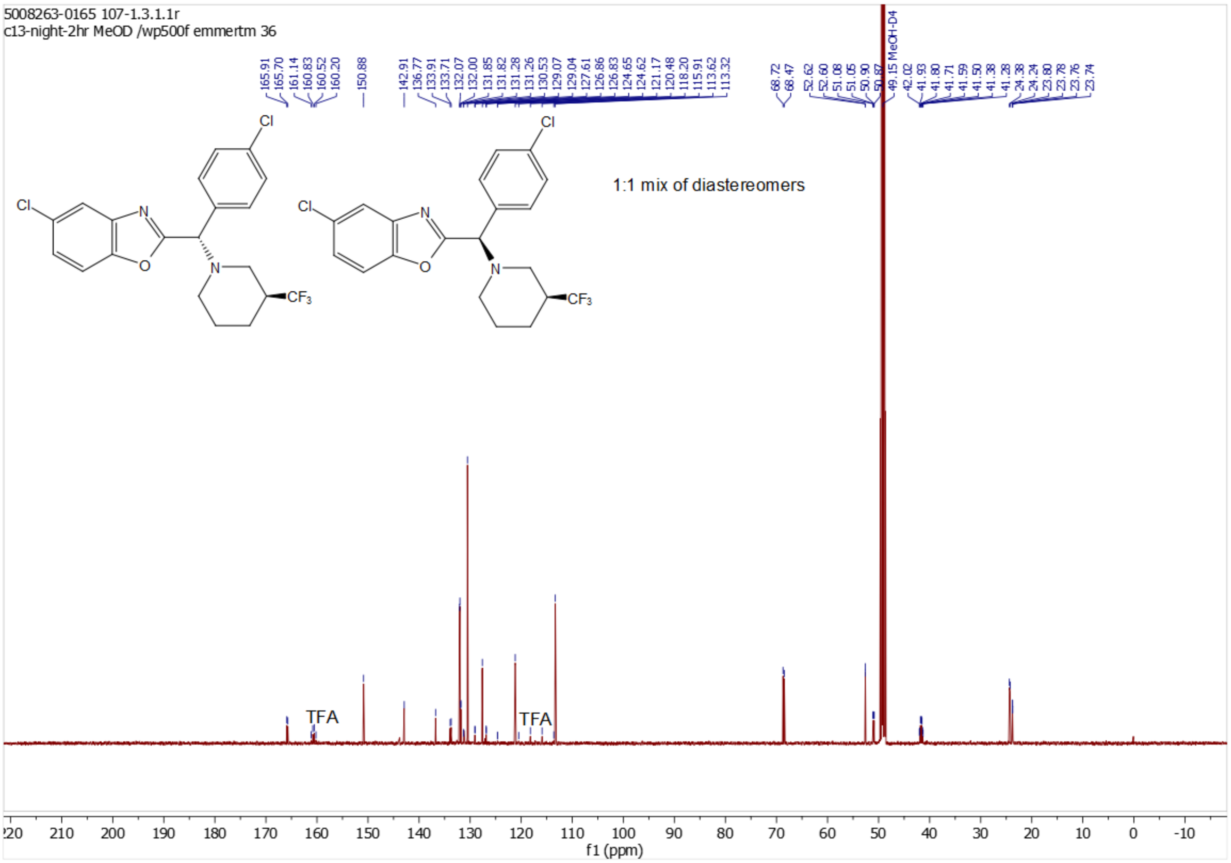

5008263-0165 107-1.3.1.1r
c13-night-2hr MeOD /wp500f emmertm 36
165.91 165.70 161.14 160.83 160.52 160.20 150.88 142.91 136.77 133.91 133.71 132.07 132.00 131.85 131.82 131.28 131.26 130.53 129.07 129.04 127.61 126.86 126.83 124.65 124.62 121.17 120.48 118.20 115.91 113.62 113.32
68.72 68.47 52.62 52.60 51.08 51.05 50.90 50.87 49.15 MeOH-D4 42.02 41.93 41.80 41.71 41.59 41.50 41.38 41.28 24.38 24.24 23.80 23.78 23.76 23.74
Cl
Cl
N
O
N
CF3
1:1 mix of diastereomers
TFA
TFA
f1 (ppm)

5-Chloro-2-((4-chlorophenyl)(3,3-dimethyl-4-(trifluoromethyl)piperidin-1-yl)methyl)benzo[d]oxazole

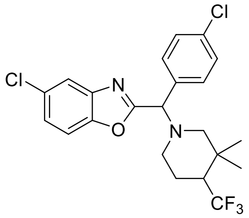

Chemical Formula: $C_{22}H_{21}Cl_2F_3N_2O$
Exact Mass: 456.0983
Molecular Weight: 457.3182

The title compound was prepared according to General Procedure 3 (PMC). Isolated as $C_{22}H_{21}Cl_2F_3N_2O(F_3CCO_2H)$ (M_w 457.3182 + 114.0232 = 571.3414 g/mol).

Yield: 44 mg (43%); 39 LCAP before workup/column chromatography

^{1}H NMR (500 MHz, Methanol-d_4, ppm) δ 7.75 (d, J = 1.9 Hz, 1H), 7.61 (d, J = 8.7 Hz, 1H), 7.56 (d, J = 8.5 Hz, 2H), 7.45 – 7.41 (m, 3H), 5.18 (s, 1H), 3.05 (d, J = 11.1 Hz, 1H), 2.73 (d, J = 11.4 Hz, 1H), 2.40 (t, J = 12.6 Hz, 1H), 2.10 (m, 2H), 1.95 (qd, J = 12.8, 12.3, 4.2 Hz, 1H), 1.88 – 1.81 (m, 1H), 1.22 (s, 3H), 1.04 (s, 3H).

^{19}F NMR (470 MHz, Methanol-d_4, ppm) δ -66.25, -77.57 (TFA).

^{13}C NMR (126 MHz, Methanol-d_4, ppm) δ 166.6, 160.5 (q, J = 39.3 Hz), 150.7, 142.7, 136.1, 130.1, 129.4 (d, J = 281.0 Hz), 127.2, 120.8, 116.9 (q, J = 289.4 Hz), 113.1, 68.6, 65.8, 51.8, 34.1, 28.2, 23.2, 21.0.

HRMS: Calc. $C_{22}H_{22}Cl_2F_3N_2O^+$ [M+H$^+$] 457.1056; found 457.1053.

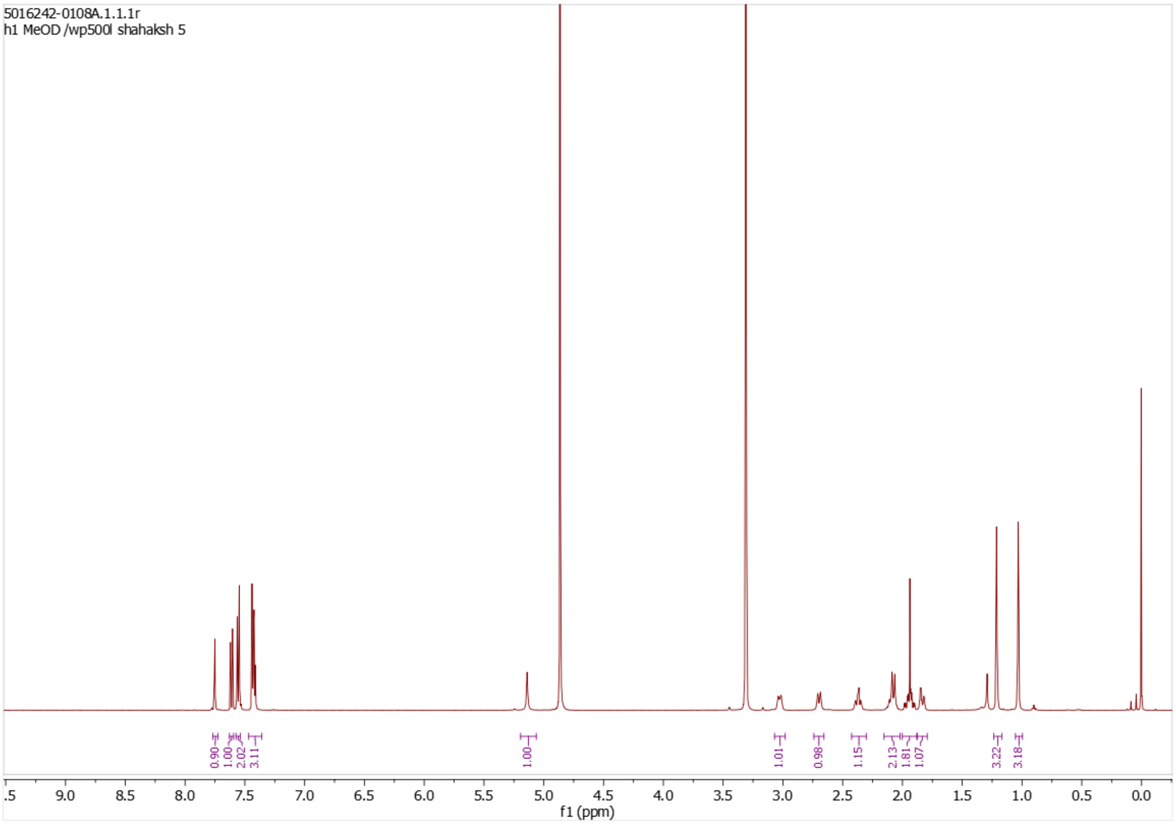
5016242-0108A.1.1.1r
h1 MeOD /wp500l shahaksh 5
0.90
1.00
2.02
3.11
1.00
1.01
0.98
1.15
2.13
1.81
1.07
3.22
3.18
.5 9.0 8.5 8.0 7.5 7.0 6.5 6.0 5.5 5.0 4.5 4.0 3.5 3.0 2.5 2.0 1.5 1.0 0.5 0.0
f1 (ppm)

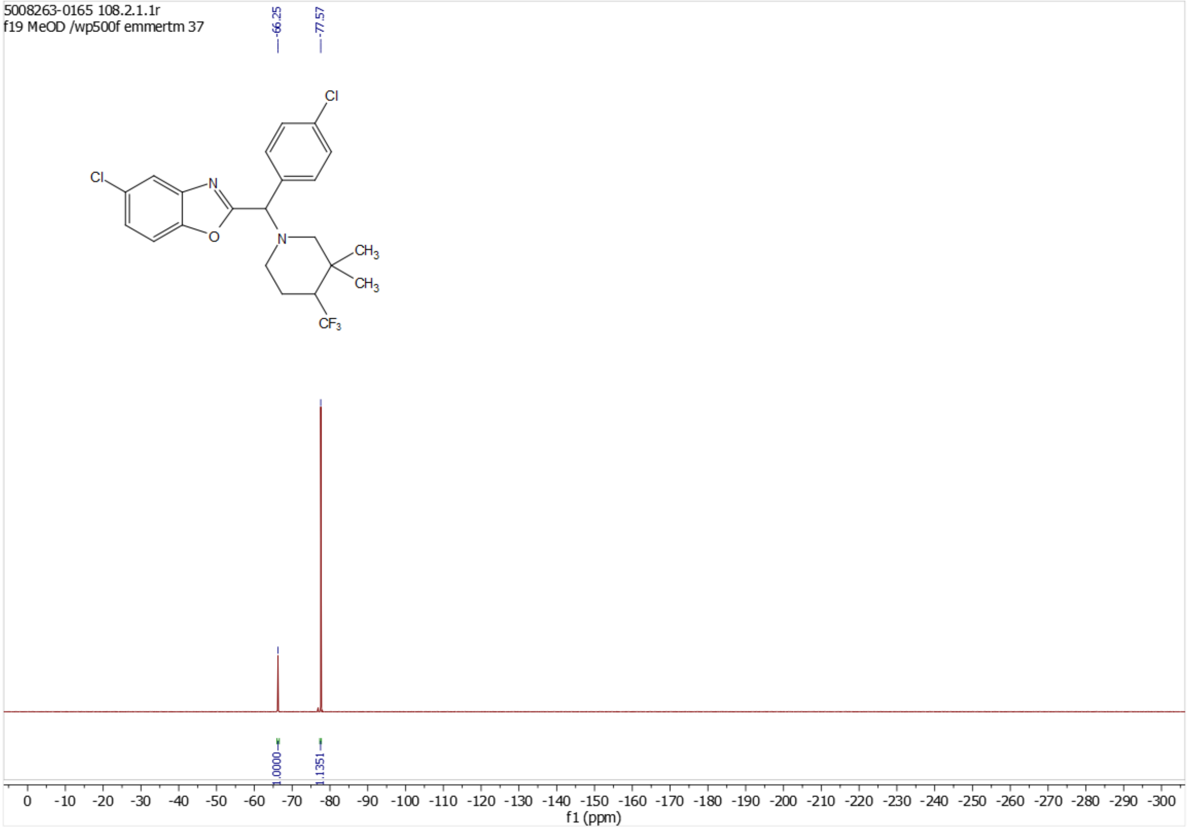
5008263-0165 108.2.1.1r
f19 MeOD /wp500f emmertm 37
-66.25
-77.57
Cl
Cl
N
O
N
CH_3
CH_3
CF_3
1.0000
1.1351
0 -10 -20 -30 -40 -50 -60 -70 -80 -90 -100 -110 -120 -130 -140 -150 -160 -170 -180 -190 -200 -210 -220 -230 -240 -250 -260 -270 -280 -290 -300
f1 (ppm)

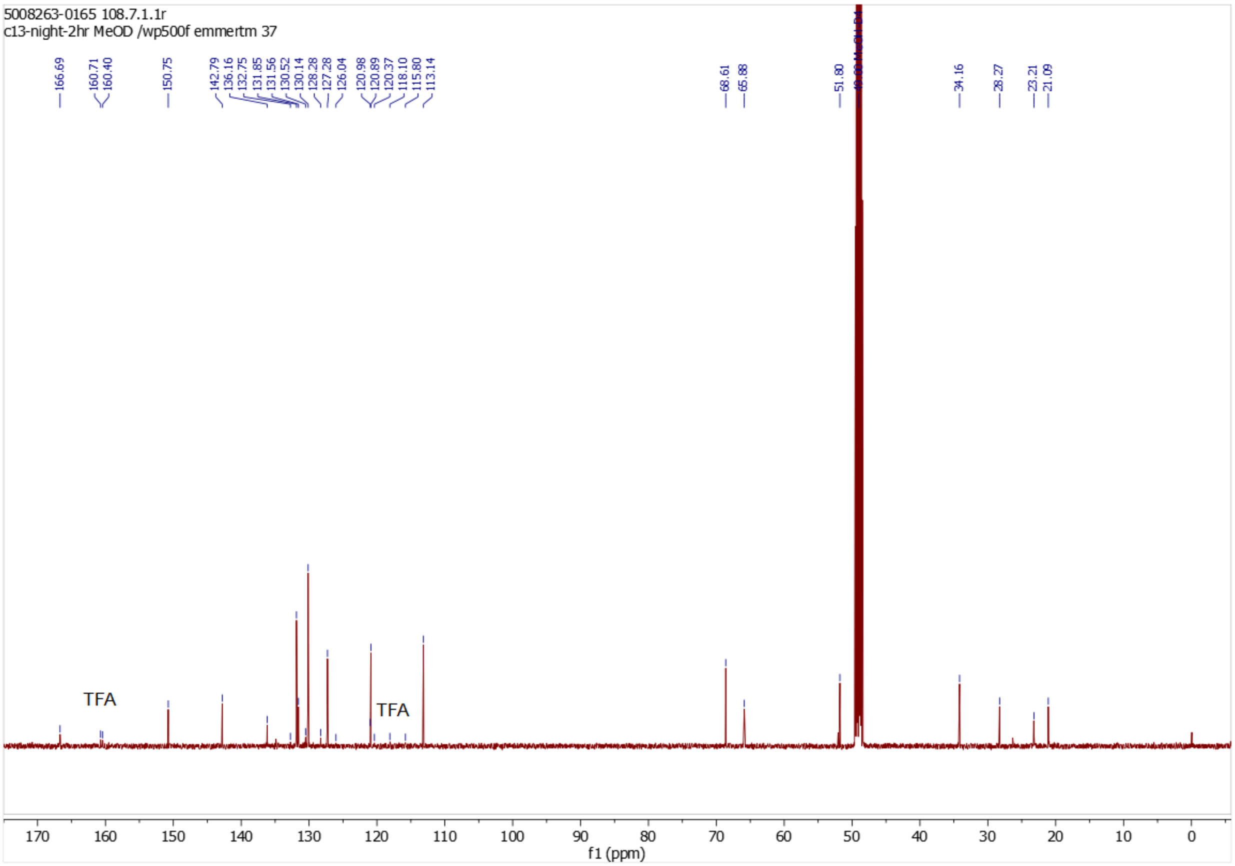
5008263-0165 108.7.1.1r
c13-night-2hr MeOD /wp500f emmertm 37
166.69
160.71
160.40
150.75
142.79
136.16
132.75
131.85
131.56
130.52
130.14
128.28
127.28
126.04
120.98
120.89
120.37
118.10
115.80
113.14
68.61
65.88
51.80
34.16
28.27
23.21
21.09
TFA
TFA
f1 (ppm)

5-Chloro-2-((4-chlorophenyl)(7,7-difluoro-6-methyl-3-azabicyclo[4.1.0]heptan-3-yl)methyl)benzo[d]oxazole

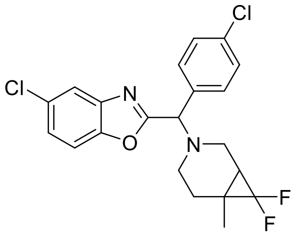

Chemical Formula: $C_{21}H_{18}Cl_2F_2N_2O$
Exact Mass: 422.0764
Molecular Weight: 423.2848

The title compound was prepared according to General Procedure 3 (PMC). Isolated as two separated diastereomers of $C_{21}H_{18}Cl_2F_2N_2O(F_3CCO_2H)_{2.8}$ (apparent M_w 423.2848 + 114.0232*2.8 = 742.5498 g/mol).

Yield: 33 mg (both diastereomers combined; 25%); 46 LCAP before workup/column chromatography

Diastereomer 1:

^{1}H NMR (500 MHz, Methanol-d_4, ppm) δ 7.83 (d, J = 2.1 Hz, 1H), 7.64 – 7.58 (m, 3H), 7.53 – 7.50 (m, 2H), 7.46 (dd, J = 8.8, 2.1 Hz, 1H), 5.65 (s, 1H), 3.39 – 3.33 (m, 1H), 3.28 – 3.15 (m, 2H), 2.98 (dd, J = 13.1, 3.7 Hz, 1H), 2.82 – 2.74 (m, 1H), 2.19 (d, J = 14.0 Hz, 1H), 2.10 (ddq, J = 12.6, 9.2, 5.2, 4.6 Hz, 1H), 1.64 (ddd, J = 13.1, 9.6, 3.8 Hz, 1H), 1.32 (s, 3H).

^{19}F NMR (470 MHz, Methanol-d_4, ppm) δ -77.23 (TFA), -137.80 (d, J = 157.5 Hz), -146.66 (d, J = 157.5 Hz).

^{13}C NMR (126 MHz, Methanol-d_4, ppm) δ 164.6, 150.9, 142.6, 137.3, 132.0, 131.8, 130.7, 130.5, 127.7, 121.1, 116.4 (t, J = 289 Hz), 113.2, 67.2, 49.2, 46.6 (t, J = 172.0 Hz), 45.1, 26.2, 24.8, 23.7 – 23.1 (m), 22.5 – 22.0 (m), 19.2 – 19.0 (m).

Diastereomer 2:

^{1}H NMR (500 MHz, Methanol-d_4, ppm) δ 7.79 (d, J = 1.9 Hz, 1H), 7.60 (dd, J = 18.7, 8.6 Hz, 3H), 7.49 – 7.40 (m, 3H), 5.44 (s, 1H), 3.41 (t, J = 10.9 Hz, 1H), 2.99 (dd, J = 12.8, 3.2 Hz, 1H), 2.93 – 2.83 (m, 1H), 2.55 – 2.37 (m, 1H), 2.14 – 2.05 (m, 1H), 1.95 (tdt, J = 10.1, 6.0, 3.6 Hz, 1H), 1.60 (ddd, J = 12.8, 9.2, 3.4 Hz, 1H), 1.29 (s, 3H).

^{19}F NMR (470 MHz, Methanol-d_4, ppm) δ -77.23, -137.82 (d, J = 156.5 Hz), -146.75 (d, J = 156.5 Hz).

^{13}C NMR (126 MHz, Methanol-d_4, ppm) δ 165.1, 150.8, 142.7, 136.9, 133.2, 132.0, 131.7, 130.5, 127.6, 121.1, 116.7 (t, J = 289.9 Hz), 113.2, 67.2, 47.7 – 47.3 (m), 45.2, 24.5, 24.0 – 23.2 (m), 22.3 – 21.7 (m), 19.6 – 18.5 (m).

HRMS: Calc. $C_{21}H_{19}Cl_2F_2N_2O^+$ [M+H$^+$] 423.0837; found 423.0832.

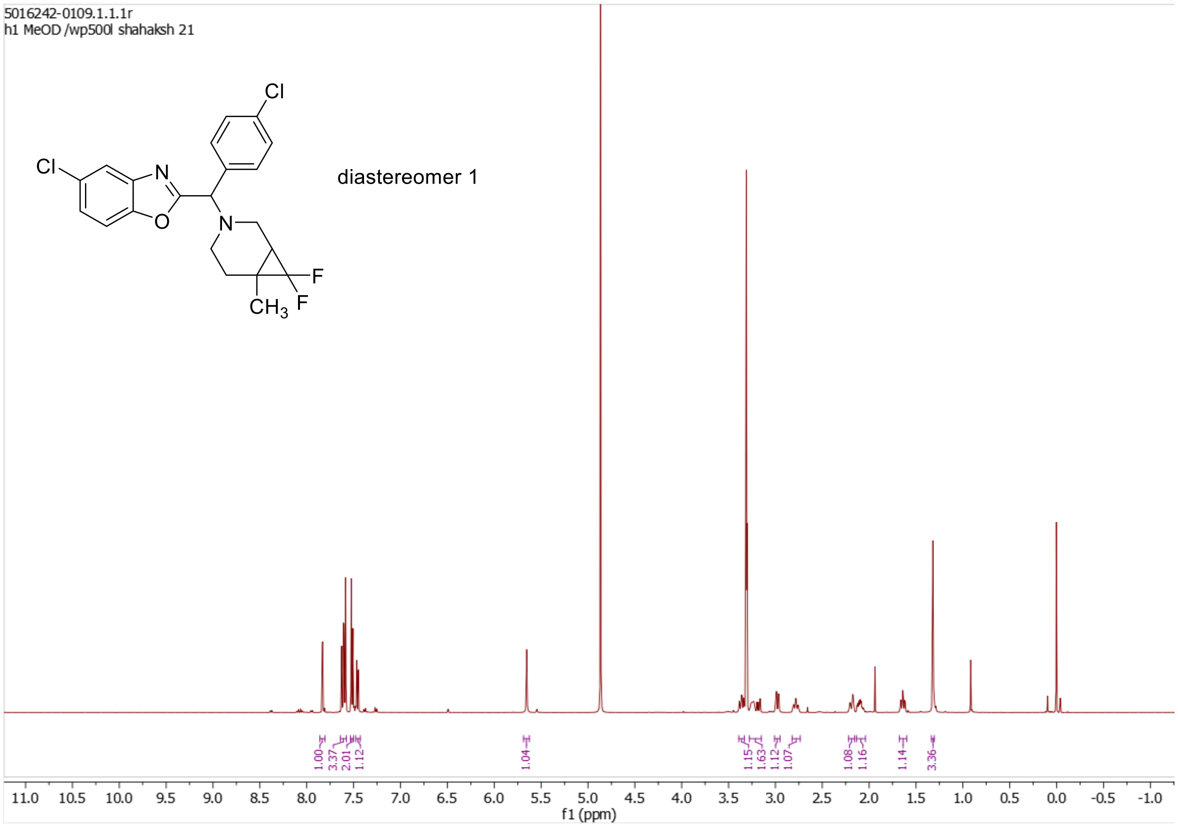
5016242-0109.1.1.1r
h1 MeOD /wp500l shahaksh 21
diastereomer 1
Cl
Cl
N
O
N
F
F
CH3
1.00
3.37
2.01
1.12
1.04
1.15
1.63
1.12
1.07
1.08
1.16
1.14
3.36
11.0 10.5 10.0 9.5 9.0 8.5 8.0 7.5 7.0 6.5 6.0 5.5 5.0 4.5 4.0 3.5 3.0 2.5 2.0 1.5 1.0 0.5 0.0 -0.5 -1.0
f1 (ppm)

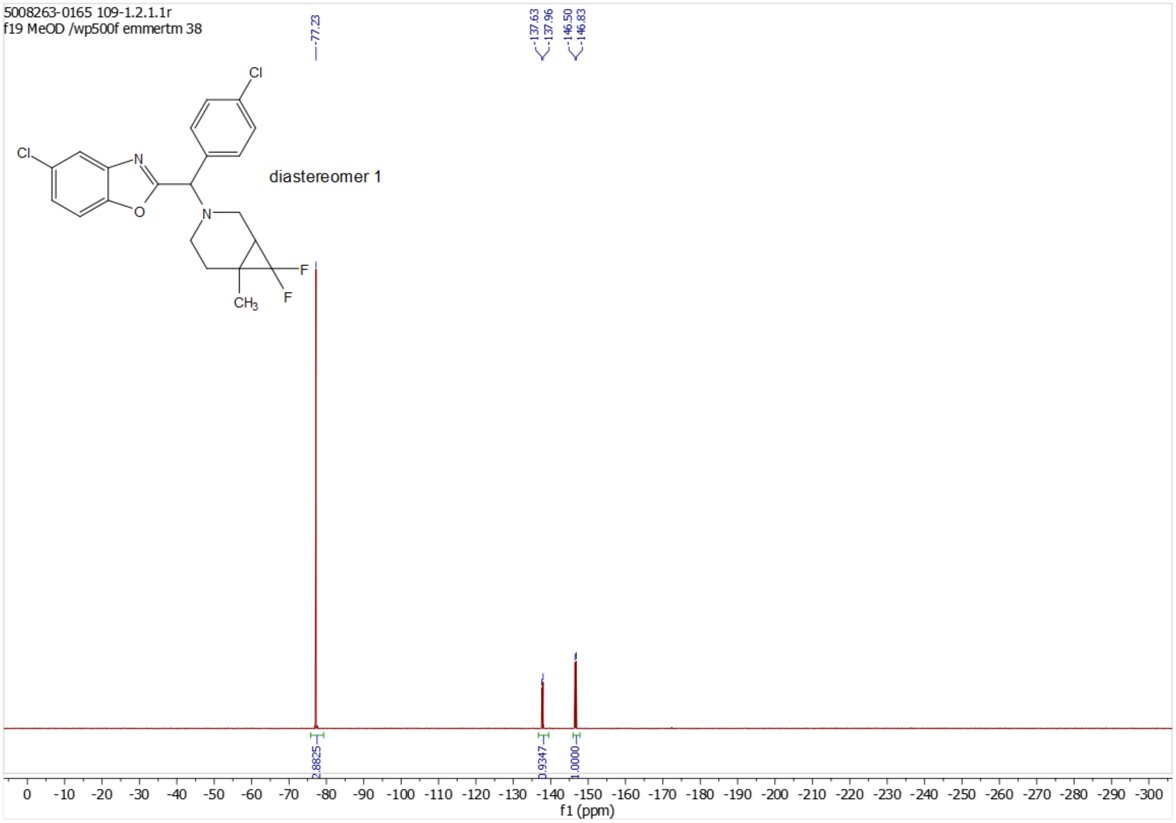
5008263-0165 109-1.2.1.1r
f19 MeOD /wp500f emmertm 38
-77.23
-137.63
-137.96
-146.50
-146.83
diastereomer 1
Cl
Cl
N
O
N
F
F
CH3
2.8825
0.9347
1.0000
0 -10 -20 -30 -40 -50 -60 -70 -80 -90 -100 -110 -120 -130 -140 -150 -160 -170 -180 -190 -200 -210 -220 -230 -240 -250 -260 -270 -280 -290 -300
f1 (ppm)

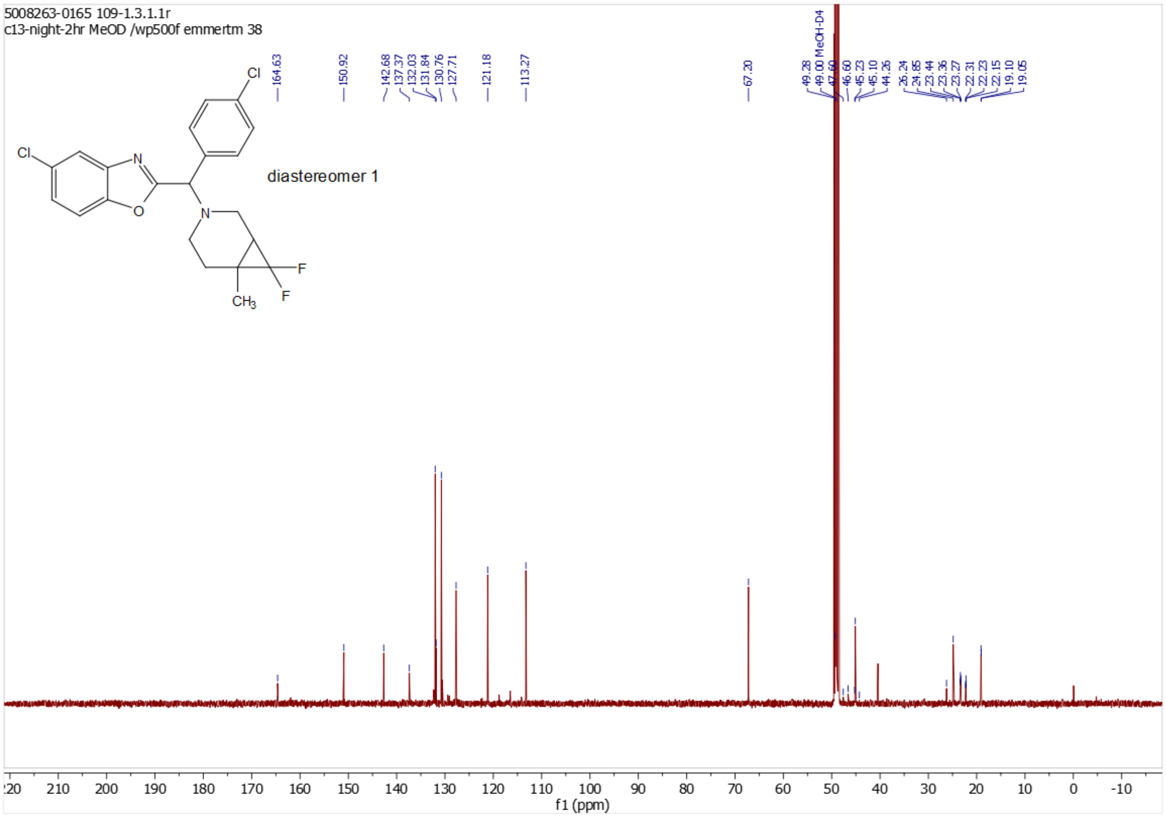
5008263-0165 109-1.3.1.1r
c13-night-2hr MeOD /wp500f emmertm 38
diastereomer 1
f1 (ppm)

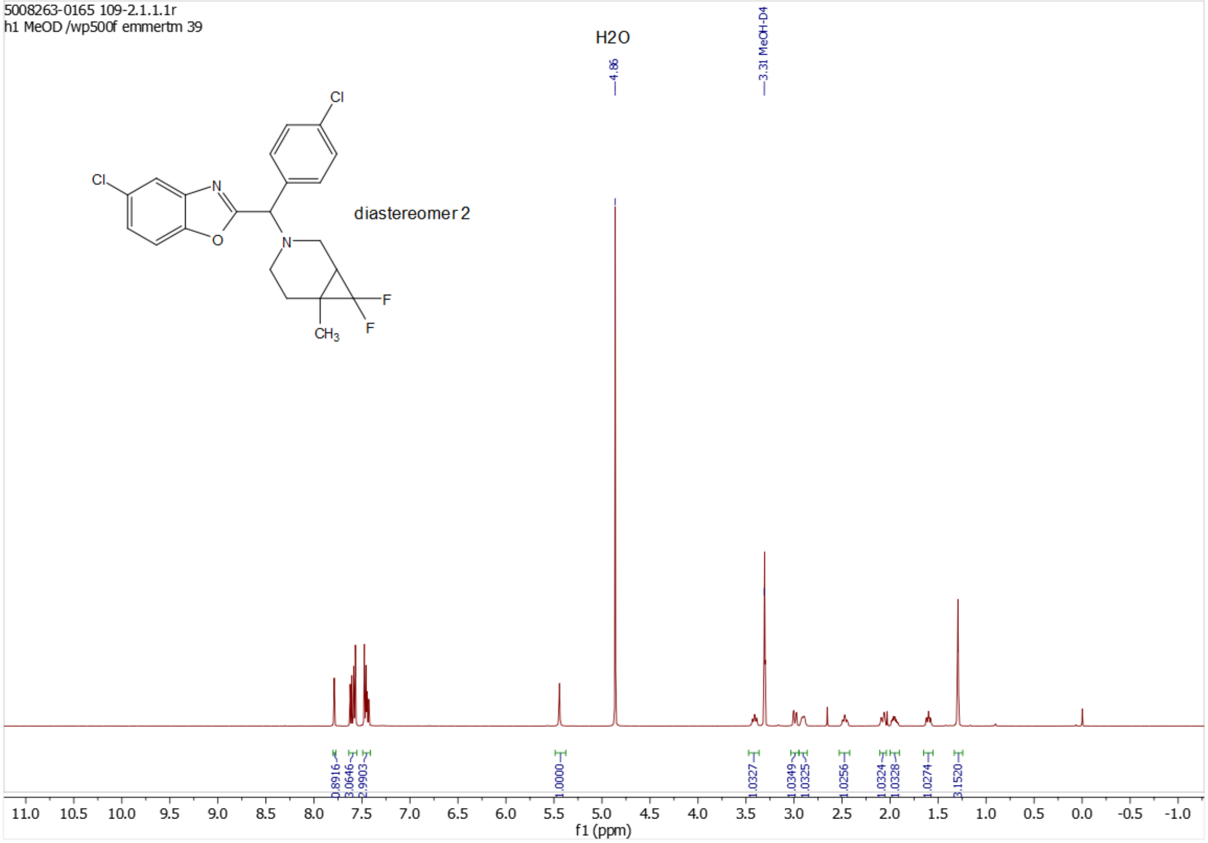
5008263-0165 109-2.1.1.1r
h1 MeOD /wp500f emmertm 39
H2O
diastereomer 2
f1 (ppm)

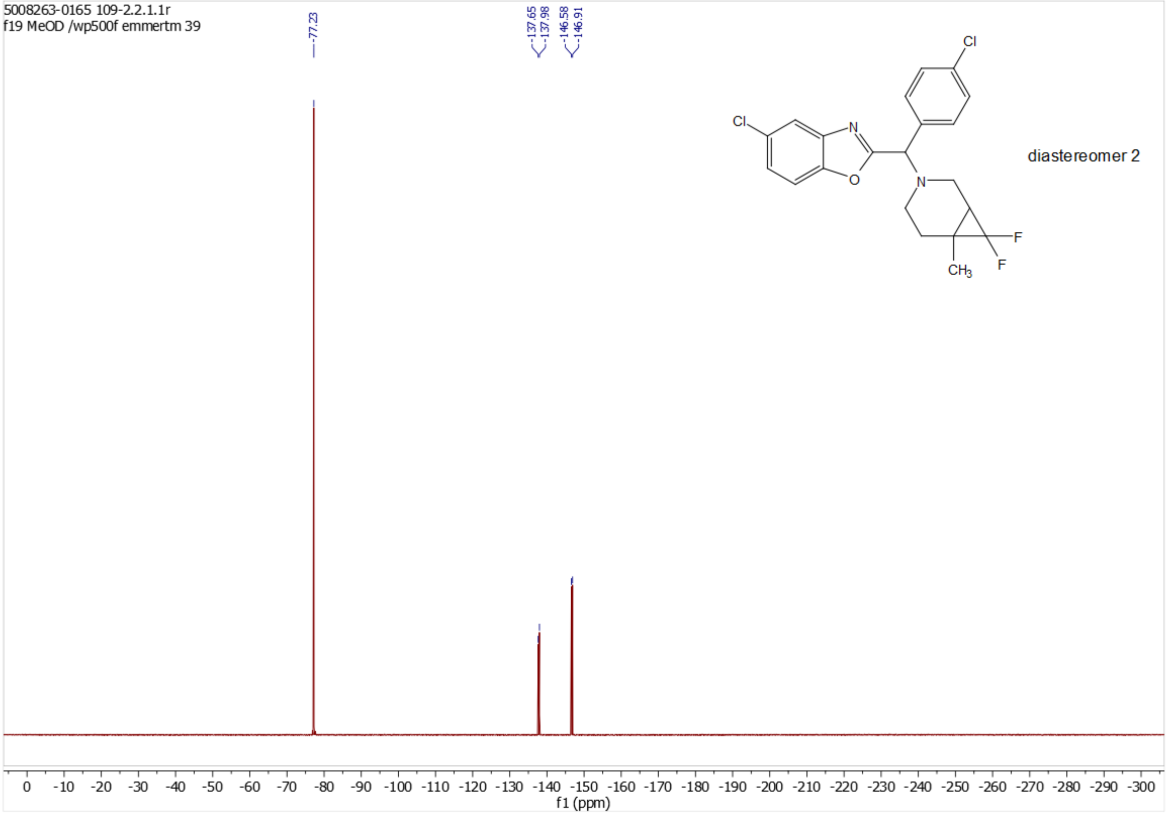
5008263-0165 109-2.2.1.1r
f19 MeOD /wp500f emmertm 39
-77.23
-137.65
-137.98
-146.58
-146.91
Cl
N
O
N
F
F
CH3
Cl
diastereomer 2
0 -10 -20 -30 -40 -50 -60 -70 -80 -90 -100 -110 -120 -130 -140 -150 -160 -170 -180 -190 -200 -210 -220 -230 -240 -250 -260 -270 -280 -290 -300
f1 (ppm)

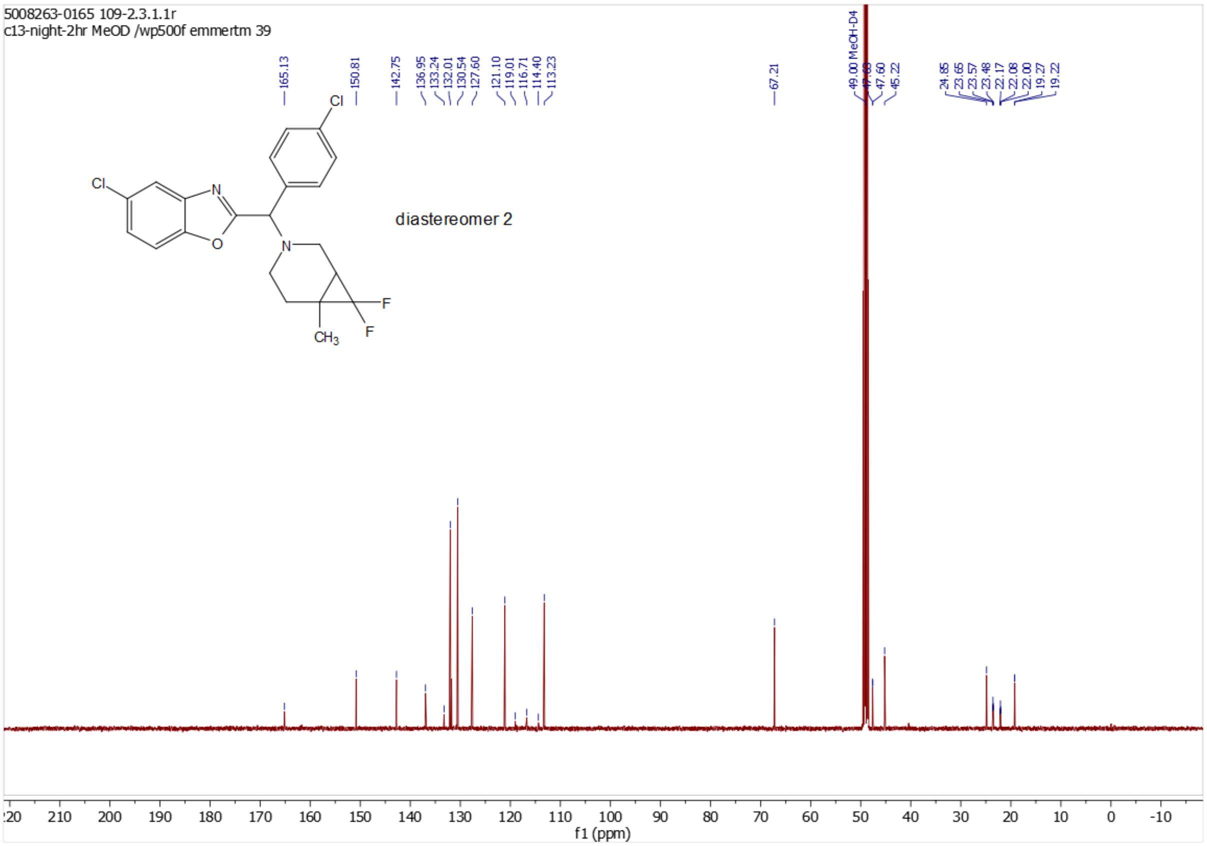
5008263-0165 109-2.3.1.1r
c13-night-2hr MeOD /wp500f emmertm 39
165.13
150.81
142.75
136.95
133.24
132.01
130.54
127.60
121.10
119.01
116.71
114.40
113.23
67.21
49.00 MeOH-D4
47.60
45.22
24.85
23.65
23.57
23.48
22.17
22.08
22.00
19.27
19.22
Cl
N
O
N
F
F
CH3
Cl
diastereomer 2
210 200 190 180 170 160 150 140 130 120 110 100 90 80 70 60 50 40 30 20 10 0 -10
f1 (ppm)

5-Chloro-2-((4-chlorophenyl)(6,6-difluoro-3-azabicyclo[3.1.0]hexan-3-yl)methyl)benzo[d]oxazole

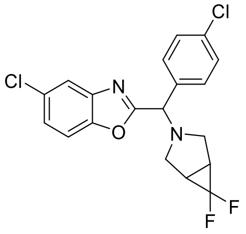

Chemical Formula: $C_{19}H_{14}Cl_2F_2N_2O$
Exact Mass: 394.0451
Molecular Weight: 395.2308

The title compound was prepared according to General Procedure 3 (PMC). Isolated as $C_{19}H_{14}Cl_2F_2N_2O(F_3CCO_2H)_3$ (apparent M_w 395.2308 + 114.0232*3 = 737.3004 g/mol).

Yield: 21 mg (16%); 34 LCAP before workup/column chromatography

^{1}H NMR (500 MHz, Methanol-d_4, ppm) δ 7.76 (d, J = 1.9 Hz, 1H), 7.61 – 7.53 (m, 3H), 7.47 – 7.38 (m, 3H), 5.41 (s, 1H), 3.45 (dd, J = 9.6, 5.4 Hz, 1H), 3.28 (d, J = 4.9 Hz, 1H), 3.19 (t, J = 10.8 Hz, 2H), 2.54 – 2.40 (m, 2H).

^{19}F NMR (470 MHz, Methanol-d_4, ppm) δ -77.42 (TFA), -128.01 (d, J = 157.8 Hz), -151.78 (d, J = 157.8 Hz).

^{13}C NMR (126 MHz, Methanol-d_4, ppm) δ 166.3, 161.1 (q, J = 37.9 Hz), 150.7, 142.7, 136.4, 135.4, 131.5, 131.3, 130.3, 127.4, 120.9, 117.6 (t, J = 189.3 Hz), 114.9 (q, J = 297.5 Hz), 113.1, 66.4, 52.6, 52.0, 28.0 (t, J = 12.8 Hz), 27.9 (t, J = 12.8 Hz).

HRMS: Calc. $C_{19}H_{15}Cl_2F_2N_2O^+$ [M+H$^+$] 395.0524; found 395.0528.

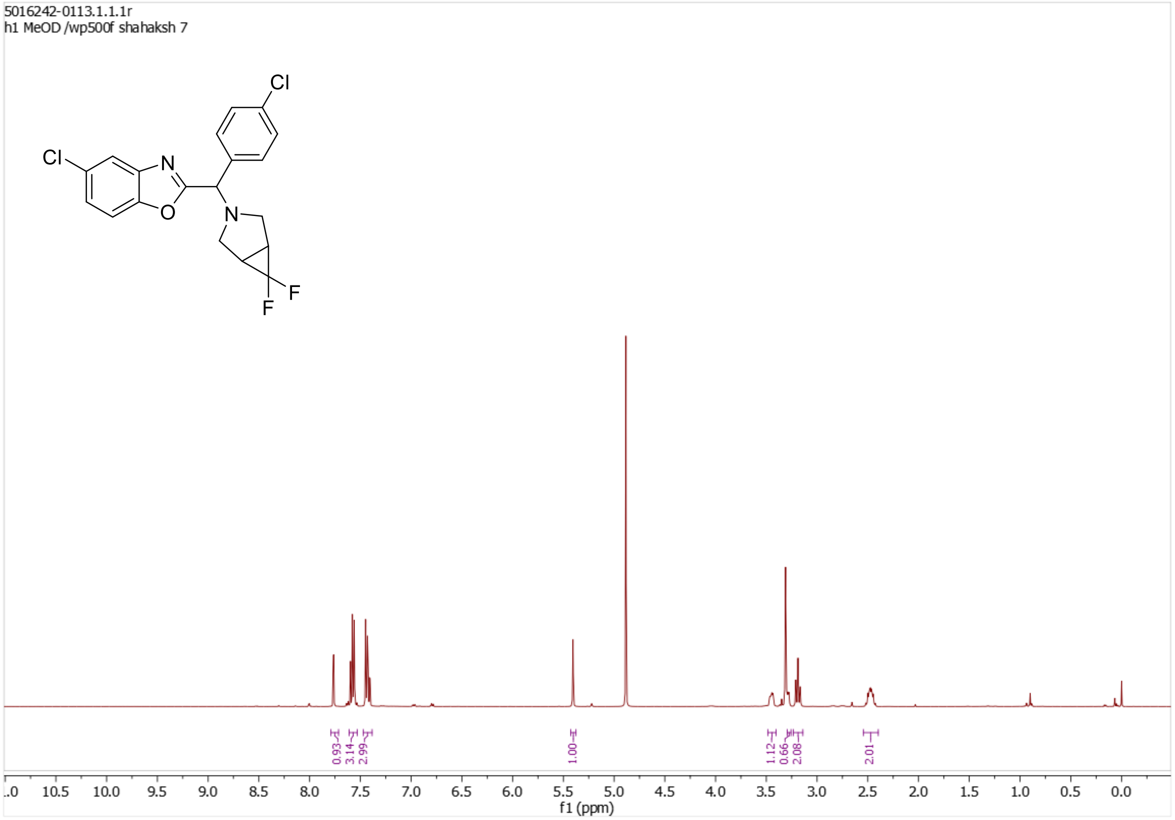
5016242-0113.1.1.1r
h1 MeOD /wp500f shahaksh 7
Cl
Cl
N
O
N
F
F
0.93
3.14
2.99
1.00
1.12
0.66
2.08
2.01
f1 (ppm)

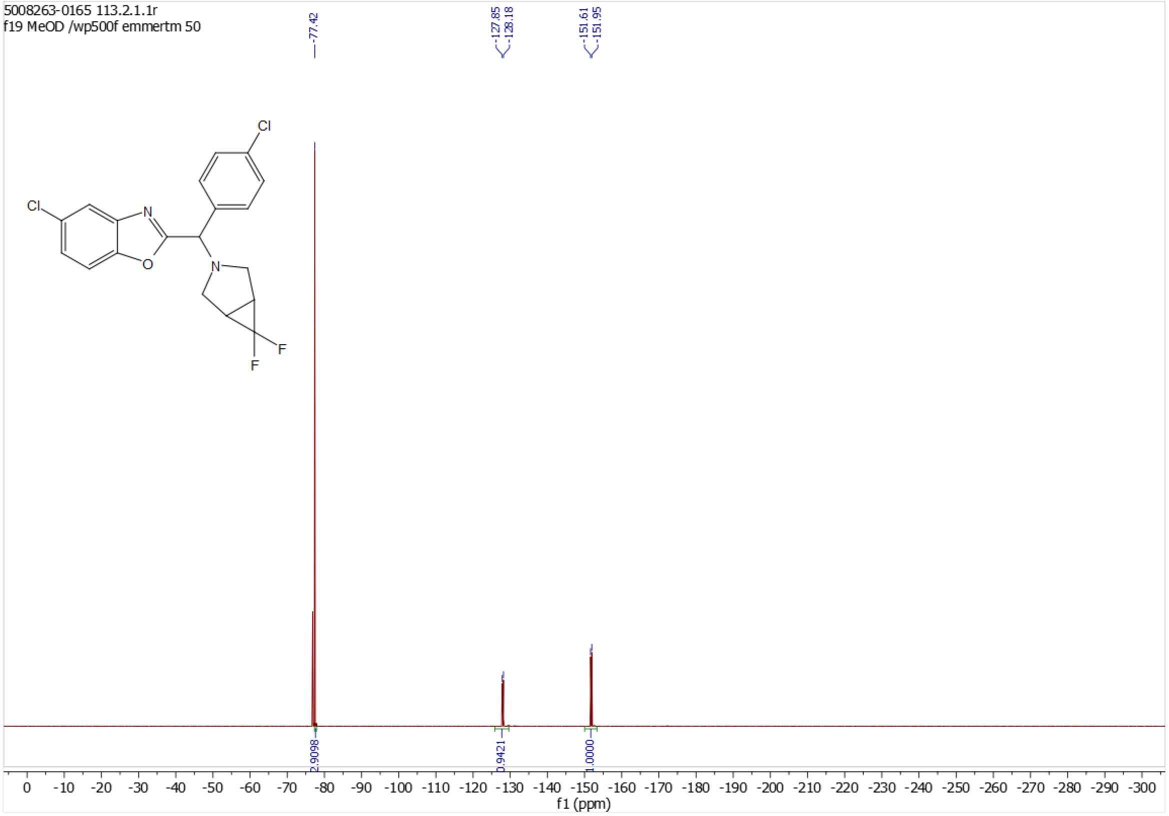
5008263-0165 113.2.1.1r
f19 MeOD /wp500f emmertm 50
-77.42
-127.85
-128.18
-151.61
-151.95
Cl
Cl
N
O
N
F
F
2.9098
0.9421
1.0000
f1 (ppm)

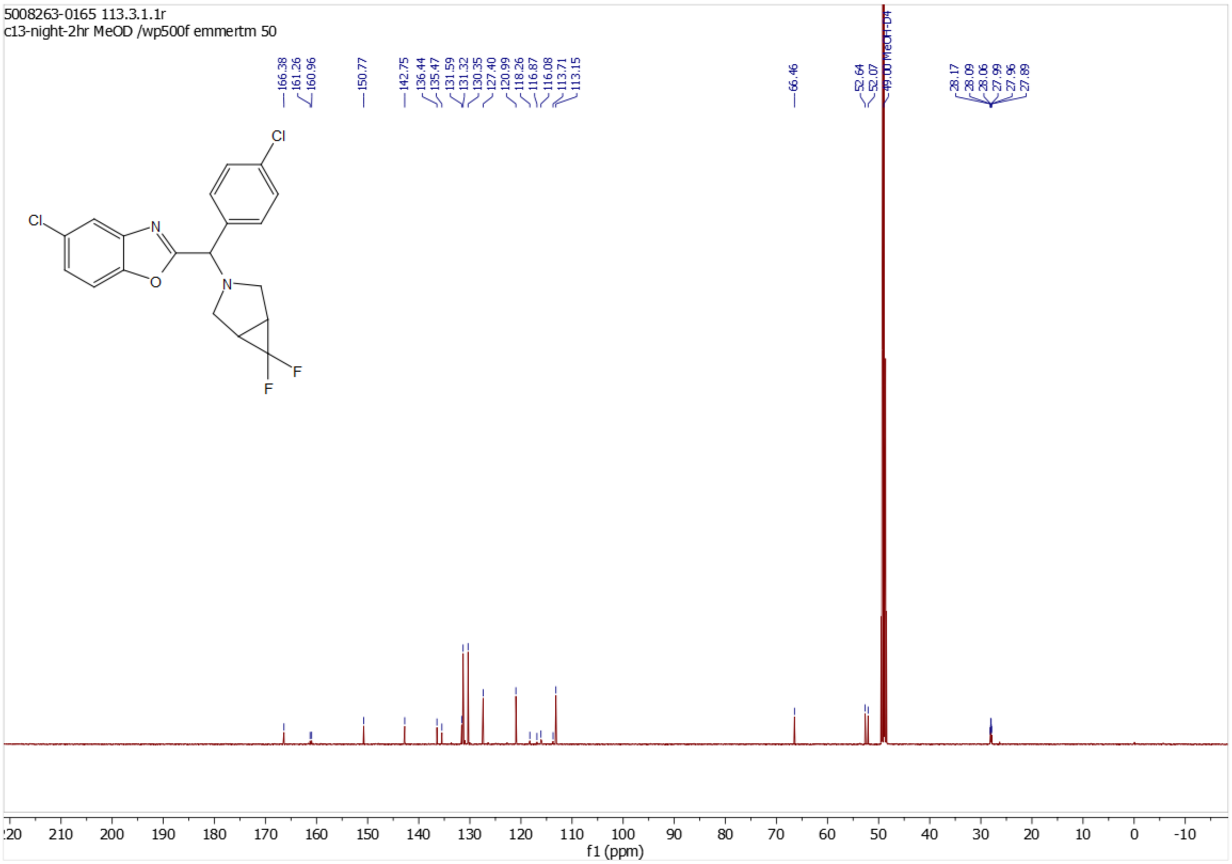

5008263-0165 113.3.1.1r
c13-night-2hr MeOD /wp500f emmertm 50
166.38
161.26
160.96
150.77
142.75
136.44
135.47
131.59
131.32
130.35
127.40
120.99
118.26
116.87
116.08
113.71
113.15
66.46
52.64
52.07
49.00 MeOH-D4
28.17
28.09
28.06
27.99
27.96
27.89
Cl
N
O
F
f1 (ppm)

5-Chloro-2-((4-chlorophenyl)(3,3-difluoro-2-methylpyrrolidin-1-yl)methyl)benzo[d]oxazole

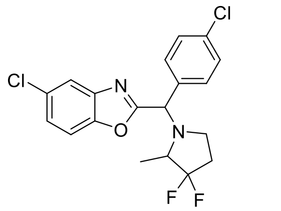

Chemical Formula: $C_{19}H_{16}Cl_2F_2N_2O$
Exact Mass: 396.0608
Molecular Weight: 397.2468

The title compound was prepared according to General Procedure 3 (PMC). Isolated as mixture of two diastereomers (2:1) $C_{19}H_{14}Cl_2F_2N_2O(F_3CCO_2H)_3$ (apparent M_w 397.2468 + 114.0232*3 = 739.3164 g/mol).

Yield: 31 mg (24%); 20 LCAP before workup/column chromatography

^{1}H NMR (500 MHz, Methanol-d_4, ppm) δ 7.93 – 7.14 (m, 7H), 5.46 (s, 0.66H; major diastereomer), 5.22 (s, 0.33H; minor diastereomer), 3.19 – 2.84 (several overlapping signals, 3H), 2.62 – 2.08 (several overlapping signals, 2H), 1.16 (d, *J* = 6.4 Hz, 2H; major diastereomer), 0.94 (dd, *J* = 6.5, 2.9 Hz, 1H; minor diastereomer).

<u>Minor Diastereomer:</u>

^{19}F NMR (470 MHz, Methanol-d_4, ppm) δ -76.84 (TFA), -96.54 (d, *J* = 228.7 Hz), -105.99 (d, *J* = 228.7 Hz).

<u>Major Diastereomer:</u>

^{19}F NMR (470 MHz, Methanol-d_4, ppm) δ -77.61 (TFA), -98.45 (d, *J* = 228.3 Hz), -106.37 (d, *J* = 228.3 Hz).

^{13}C NMR (126 MHz, Methanol-d_4, ppm): Due to many overlapping peaks and fluorine couplings, the 13C NMR could not successfully be interpreted.

HRMS: Calc. $C_{19}H_{17}Cl_2F_2N_2O^+$ [M+H$^+$] 397.0681; found 397.0677.

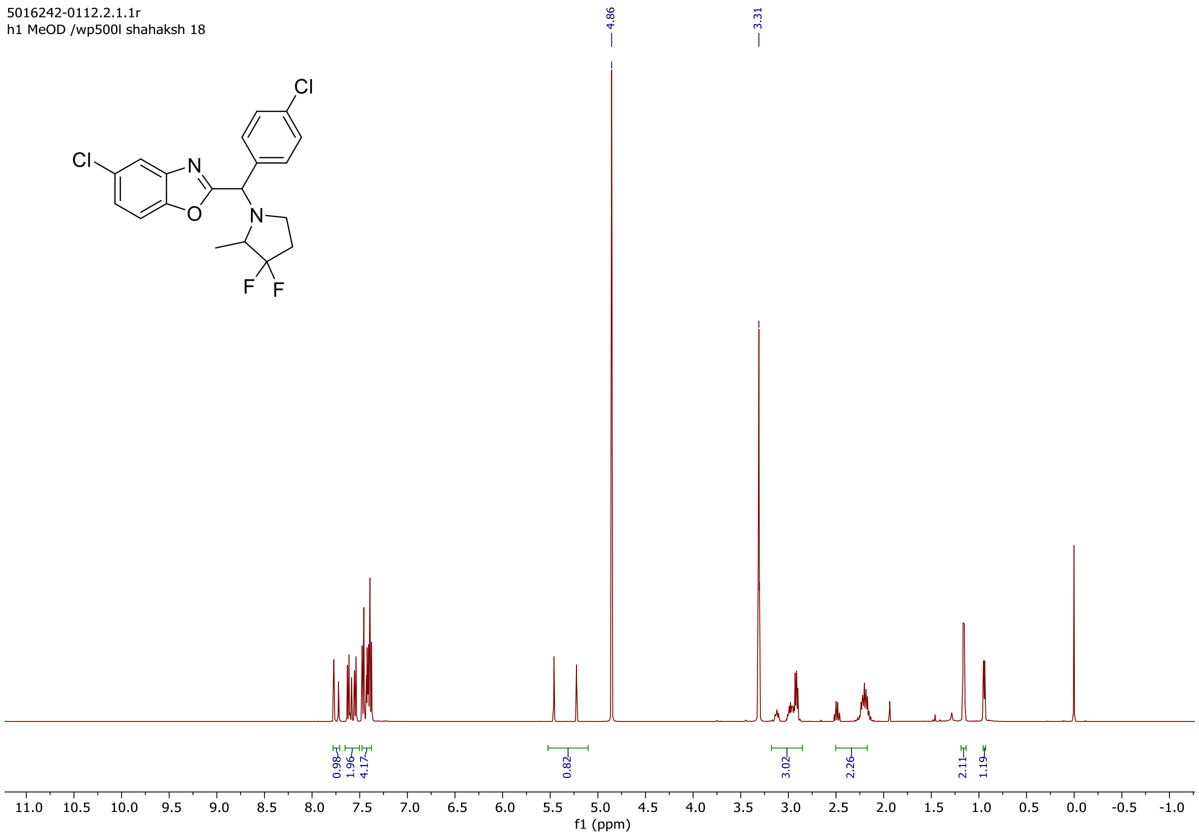
5016242-0112.2.1.1r
h1 MeOD /wp500l shahaksh 18
4.86
3.31
Cl
Cl
N
O
N
F
F
0.98
1.96
4.17
0.82
3.02
2.26
2.11
1.19
11.0 10.5 10.0 9.5 9.0 8.5 8.0 7.5 7.0 6.5 6.0 5.5 5.0 4.5 4.0 3.5 3.0 2.5 2.0 1.5 1.0 0.5 0.0 -0.5 -1.0
f1 (ppm)

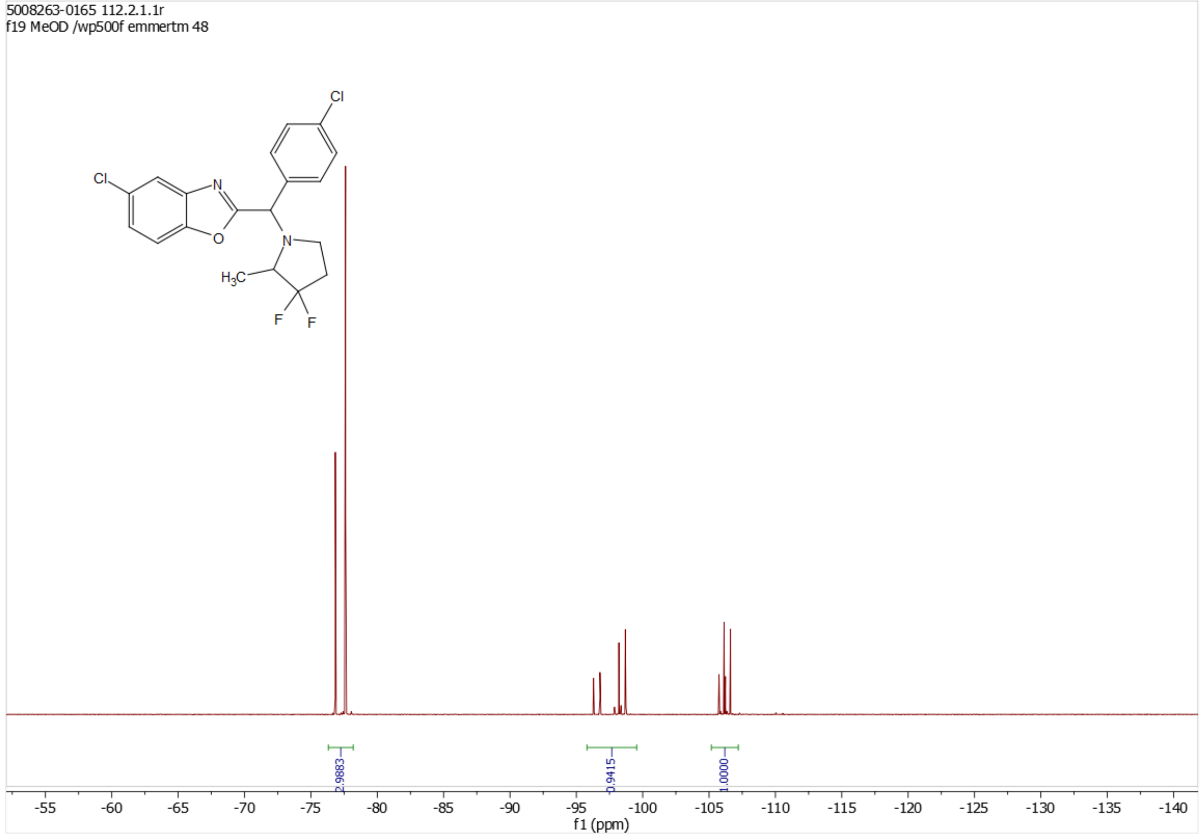
5008263-0165 112.2.1.1r
f19 MeOD /wp500f emmertm 48
Cl
Cl
N
O
N
H_3C
F
F
2.9883
0.9415
1.0000
-55 -60 -65 -70 -75 -80 -85 -90 -95 -100 -105 -110 -115 -120 -125 -130 -135 -140
f1 (ppm)

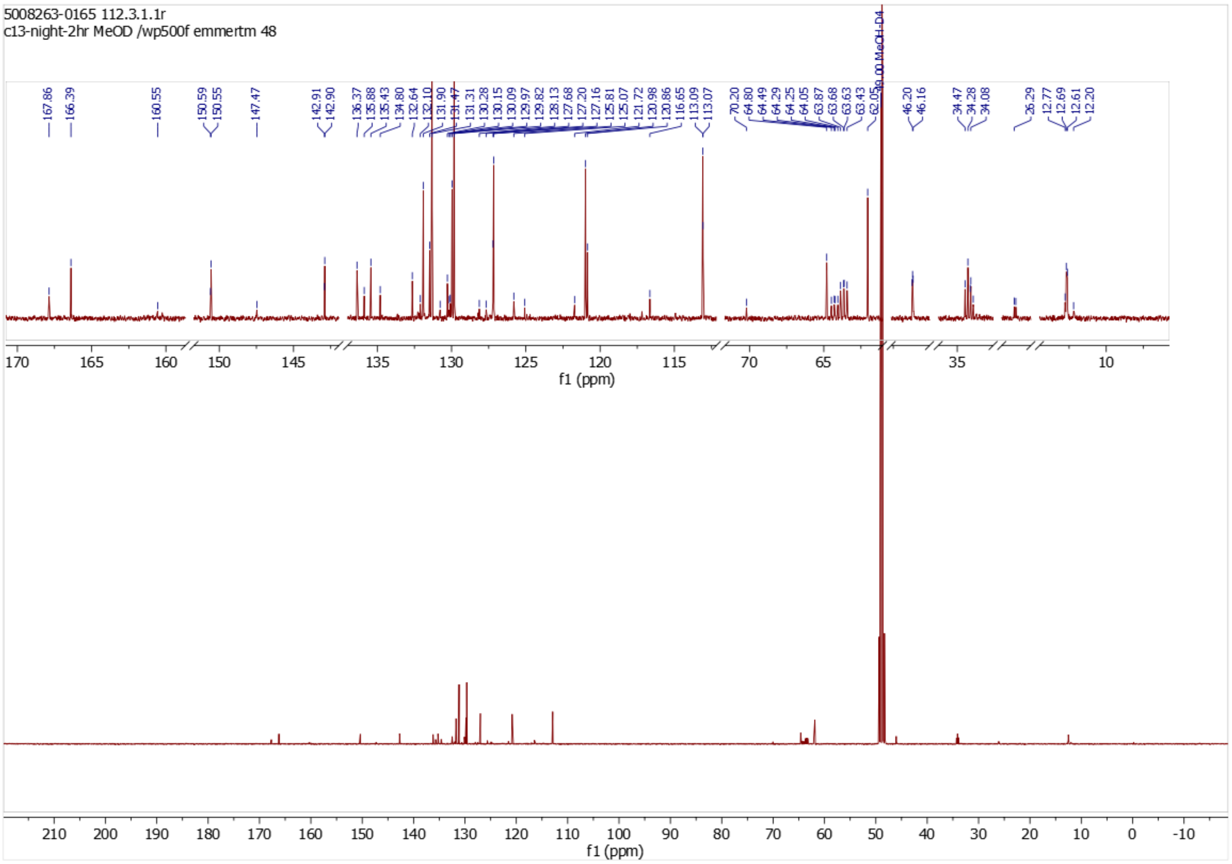

5008263-0165 112.3.1.1r
c13-night-2hr MeOD /wp500f emmertm 48
f1 (ppm)
f1 (ppm)

5-Chloro-2-((4-chlorophenyl)(7-(trifluoromethyl)-1,4-oxazepan-4-yl)methyl)benzo[d]oxazole

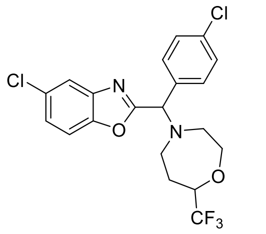

Chemical Formula: $C_{20}H_{17}Cl_2F_3N_2O_2$
Exact Mass: 444.0619
Molecular Weight: 445.2632

The title compound was prepared according to General Procedure 3 (PMC). Isolated as ~3:2 mixture of diastereomers $C_{20}H_{17}Cl_2F_3N_2O_2(F_3CCO_2H)_{1.3}$ (apparent M_w 444.0619 + 114.0232*1.3 = 592.2621 g/mol).

Yield: 35.3 mg (34%); 41 LCAP before workup/column chromatography

^{1}H NMR (500 MHz, Methanol-d_4, ppm) δ 7.78 (d, *J* = 1.9 Hz, 1H), 7.61 (d, *J* = 8.7 Hz, 1H), 7.55 (dd, *J* = 8.5, 3.1 Hz, 2H), 7.44 (dd, *J* = 10.3, 7.8 Hz, 3H), 5.66 (s, 0.6H; major diastereomer), 5.66 (s, 0.4H; minor diastereomer), 4.29 (hept, *J* = 7.2 Hz, 1H), 4.08 (ddt, *J* = 13.4, 8.7, 4.7 Hz, 1H), 3.80 (dddd, *J* = 16.1, 13.6, 6.7, 2.9 Hz, 1H), 3.30 – 2.97 (m, 4H), 2.25 – 2.14 (m, 2H).

^{19}F NMR (470 MHz, Methanol-d_4, ppm) δ -76.83 (TFA), -77.45 (TFA), -79.95 (minor), -79.99 (major).

^{13}C NMR (126 MHz, Methanol-d_4, ppm) δ 166.1 (minor), 166.0 (major), 161.0 (q, *J* = 37.9 Hz; TFA), 158.9 (q, *J* = 42.7 Hz; TFA), 150.7, 142.8, 136.4, 134.5, 131.8 (major), 131.8 (minor), 131.6, 130.3, 127.4, 126.2 (q, *J* = 281.2 Hz; CF_3), 121.0, 117.1 (q, *J* = 288.3 Hz), 116.0 (q, *J* = 284.4 Hz; TFA), 113.1, 76.7 (q, *J* = 30.3 Hz; major), 76.7 (q, *J* = 30.3 Hz; minor), 68.5 (major), 68.4 (minor), 67.9 (major), 67.8 (minor), 51.5 (major), 51.4 (minor), 28.3.

HRMS: Calc. $C_{20}H_{18}Cl_2F_3N_2O_2^+$ [M+H$^+$] 445.0692; found 445.0678.

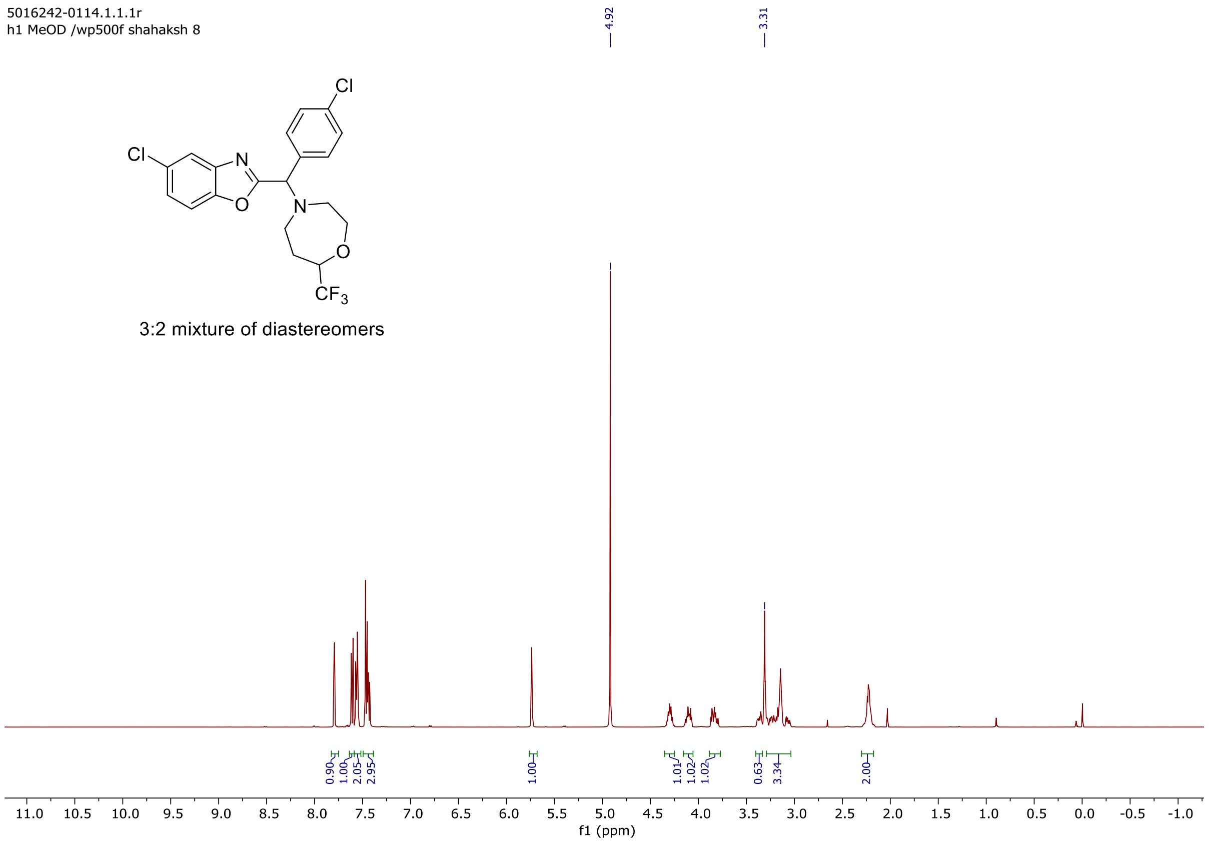
5016242-0114.1.1.1r
h1 MeOD /wp500f shahaksh 8
4.92
3.31
3:2 mixture of diastereomers
0.90
1.00
2.05
2.95
1.00
1.01
1.02
1.02
0.63
3.34
2.00
f1 (ppm)

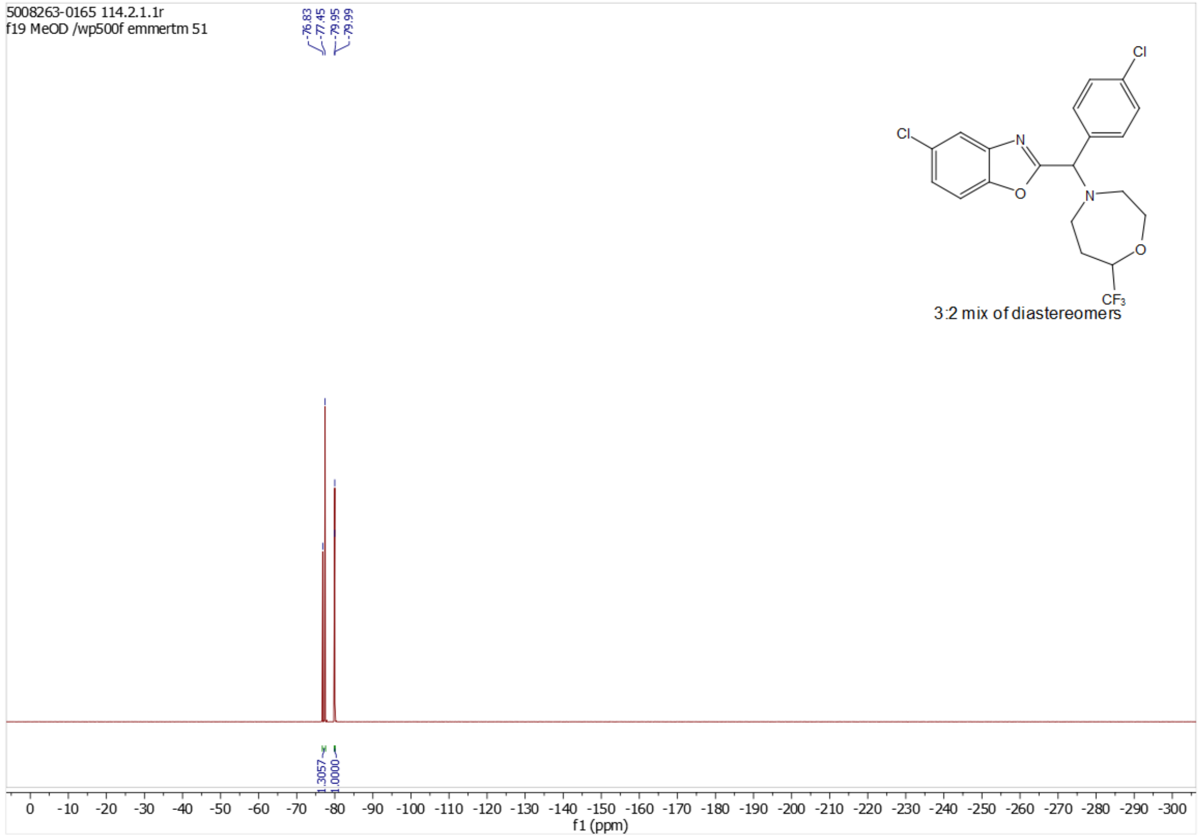
5008263-0165 114.2.1.1r
f19 MeOD /wp500f emmertm 51
-76.83
-77.45
-79.95
-79.99
3:2 mix of diastereomers
1.3057
1.0000
f1 (ppm)

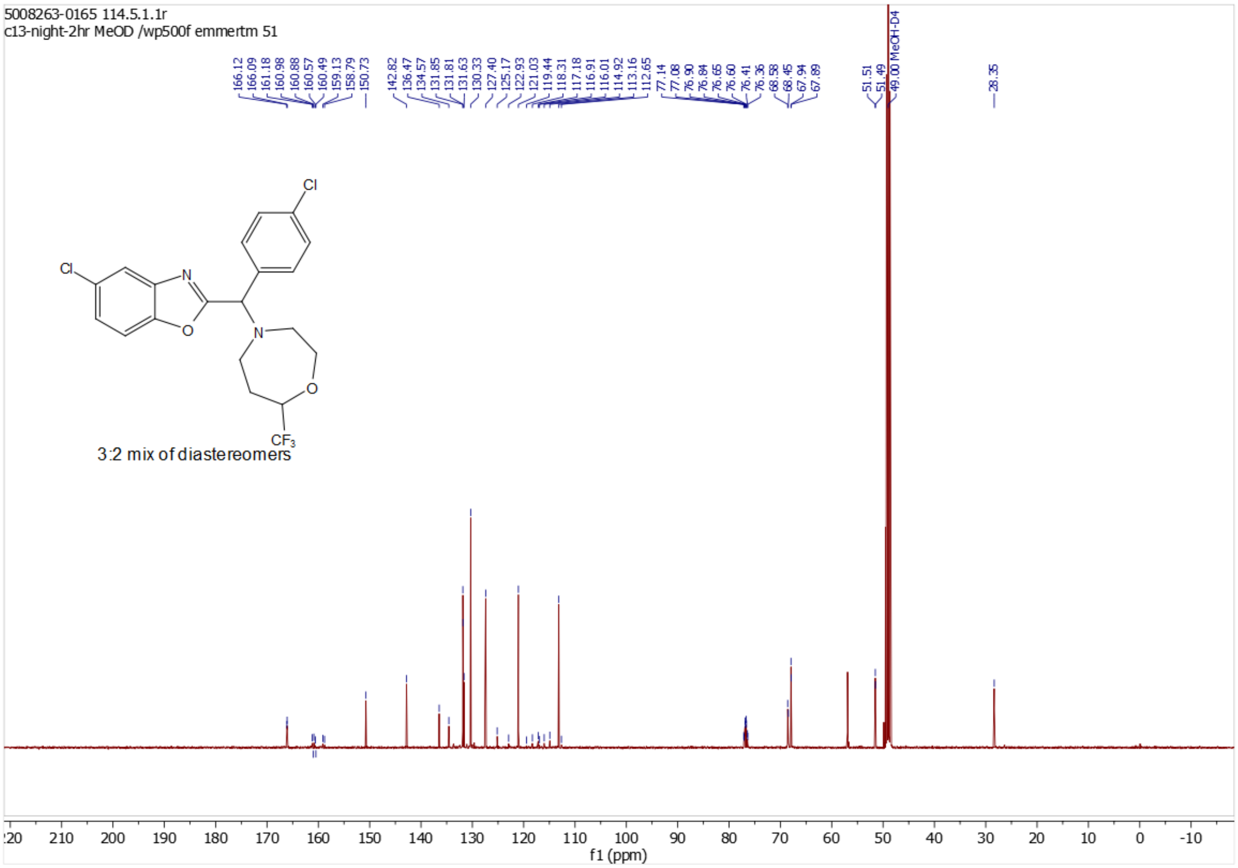

5008263-0165 114.5.1.1r
c13-night-2hr MeOD /wp500f emmertm 51
3:2 mix of diastereomers
f1 (ppm)

5-Chloro-2-((4-chlorophenyl)(6,6-difluoro-3-azabicyclo[3.1.1]heptan-3-yl)methyl)benzo[d]oxazole

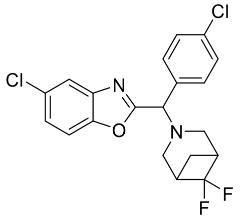

Chemical Formula: $C_{20}H_{16}Cl_2F_2N_2O$
Exact Mass: 408.0608
Molecular Weight: 409.2578

The title compound was prepared according to General Procedure 3 (PMC). Isolated as $C_{20}H_{16}Cl_2F_2N_2O(F_3CCO_2H)_3$ (apparent M_w 409.2578 + 114.0232*3 = 751.3274 g/mol).

Yield: 7.8 mg (6%); 20 LCAP before workup/column chromatography

^{1}H NMR (500 MHz, Methanol-d_4, ppm) δ 7.73 (d, J = 1.9 Hz, 1H), 7.61 (d, J = 8.7 Hz, 1H), 7.54 (d, J = 8.5 Hz, 2H), 7.43 – 7.37 (m, 3H), 5.21 (s, 1H), 3.22 (d, J = 10.4 Hz, 1H), 3.18 – 3.07 (m, 2H), 2.90 (d, J = 10.4 Hz, 1H), 2.70 (s, 2H), 2.25 (dd, J = 16.9, 9.0 Hz, 1H), 1.86 – 1.77 (m, 1H).

^{19}F NMR (470 MHz, Methanol-d_4, ppm) δ -77.53 (TFA), -106.61 (d, J = 181.7 Hz), -124.17 (d, J = 181.8 Hz).

^{13}C NMR (126 MHz, Methanol-d_4, ppm) δ 167.8, 150.6, 142.9, 136.3, 135.5, 131.4, 131.4, 129.9, 127.0, 123.6 (m), 120.8, 113.0, 66.6, 51.5 (dd, J = 6.2, 1.7 Hz), 50.0 (dd, J = 6.2, 1.7 Hz), 44.9 (t, J = 20.0 Hz), 44.8 (t, J = 19.8 Hz), 20.5 (d, J = 13.9 Hz).

HRMS: Calc. $C_{20}H_{17}Cl_2F_2N_2O^+$ [M+H$^+$] 409.0681; found 409.0680.

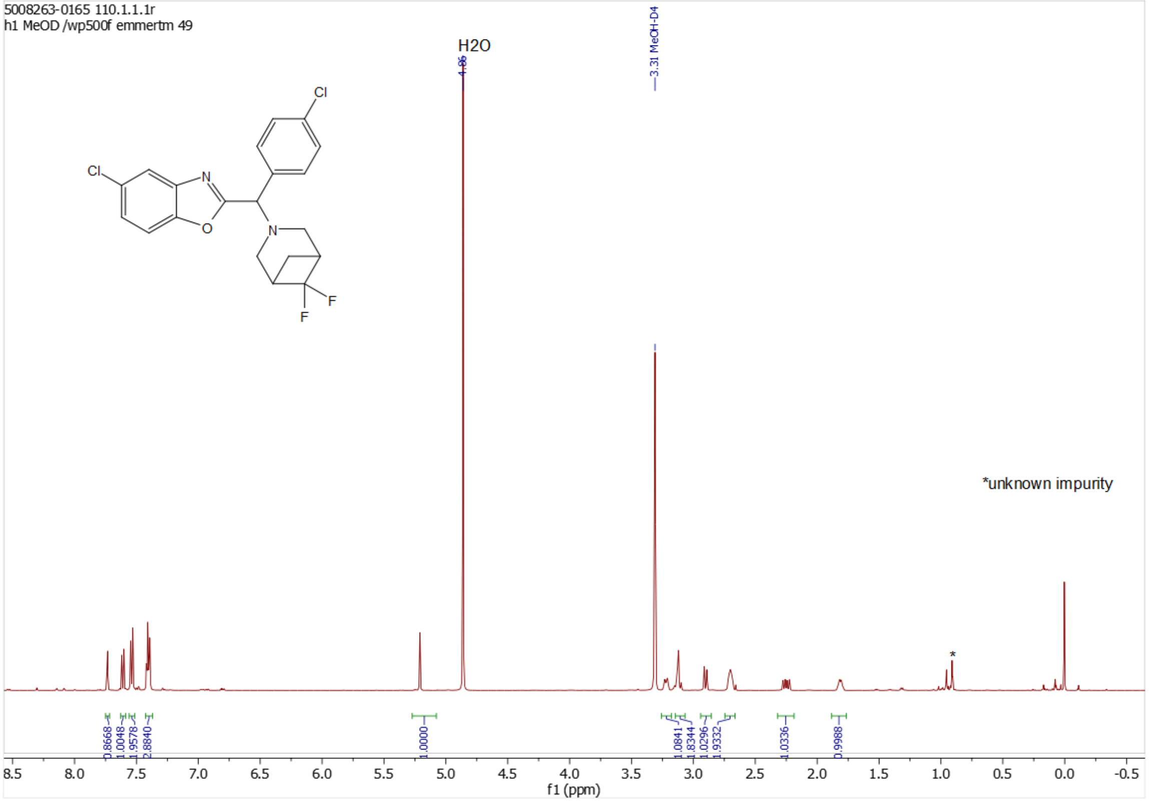
5008263-0165 110.1.1.1r
h1 MeOD /wp500f emmertm 49
H2O
4.86
3.31 MeOH-D4
Cl
Cl
N
O
N
F
F
*unknown impurity
*
0.8668
1.0048
1.9578
2.8840
1.0000
1.0841
1.8344
1.0296
1.9332
1.0336
0.9988
f1 (ppm)

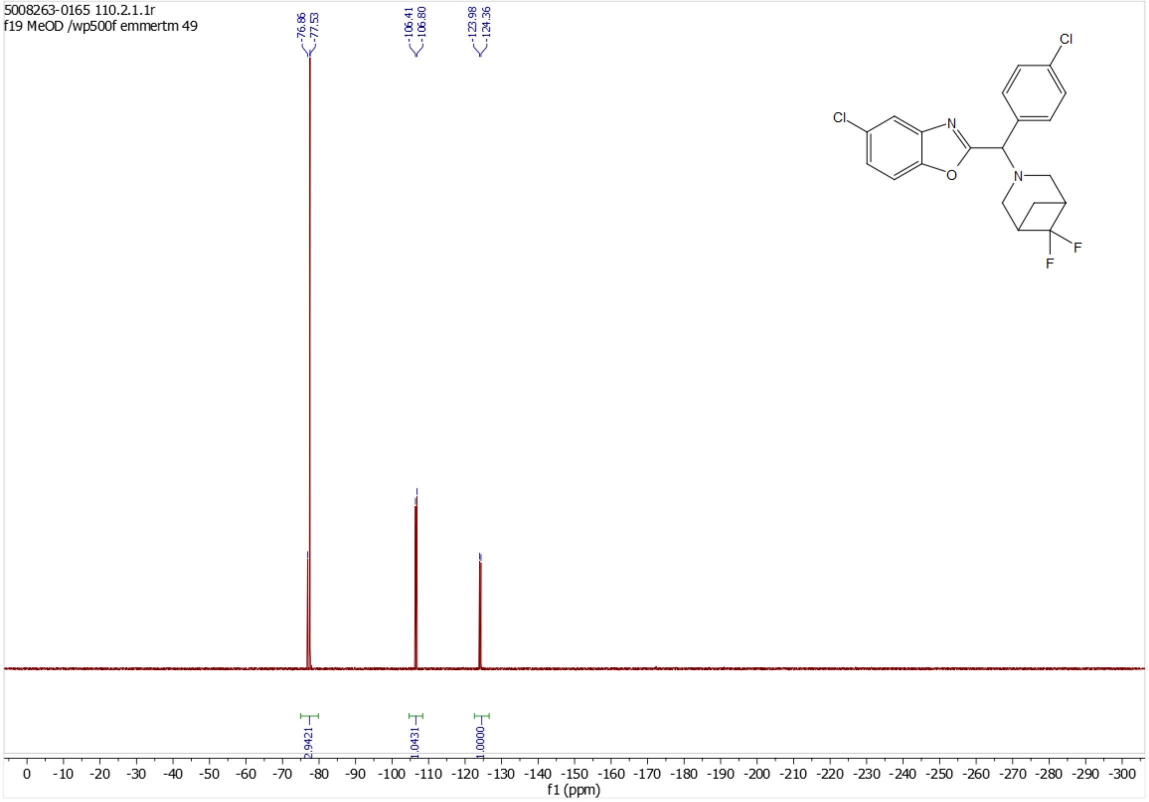
5008263-0165 110.2.1.1r
f19 MeOD /wp500f emmertm 49
-76.86
-77.53
-106.41
-106.80
-123.98
-124.36
Cl
Cl
N
O
N
F
F
2.9421
1.0431
1.0000
f1 (ppm)

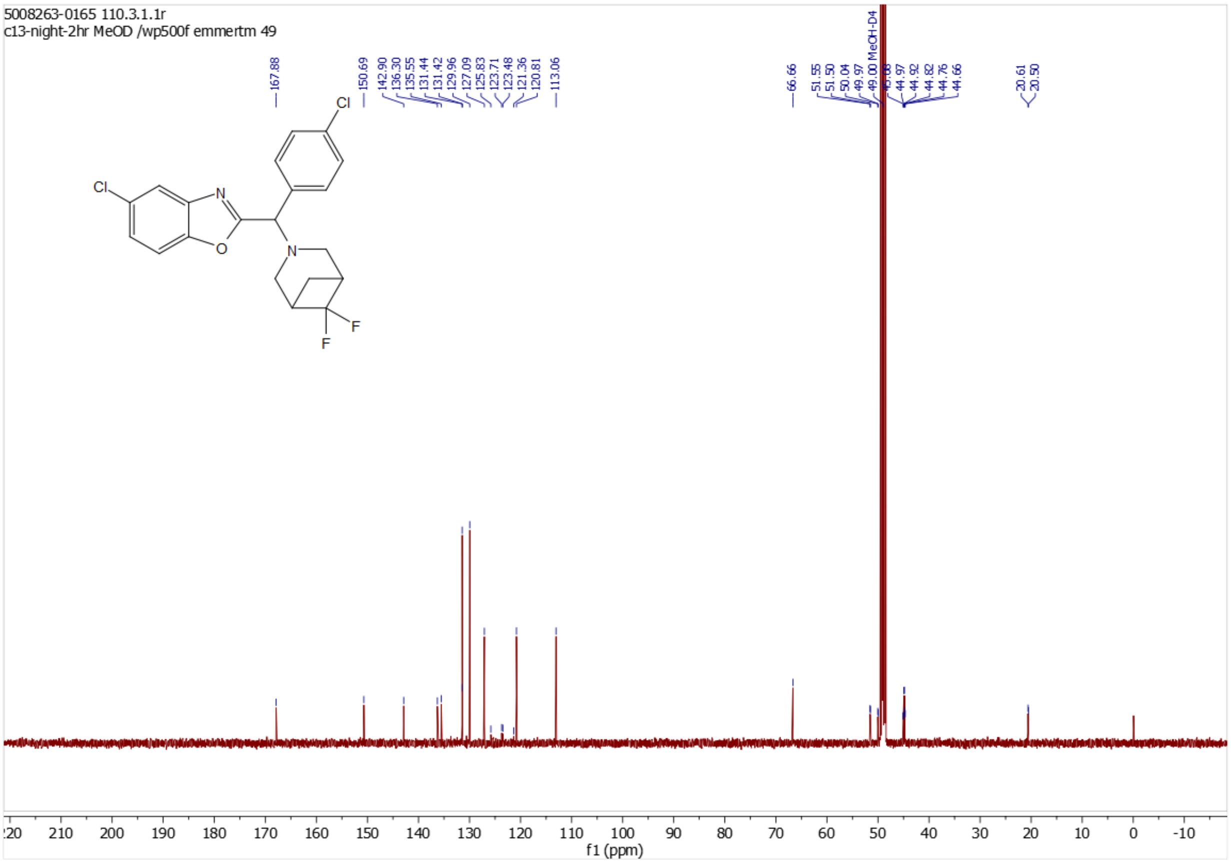
5008263-0165 110.3.1.1r
c13-night-2hr MeOD /wp500f emmertm 49
167.88
150.69
142.90
136.30
135.55
131.44
131.42
129.96
127.09
125.83
123.71
123.48
121.36
120.81
113.06
66.66
51.55
51.50
50.04
49.97
49.00 MeOH-D4
44.97
44.92
44.82
44.76
44.66
20.61
20.50
Cl
Cl
N
O
N
F
F
f1 (ppm)

5-Chloro-2-((4-chlorophenyl)(8,8-difluoro-3-azabicyclo[3.2.1]octan-3-yl)methyl)benzo[d]oxazole

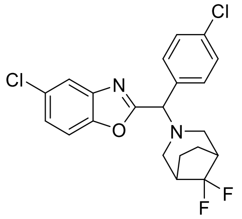

Chemical Formula: $C_{21}H_{18}Cl_2F_2N_2O$
Exact Mass: 422.0764
Molecular Weight: 423.2848

The title compound was prepared according to General Procedure 3 (PMC). Isolated as $C_{21}H_{18}Cl_2F_2N_2O(F_3CCO_2H)_{3.5}$ (apparent M_w 423.2848 + 114.0232*3.5 = 822.3660 g/mol).

Yield: 19.4 mg (13%); 23 LCAP before workup/column chromatography

^{1}H NMR (500 MHz, Methanol-d_4, ppm) δ 7.71 (d, *J* = 1.9 Hz, 1H), 7.60 (d, *J* = 8.7 Hz, 1H), 7.54 (d, *J* = 8.4 Hz, 2H), 7.39 (dd, *J* = 8.7, 2.1 Hz, 3H), 5.07 (s, 1H), 2.76 (t, *J* = 11.9 Hz, 2H), 2.57 (d, *J* = 10.8 Hz, 2H), 2.12 (s, 2H), 1.95 – 1.88 (m, 2H), 1.88 – 1.79 (m, 2H).

^{19}F NMR (470 MHz, Methanol-d_4, ppm) δ -77.64 (TFA), -108.80 (d, *J* = 214.9 Hz), -128.04 (d, *J* = 214.9 Hz).

^{13}C NMR (126 MHz, Methanol-d_4, ppm) δ 167.8, 150.6, 146.8, 142.9, 136.8, 135.4, 132.3, 131.3, 130.2 (dd, *J* = 257.5, 249.3 Hz), 129.9, 127.0, 120.8, 113.0, 67.6, 53.7 (dd, *J* = 169.9, 9.1 Hz), 41.1 (ddd, *J* = 19.9, 17.9, 14.3 Hz), 24.8 (dd, *J* = 19.5, 6.1 Hz).

HRMS: Calc. $C_{21}H_{19}Cl_2F_2N_4O^+$ [M+H$^+$] 423.0837; found 423.0821.

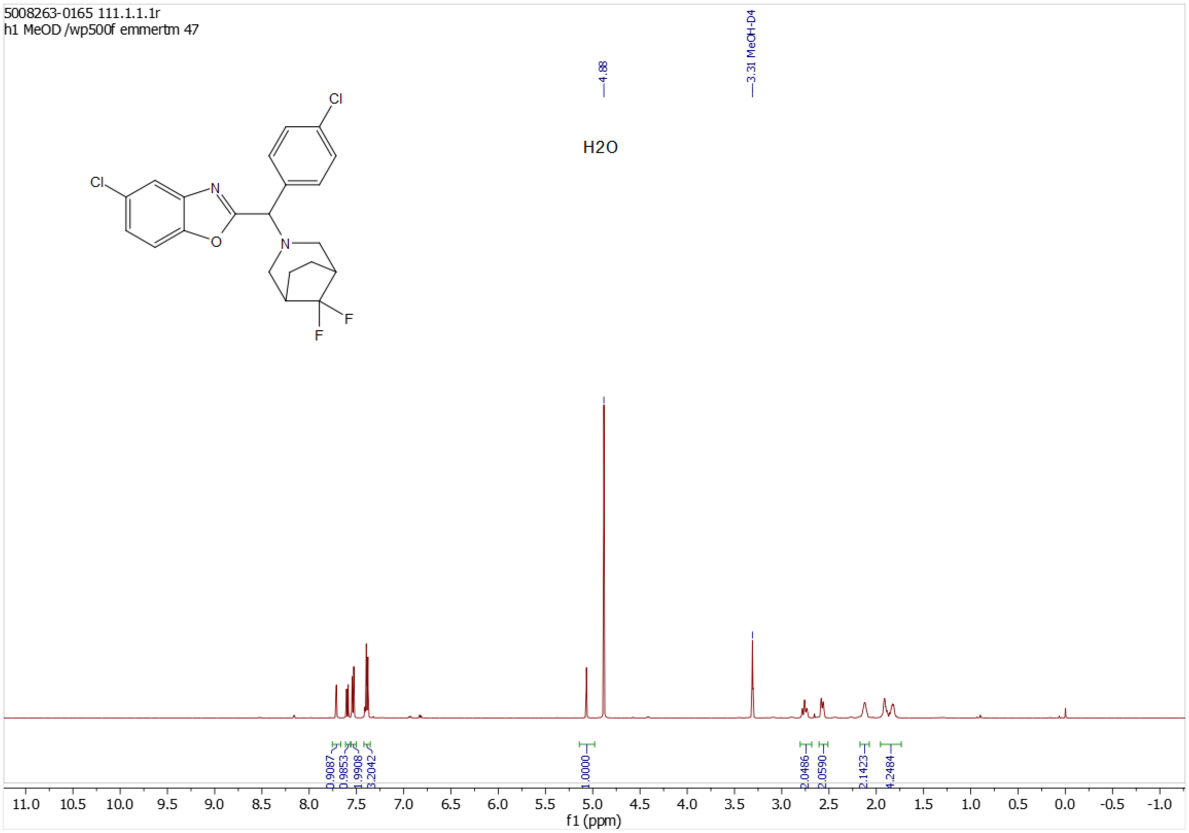
5008263-0165 111.1.1.1r
h1 MeOD /wp500f emmertm 47
4.88
3.31 MeOH-D4
H2O
Cl
Cl
N
O
N
F
F
0.9087
0.9853
1.9908
3.2042
1.0000
2.0486
2.0590
2.1423
4.2484
11.0 10.5 10.0 9.5 9.0 8.5 8.0 7.5 7.0 6.5 6.0 5.5 5.0 4.5 4.0 3.5 3.0 2.5 2.0 1.5 1.0 0.5 0.0 -0.5 -1.0
f1 (ppm)

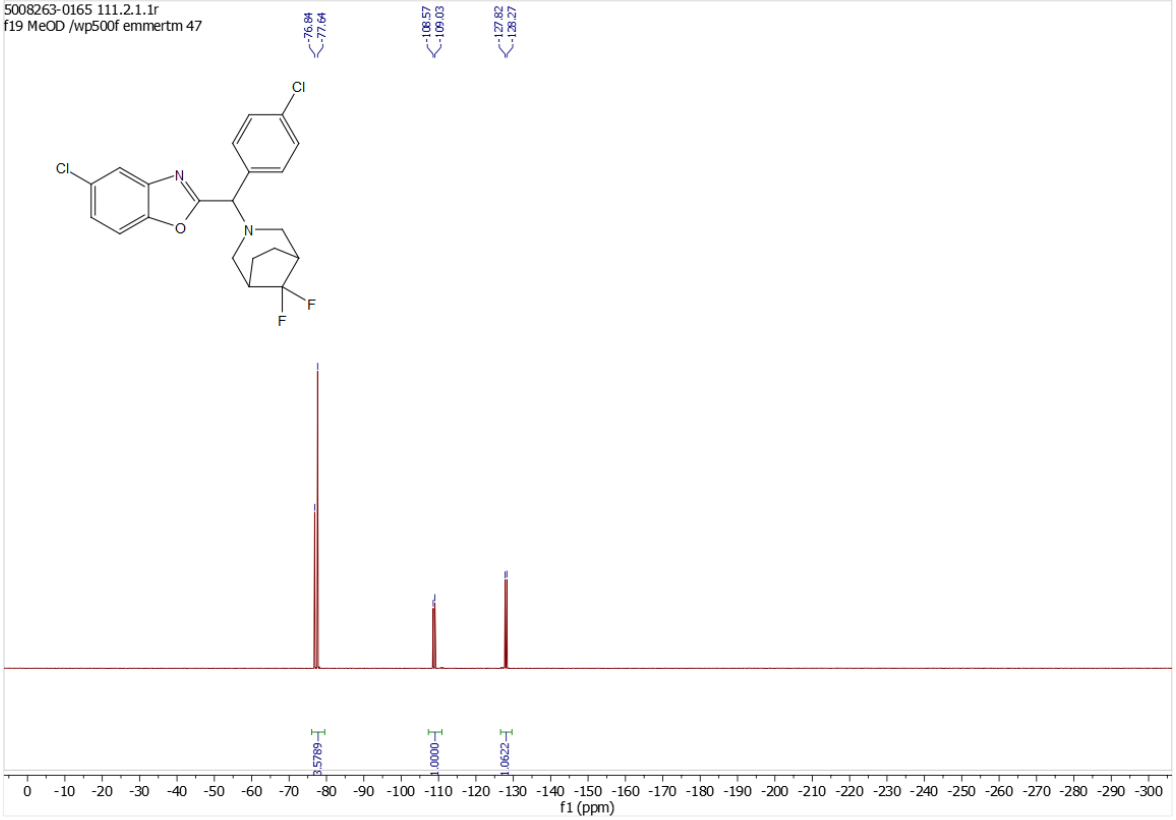
5008263-0165 111.2.1.1r
f19 MeOD /wp500f emmertm 47
-76.84
-77.64
-108.57
-109.03
-127.82
-128.27
Cl
Cl
N
O
N
F
F
3.5789
1.0000
1.0622
0 -10 -20 -30 -40 -50 -60 -70 -80 -90 -100 -110 -120 -130 -140 -150 -160 -170 -180 -190 -200 -210 -220 -230 -240 -250 -260 -270 -280 -290 -300
f1 (ppm)

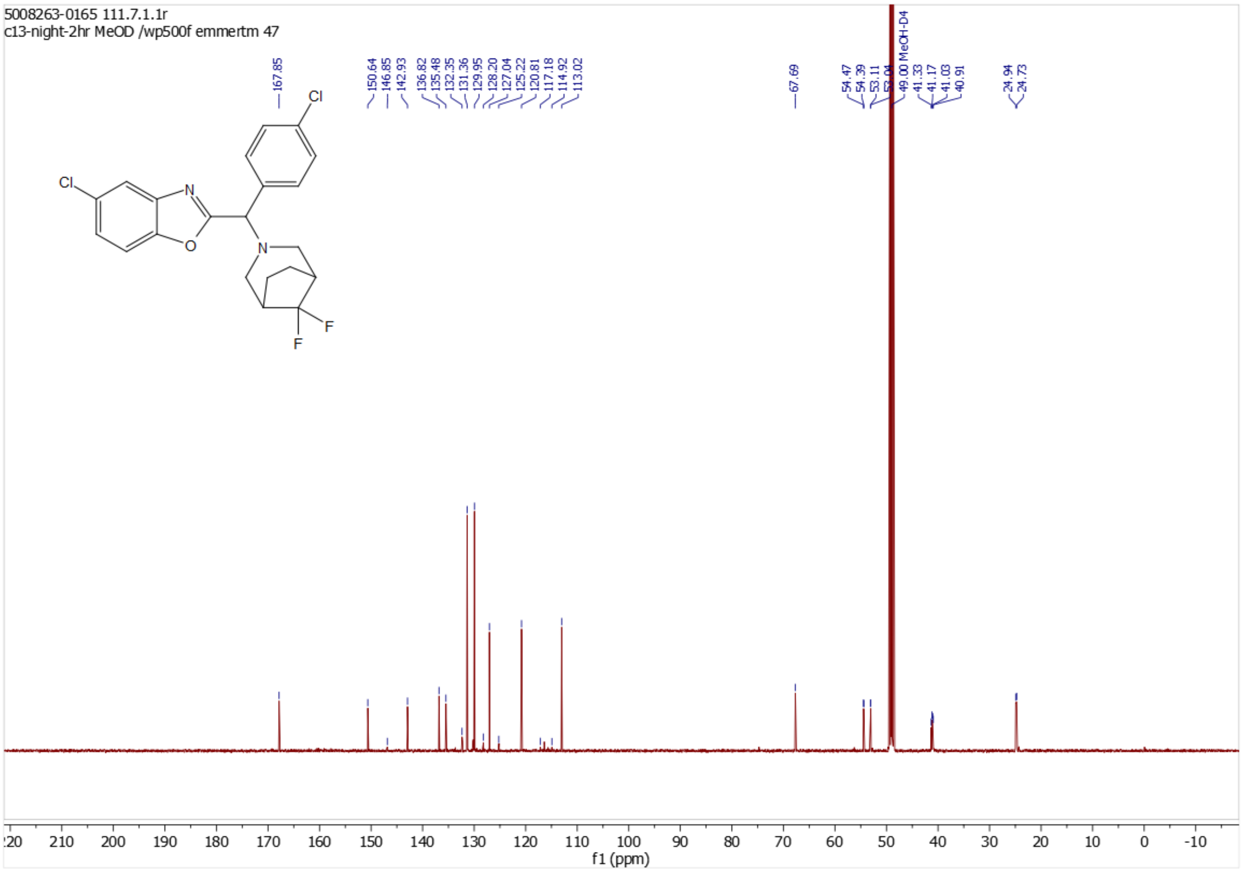
5008263-0165 111.7.1.1r
c13-night-2hr MeOD /wp500f emmertm 47
f1 (ppm)

5-Chloro-2-((4-chlorophenyl)(4,4-difluoro-5-methylazepan-1-yl)methyl)benzo[d]oxazole

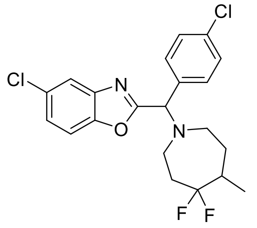

Chemical Formula: $C_{21}H_{20}Cl_2F_2N_2O$
Exact Mass: 424.0921
Molecular Weight: 425.3008

The title compound was prepared according to General Procedure 3 (PMC). Isolated as ~3:2 mixture of diastereomers, $C_{21}H_{20}Cl_2F_2N_2O(F_3CCO_2H)_{2.7}$ (apparent M_w 425.3008 + 114.0232*2.7 = 733.1634 g/mol).

Yield: 19.3 mg (15%); 32 LCAP before workup/column chromatography

^{1}H NMR (500 MHz, Methanol-d_4, ppm) δ 7.81 (t, J = 2.4 Hz, 1H), 7.62 (d, J = 8.7 Hz, 1H), 7.58 – 7.54 (m, 2H), 7.51 – 7.42 (m, 3H), 5.77 (s, 0.6H, major diastereomer), 5.76 (s, 0.4H, minor diastereomer), 3.29 – 3.05 (m, 4H), 2.31 (dddt, J = 41.2, 33.6, 22.5, 6.3 Hz, 3H), 1.82 (dddt, J = 18.0, 15.0, 11.3, 5.1 Hz, 2H), 1.11 (d, J = 7.0 Hz, 2H; Me, major diastereomer), 1.09 (d, J = 7.1 Hz, 1H; Me, minor diastereomer).

^{19}F NMR (470 MHz, Methanol-d_4, ppm) δ -77.22, -92.53 (d, J = 244.8 Hz), -92.67 (d, J = 244.6 Hz).

^{13}C NMR (126 MHz, Methanol-d_4, ppm) δ 165.4 (minor), 165.3 (major), 161.8 (1, J = 35.8 Hz; TFA), 150.8 (major), 150.7 (minor), 142.7, 136.9, 133.7 (minor), 133.6 (major), 132.0 (minor), 131.9 (major), 131.7 (major), 131.7 (minor), 130.5, 127.5, 128.2 – 124.2 (m; CF_2), 121.13, 113.2 (major), 113.2 (minor), 67.89 (minor), 67.7 (major), 53.1 (major), 52.6 (minor), 46.0 (t, J = 7.6 Hz; major), 45.9 (dd, J = 9.1, 6.2 Hz; minor), 41.3 (t, J = 24.4 Hz; major), 41.1 (t, J = 24.4 Hz; minor), 36.5 (t, J = 28.3; minor), 36.1 (t, J = 28.3; major), 28.5 (d, J = 8.0 Hz; minor), 28.3 (d, J = 8.8 Hz; major), 14.1 (dd, J = 6.2, 4.6 Hz; minor), 13.9 (dd, J = 6.4, 4.0 Hz; major).

HRMS: Calc. $C_{21}H_{21}Cl_2F_2N_2O^+$ [M+H$^+$] 425.0994; found 425.0995.

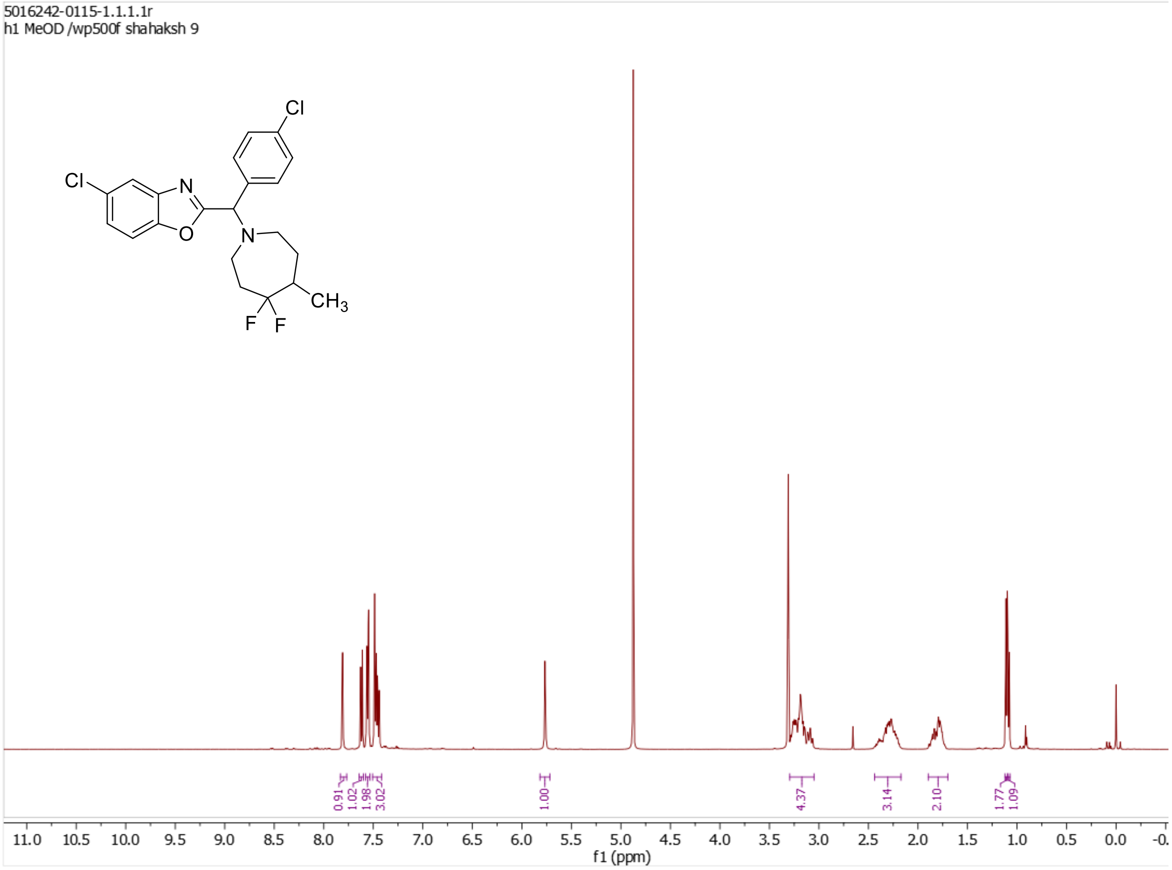
5016242-0115-1.1.1.1r
h1 MeOD /wp500f shahaksh 9
Cl
Cl
N
O
N
CH3
F F
0.91
1.02
1.98
3.02
1.00
4.37
3.14
2.10
1.77
1.09
f1 (ppm)

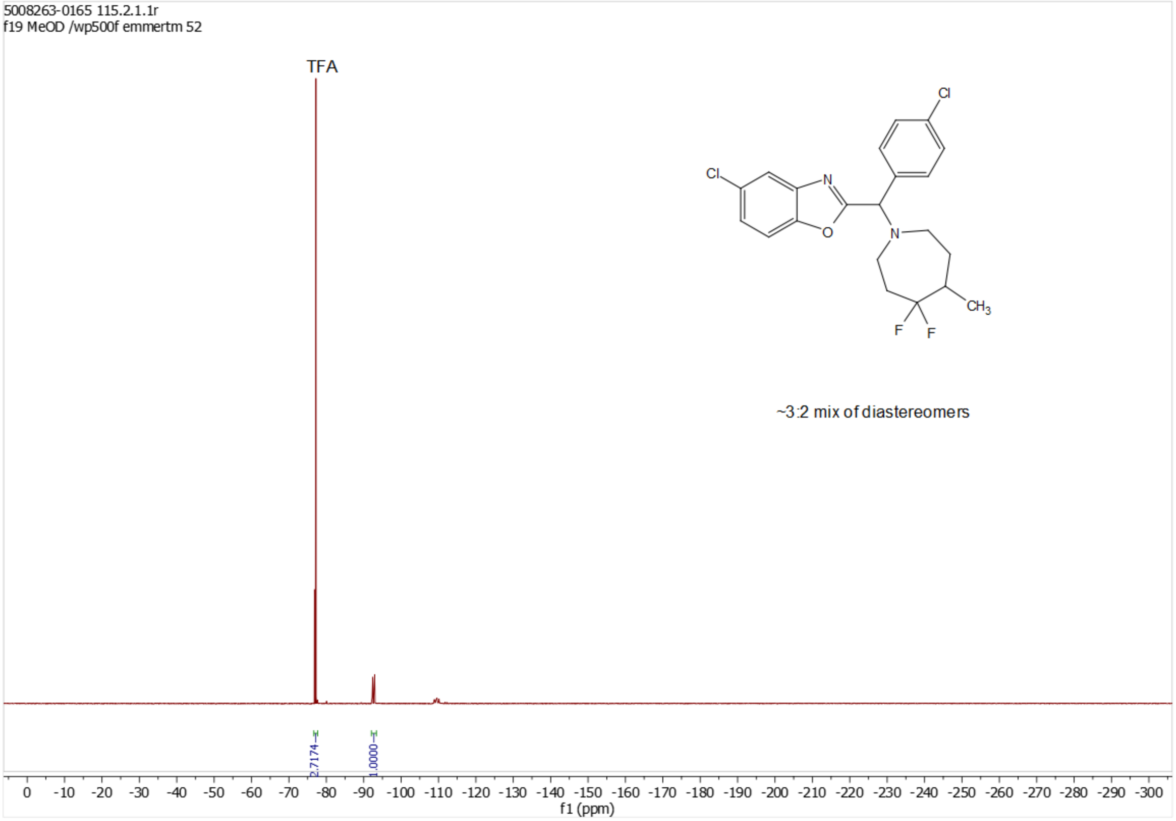
5008263-0165 115.2.1.1r
f19 MeOD /wp500f emmertm 52
TFA
Cl
Cl
N
O
N
CH3
F F
~3:2 mix of diastereomers
2.7174
1.0000
f1 (ppm)

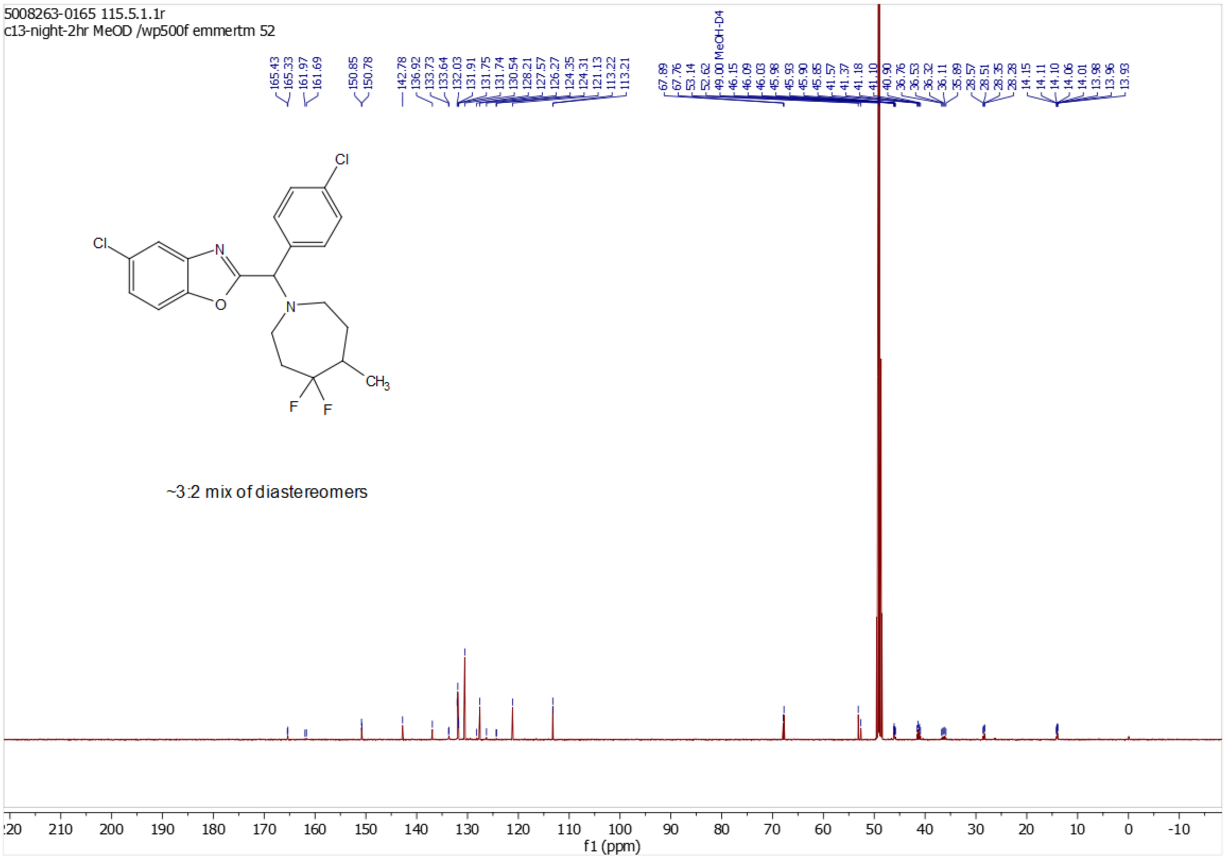
5008263-0165 115.5.1.1r
c13-night-2hr MeOD /wp500f emmertm 52
165.43 165.33 161.97 161.69 150.85 150.78 142.78 136.92 133.73 133.64 132.03 131.91 131.75 131.74 130.54 128.21 127.57 126.27 124.35 124.31 121.13 113.22 113.21
67.89 67.76 53.14 52.62 49.00 MeOH-D4 46.15 46.09 46.03 45.98 45.93 45.90 45.85 41.57 41.37 41.18 41.10 40.90 36.76 36.53 36.32 36.11 35.89 28.57 28.51 28.35 28.28 14.15 14.11 14.10 14.06 14.01 13.98 13.96 13.93
Cl
Cl
N
O
N
CH3
F
F
~3:2 mix of diastereomers
f1 (ppm)

Azole Scope

N-((5-Chlorobenzo[d]oxazol-2-yl)(phenyl)methyl)-*N*-ethylethanamine

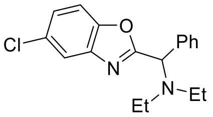

Chemical Formula: $C_{18}H_{19}ClN_2O$
Exact Mass: 314.12
Molecular Weight: 314.81

The title compound was prepared according to General Procedure 2 (reaction time 18 h).

Yield: 224 mg (71%); 75 LCAP before workup/column chromatography

^{1}H NMR (500 MHz, Methanol-d_4, ppm) δ 7.69 (s, 1H), 7.56 (d, *J* = 8.6 Hz, 1H), 7.50 (d, *J* = 7.4 Hz, 2H), 7.42 – 7.31 (m, 3H), 7.31 – 7.25 (m, 1H), 5.26 (s, 1H), 2.69 (dq, *J* = 13.5, 6.9 Hz, 2H), 2.58 (dq, *J* = 13.5, 6.6 Hz, 2H), 1.03 (t, *J* = 6.9 Hz, 6H).

^{13}C NMR (126 MHz, Methanol-d_4, ppm) δ 169.1, 150.6, 142.8, 138.7, 131.2, 129.6, 129.6, 129.3, 126.8, 120.5, 112.9, 65.7, 44.8, 12.0.

HRMS: Calc. $C_{18}H_{20}ClN_2O^+$ [M+H$^+$] 315.1259; found 315.1258.

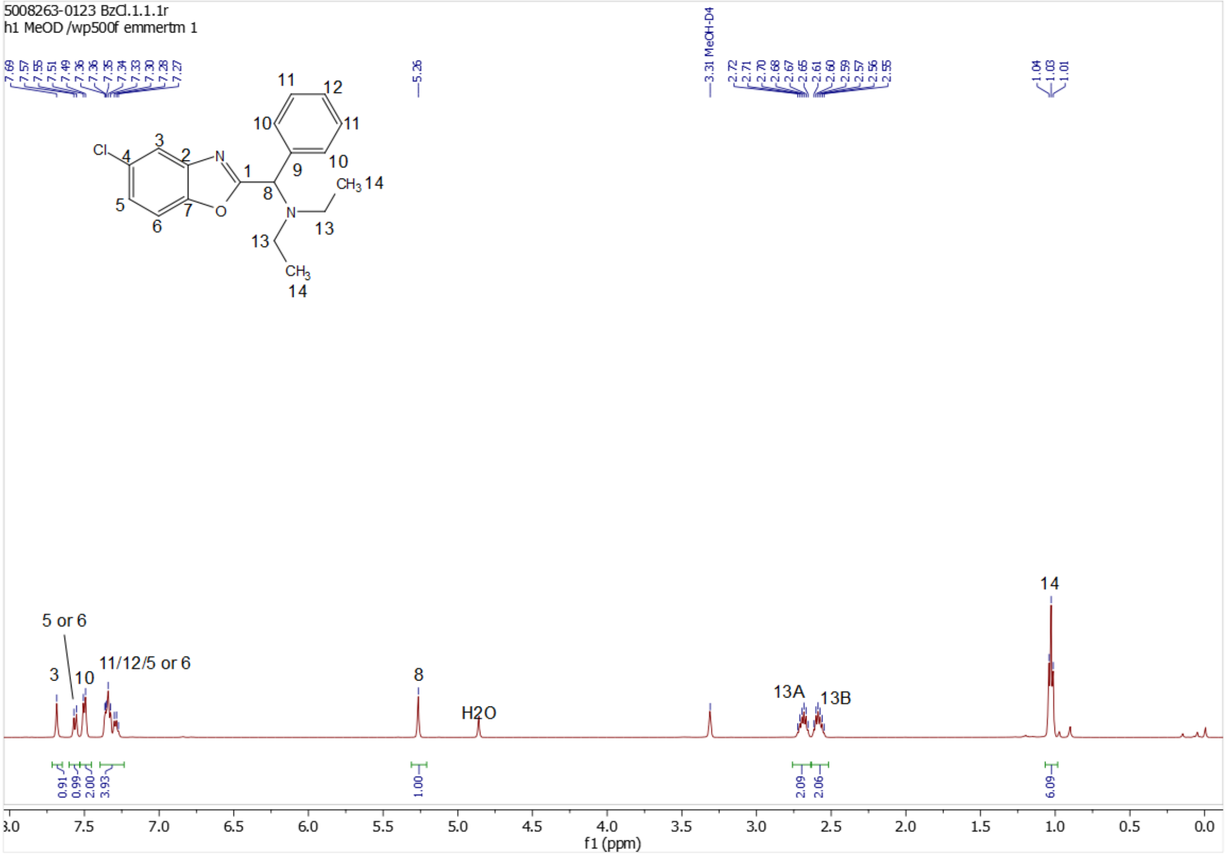

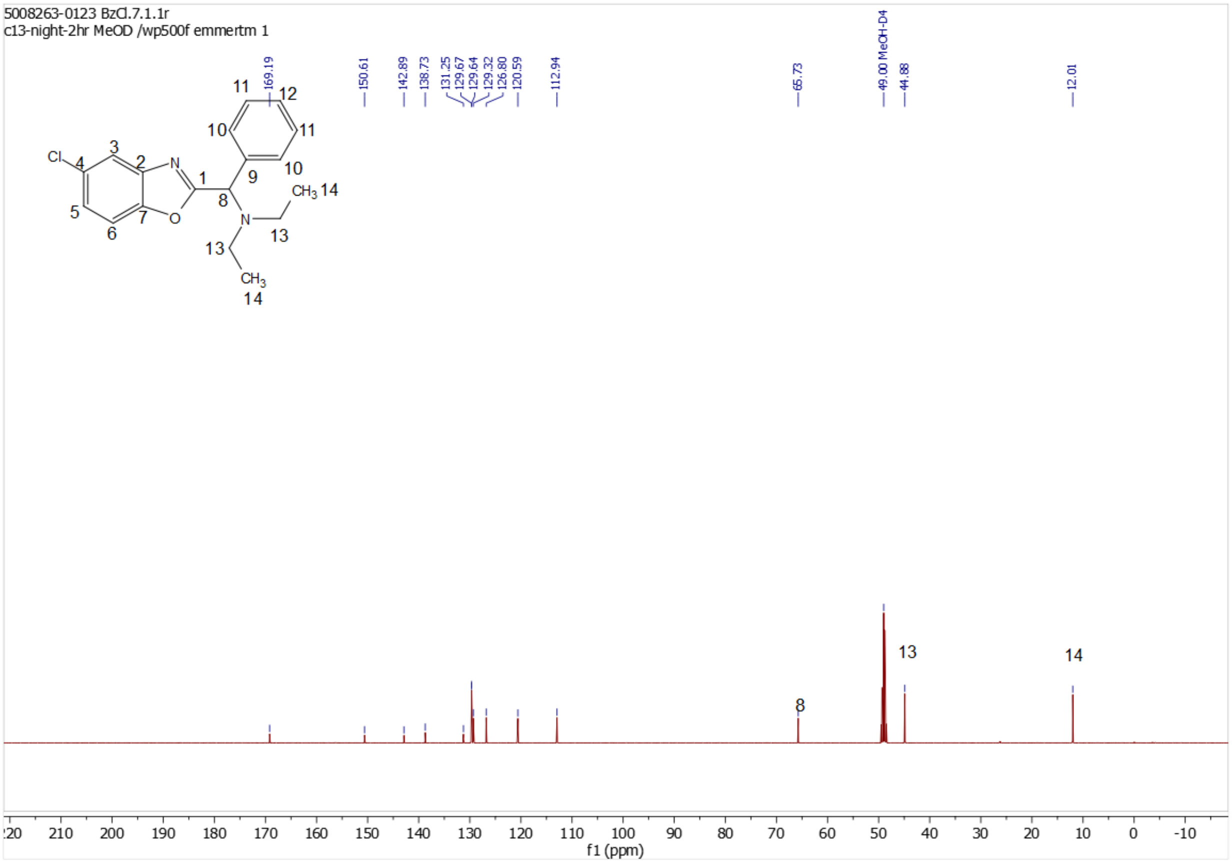
5008263-0123 BzCl.7.1.1r
c13-night-2hr MeOD /wp500f emmertm 1
169.19
150.61
142.89
138.73
131.25
129.67
129.64
129.32
126.80
120.59
112.94
65.73
49.00 MeOH-D4
44.88
12.01
8
13
14
f1 (ppm)

N-Ethyl-*N*-((4-fluorobenzo[d]oxazol-2-yl)(phenyl)methyl)ethanamine

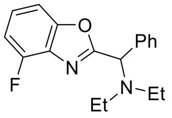

Chemical Formula: $C_{18}H_{19}FN_2O$
Exact Mass: 298.15
Molecular Weight: 298.36

The title compound was prepared according to General Procedure 2 (reaction time 18 h).

Yield: 188 mg (63%); 67 LCAP before workup/column chromatography

^{1}H NMR (500 MHz, Methanol-d_4, ppm) δ 7.51 (s, 2H), 7.44 (d, J = 8.0 Hz, 1H), 7.41 – 7.33 (m, 3H), 7.33 – 7.27 (m, 1H), 7.12 (t, J = 9.0 Hz, 1H), 5.28 (s, 1H), 2.71 (dq, J = 13.5, 6.9 Hz, 2H), 2.60 (dq, J = 13.5, 6.6 Hz, 2H), 1.04 (t, J = 6.9 Hz, 6H).

^{19}F NMR (470 MHz, Methanol-d_4, ppm) δ -127.98.

^{13}C NMR (126 MHz, Methanol-d_4, ppm) δ 167.9, 154.2 (d, J = 7.0 Hz), 153.5, 138.7, 130.1 (d, J = 16.9 Hz), 129.6, 129.6, 129.3, 127.2 (d, J = 7.2 Hz), 111.8 (d, J = 17.8 Hz), 108.2 (d, J = 4.5 Hz), 65.7, 44.8, 11.9.

HRMS: Calc. $C_{18}H_{20}FN_2O^+$ [M+H$^+$] 299.1554; found 299.1566.

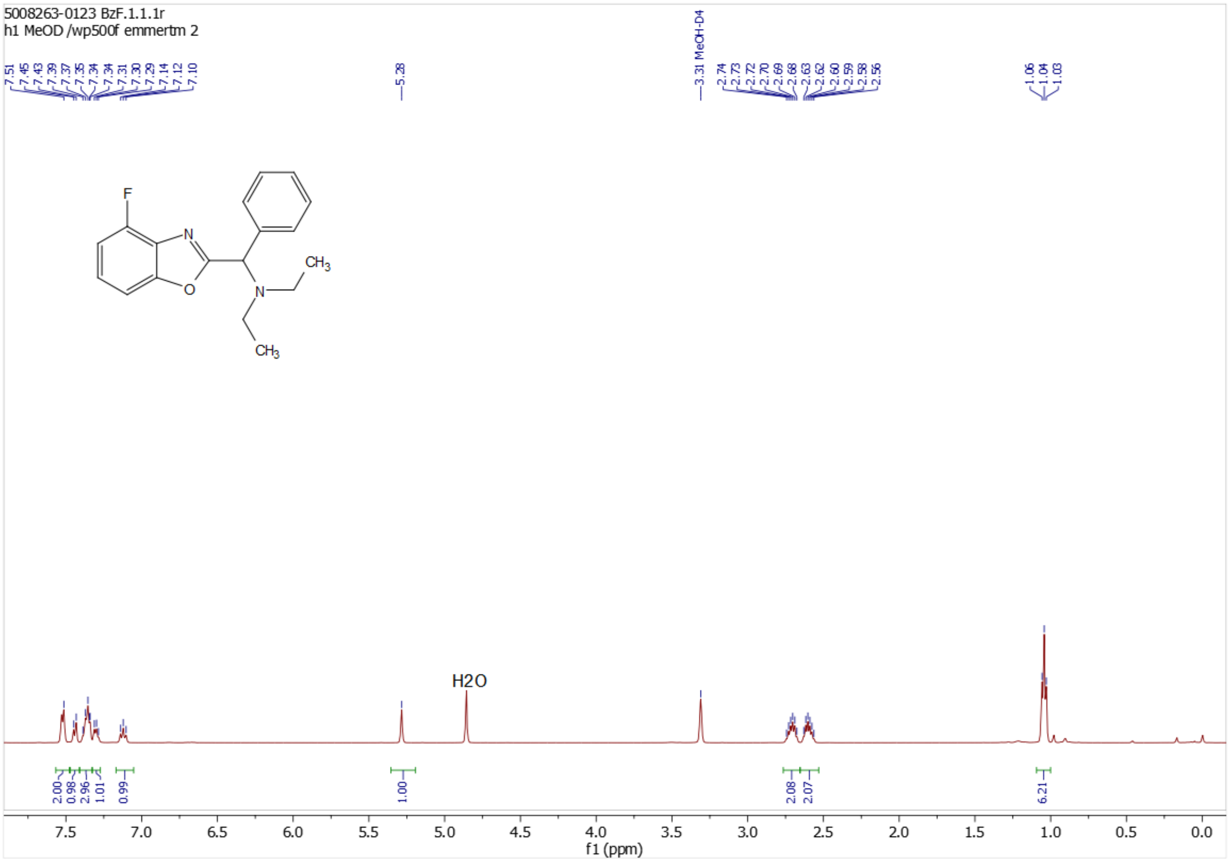

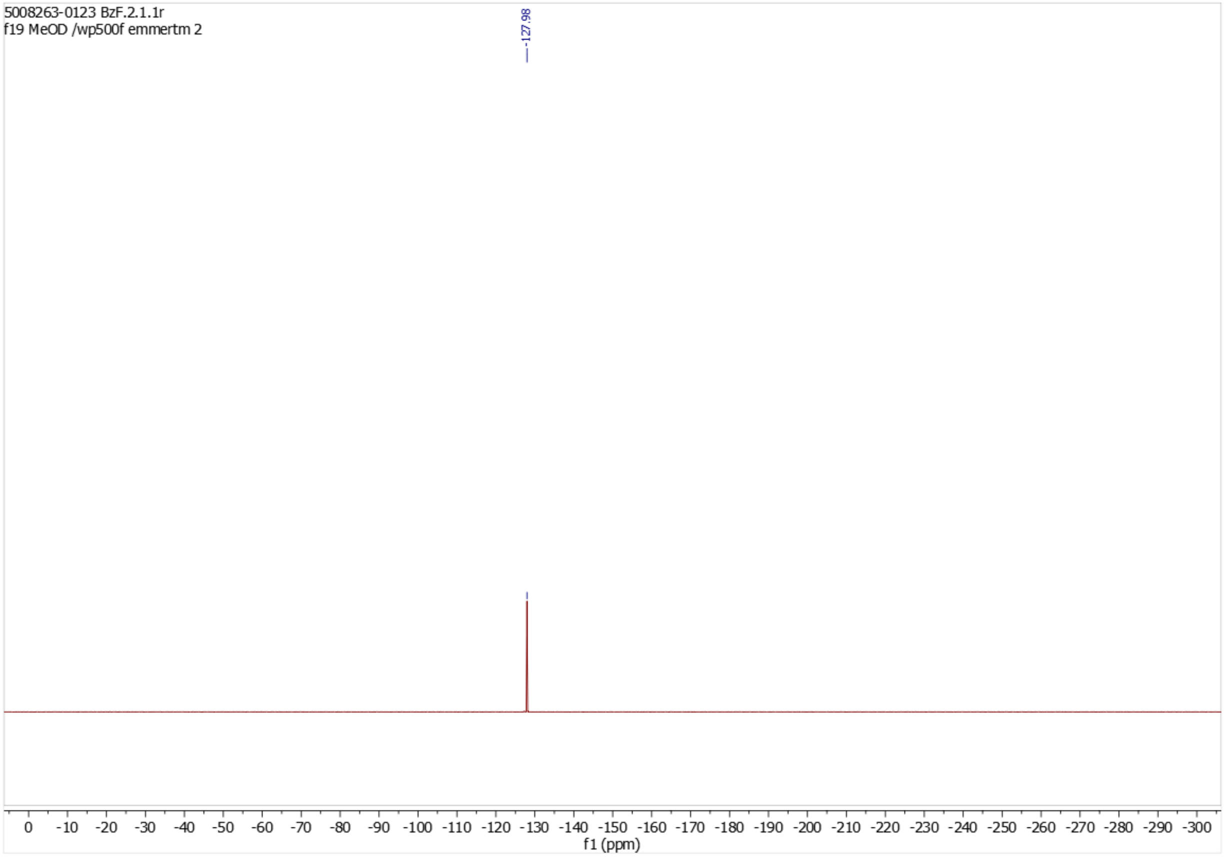

5008263-0123 BzF.2.1.1r
f19 MeOD /wp500f emmertm 2
-127.98
0 -10 -20 -30 -40 -50 -60 -70 -80 -90 -100 -110 -120 -130 -140 -150 -160 -170 -180 -190 -200 -210 -220 -230 -240 -250 -260 -270 -280 -290 -300
f1 (ppm)

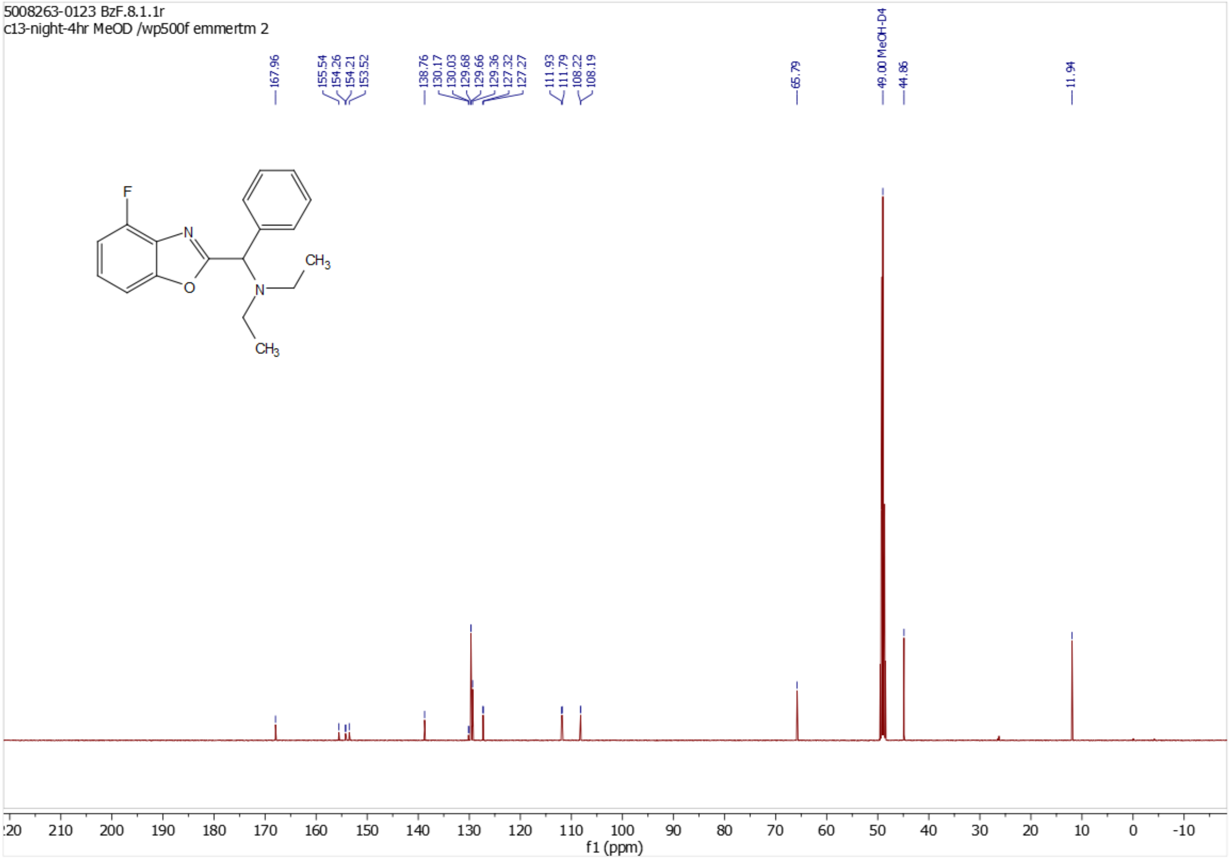

5008263-0123 BzF.8.1.1r
c13-night-4hr MeOD /wp500f emmertm 2
167.96
155.54
154.26
154.21
153.52
138.76
130.17
130.03
129.68
129.66
129.36
127.32
127.27
111.93
111.79
108.22
108.19
65.79
49.00 MeOH-D4
44.86
11.94
F
N
O
N
CH3
CH3
f1 (ppm)

N-Ethyl-N-((5-nitrobenzo[d]oxazol-2-yl)(phenyl)methyl)ethanamine

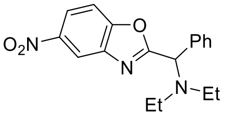

Chemical Formula: $C_{18}H_{19}N_3O_3$
Exact Mass: 325.14
Molecular Weight: 325.37

The title compound was prepared according to General Procedure 2 (reaction time 18 h).

Yield: 224 mg (69%); 61 LCAP before workup/column chromatography

^{1}H NMR (500 MHz, Methanol-d_4, ppm) δ 8.57 (s, 1H), 8.34 (d, J = 8.7 Hz, 1H), 7.88 (d, J = 8.6 Hz, 1H), 7.53 (d, J = 7.2 Hz, 2H), 7.42 – 7.28 (m, 3H), 5.37 (s, 1H), 2.73 (dq, J = 13.6, 6.8 Hz, 2H), 2.63 (dq, J = 13.6, 6.8 Hz, 2H), 1.06 (t, J = 6.8 Hz, 6H).

^{13}C NMR (126 MHz, Methanol-d_4, ppm) δ 172.4, 151.1, 147.0, 146.9, 138.3, 129.7, 129.7, 129.4, 121.7, 121.0, 108.5, 65.8, 44.9, 12.1.

HRMS: Calc. $C_{18}H_{20}N_3O_3^+$ [M+H$^+$] 326.1499; found 326.1516.

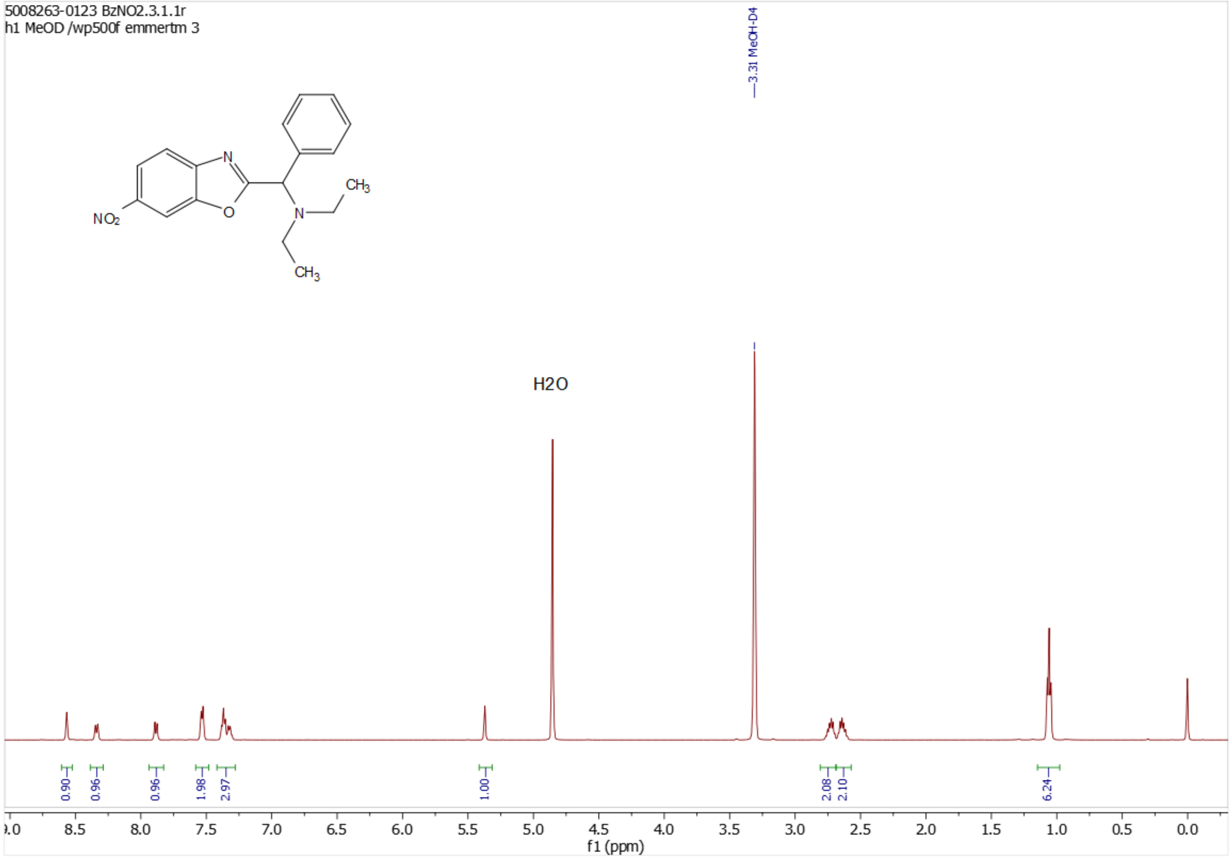

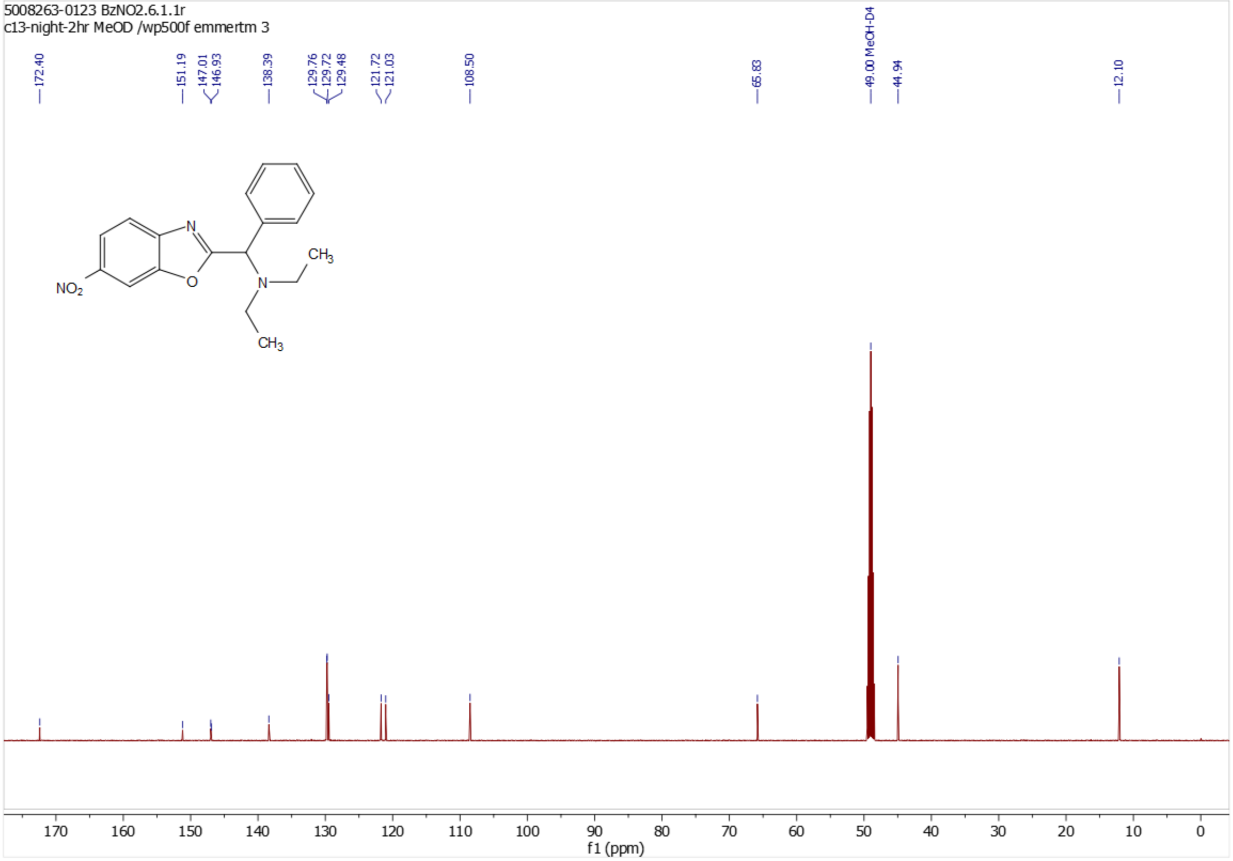

5008263-0123 BzNO2.6.1.1r
c13-night-2hr MeOD /wp500f emmertm 3
172.40
151.19
147.01
146.93
138.39
129.76
129.72
129.48
121.72
121.03
108.50
65.83
49.00 MeOH-D4
44.94
12.10
NO2
N
O
CH3
f1 (ppm)
170
160
150
140
130
120
110
100
90
80
70
60
50
40
30
20
10
0

N-ethyl-*N*-((5-(4-fluorophenyl)oxazol-2-yl)(phenyl)methyl)ethanamine

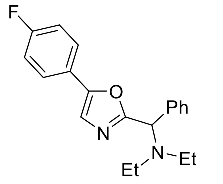

Chemical Formula: $C_{20}H_{21}FN_2O$
Exact Mass: 324.16
Molecular Weight: 324.40

Yield: 175 mg (54%); 89 LCAP before workup/column chromatography

The title compound was prepared according to General Procedure 2 (reaction time 4 h).

^{1}H NMR (500 MHz, Methanol-d_4, ppm) δ 7.73 – 7.64 (m, 2H), 7.50 (d, J = 7.4 Hz, 2H), 7.34 (t, J = 7.1 Hz, 2H), 7.28 (t, J = 7.0 Hz, 1H), 7.20 – 7.12 (m, 2H), 5.15 (s, 1H), 2.69 (dq, J = 13.5, 6.8 Hz, 2H), 2.55 (dq, J = 13.1, 6.5 Hz, 2H), 1.03 (t, J = 6.9 Hz, 6H).

^{19}F NMR (470 MHz, Methanol-d_4, ppm) δ -114.18.

^{13}C NMR (126 MHz, Methanol-d_4, ppm) δ 165.1, 164.6, 164.2 (d, J = 247.7 Hz), 152.5, 129.6, 129.5, 129.2, 127.4 (d, J = 8.4 Hz), 122.0 (d, J = 1.2 Hz), 117.1, 116.9, 65.2, 44.9, 11.9.

HRMS: Calc. $C_{20}H_{22}FN_2O^+$ [M+H$^+$] 325.1711; found 325.1715.

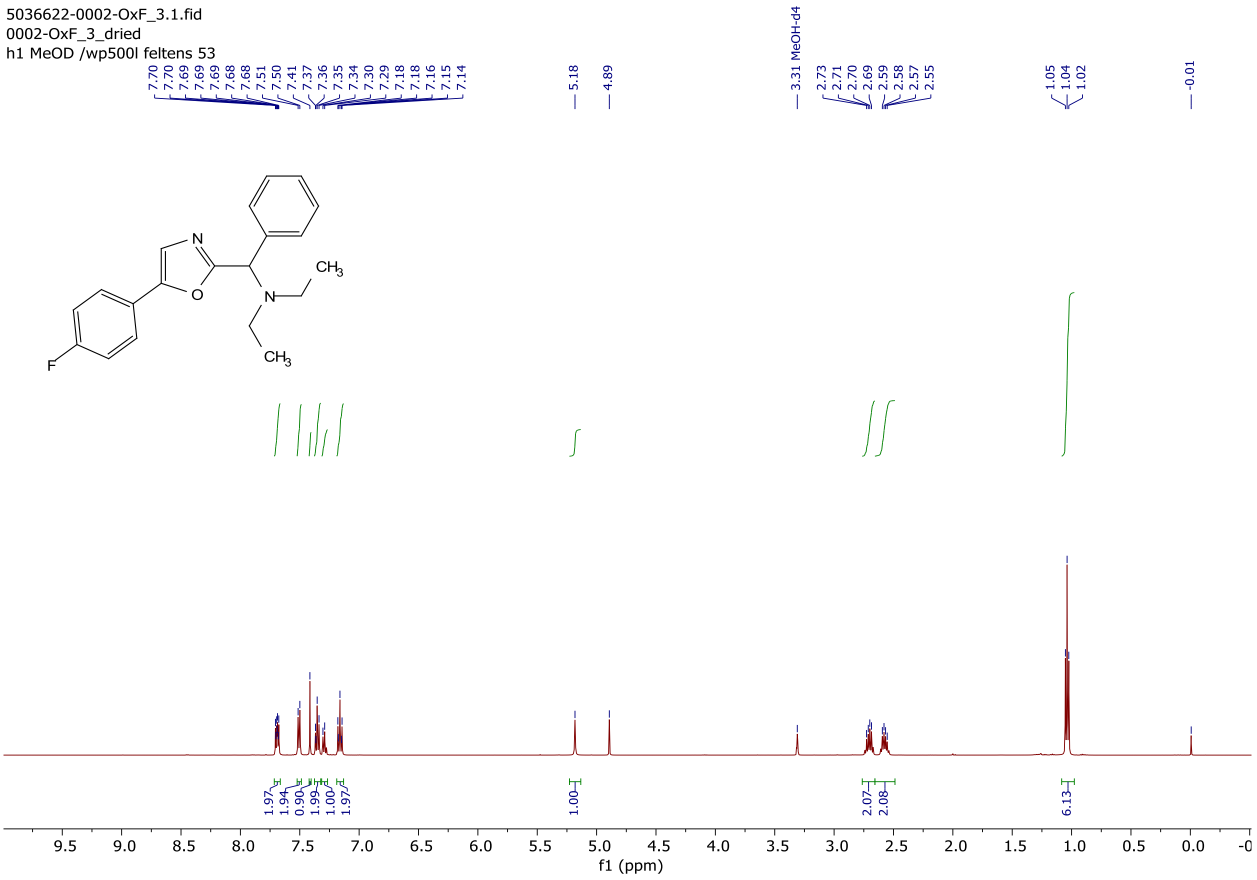

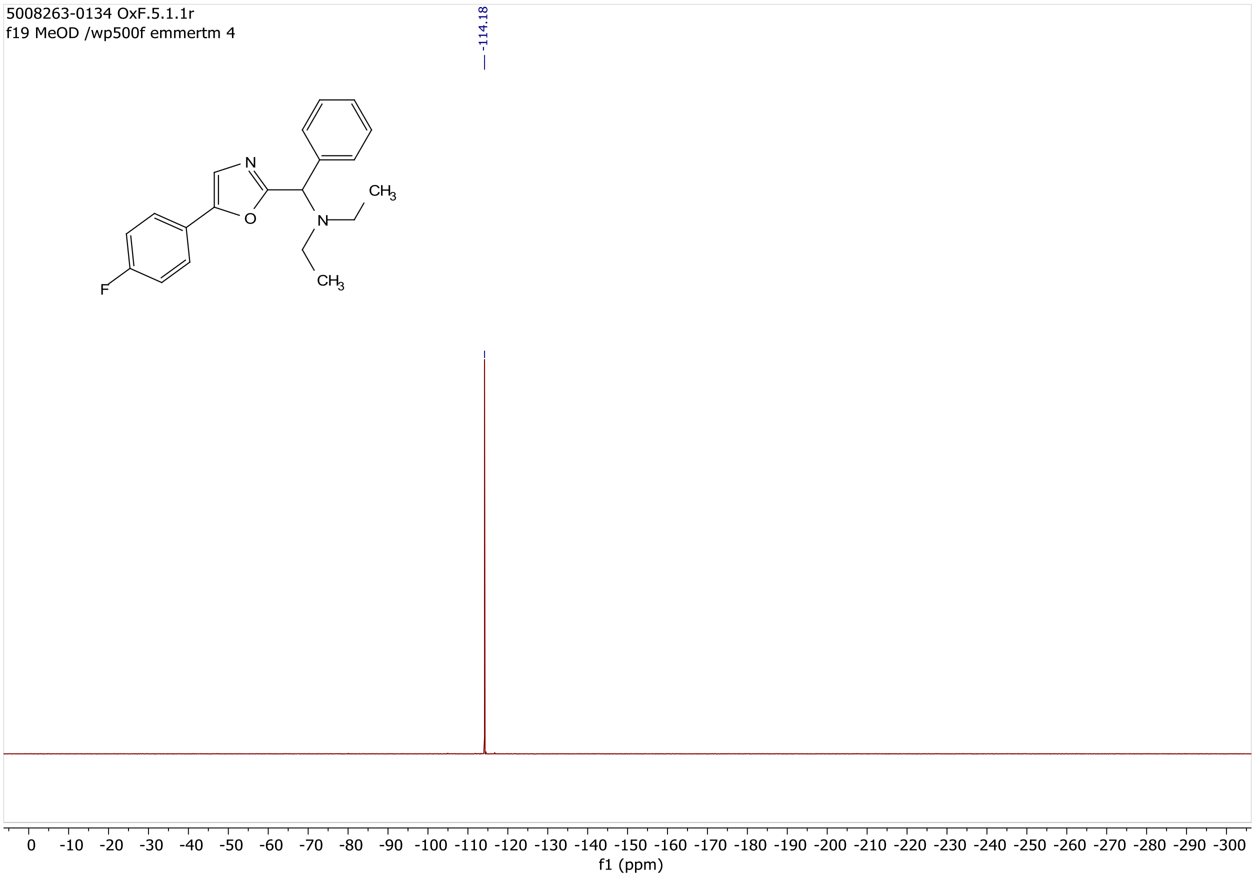
5008263-0134 OxF.5.1.1r
f19 MeOD /wp500f emmertm 4
-114.18
N
O
CH3
N
CH3
F
0 -10 -20 -30 -40 -50 -60 -70 -80 -90 -100 -110 -120 -130 -140 -150 -160 -170 -180 -190 -200 -210 -220 -230 -240 -250 -260 -270 -280 -290 -300
f1 (ppm)

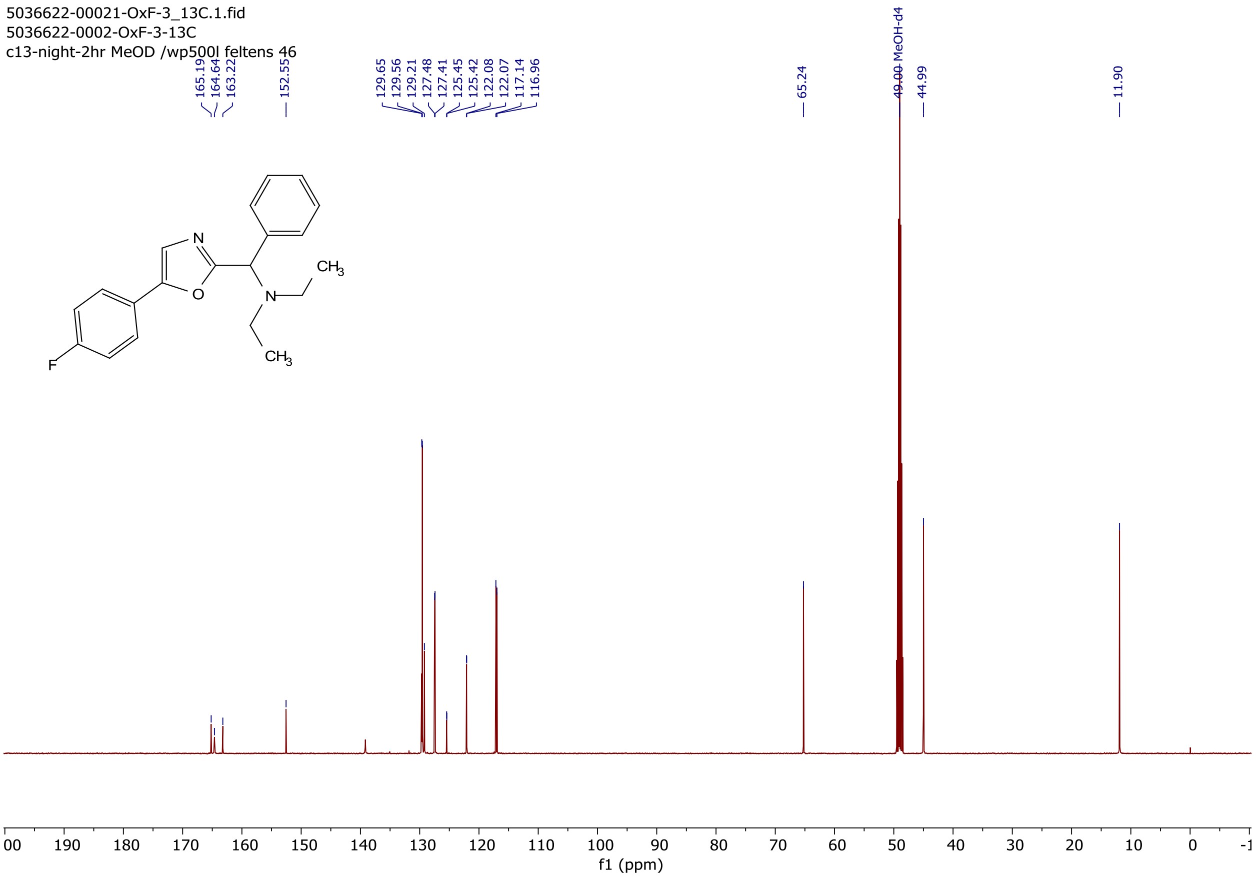
5036622-00021-OxF-3_13C.1.fid
5036622-0002-OxF-3-13C
c13-night-2hr MeOD /wp500l feltens 46
165.19
164.64
163.22
152.55
129.65
129.56
129.21
127.48
127.41
125.45
125.42
122.08
122.07
117.14
116.96
65.24
49.00 MeOH-d4
44.99
11.90
N
O
F
CH3
CH3
f1 (ppm)

N-((1H-imidazol-2-yl)(phenyl)methyl)-*N*-ethylethanamine

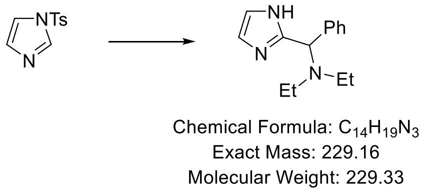

The title compound was prepared according to General Procedure 2 (reaction time 4 h), starting from N-tosylimidazole. After hydrolysis of the tosyl protecting group and silica gel column chromatography, the obtained yellow residue was dissolved in 5 mL dichloromethane and extracted with 3 x 5 mL 1M HCl. 25 mL 1 M NaOH was added to the combined extracts, resulting in precipitation of a crystalline solid, which was collected by filtration.

Yield: 202 mg (88%); 24 LCAP imidazole-H product (59 LCAP imidazole-tosyl intermediate before hydrolysis)

^{1}H NMR (500 MHz, Methanol-*d*$_4$, ppm) δ 7.46 (d, *J* = 7.2 Hz, 2H), 7.30 (t, *J* = 7.0 Hz, 2H), 7.23 (t, *J* = 7.1 Hz, 1H), 6.93 (broad s, 2H), 4.94 (s, 1H), 2.54 (m, 4H), 0.99 (t, *J* = 6.7 Hz, 6H).

^{13}C NMR (126 MHz, Methanol-*d*$_4$, ppm) δ 150.2, 141.6, 129.4, 129.3, 128.4, 66.4, 44.3, 11.4.

HRMS: Calc. $C_{14}H_{20}N_3^+$ [M+H$^+$] 230.1652; found 230.1662.

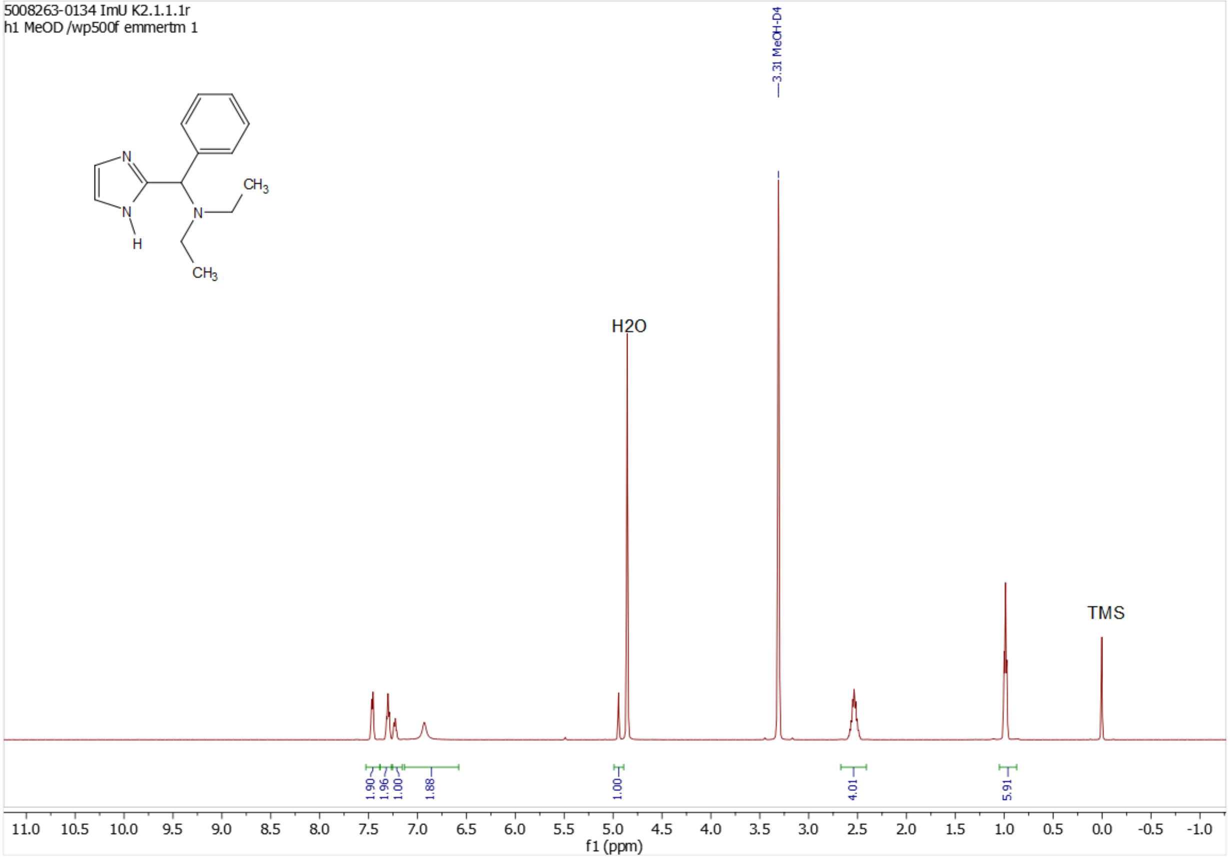

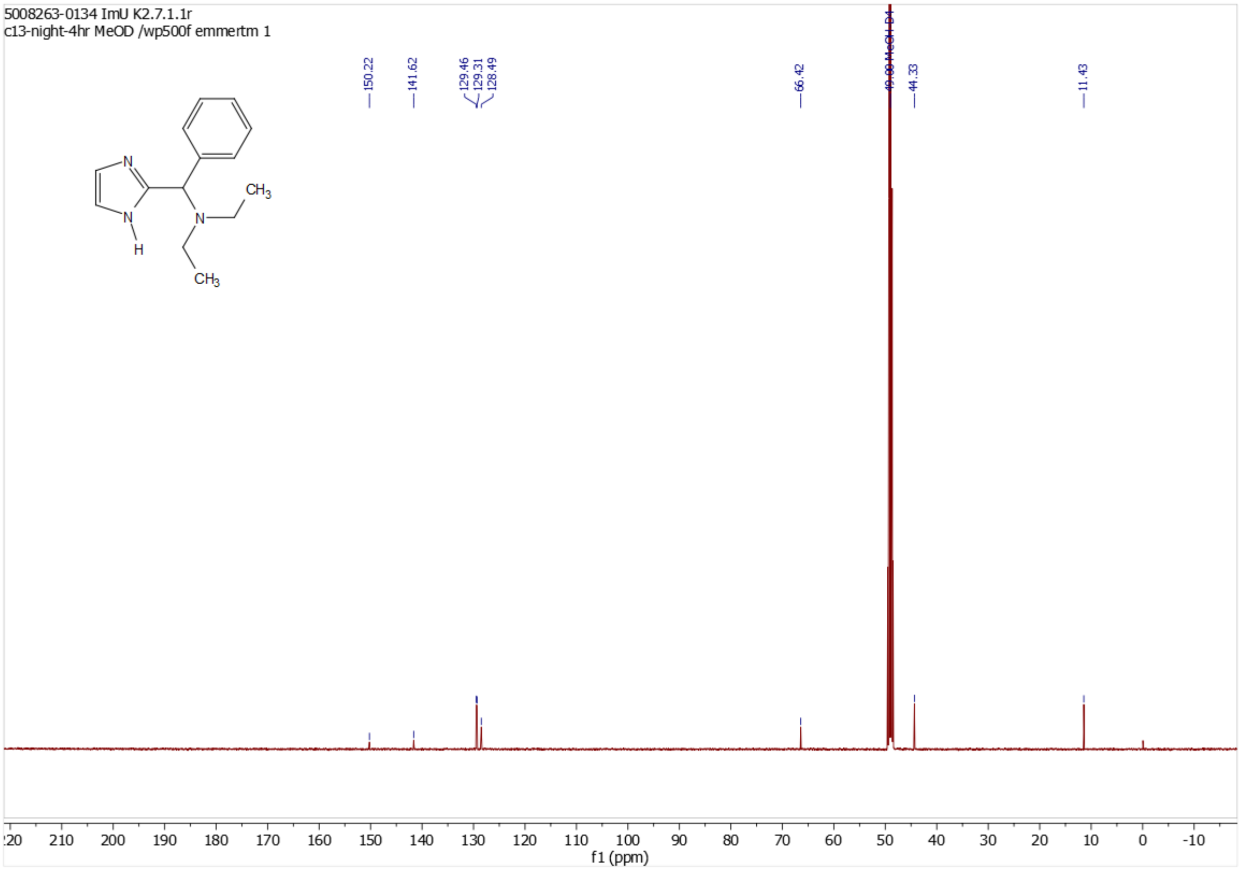
5008263-0134 ImU K2.7.1.1r
c13-night-4hr MeOD /wp500f emmertm 1
150.22
141.62
129.46
129.31
128.49
66.42
49.00 MeOH-D4
44.33
11.43
N
N
H
N
CH3
CH3
220
210
200
190
180
170
160
150
140
130
120
110
100
90
80
70
60
50
40
30
20
10
0
-10
f1 (ppm)

2-((diethylamino)(phenyl)methyl)-*N*,*N*-diphenyl-1H-imidazole-1-carboxamide

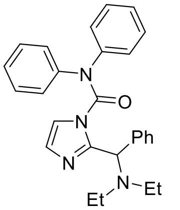

Chemical Formula: $C_{27}H_{28}N_4O$
Exact Mass: 424.23
Molecular Weight: 424.55

The title compound was prepared according to General Procedure 2 (reaction time 4 h).

Yield: 14.7 mg (3%); 41 LCAP before workup/column chromatography

^{1}H NMR (500 MHz, Methanol-d_4, ppm) δ 7.45 – 7.35 (m, 6H), 7.23 (m, 6H), 6.94 (s, 1H), 6.83 (broad s, 3H), 6.70 (s, 1H), 5.59 (s, 1H), 2.77 (dq, J = 12.9, 6.4 Hz, 2H), 2.65 (dq, J = 13.3, 6.7 Hz, 2H), 1.01 (t, J = 6.8 Hz, 7H).

^{13}C NMR (126 MHz, Methanol-d_4, ppm) δ 152.5, 151.3, 143.5, 140.0, 130.9, 130.5, 129.6, 129.2, 128.4, 127.8, 127.6, 120.9, 63.5, 44.5, 11.6.

HRMS: Calc. $C_{27}H_{29}N_4O^+$ [M+H$^+$] 425.2336; found 425.2323.

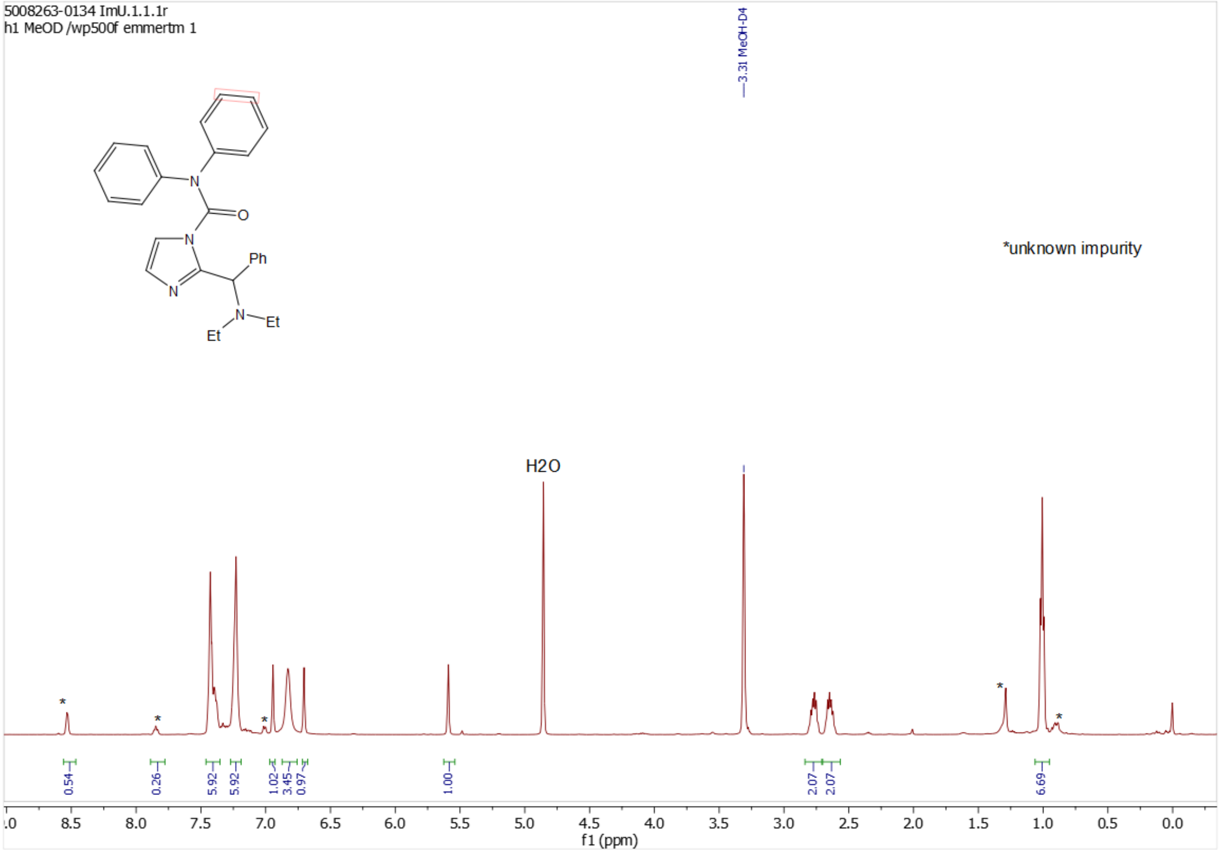
5008263-0134 ImU.1.1.1r
h1 MeOD /wp500f emmertm 1
3.31 MeOH-D4
*unknown impurity
H2O
0.54
0.26
5.92
5.92
1.02
3.45
0.97
1.00
2.07
2.07
6.69
f1 (ppm)

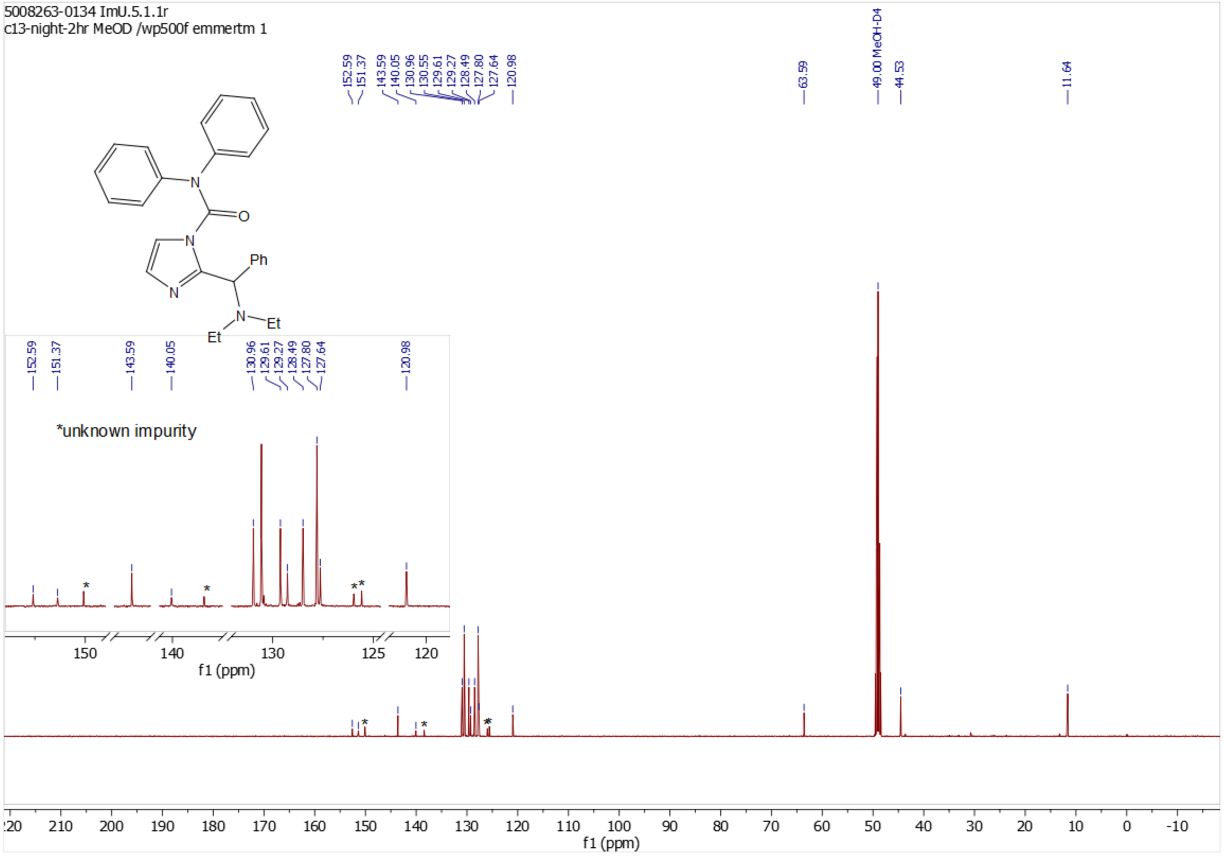
5008263-0134 ImU.5.1.1r
c13-night-2hr MeOD /wp500f emmertm 1
152.59
151.37
143.59
140.05
130.96
130.55
129.61
129.27
128.49
127.80
127.64
120.98
63.59
49.00 MeOH-D4
44.53
11.64
Ph
N
O
Et
*unknown impurity
f1 (ppm)

N-ethyl-*N*-(phenyl(5-((trimethylsilyl)ethynyl)thiazol-2-yl)methyl)ethanamine

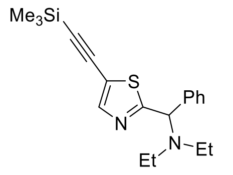

Chemical Formula: $C_{19}H_{26}N_2SSi$
Exact Mass: 342.16
Molecular Weight: 342.58

The title compound was prepared according to General Procedure 2 (reaction time 4 h).

Yield: 170 mg (50%); 74 LCAP before workup/column chromatography

1H NMR (500 MHz, Methanol-d_4, ppm) δ 7.71 (s, 1H), 7.43 – 7.21 (m, 5H), 5.16 (s, 1H), 2.67 (dq, *J* = 13.3, 6.7 Hz, 2H), 2.48 (dq, *J* = 13.1, 6.5 Hz, 2H), 1.04 (t, *J* = 6.8 Hz, 6H), 0.23 (s, 9H).

^{13}C NMR (126 MHz, Methanol-d_4, ppm) δ 178.8, 147.3, 139.1, 130.3, 129.5, 129.0, 120.7, 103.3, 94.8, 69.6, 44.7, 12.1, -0.2.

HRMS: Calc. $C_{19}H_{27}N_2Si^+$ [M+H$^+$] 343.1659; found 343.1664.

5036622-0003_ThSi_2.1.fid
0003_ThSi_2_FR20
h1 MeOD /wp500l feltens 12

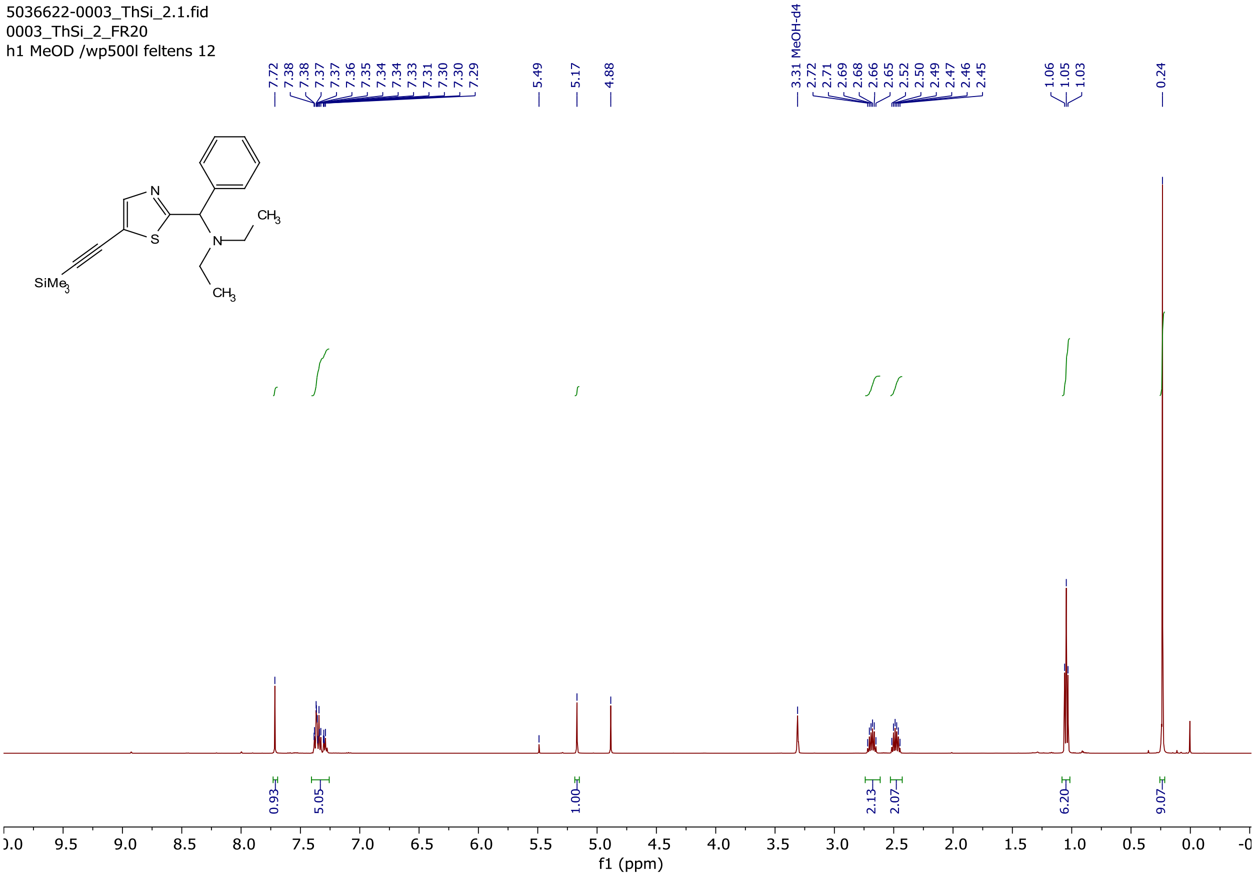

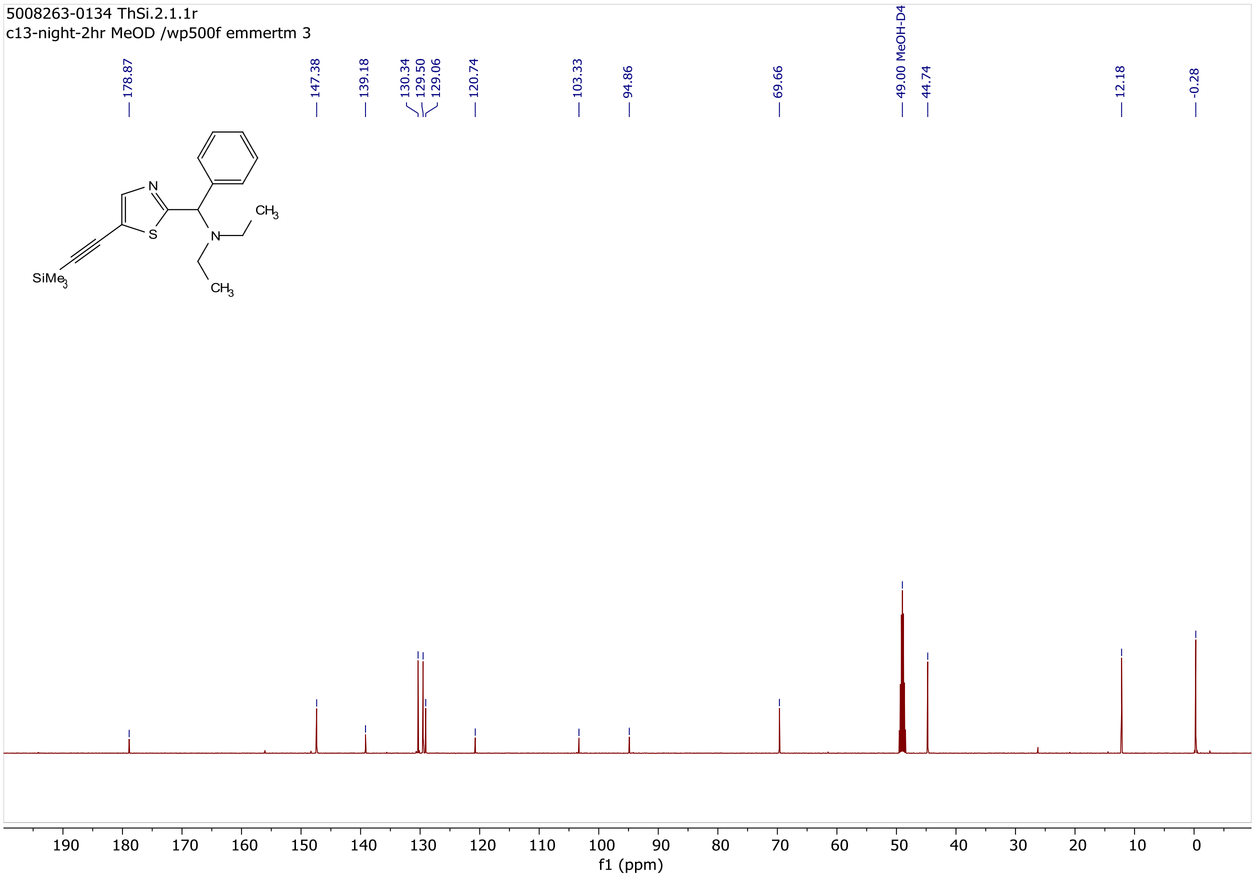
5008263-0134 ThSi.2.1.1r
c13-night-2hr MeOD /wp500f emmertm 3
178.87
147.38
139.18
130.34
129.50
129.06
120.74
103.33
94.86
69.66
49.00 MeOH-D4
44.74
12.18
-0.28
N
S
CH3
N
SiMe3
CH3
190 180 170 160 150 140 130 120 110 100 90 80 70 60 50 40 30 20 10 0
f1 (ppm)

N-((6-bromopyridin-2-yl)methyl)-2-((diethylamino)(phenyl)methyl)oxazole-5-carboxamide

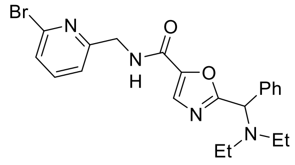

Chemical Formula: $C_{21}H_{23}BrN_4O_2$
Exact Mass: 442.10
Molecular Weight: 443.35

The title compound was prepared according to General Procedure 2 (reaction time 4 h).

Yield: 27.3 mg (6%); 29 LCAP before workup/column chromatography

The title compound was prepared according the modified general procedure 2, using 4.0 eq. TBSOTf and 2.0 eq. $^{i}Pr_2NEt$. The quench was performed with 8 eq. KF in pyridine.

Yield: 266 mg (60%); 50 LCAP before workup/column chromatography

^{1}H NMR (500 MHz, Methanol-d_4, ppm) δ 7.72 (s, 1H), 7.64 (t, *J* = 7.6 Hz, 1H), 7.47 (t, *J* = 9.9 Hz, 3H), 7.32 (dt, *J* = 27.7, 6.5 Hz, 4H), 5.19 (s, 1H), 4.60 (s, 2H), 2.66 (dq, *J* = 13.4, 6.7 Hz, 2H), 2.55 (dq, *J* = 13.1, 6.5 Hz, 2H), 1.02 (t, *J* = 6.8 Hz, 6H).

^{13}C NMR (126 MHz, Methanol-d_4, ppm) δ 167.2, 160.5, 159.2, 146.7, 142.4, 140.9, 138.7, 131.3, 129.6, 129.6, 129.2, 127.9, 121.7, 65.3, 44.8, 12.0.

HRMS: Calc. $C_{21}H_{24}BrN_4O_2^+$ [M+H$^+$] 443.1077; found 443.1062.

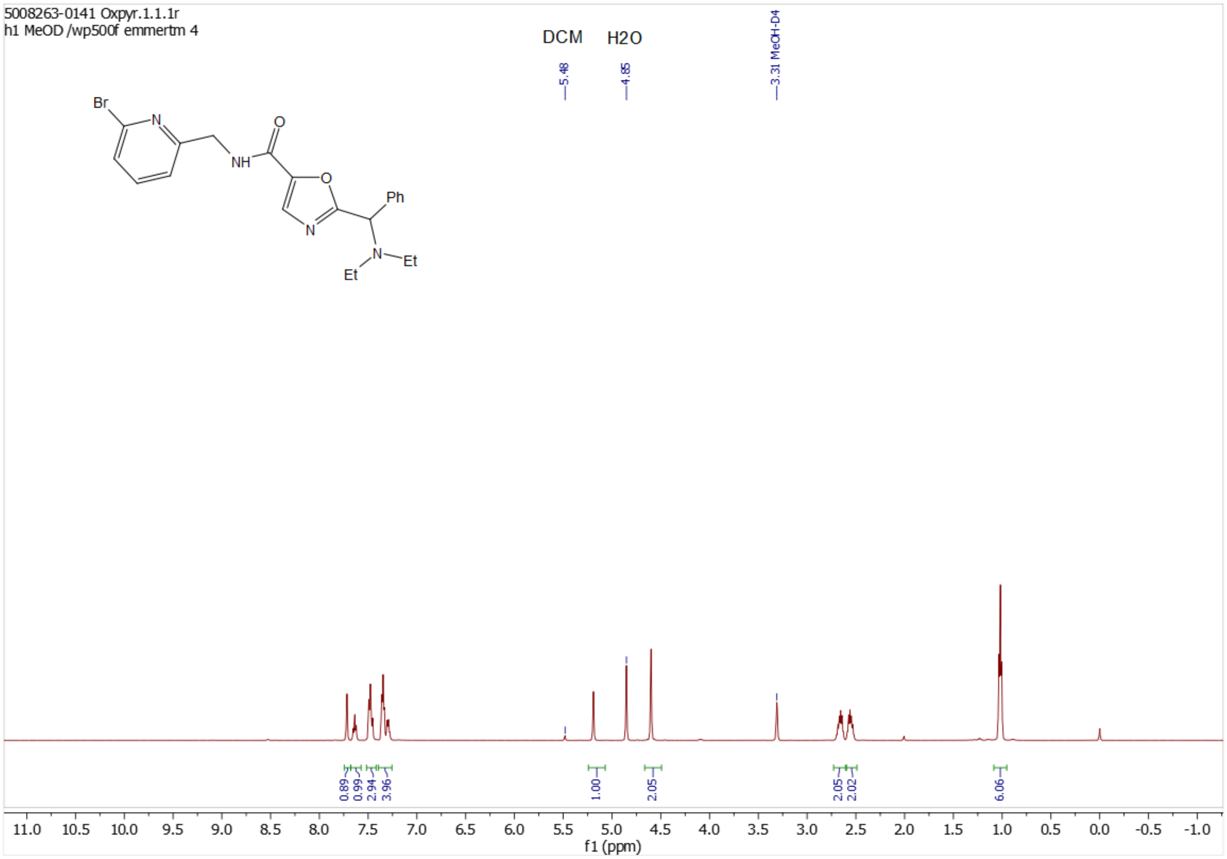

5008263-0141 Oxpyr.1.1.1r
h1 MeOD /wp500f emmertm 4
DCM
H2O
5.48
4.85
3.31 MeOH-D4
Br
N
NH
O
O
N
Ph
N
Et
Et
0.89
0.99
2.94
3.96
1.00
2.05
2.05
2.02
6.06
11.0 10.5 10.0 9.5 9.0 8.5 8.0 7.5 7.0 6.5 6.0 5.5 5.0 4.5 4.0 3.5 3.0 2.5 2.0 1.5 1.0 0.5 0.0 -0.5 -1.0
f1 (ppm)

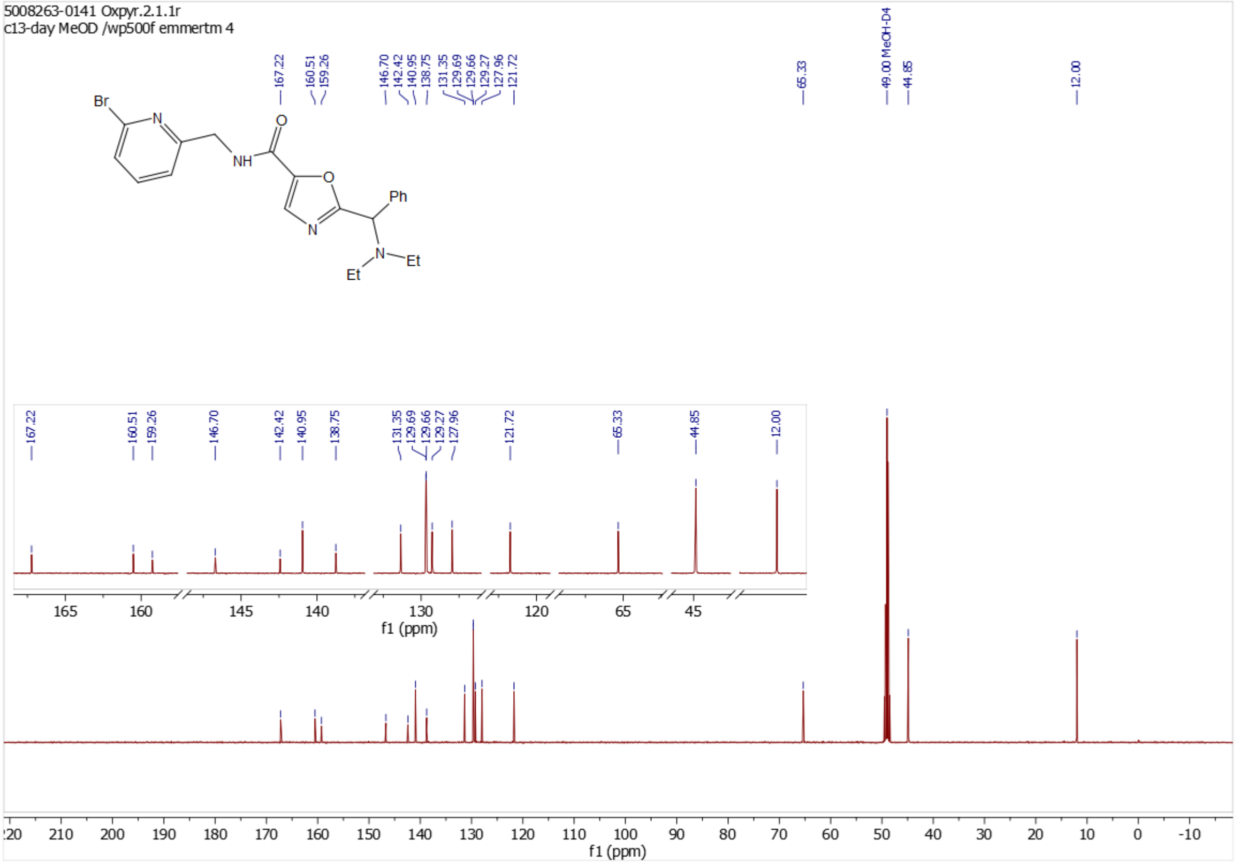
5008263-0141 Oxpyr.2.1.1r
c13-day MeOD /wp500f emmertm 4
167.22
160.51
159.26
146.70
142.42
140.95
138.75
131.35
129.69
129.66
129.27
127.96
121.72
65.33
49.00 MeOH-D4
44.85
12.00
Br
N
NH
O
O
N
Ph
N
Et
Et
f1 (ppm)
f1 (ppm)

N-((5-(5-bromothiophen-2-yl)oxazol-2-yl)(phenyl)methyl)-*N*-ethylethanamine

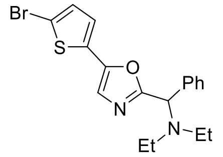

Chemical Formula: $C_{18}H_{19}BrN_2OS$
Exact Mass: 390.04
Molecular Weight: 391.33

The title compound was prepared according to General Procedure 2 (reaction time 4 h).

Yield: 265.5 mg (68%); 75 LCAP before workup/column chromatography

^{1}H NMR (500 MHz, Methanol-*d*$_4$, ppm) δ 7.47 (d, *J* = 7.6 Hz, 2H), 7.35 (t, *J* = 7.2 Hz, 2H), 7.29 (m, 2H), 7.15 (d, *J* = 3.5 Hz, 1H), 7.11 (d, *J* = 3.6 Hz, 1H), 5.14 (s, 1H), 2.67 (dq, *J* = 14.0, 7.0 Hz, 2H), 2.54 (dq, *J* = 13.6, 6.8 Hz, 2H), 1.02 (t, *J* = 7.0 Hz, 6H).

^{13}C NMR (126 MHz, Methanol-*d*$_4$, ppm) δ 164.0, 147.5, 138.4, 132.1, 131.9, 129.5, 129.4, 129.2, 126.1, 122.3, 113.9, 64.9, 44.9, 11.6.

HRMS: Calc. $C_{18}H_{20}BrN_2OS^+$ [M+H$^+$] 391.0474; found 391.0462.

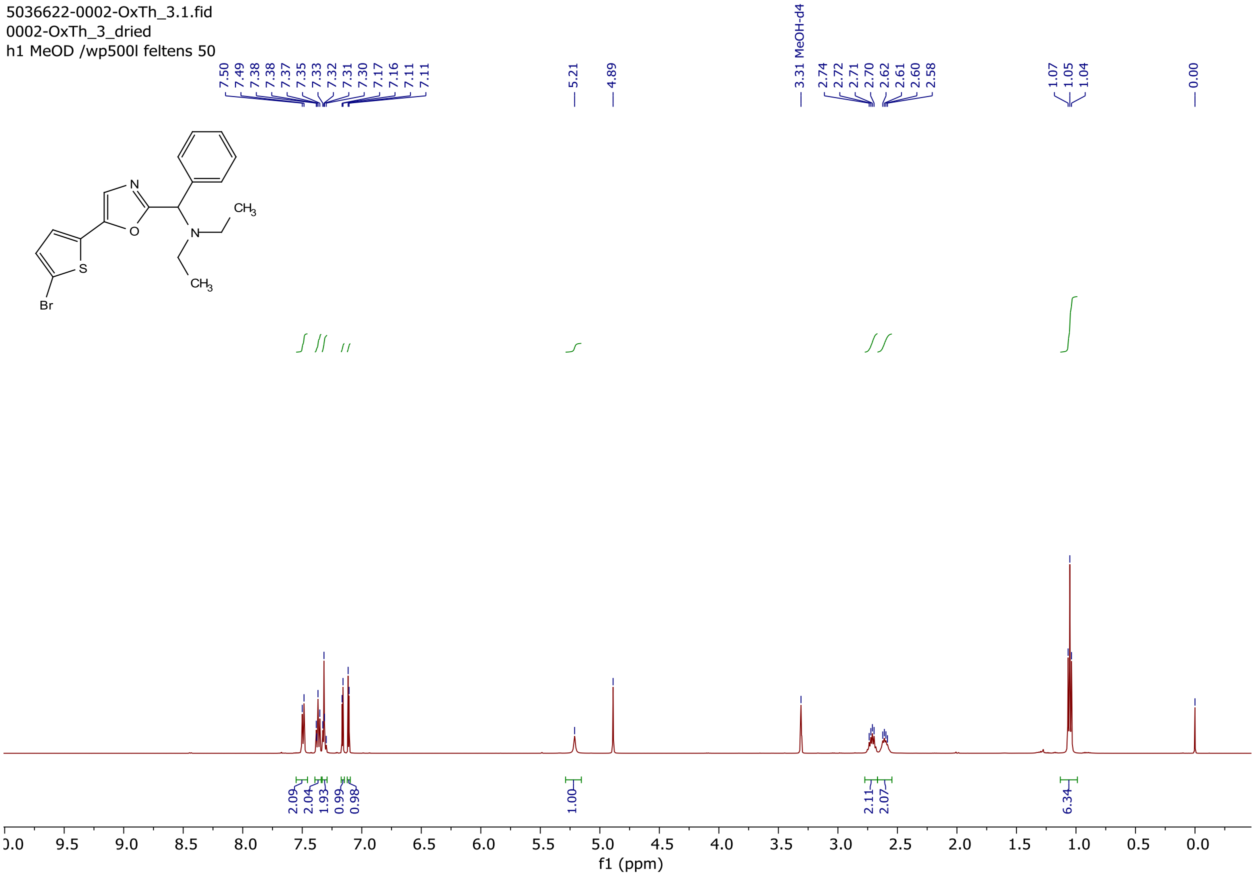

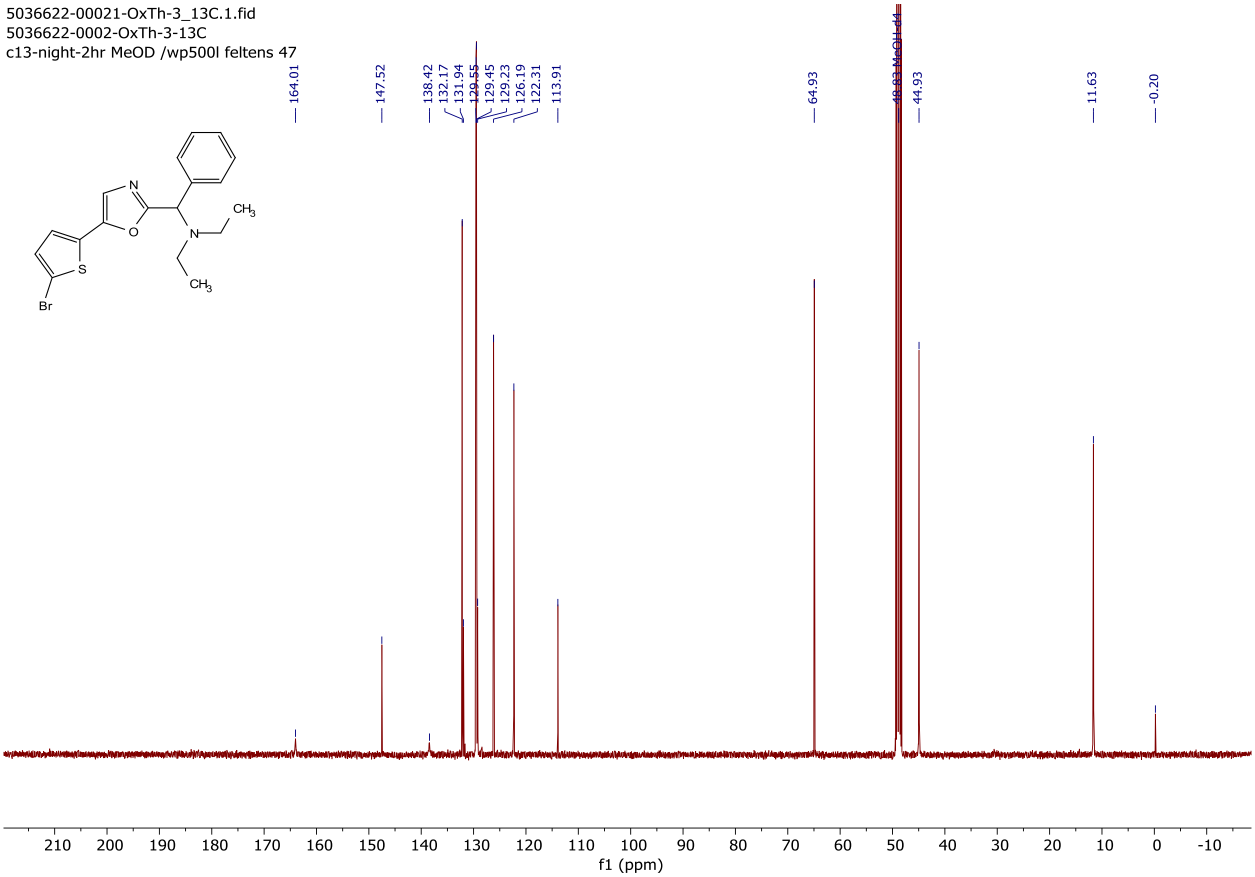
5036622-00021-OxTh-3_13C.1.fid
5036622-0002-OxTh-3-13C
c13-night-2hr MeOD /wp500l feltens 47
164.01
147.52
138.42
132.17
131.94
129.55
129.45
129.23
126.19
122.31
113.91
64.93
48.83 MeOH-d4
44.93
11.63
-0.20
N
O
S
Br
N
CH3
CH3
210 200 190 180 170 160 150 140 130 120 110 100 90 80 70 60 50 40 30 20 10 0 -10
f1 (ppm)

(4-chlorophenyl)(2-((diethylamino)(phenyl)methyl)oxazol-5-yl)methanone

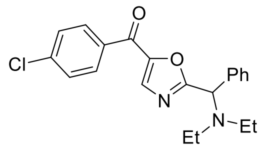

Chemical Formula: $C_{21}H_{21}ClN_2O_2$
Exact Mass: 368.13
Molecular Weight: 368.86

The title compound was prepared according to General Procedure 2 (reaction time 4 h).

Yield: 81.0 mg (22%); 28 LCAP before workup/column chromatography

^{1}H NMR (500 MHz, Methanol-d_4, ppm) δ 8.01 – 7.88 (m, 3H), 7.57 (d, J = 8.2 Hz, 2H), 7.51 (d, J = 7.4 Hz, 2H), 7.34 (m, 3H), 5.27 (s, 1H), 2.69 (dq, J = 13.9, 7.0 Hz, 2H), 2.58 (dq, J = 13.5, 6.7 Hz, 2H), 1.04 (t, J = 6.9 Hz, 6H).

^{13}C NMR (126 MHz, Methanol-d_4, ppm) δ 181.4, 169.3, 150.5, 140.8, 138.7, 136.9, 136.2, 131.8, 130.1, 129.7, 129.7, 129.3, 65.4, 44.9, 12.0.

HRMS: Calc. $C_{21}H_{22}ClN_2O_2^+$ [M+H$^+$] 369.1365; found 369.1358.

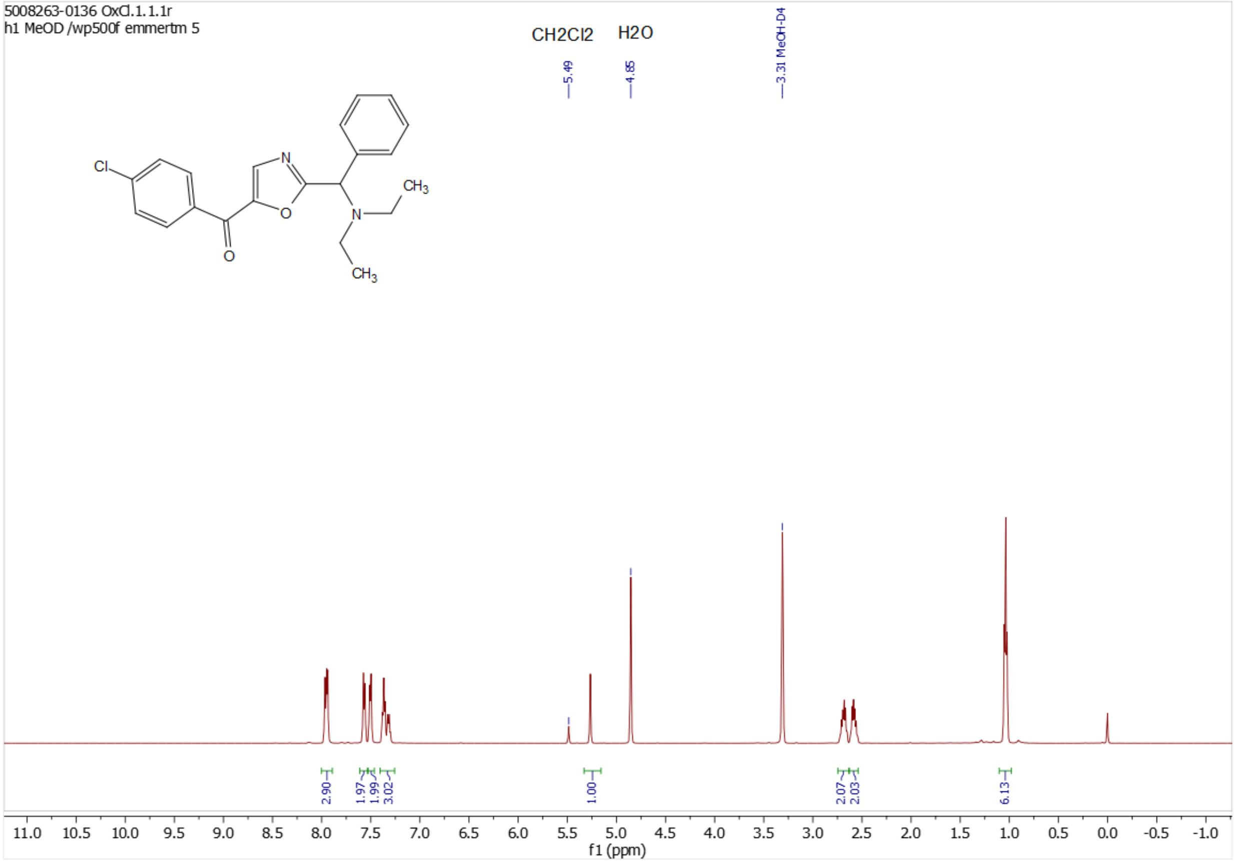

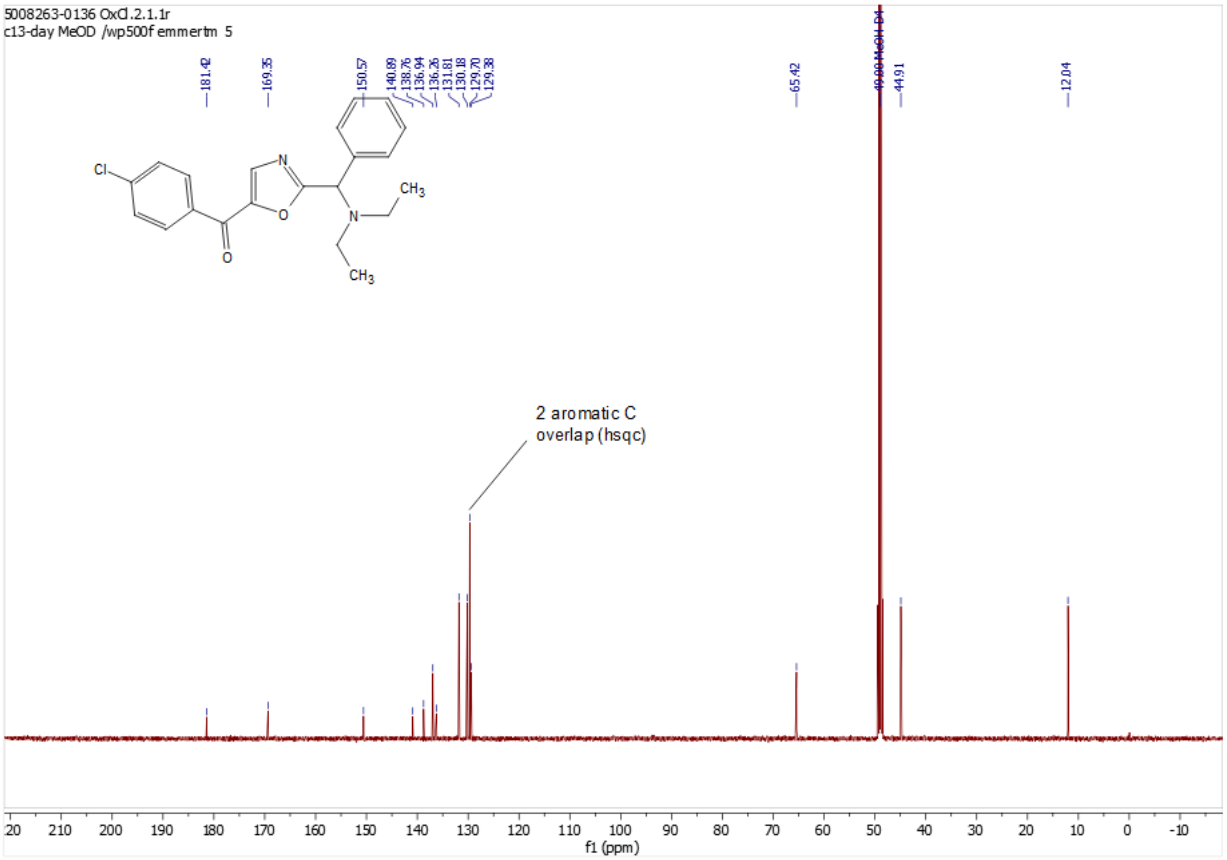

5008263-0136 OxCl.2.1.1r
c13-day MeOD /wp500f emmertm 5
181.42
169.35
150.57
140.89
138.76
136.94
136.26
131.81
130.18
129.70
129.38
65.42
49.00 MeOH-D4
44.91
12.04
Cl
N
O
CH3
2 aromatic C
overlap (hsqc)
f1 (ppm)

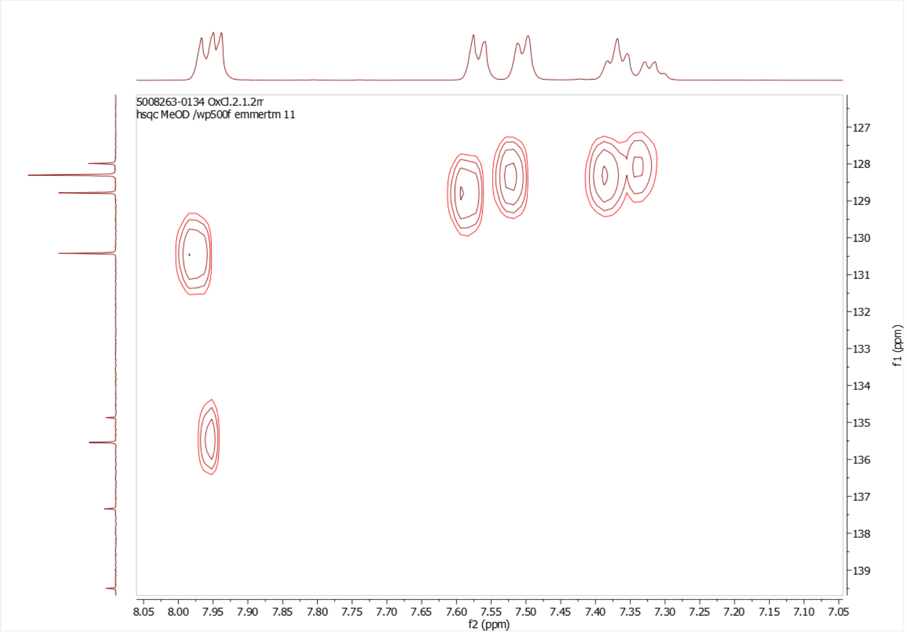

5008263-0134 OxCl.2.1.2rr
hsqc MeOD /wp500f emmertm 11
f2 (ppm)
f1 (ppm)

1-(2-((diethylamino)(phenyl)methyl)oxazol-5-yl)ethan-1-one

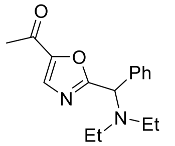

Chemical Formula: $C_{16}H_{20}N_2O_2$
Exact Mass: 272.15
Molecular Weight: 272.35

Synthesis according to General Procedure 2

The title compound was prepared according to General Procedure 2 (reaction time 4 h). After the reaction, UPLC analysis (see below) revealed the presence of a TBS-adduct ($[M+H]^+$ m/z 388, UPLC retention time 1.37 min). 1.0 mL pyridine and 500 uL H_2O. Subsequent column chromatography of the mixture on silica only achieved isolation of the TBS adduct with impurities (1H NMR data suggest that the impurity is the TBS-adduct of the starting material, m/z 227, UPLC retention time 1.53 min); the title compound (hydrolysis product, m/z 273, UPLC retention time 0.73 min) was isolated in pure form.

Crude UPLC/MS before hydrolysis

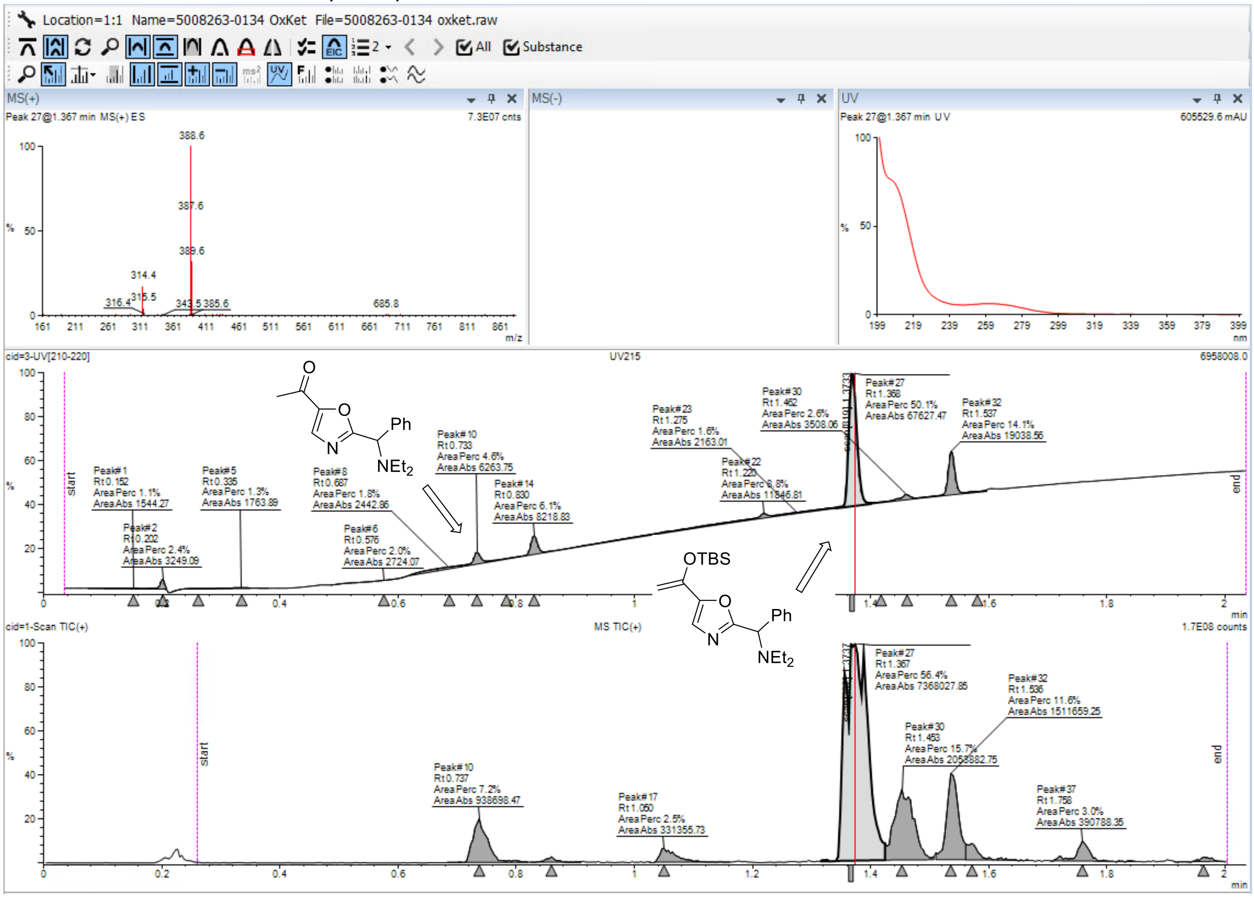

Crude UPLC/MS after hydrolysis

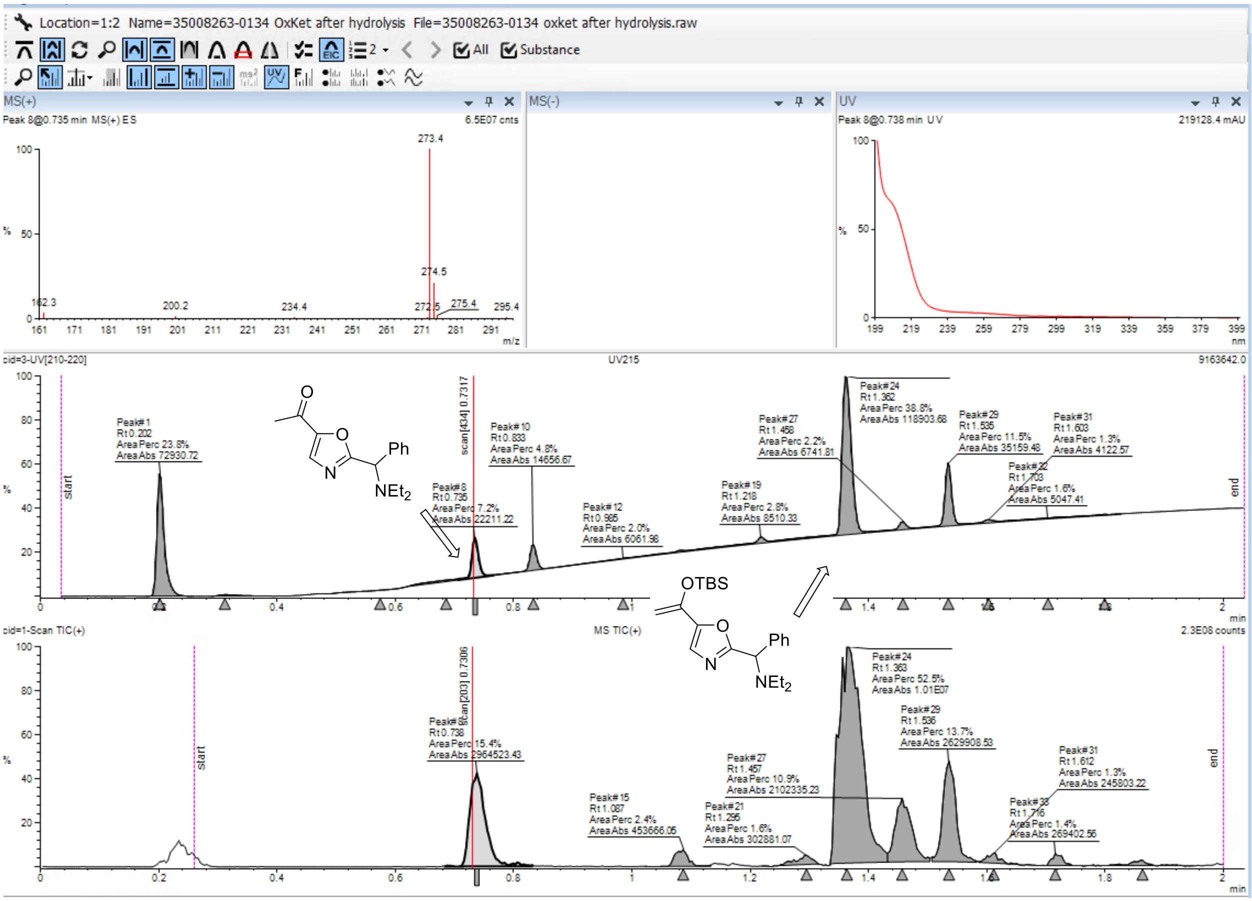

Title compound: 1-(2-((diethylamino)(phenyl)methyl)oxazol-5-yl)ethan-1-one

Synthesis according to General Procedure 2: Yield: 80 mg (29%); 5 LCAP before hydrolysis, 7 LCAP after hydrolysis (before column chromatography)

Synthesis according to General Procedure 2 with modifications for substrates with acidic NH or CH bonds: Yield: 224.3 mg (82%); 39 LCAP before workup (43 LCAP silylated side product), 55 LCAP after workup (before column chromatography; no silylated side product was detected)

^{1}H NMR (500 MHz, Methanol-d_4, ppm) δ 7.94 (s, 1H), 7.46 (m, 2H), 7.32 (m, 3H), 5.21 (s, 1H), 2.66 (dq, J = 14.0, 6.9 Hz, 2H), 2.54 (dq, J = 13.6, 6.9 Hz, 2H), 2.47 (s, 3H), 1.02 (t, J = 6.9 Hz, 6H).

^{13}C NMR (126 MHz, Methanol-d_4, ppm) δ 187.2, 168.8, 151.2, 138.7, 135.0, 129.6, 129.6, 129.2, 65.2, 44.8, 26.5, 12.0.

HRMS: Calc. $C_{16}H_{21}N_2O_2^+$ [M+H$^+$] 273.1598; found 273.1606.

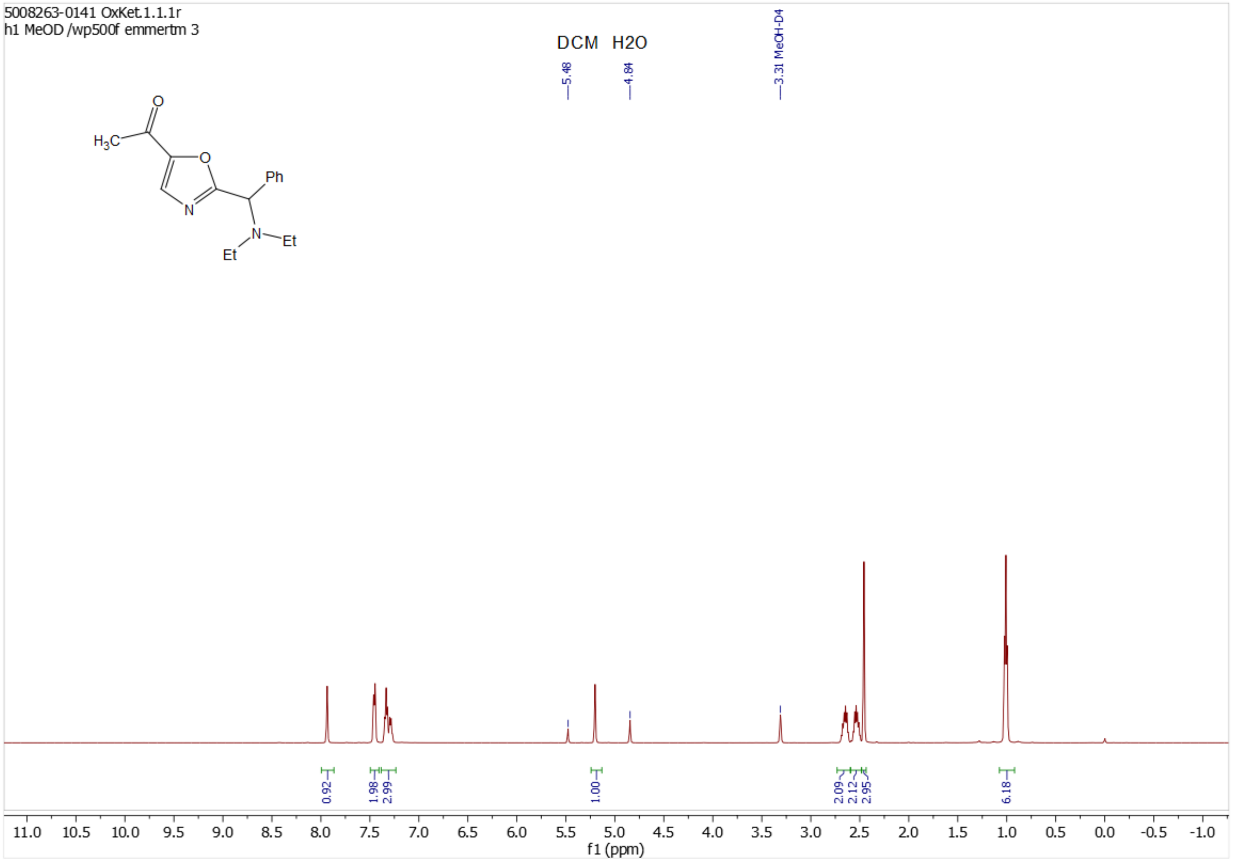

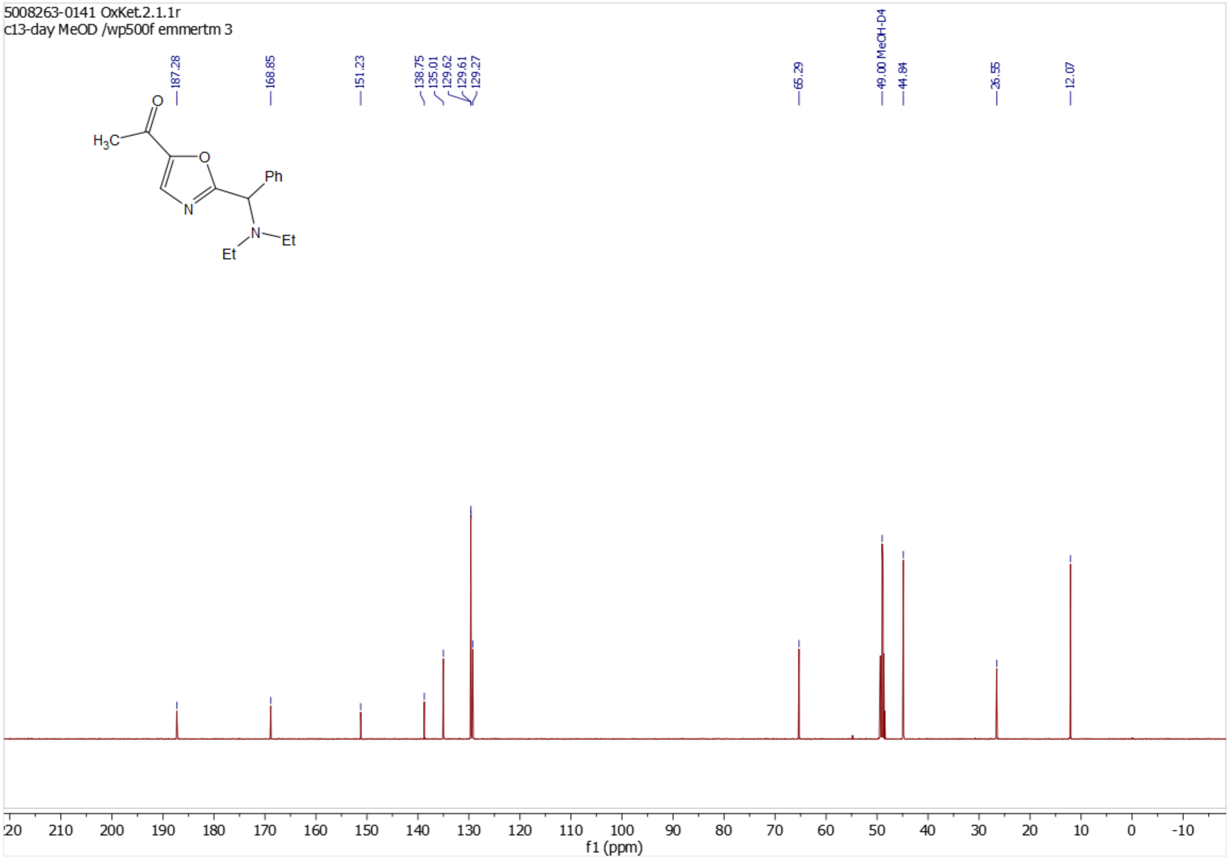
5008263-0141 OxKet.2.1.1r
c13-day MeOD /wp500f emmertm 3
187.28
168.85
151.23
138.75
135.01
129.62
129.61
129.27
65.29
49.00 MeOH-D4
44.84
26.55
12.07
O
H_3C
O
N
Ph
N
Et
Et
f1 (ppm)

Silyl enol ether of 1-(2-((diethylamino)(phenyl)methyl)oxazol-5-yl)ethan-1-one
Yield: 80 mg (29%); 50 LCAP before hydrolysis, 39 LCAP after hydrolysis (before column chromatography)

^{1}H NMR (500 MHz, Methanol-d_4, ppm) δ 7.44 (d, J = 7.3 Hz, 2H), 7.31 (dt, J = 26.8, 6.7 Hz, 3H), 7.04 (s, 1H), 5.11 (s, 1H), 4.89 (s, 1H), 4.50 (s, 1H), 2.66 (dq, J = 13.8, 6.9 Hz, 2H), 2.51 (dq, J = 13.3, 6.6 Hz, 2H), 1.02 (t, J = 6.9 Hz, 6H), 0.98 (s, 9H), 0.21 (s, 6H).

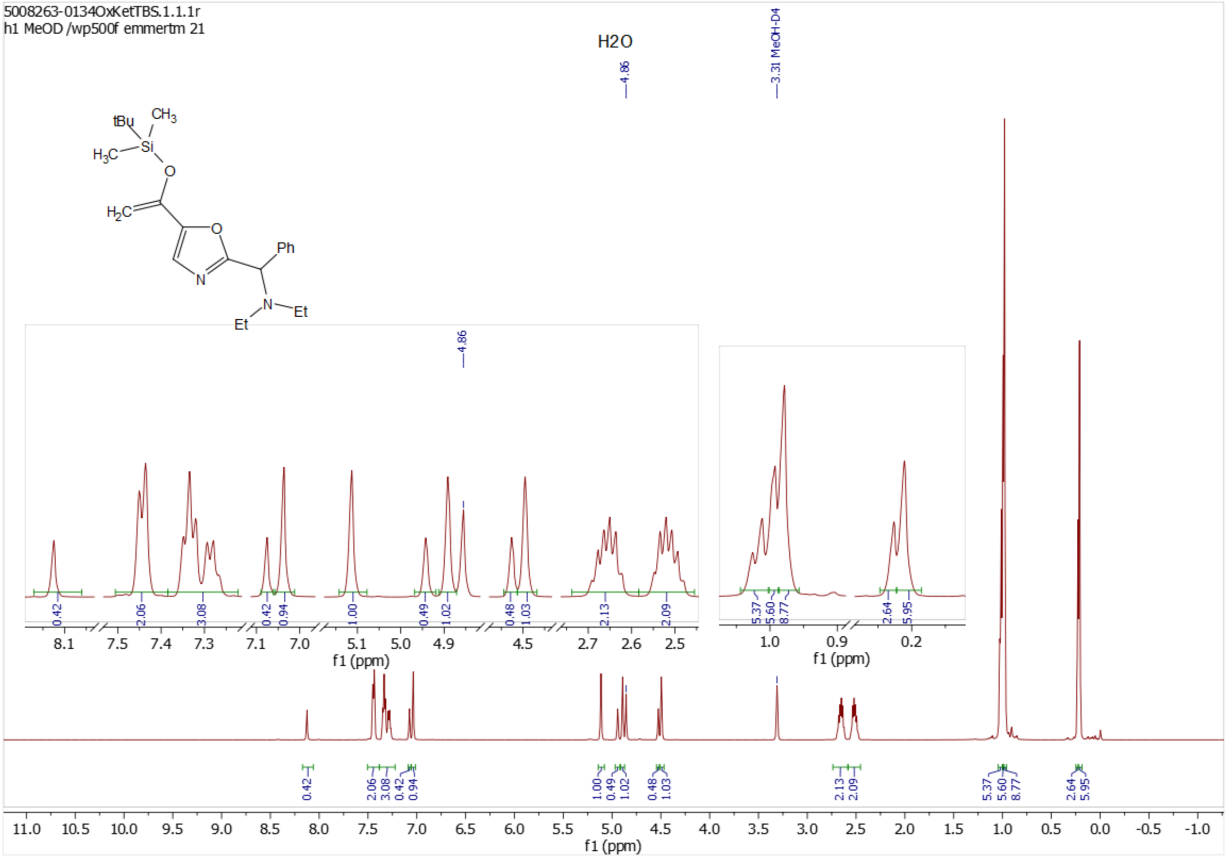

N-((5-(4-bromophenyl)thiazol-2-yl)(phenyl)methyl)-*N*-ethylethanamine

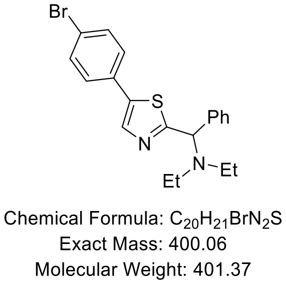

The title compound was prepared according to General Procedure 2 (reaction time 4 h).

Yield: 229 mg (57%); 79 LCAP before workup/column chromatography

^{1}H NMR (500 MHz, Methanol-d_4, ppm) δ 7.90 (s, 1H), 7.55 (m, 4H), 7.45 (d, *J* = 7.5 Hz, 2H), 7.35 (t, *J* = 7.2 Hz, 2H), 7.29 (t, *J* = 7.1 Hz, 1H), 5.18 (s, 1H), 2.72 (dq, *J* = 13.6, 6.8 Hz, 2H), 2.56 (dq, *J* = 13.6, 6.8 Hz, 2H), 1.06 (t, *J* = 6.9 Hz, 6H).

^{13}C NMR (126 MHz, Methanol-d_4, ppm) δ 177.3, 140.0, 139.9, 138.6, 133.3, 131.8, 130.2, 129.5, 129.2, 129.0, 123.1, 69.9, 44.6, 11.9.

HRMS: Calc. $C_{20}H_{22}BrN_2S^+$ [M+H$^+$] 401.0682; found 401.0689.

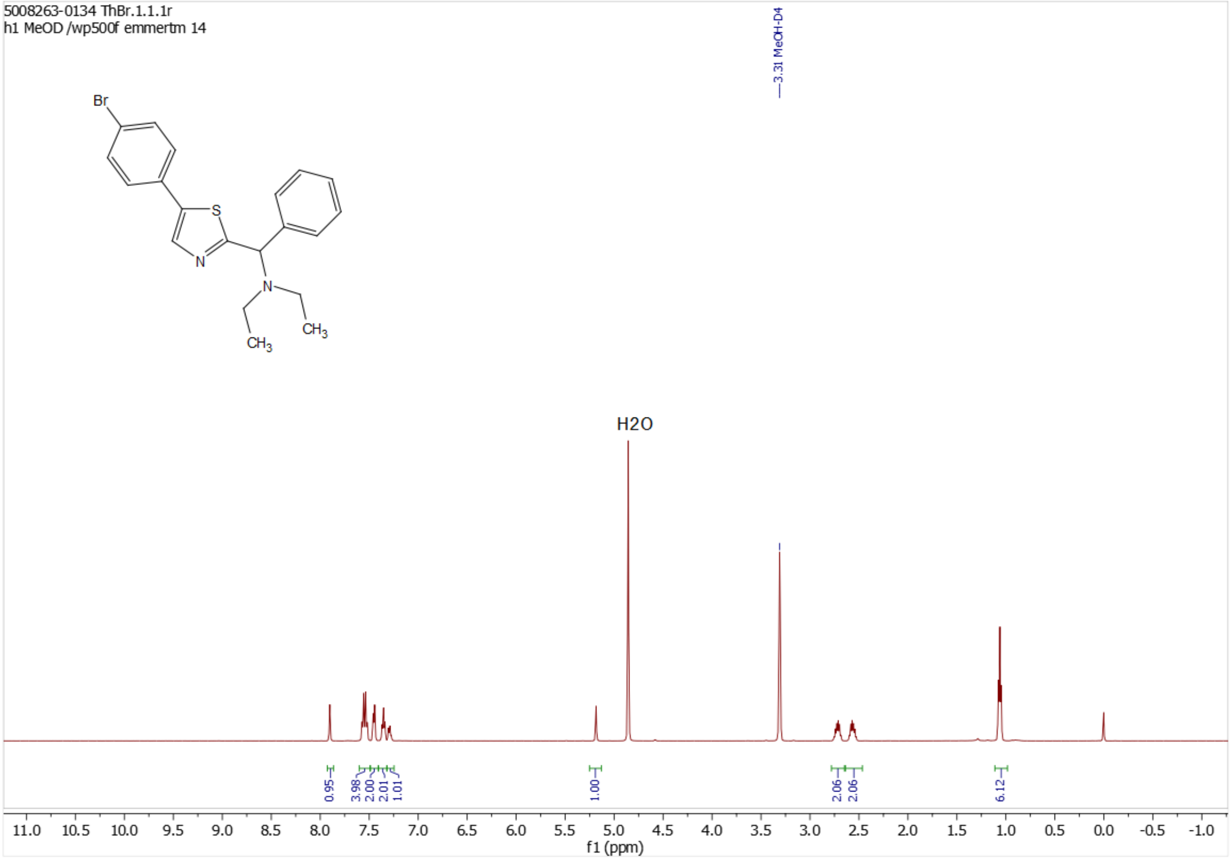

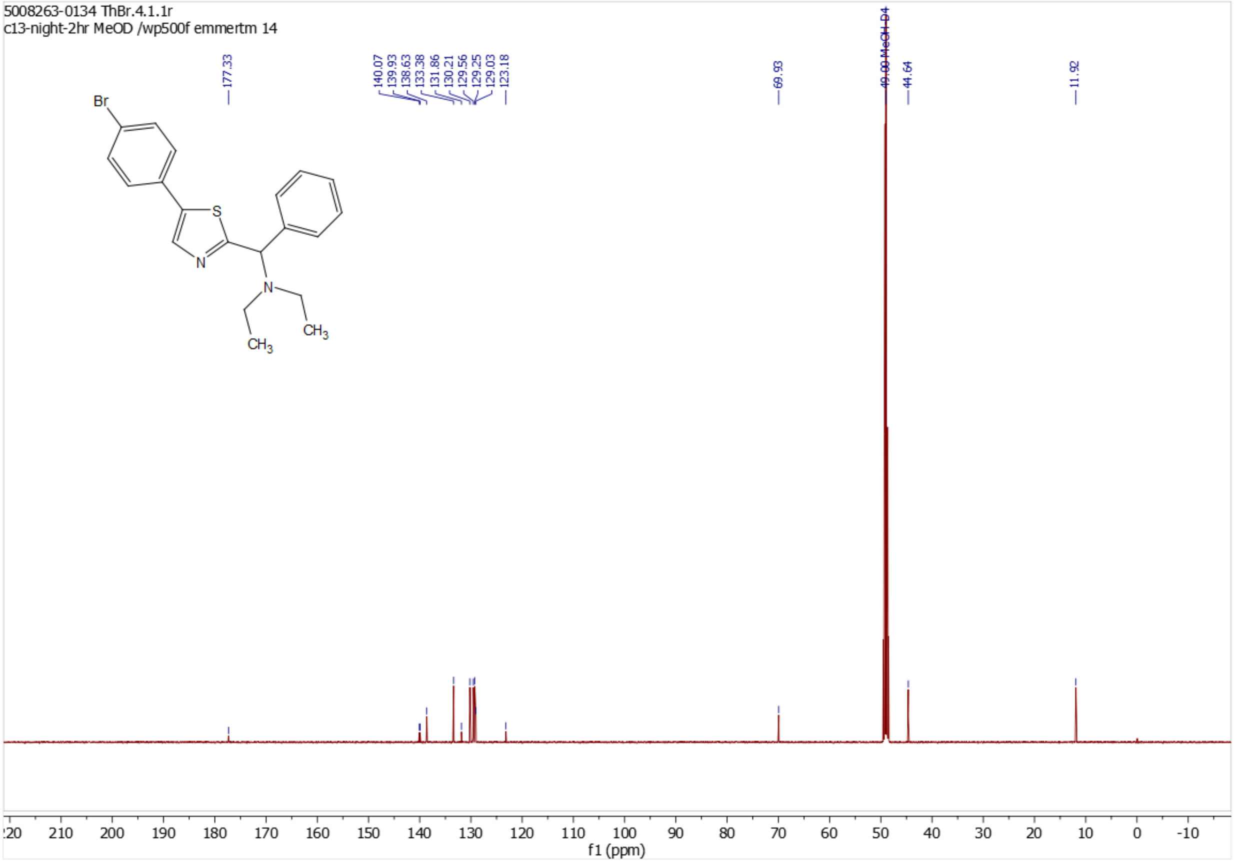
5008263-0134 ThBr.4.1.1r
c13-night-2hr MeOD /wp500f emmertm 14
177.33
140.07
139.93
138.63
133.38
131.86
130.21
129.56
129.25
129.03
123.18
69.93
49.00 MeOH-D4
44.64
11.92
Br
S
N
N
CH3
CH3
f1 (ppm)

N-((6-Bromobenzo[d]thiazol-2-yl)(phenyl)methyl)-*N*-ethylethanamine

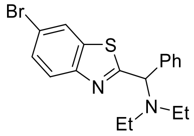

Chemical Formula: $C_{18}H_{19}BrN_2S$
Exact Mass: 374.05
Molecular Weight: 375.33

The title compound was prepared according to General Procedure 2 (reaction time 4 h).

Yield: 272 mg (73%); 71 LCAP before workup/column chromatography

^{1}H NMR (500 MHz, Methanol-d_4, ppm) δ 8.10 (s, 1H), 7.71 (d, *J* = 8.6 Hz, 1H), 7.53 (d, *J* = 8.6 Hz, 1H), 7.45 (d, *J* = 7.4 Hz, 2H), 7.35 (t, *J* = 7.1 Hz, 2H), 7.29 (t, *J* = 6.7 Hz, 1H), 5.26 (s, 1H), 2.74 (dq, *J* = 13.7, 6.9 Hz, 2H), 2.53 (dq, *J* = 13.3, 6.6 Hz, 2H), 1.06 (t, *J* = 6.9 Hz, 6H).

^{13}C NMR (126 MHz, Methanol-d_4, ppm) δ 180.5, 153.0, 138.8, 138.3, 130.5, 130.4, 129.5, 129.1, 125.6, 124.6, 119.6, 70.2, 44.7, 11.9.

HRMS: Calc. $C_{18}H_{20}BrN_2S^+$ [M+H$^+$] 375.0525; found 375.0513.

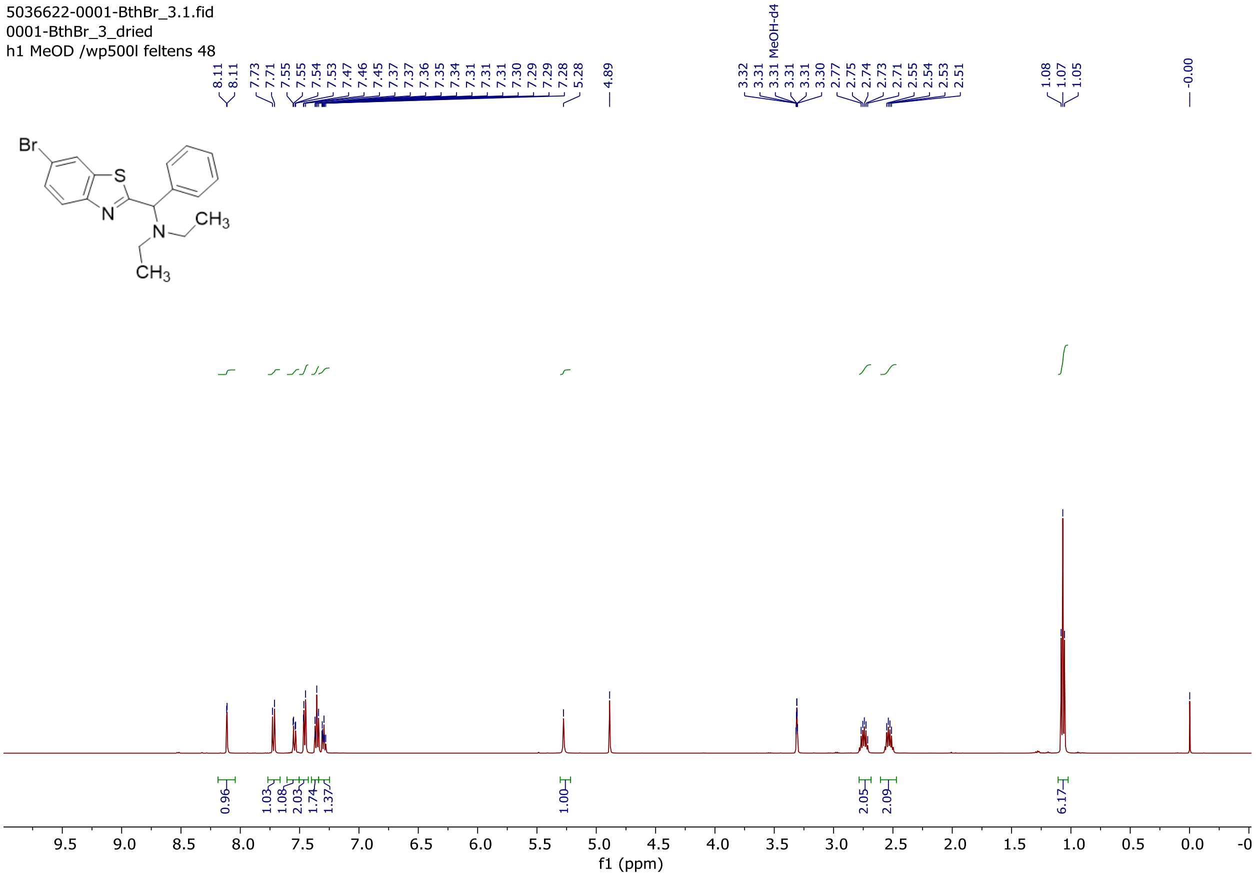

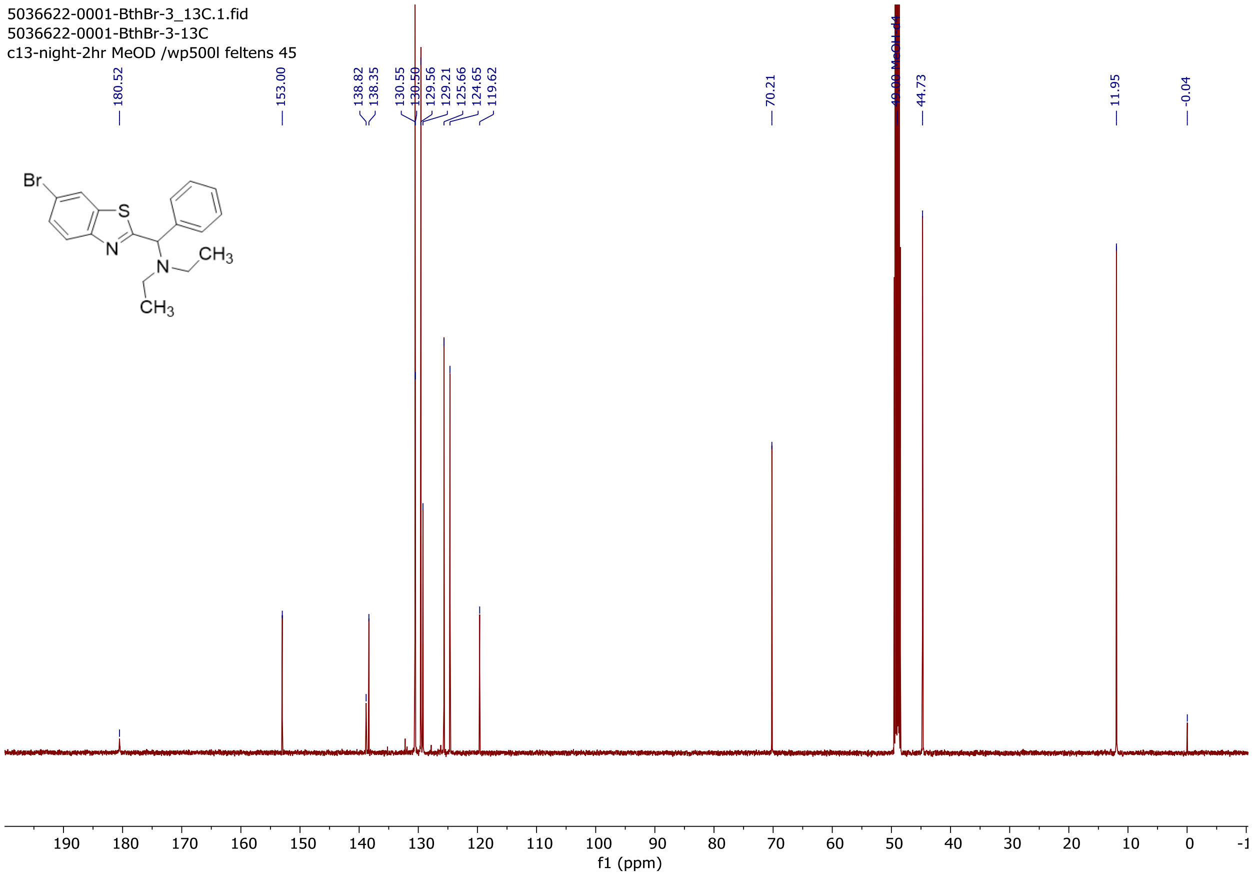
5036622-0001-BthBr-3_13C.1.fid
5036622-0001-BthBr-3-13C
c13-night-2hr MeOD /wp500l feltens 45
180.52
153.00
138.82
138.35
130.55
130.50
129.56
129.21
125.66
124.65
119.62
70.21
44.73
11.95
-0.04
Br
S
N
N
CH3
CH3
f1 (ppm)

N-(benzo[d]thiazol-2-yl(phenyl)methyl)-*N*-ethylethanamine

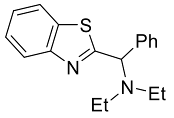

Chemical Formula: $C_{18}H_{20}N_2S$
Exact Mass: 296.13
Molecular Weight: 296.43

The title compound was prepared according to General Procedure 2 (reaction time 4 h).

Yield: 275 mg (93%); 81 LCAP before workup/column chromatography

^{1}H NMR (500 MHz, Methanol-d_4, ppm) δ 7.92 (d, *J* = 7.9 Hz, 1H), 7.84 (d, *J* = 8.0 Hz, 1H), 7.48 (d, *J* = 7.4 Hz, 2H), 7.43 (t, *J* = 7.5 Hz, 1H), 7.35 (q, *J* = 7.7 Hz, 3H), 7.28 (t, *J* = 6.9 Hz, 1H), 5.27 (s, 1H), 2.74 (dq, *J* = 13.6, 6.8 Hz, 2H), 2.56 (dq, *J* = 13.3, 6.6 Hz, 2H), 1.06 (t, *J* = 6.9 Hz, 6H).

^{13}C NMR (126 MHz, Methanol-d_4, ppm) δ 179.3, 153.7, 139.3, 136.3, 130.4, 129.5, 129.1, 127.1, 126.3, 123.2, 122.9, 70.4, 44.6, 11.8.

HRMS: Calc. $C_{18}H_{21}N_2S^+$ [M+H$^+$] 297.1420; found 297.1411.

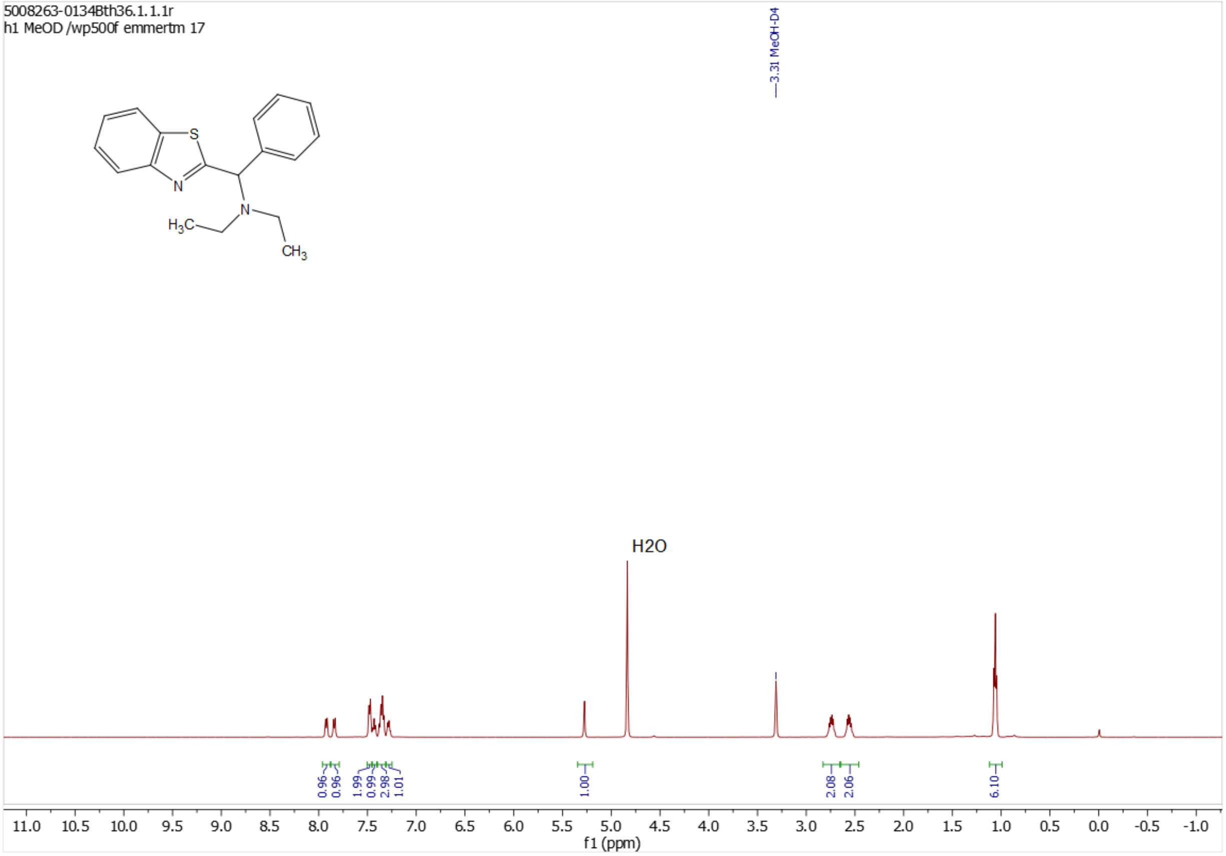

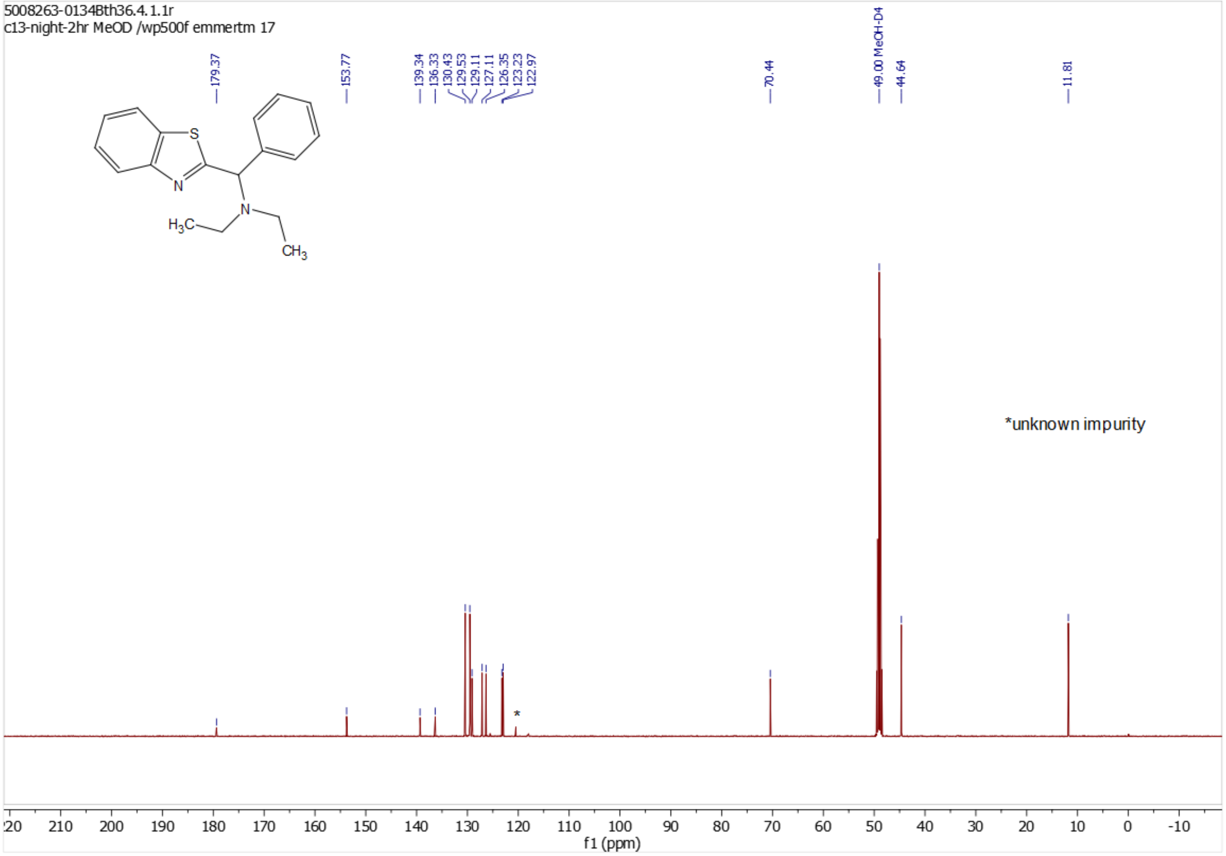
5008263-0134Bth36.4.1.1r
c13-night-2hr MeOD /wp500f emmertm 17
179.37
153.77
139.34
136.33
130.43
129.53
129.11
127.11
126.35
123.23
122.97
70.44
49.00 MeOH-D4
44.64
11.81
S
N
N
H_3C
CH_3
*unknown impurity
*
f1 (ppm)

N-((5-Bromooxazolo[4,5-b]pyridin-2-yl)(phenyl)methyl)-*N*-ethylethanamine

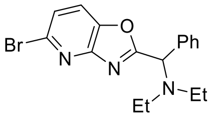

Chemical Formula: $C_{17}H_{18}BrN_3O$
Exact Mass: 359.06
Molecular Weight: 360.26

The title compound was prepared according to General Procedure 2 (reaction time 3 h) using 4 equiv. TBSOTf instead of 3 equiv. TBSOTf and 2 equiv. $^{i}Pr_2NEt$ instead of 1 equiv. $^{i}Pr_2NEt$. Workup was performed in analogy to the modified procedure for compounds with acidic CH or NH bonds, using 4 equiv. KF/pyridine as a quenching reagent.

Yield: 192 mg (76%); 76 LCAP before workup/column chromatography

^{1}H NMR (500 MHz, Methanol-d_4, ppm) δ 7.97 (d, *J* = 8.4 Hz, 1H), 7.59 (d, *J* = 8.5 Hz, 1H), 7.51 (d, *J* = 7.3 Hz, 2H), 7.33 (m, 3H), 5.35 (s, 1H), 2.71 (dq, *J* = 13.6, 6.9 Hz, 2H), 2.62 (dq, *J* = 13.2, 6.6 Hz, 2H), 1.04 (t, *J* = 6.8 Hz, 6H).

^{13}C NMR (126 MHz, Methanol-d_4, ppm) δ 171.9, 156.3, 143.9, 138.3, 138.2, 129.7, 129.7, 129.4, 125.9, 122.9, 65.8, 44.9, 12.1.

HRMS: Calc. $C_{17}H_{19}BrN_3O^+$ [M+H$^+$] 360.0706; found 360.0705.

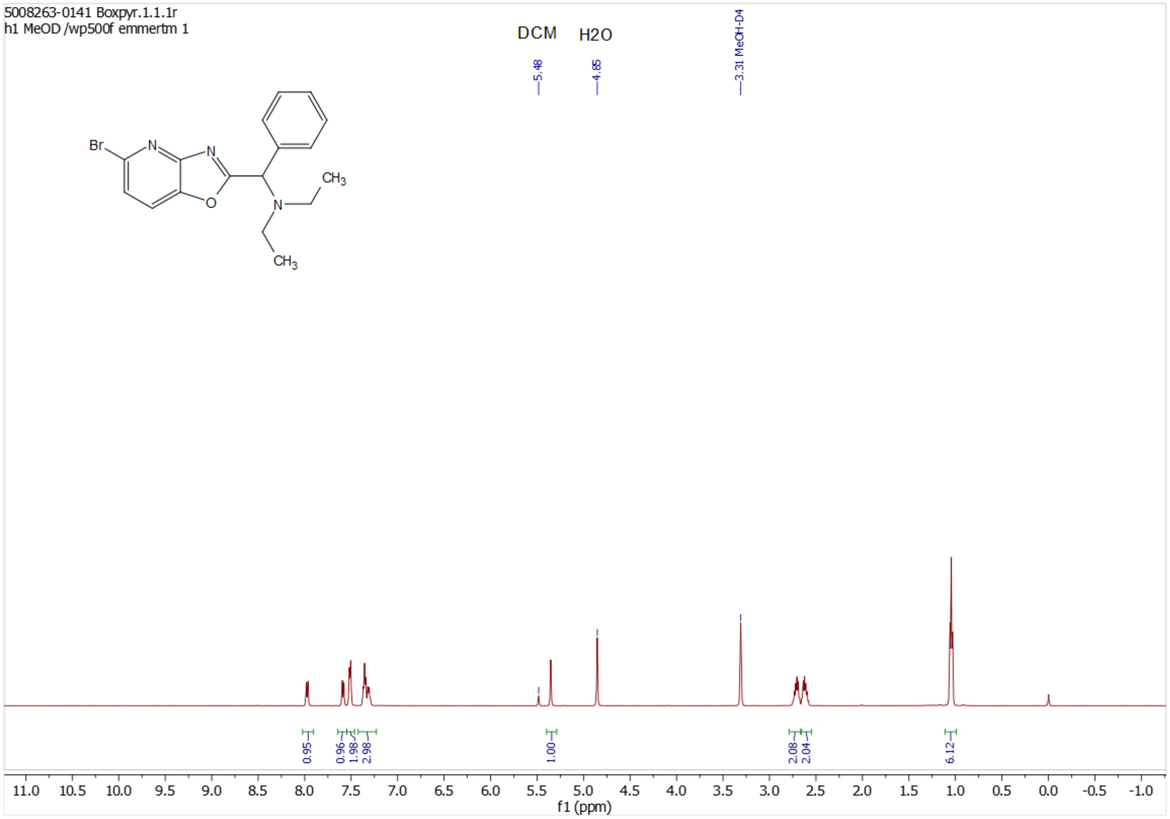

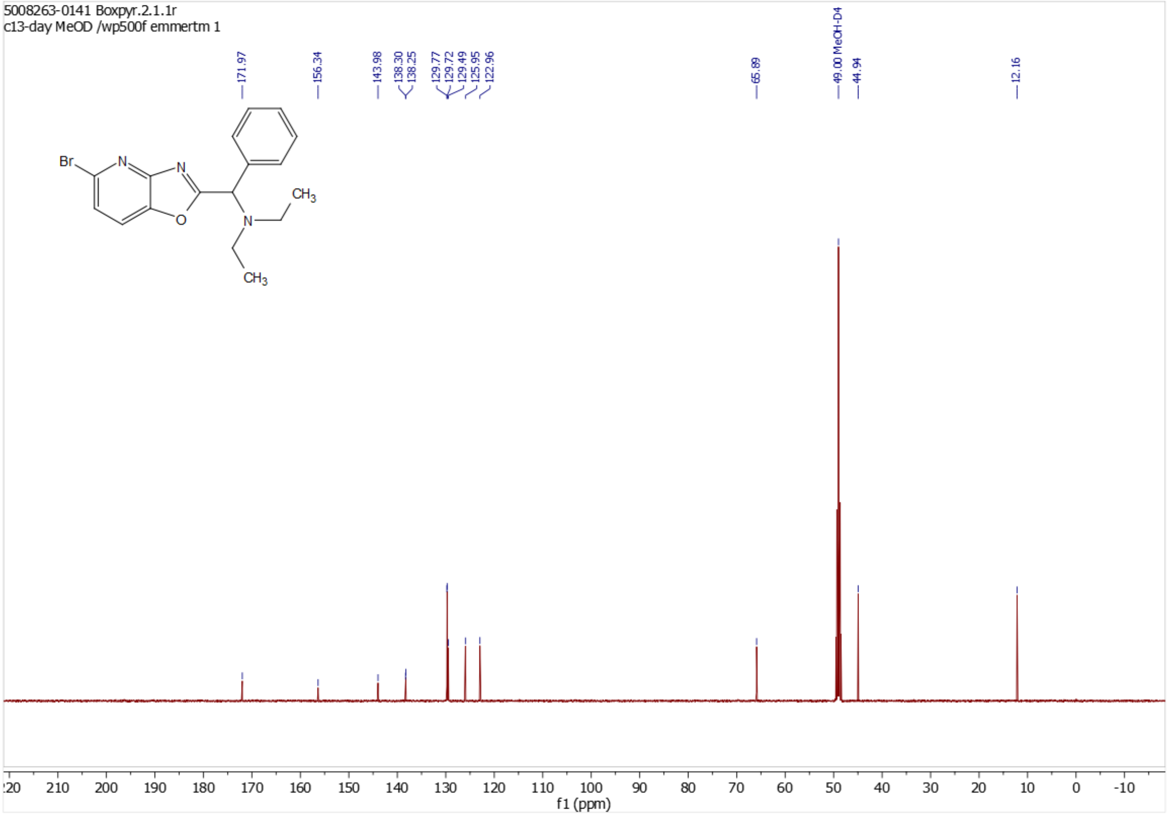
5008263-0141 Boxpyr.2.1.1r
c13-day MeOD /wp500f emmertm 1
171.97
156.34
143.98
138.30
138.25
129.77
129.72
129.49
125.95
122.96
65.89
49.00 MeOH-D4
44.94
12.16
Br
N
N
O
N
CH3
CH3
f1 (ppm)

N-((7-Bromo-5-methoxybenzo[d]oxazol-2-yl)(phenyl)methyl)-*N*-ethylethanamine

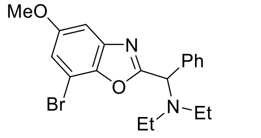

Chemical Formula: $C_{19}H_{21}BrN_2O_2$
Exact Mass: 388.0786
Molecular Weight: 389.2930

The title compound was prepared according to General Procedure 2 (reaction time 3 h).

Yield: 307 mg (79%); 76 LCAP before workup/column chromatography

^{1}H NMR (500 MHz, Methanol-*d*$_4$, ppm) δ 7.50 (d, *J* = 7.3 Hz, 2H), 7.31 (m, 3H), 7.17 (s, 1H), 7.12 (s, 1H), 5.26 (s, 1H), 3.81 (s, 3H), 2.70 (dq, *J* = 13.6, 6.8 Hz, 2H), 2.58 (dq, *J* = 13.0, 6.5 Hz, 2H), 1.03 (t, *J* = 6.9 Hz, 6H).

^{13}C NMR (126 MHz, Methanol-*d*$_4$, ppm) δ 168.6, 159.5, 144.8, 142.8, 138.8, 129.6, 129.6, 129.2, 118.5, 117.8, 103.4, 65.6, 56.7, 44.9, 12.1.

HRMS: Calc. $C_{19}H_{22}BrN_2O_2^+$ [M+H$^+$] 389.0859; found 389.0856.

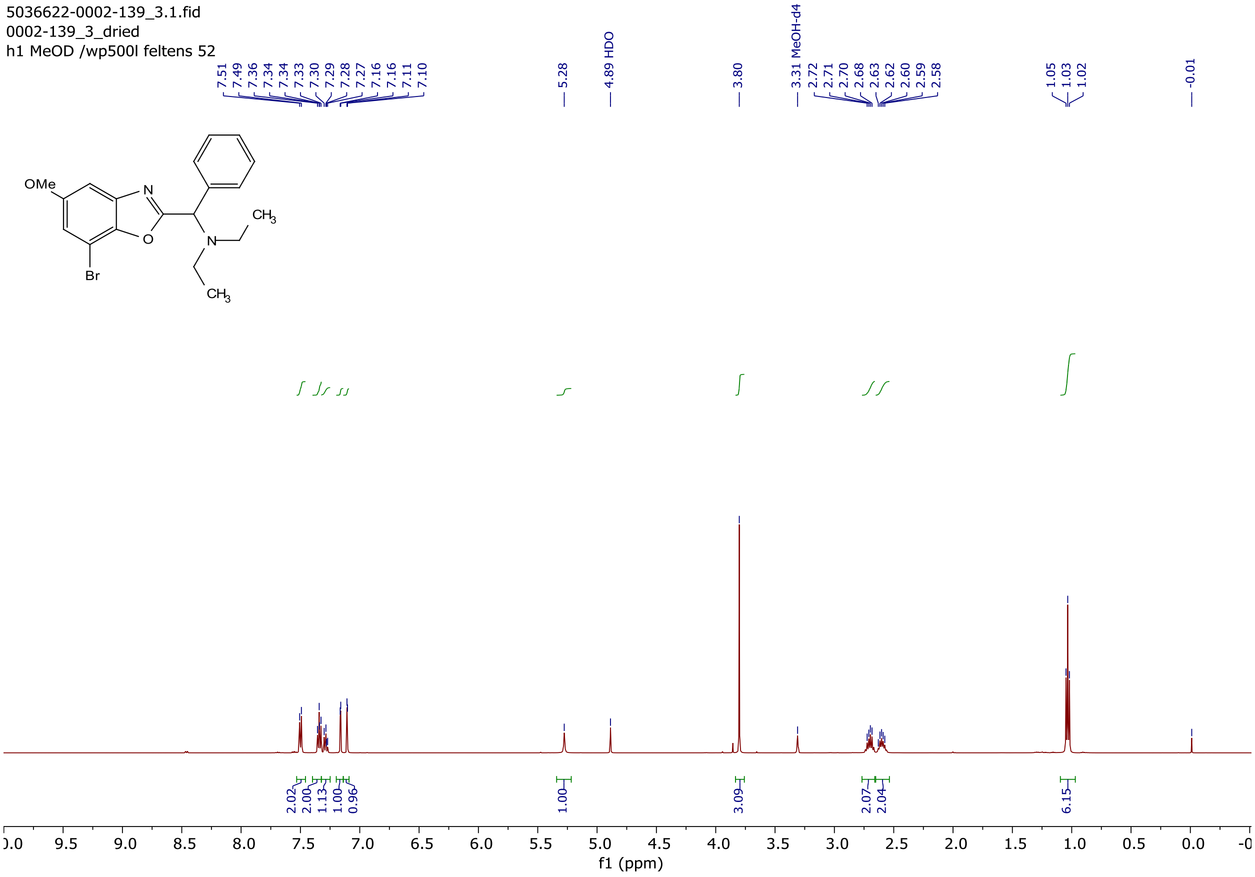

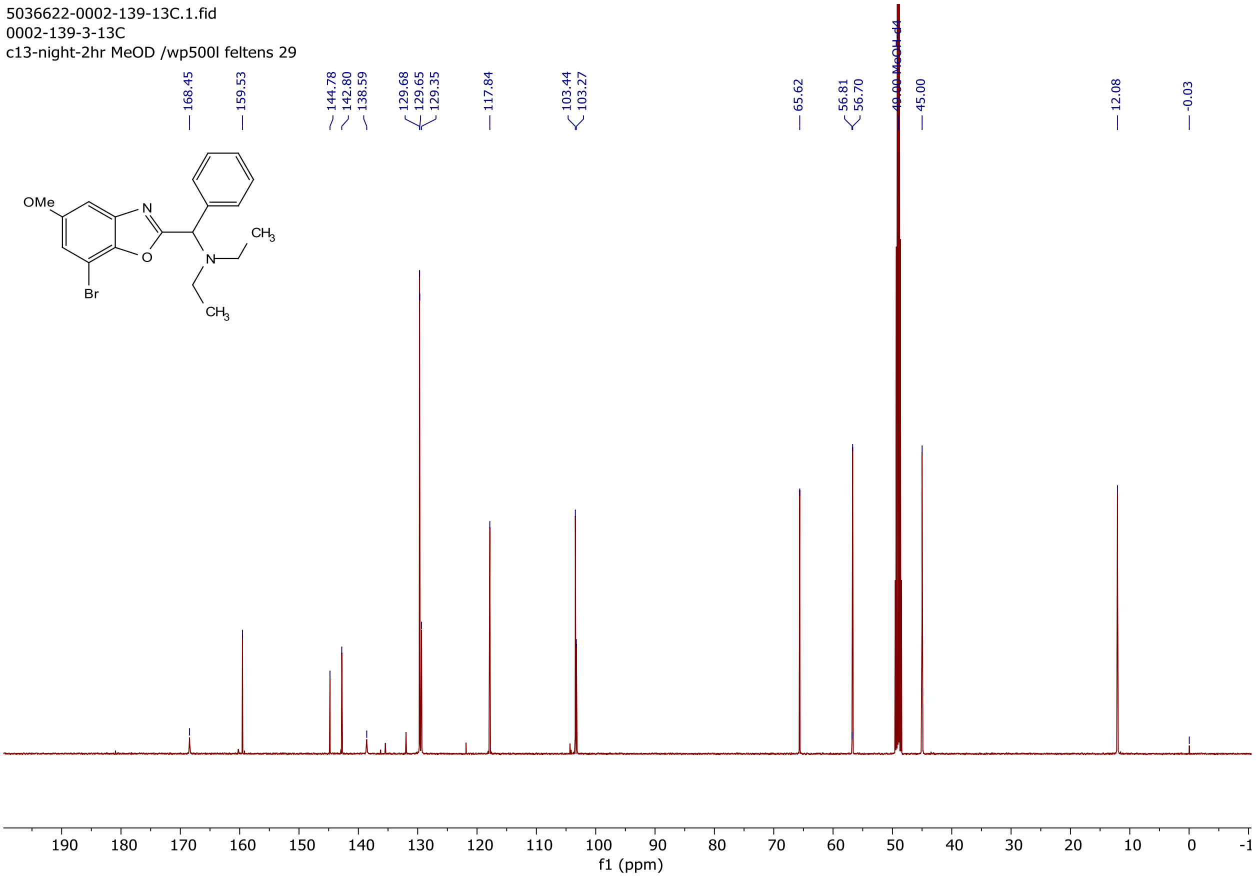
5036622-0002-139-13C.1.fid
0002-139-3-13C
c13-night-2hr MeOD /wp500l feltens 29
168.45
159.53
144.78
142.80
138.59
129.68
129.65
129.35
117.84
103.44
103.27
65.62
56.81
56.70
49.00 MeOH-d4
45.00
12.08
-0.03
OMe
N
O
Br
N
CH3
CH3
190 180 170 160 150 140 130 120 110 100 90 80 70 60 50 40 30 20 10 0 -1
f1 (ppm)

C-H aminoalkylation of imidazole and benzimidazole via *in-situ* tosylation

Imidazole: *N*-((1H-imidazol-2-yl)(phenyl)methyl)-N-ethylethanamine

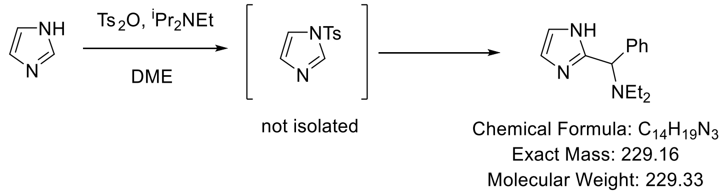

Imidazole (0.068 g, 1.00 mmol, 1.00 eq.) and tosyl anhydride (0.938 g, 1.20 mmol, 1.20 equiv.) were weighed into a 2-dram vial and brought into the glovebox together with an empty 2-dram vial. 1,2-Dimethoxyethane (1.5 ml) was added to the vial containing imidazole, followed by addition of N,*N*-diethylpropan-2-amine (0.388 ml, 0.288 g, 2.50 mmol, 2.50 equiv.). This resulted at first in a solution, with subsequent precipitate formation after ~1 min. The vial was stirred in the glovebox for 30 min at room temperature.

tert-Butyldimethylsilyl trifluoromethanesulfonate (0.689 ml, 0.793 g, 3.00 mmol, 3.0 equiv.), *N*,*N*-diethyl-1,1,1-trimethylsilanamine (0.237 ml, 0.182 g, 1.25 mmol, 1.25 equiv.),and benzaldehyde (0.102 ml, 0.106 g, 1.00 mmol, 1.00 equiv.) were mixed in 1,2-dimethoxyethane (1.0 ml) in the empty vial and stirred for 10 min at room temperature. Then, the second solution was added to the first mixture and the combined reaction mixture was removed from the glovebox and stirred at 50 °C for 18 h.

To hydrolyze the tosyl protecting group, pyridine (2.0 mL) and water (0.20 mL) were added and the reaction was stirred at 50 °C for 2 h. The resulting mixture was concentrated in vacuum and purified by column chromatography.

Yield: 103 mg (45%); 39 LCAP before tosyl hydrolysis, 22 LCAP after hydrolysis & before column chromatography

For spectroscopic (NMR/HRMS) characterization, see page 70

Benzimidazole: *N*-((1H-benzo[d]imidazol-2-yl)(phenyl)methyl)-N-ethylethanamine

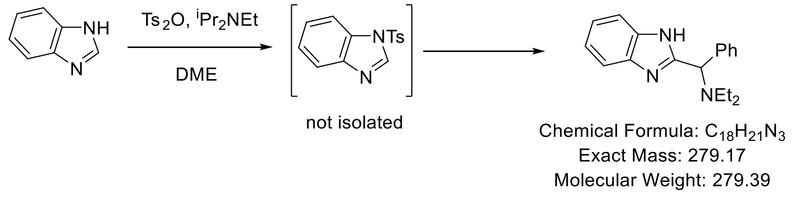

Benzimidazole (0.118 g, 1.00 mmol, 1.00 equiv.) and tosyl anhydride (0.938 g, 1.20 mmol, 1.20 equiv.) were weighed into a 2-dram vial and brought into the glovebox together with an empty 2-dram vial. 1,2-Dimethoxyethane (1.5 ml) was added to the vial containing benzimidazole, followed by addition of *N,N*-diethylpropan-2-amine (0.388 ml, 0.288 g, 2.50 mmol, 2.50 equiv.). This resulted at first in a solution, with

subsequent precipitate formation after ~1 min. The vial was stirred in the glovebox for 30 min at room temperature.

tert-Butyldimethylsilyl trifluoromethanesulfonate (0.689 ml, 0.793 g, 3.00 mmol, 3.0 equiv.), *N,N*-diethyl-1,1,1-trimethylsilanamine (0.237 ml, 0.182 g, 1.25 mmol, 1.25 equiv.), and benzaldehyde (0.102 ml, 0.106 g, 1.00 mmol, 1.00 equiv.) were mixed in 1,2-dimethoxyethane (1.0 ml) in the empty vial and stirred for 10 min at room temperature. Then, the second solution was added to the first mixture and the combined reaction mixture was removed from the glovebox and stirred at 50 °C for 18 h.

To hydrolyze the tosyl protecting group, pyridine (2.0 mL) and water (0.20 mL) were added and the reaction was stirred at 50 °C for 2 h. The resulting mixture was concentrated in vacuum and purified by column chromatography.

Yield: 207 mg (74%); 22 LCAP before tosyl hydrolysis, 16 LCAP after hydrolysis & before column chromatography

^{1}H NMR (500 MHz, Methanol-*d*$_4$, ppm) δ 7.57 (d, *J* = 7.2 Hz, 2H), 7.50 (broad s, 2H), 7.33 (t, *J* = 7.1 Hz, 2H), 7.25 (t, *J* = 6.9 Hz, 1H), 7.21 – 7.15 (m, 2H), 5.09 (s, 1H), 2.63 (q, *J* = 6.2 Hz, 4H), 1.03 (t, *J* = 6.8 Hz, 6H).

^{13}C NMR (126 MHz, Methanol-*d*$_4$, ppm) δ 157.1, 140.7, 129.6, 129.5, 128.8, 128.6, 123.4 (broad, 4 C), 67.4, 44.3, 11.2.

HRMS: Calc. $C_{18}H_{22}N_3^+$ [M+H$^+$] 280.1808; found 280.1812.

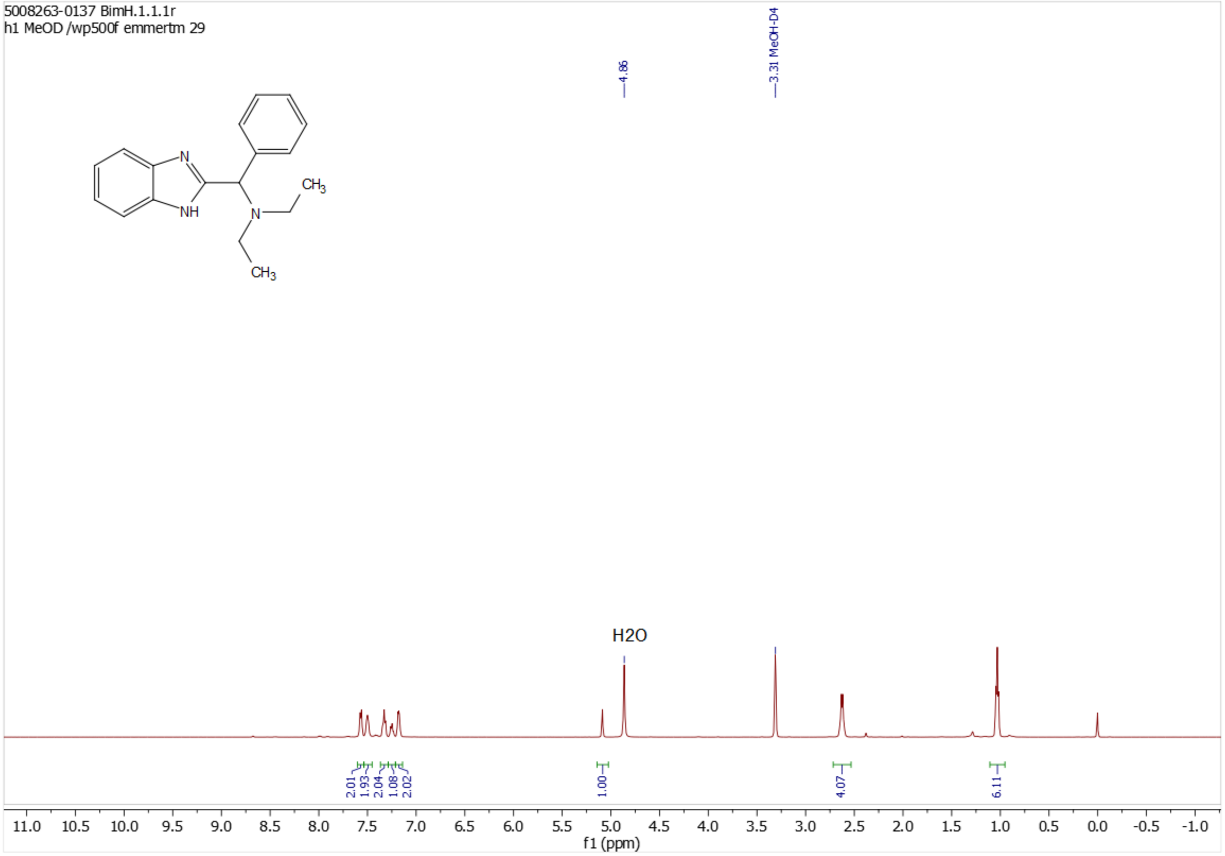

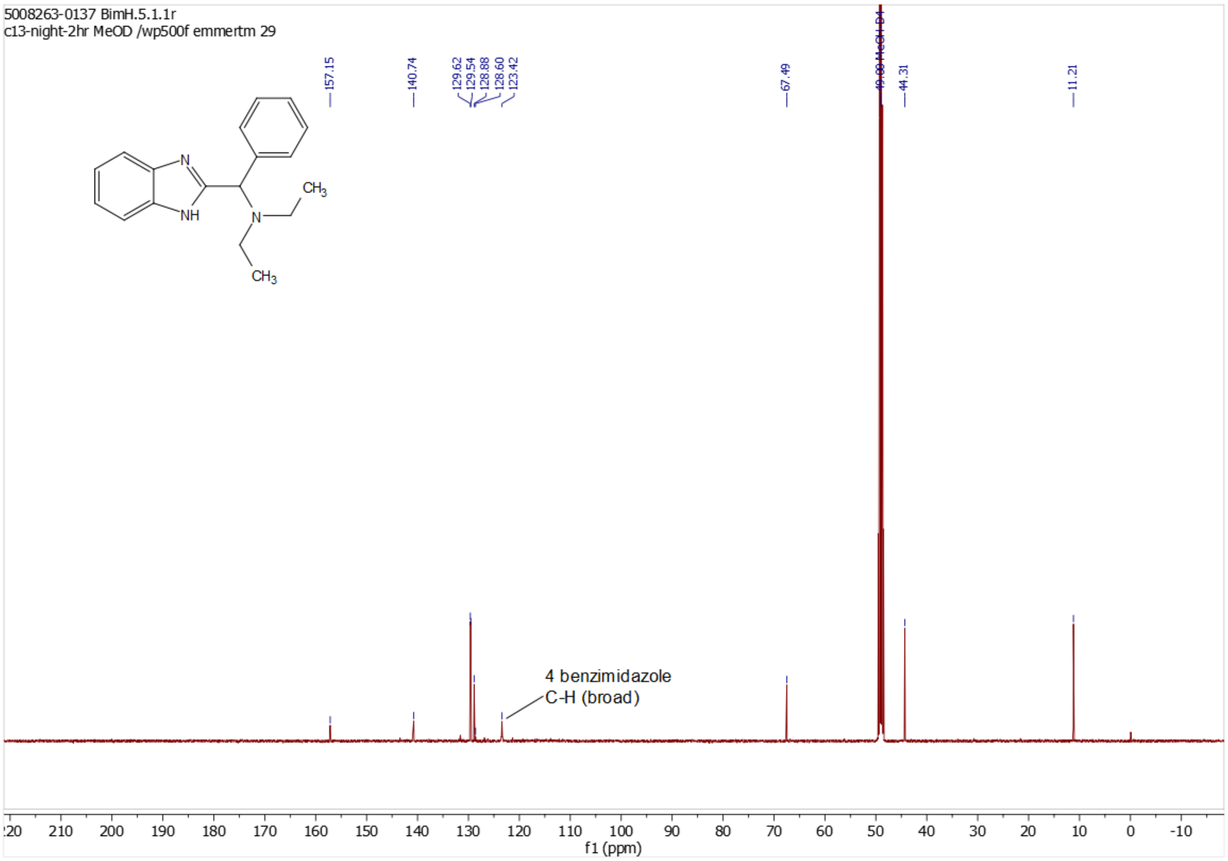
5008263-0137 BimH.5.1.1r
c13-night-2hr MeOD /wp500f emmertm 29
157.15
140.74
129.62
129.54
128.88
128.60
123.42
67.49
44.31
11.21
N
NH
N
CH3
CH3
4 benzimidazole
C-H (broad)
f1 (ppm)

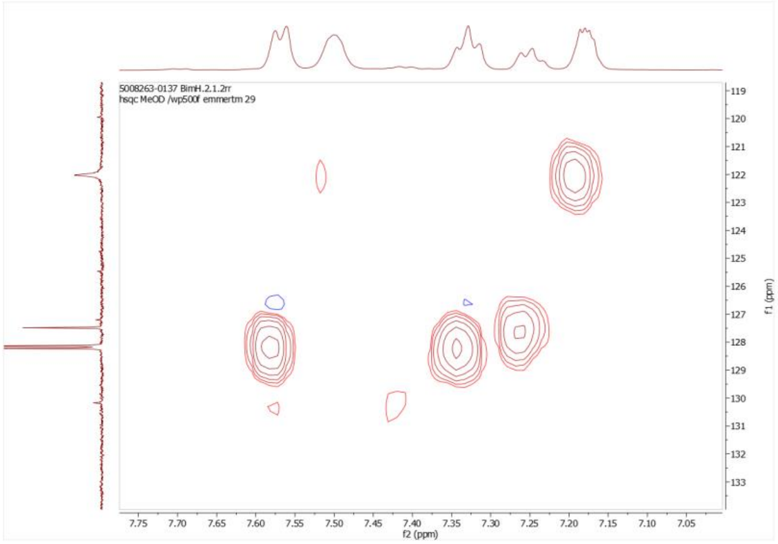
5008263-0137 BimH.2.1.2rr
hsqc MeOD /wp500f emmertm 29
f1 (ppm)
f2 (ppm)

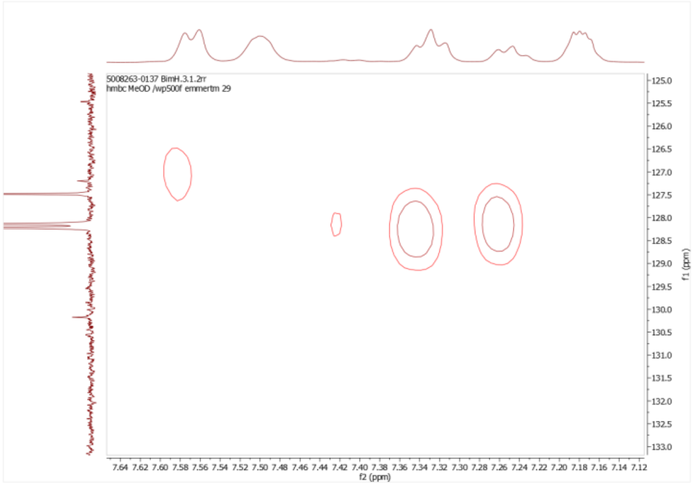
5008263-0137 BimH.3.1.2rr
hmbc MeOD /wp500f emmertm 29
7.64 7.62 7.60 7.58 7.56 7.54 7.52 7.50 7.48 7.46 7.44 7.42 7.40 7.38 7.36 7.34 7.32 7.30 7.28 7.26 7.24 7.22 7.20 7.18 7.16 7.14 7.12
f2 (ppm)
125.0 125.5 126.0 126.5 127.0 127.5 128.0 128.5 129.0 129.5 130.0 130.5 131.0 131.5 132.0 132.5 133.0
f1 (ppm)

Azoles With Low Reactivity (<2 LCAP)

The following azole structures provided only low or no reactivity, as determined by <2 LCAP of the desired 3-component coupling products.

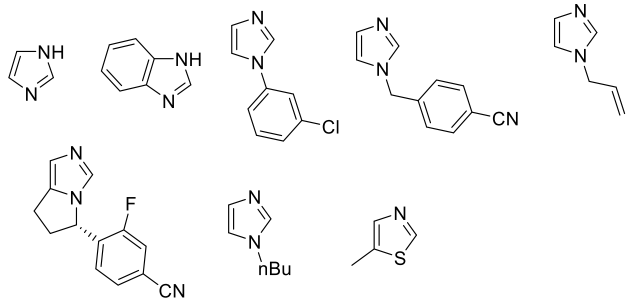

Aldehyde Scope

N-((5-Chlorobenzo[d]oxazol-2-yl)(4-fluorophenyl)methyl)-*N*-ethylethanamine

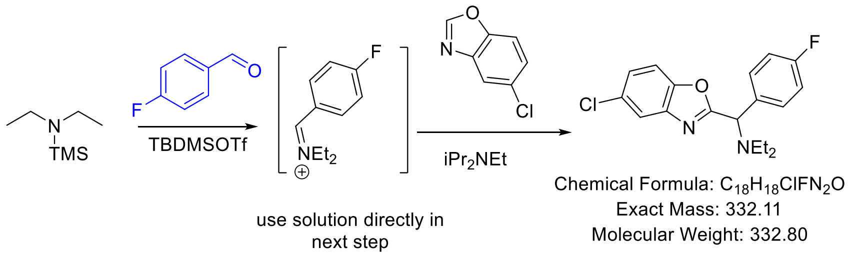

The title compound was prepared according to General Procedure 2 (reaction time 18 h).

Yield: 203 mg (61%); 62 LCAP before workup/column chromatography

^{1}H NMR (500 MHz, Methanol-d_4, ppm) δ 7.69 (s, 1H), 7.61 – 7.49 (m, 3H), 7.37 (d, *J* = 8.7 Hz, 1H), 7.15 – 7.02 (m, 2H), 5.27 (s, 1H), 2.68 (dq, *J* = 13.6, 6.8 Hz, 2H), 2.56 (dq, *J* = 13.2, 6.6 Hz, 2H), 1.03 (t, *J* = 6.9 Hz, 6H).

^{19}F NMR (470 MHz, Methanol-d_4, ppm) δ -115.95.

^{13}C NMR (126 MHz, Methanol-d_4, ppm) δ 167.4, 162.5 (d, *J* = 245.9 Hz), 149.2, 141.5, 133.4 (d, *J* = 3.2 Hz), 130.1 (d, *J* = 8.2 Hz), 129.9, 125.4, 119.2, 114.9 (d, *J* = 21.8 Hz), 111.5, 63.4, 43.4, 10.7.

HRMS: Calc. $C_{18}H_{19}ClFN_2O^+$ [M+H$^+$] 333.1165; found 333.1170.

5036622-0002-138_3.1.fid
0002-138_3_dried
h1 MeOD /wp500l feltens 51

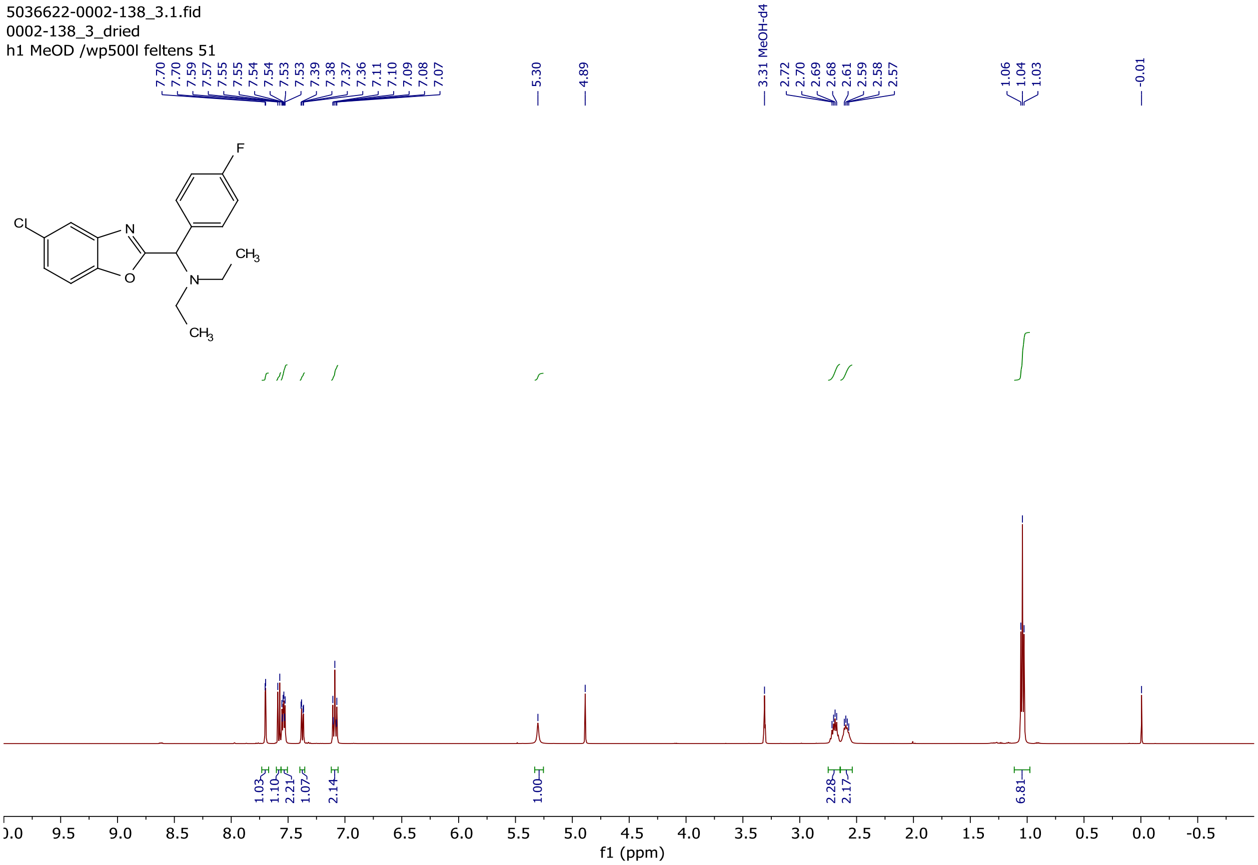

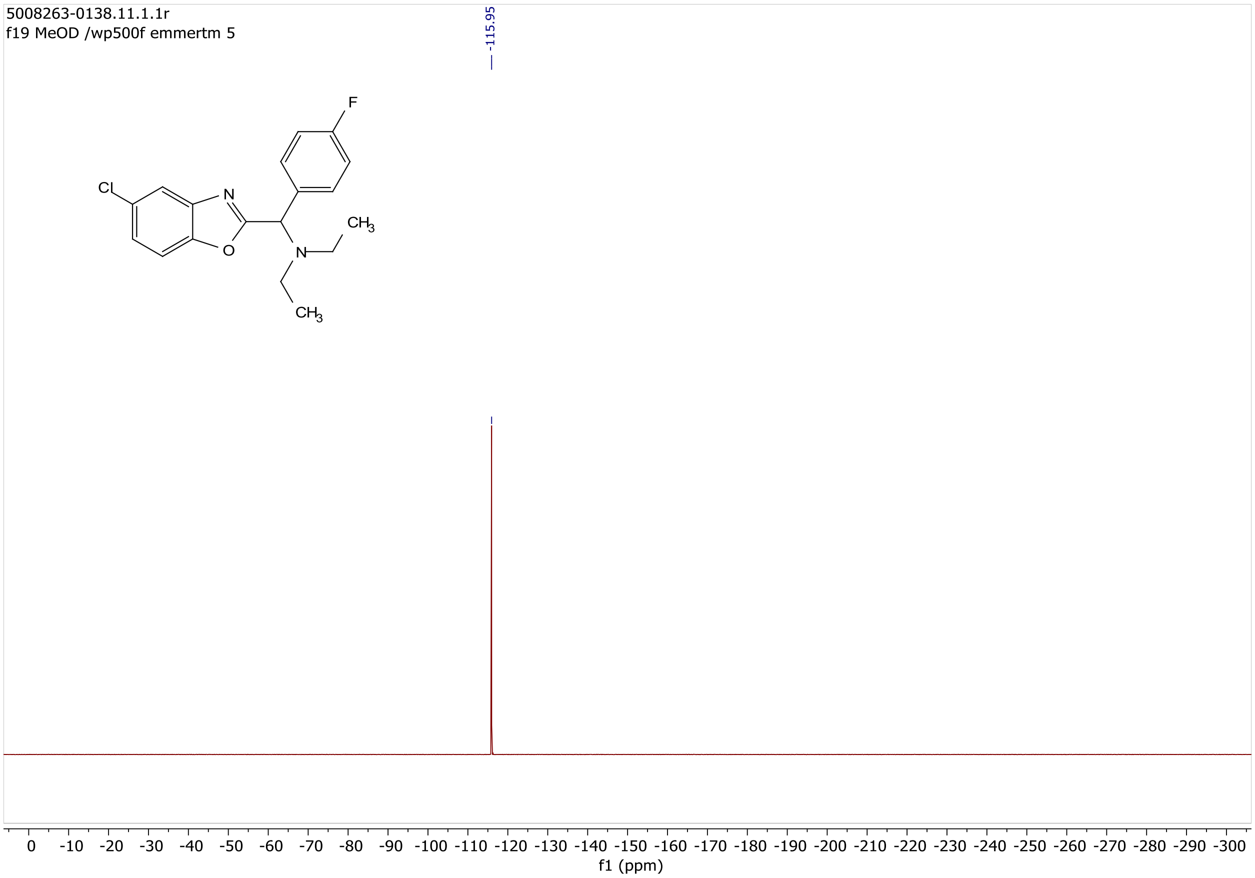
5008263-0138.11.1.1r
f19 MeOD /wp500f emmertm 5
-115.95
F
Cl
N
O
N
CH3
CH3
0 -10 -20 -30 -40 -50 -60 -70 -80 -90 -100 -110 -120 -130 -140 -150 -160 -170 -180 -190 -200 -210 -220 -230 -240 -250 -260 -270 -280 -290 -300
f1 (ppm)

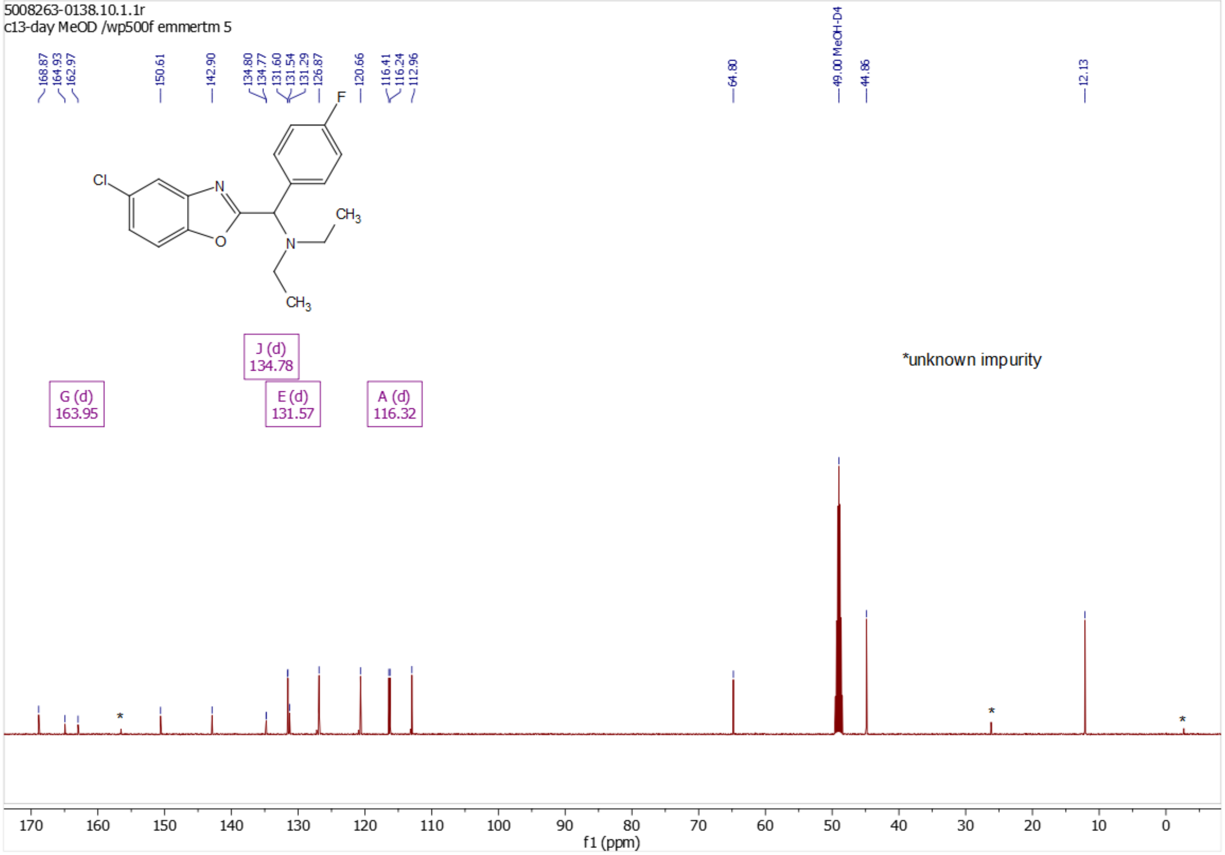
5008263-0138.10.1.1r
c13-day MeOD /wp500f emmertm 5
168.87
164.93
162.97
150.61
142.90
134.80
134.77
131.60
131.54
131.29
126.87
120.66
116.41
116.24
112.96
64.80
49.00 MeOH-D4
44.86
12.13
J (d)
134.78
G (d)
163.95
E (d)
131.57
A (d)
116.32
*unknown impurity
f1 (ppm)

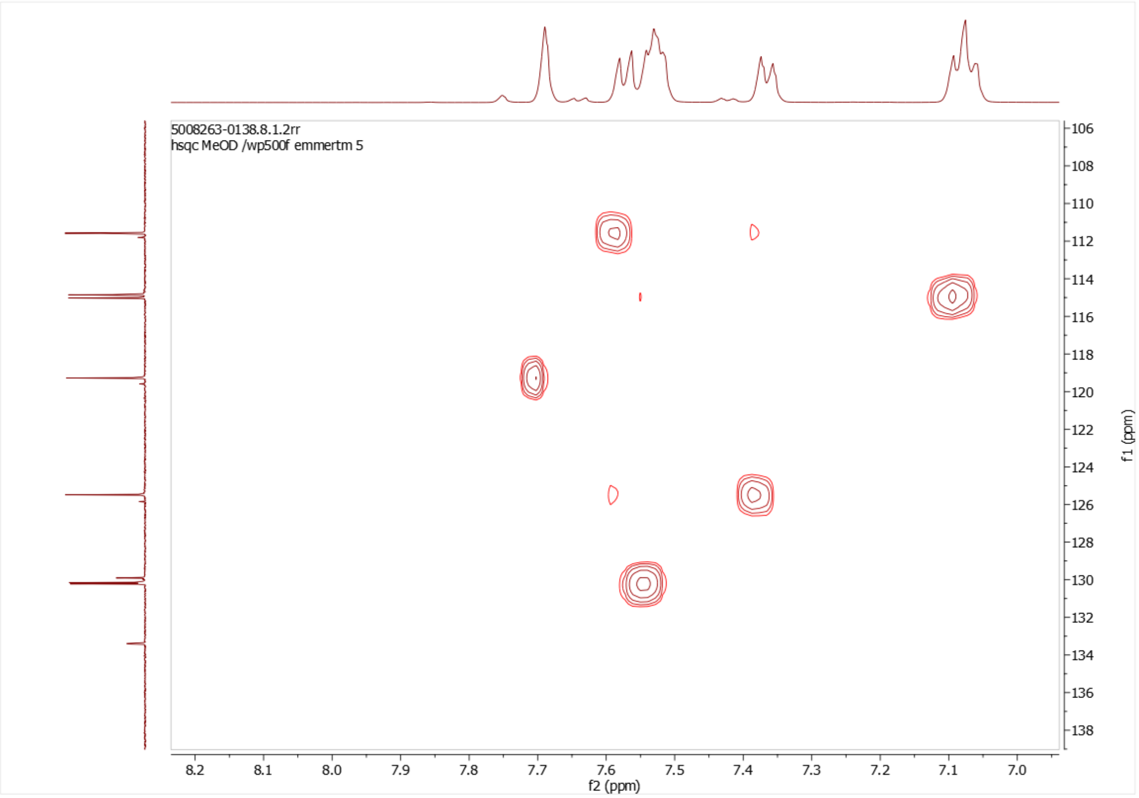
5008263-0138.8.1.2rr
hsqc MeOD /wp500f emmertm 5
f1 (ppm)
f2 (ppm)

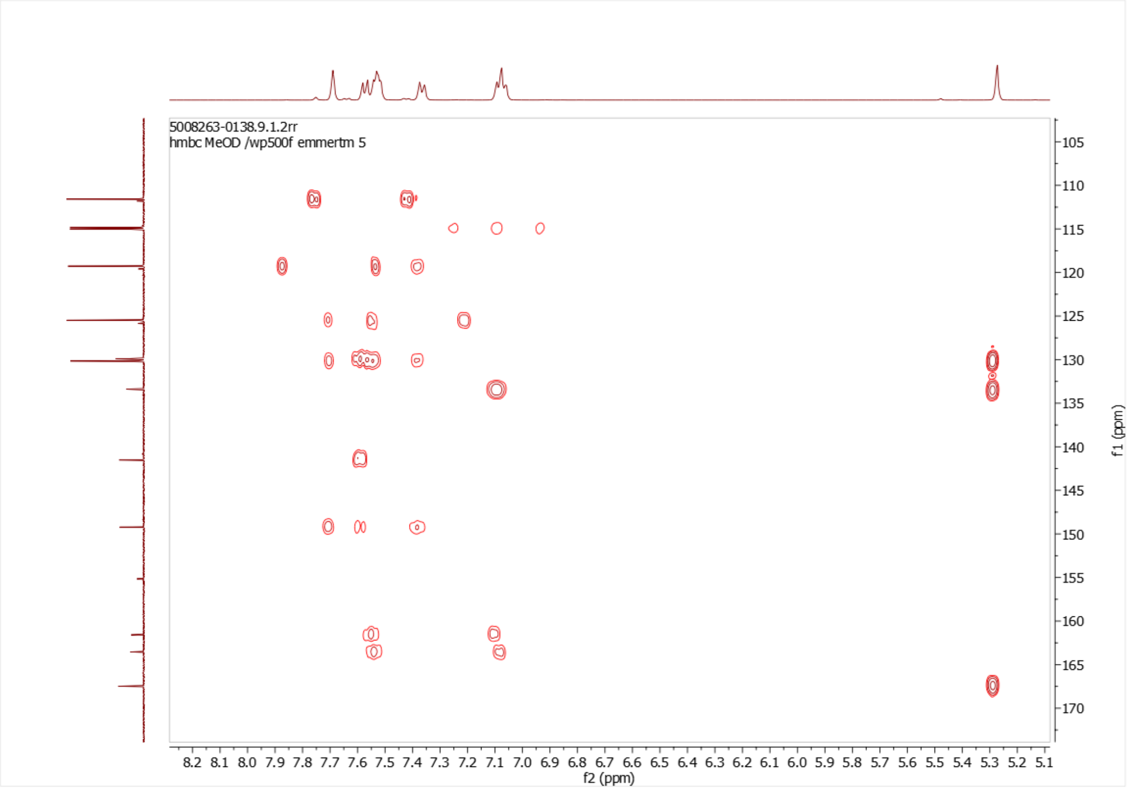
5008263-0138.9.1.2rr
hmbc MeOD /wp500f emmertm 5
f2 (ppm)
f1 (ppm)
8.2 8.1 8.0 7.9 7.8 7.7 7.6 7.5 7.4 7.3 7.2 7.1 7.0 6.9 6.8 6.7 6.6 6.5 6.4 6.3 6.2 6.1 6.0 5.9 5.8 5.7 5.6 5.5 5.4 5.3 5.2 5.1
105 110 115 120 125 130 135 140 145 150 155 160 165 170

N-((5-Chlorobenzo[d]oxazol-2-yl)(4-methoxyphenyl)methyl)-*N*-ethylethanamine

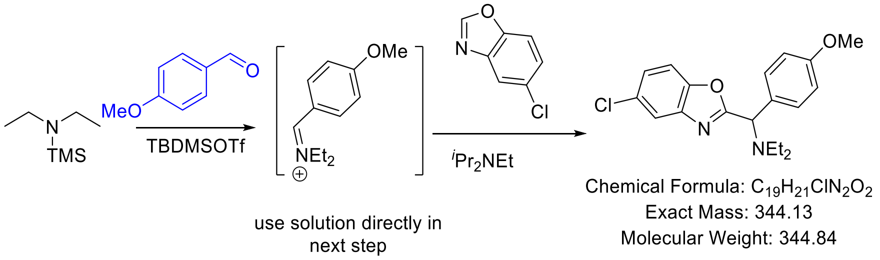

The title compound was prepared according to General Procedure 2 (reaction time: 18 h).

Yield: 122 mg (25%); 74 LCAP before workup/ Prep-HPLC

^{1}H NMR (400 MHz, Chloroform-d, ppm) δ 7.68 (d, *J* = 2.1 Hz, 1H), 7.44 (m, 3H), 7.31 – 7.27 (m, 1H), 6.91 – 6.85 (m, 2H), 5.17 (s, 1H), 3.79 (s, 3H), 2.63 (m, 4H), 1.03 (t, *J* = 7.1 Hz, 6H).

^{13}C NMR (101 MHz, Chloroform-d, ppm) δ 167.8, 159.4, 149.3, 142.0, 129.7, 129.7, 129.5, 125.1, 120.0, 113.9, 111.5, 64.1, 55.2, 43.5, 11.5.

HRMS: Calc. $C_{19}H_{21}ClN_2O_2^+$ [M+H$^+$] 345.1364; found 345.1360.

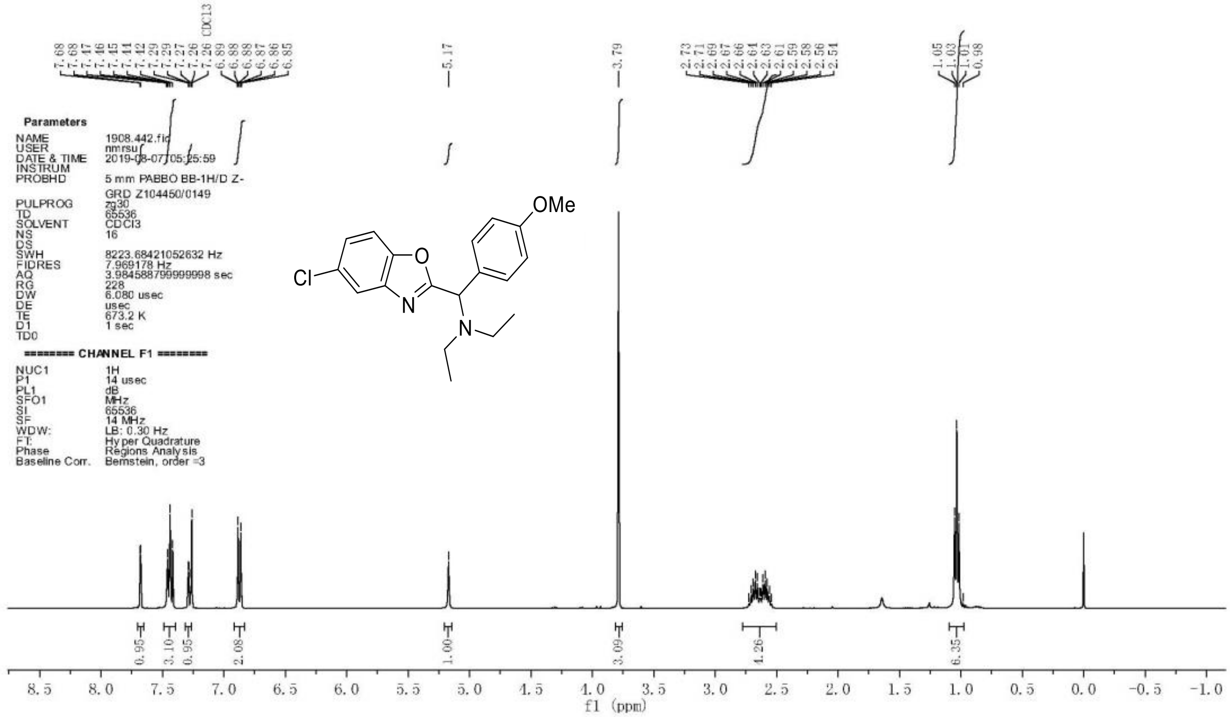

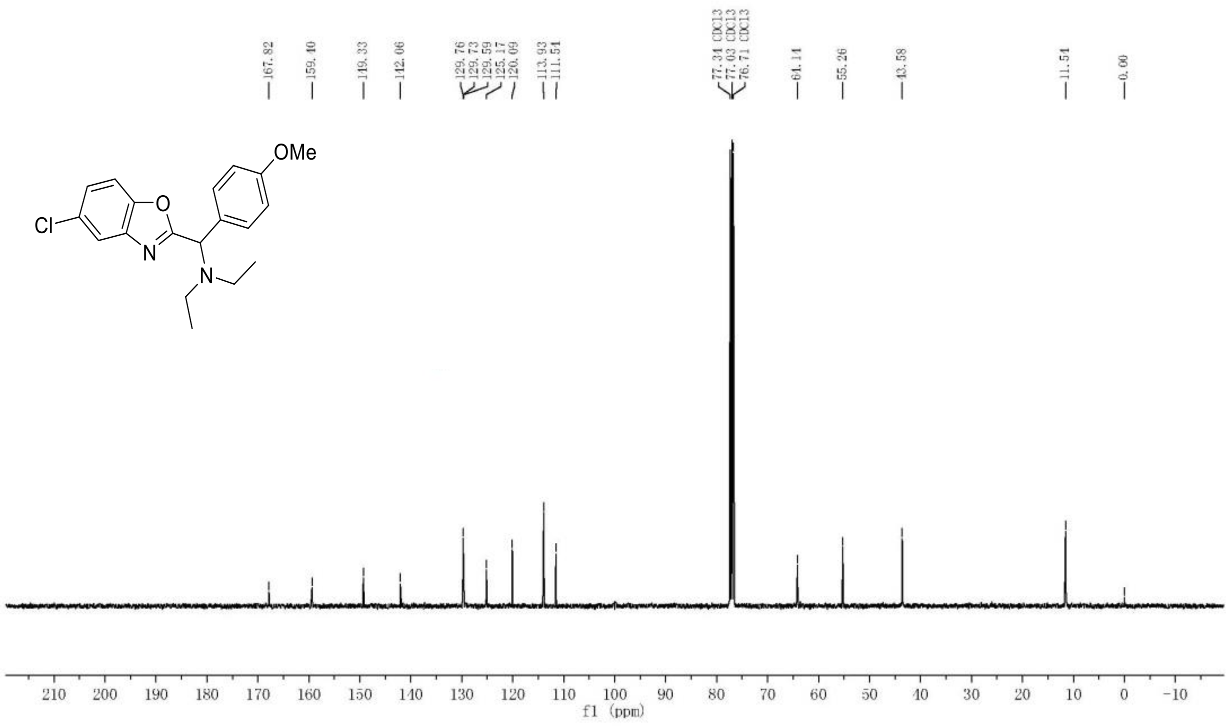
167.82
159.40
149.33
142.06
129.76
129.73
129.59
125.17
120.09
113.93
111.51
77.34 CDCl3
77.03 CDCl3
76.71 CDCl3
64.14
55.26
43.58
11.54
0.00
OMe
Cl
O
N
N
f1 (ppm)

N-((4-Bromophenyl)(5-chlorobenzo[d]oxazol-2-yl)methyl)-*N*-ethylethanamine

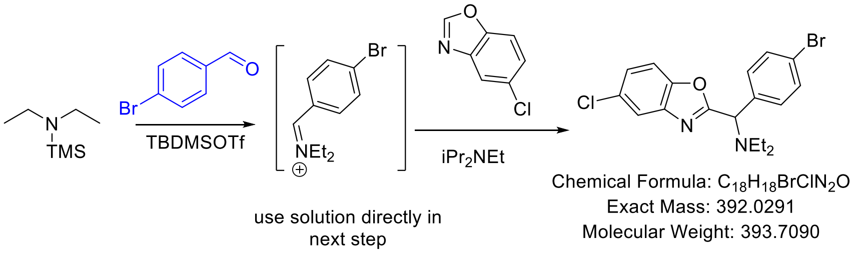

The title compound was prepared according to General Procedure 2 (reaction time: 18 h).

Yield: 280 mg (49%); 57 LCAP before workup/ Prep-HPLC

^{1}H NMR (400 MHz, Chloroform-d, ppm) δ 7.70 (d, *J* = 2.1 Hz, 1H), 7.50 – 7.45 (m, 2H), 7.45 – 7.38 (m, 3H), 7.30 (m, 1H), 5.21 (s, 1H), 2.69 (m, 2H), 2.55 (m, 2H), 1.04 (t, *J* = 7.1 Hz, 6H).

^{13}C NMR (101 MHz, Chloroform-d, ppm) δ 166.7, 149.2, 141.9, 136.8, 131.6, 130.1, 129.9, 125.4, 122.0, 120.2, 111.5, 63.8, 43.7, 11.8.

HRMS: Calc. $C_{18}H_{18}BrClN_2O^+$ [M+H$^+$] 393.0364; found 393.0359.

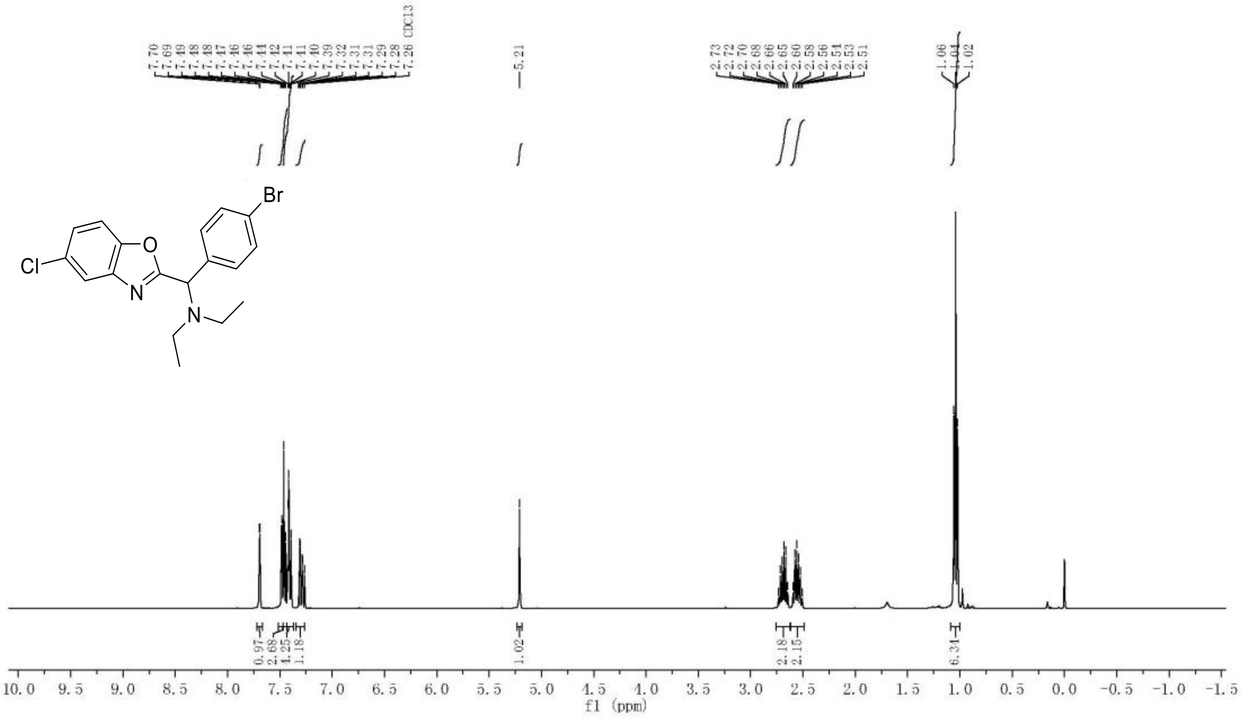

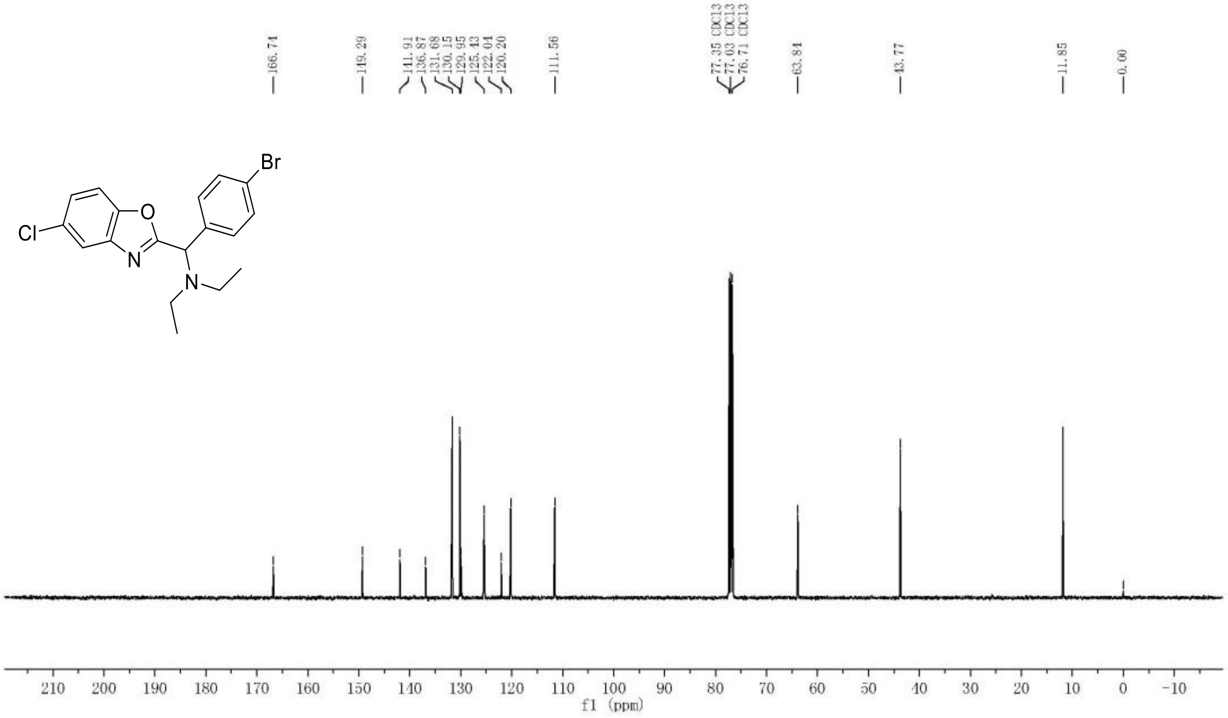
166.74
149.29
141.91
136.87
131.68
130.15
129.95
125.43
122.04
120.20
111.56
77.35 CDCl3
77.03 CDCl3
76.71 CDCl3
63.84
43.77
11.85
0.00
Br
Cl
O
N
N
210 200 190 180 170 160 150 140 130 120 110 100 90 80 70 60 50 40 30 20 10 0 -10
f1 (ppm)

N-((5-Chlorobenzo[d]oxazol-2-yl)(4-(trifluoromethyl)phenyl)methyl)-*N*-ethylethanamine

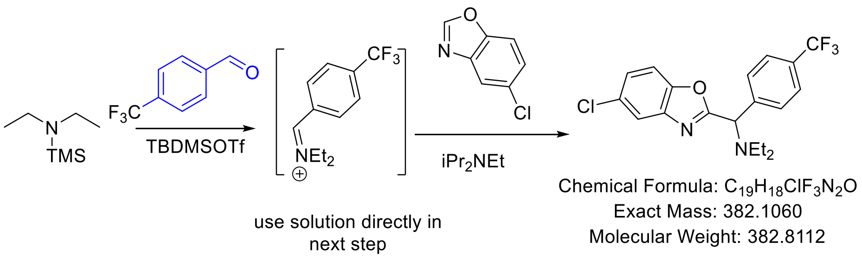

The title compound was prepared according to General Procedure 2 (reaction time: 18 h).

Yield: 189 mg (50%); 68 LCAP before workup/ Pre-HPLC

^{1}H NMR (500 MHz, Methanol-d_4, ppm) δ 7.77 – 7.63 (m, 5H), 7.61 (d, *J* = 8.7 Hz, 1H), 7.40 (dd, *J* = 8.7, 2.1 Hz, 1H), 5.44 (s, 1H), 2.72 (dq, *J* = 14.4, 7.2 Hz, 2H), 2.57 (dq, *J* = 13.9, 7.0 Hz, 2H), 1.07 (t, *J* = 7.1 Hz, 6H).

^{13}C NMR (126 MHz, Methanol-d_4, ppm) δ 168.1, 150.6, 143.6 (m), 142.9, 131.3, 131.3 (q, *J* = 32.3 Hz), 130.2, 127.0, 126.4 (q, *J* = 3.8 Hz), 125.5 (q, *J* = 271.2 Hz), 120.7, 113.0, 64.7, 45.1, 12.5.

^{19}F NMR (470 MHz, Methanol-d_4, ppm) δ -64.08.

HRMS: Calc. $C_{19}H_{18}ClF_3N_2O^+$ [M+H$^+$] 383.1133; found 383.1133.

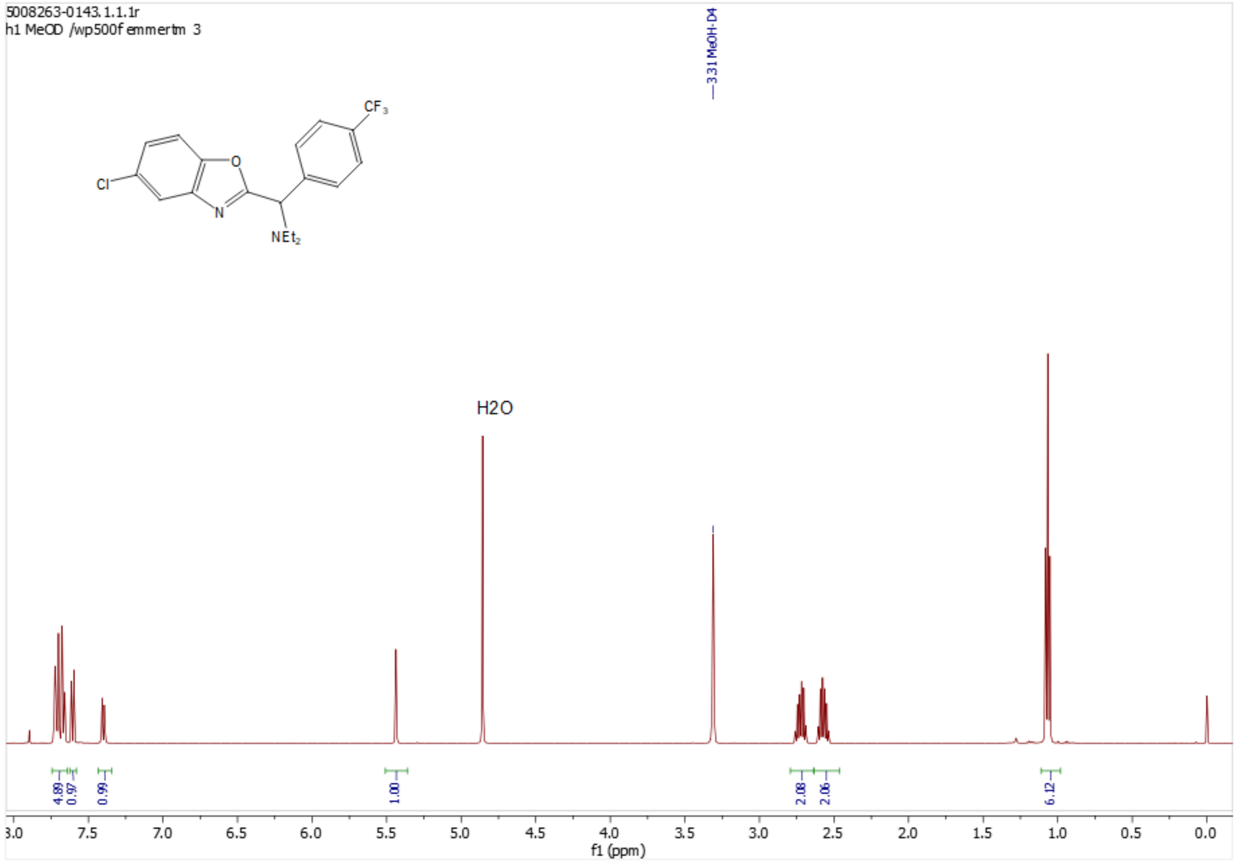

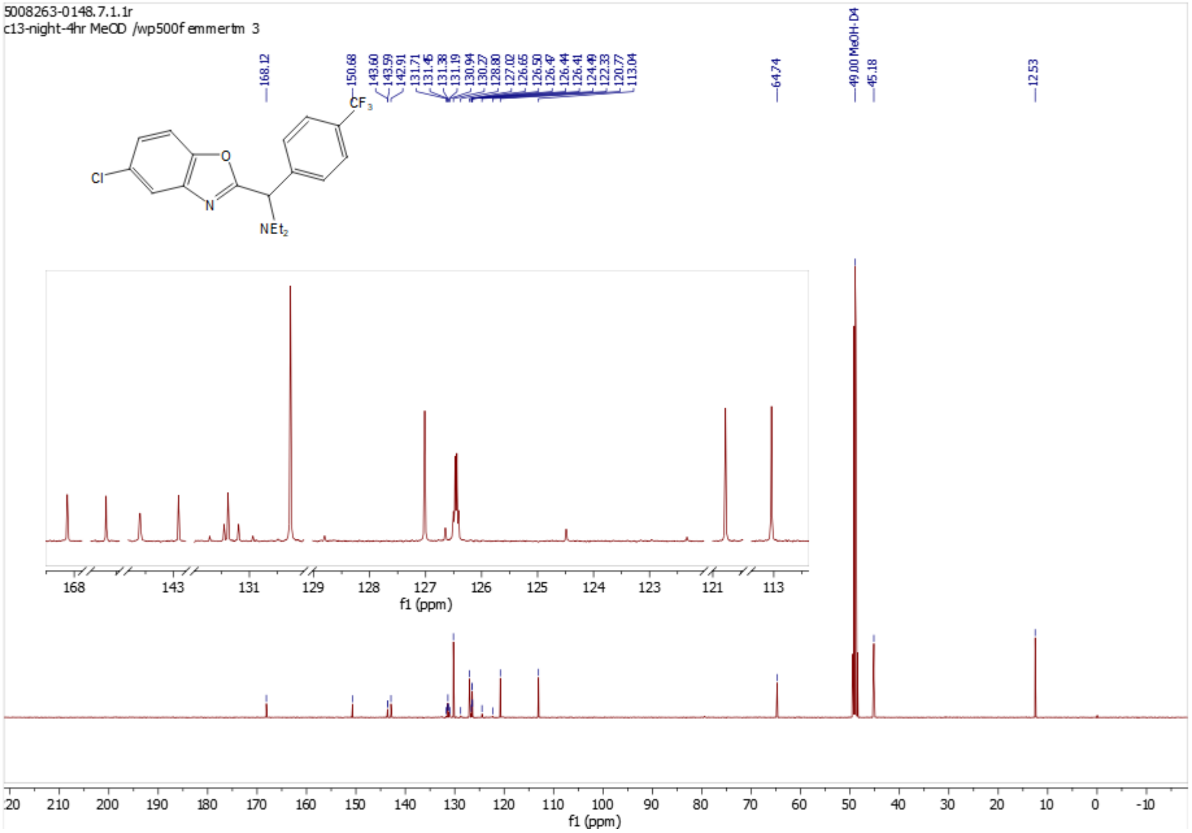
5008263-0148.7.1.1r
c13-night-4hr MeOD /wp500f emmertm 3
f1 (ppm)

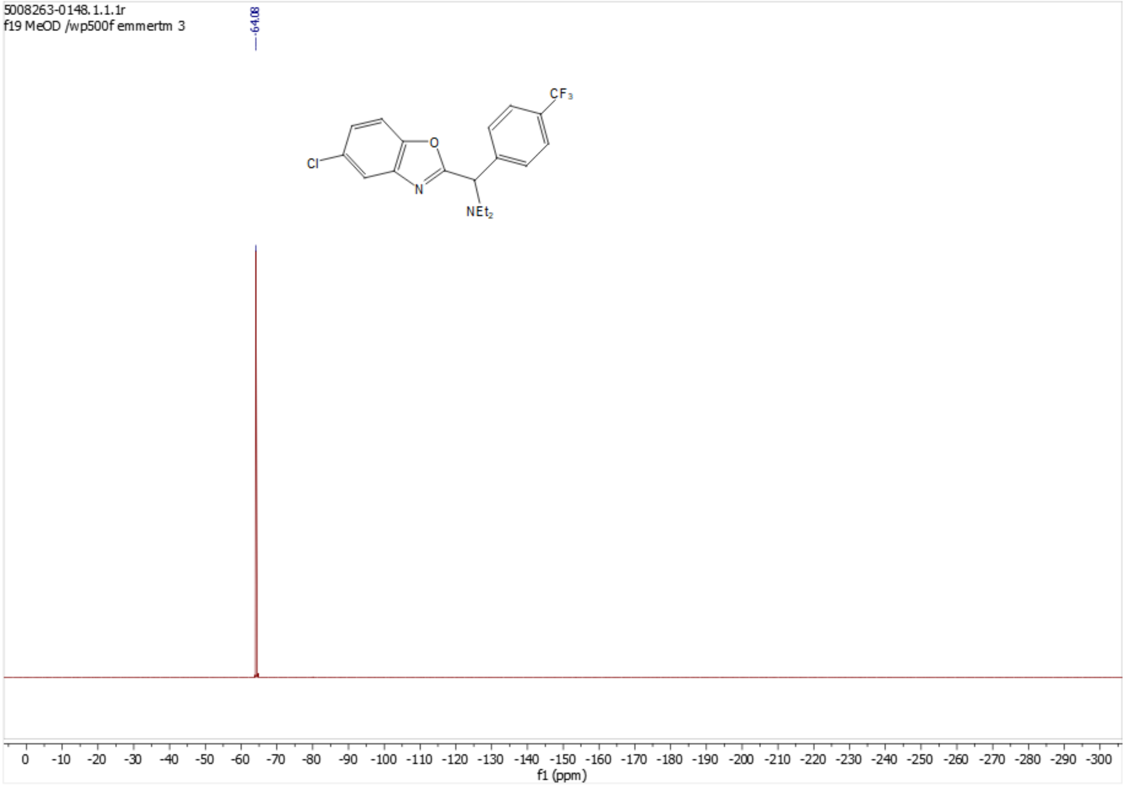
5008263-0148.1.1.1r
f19 MeOD /wp500f emmertm 3
f1 (ppm)

N-((5-Chlorobenzo[d]oxazol-2-yl)(5-methylthiophen-2-yl)methyl)-N-ethylethanamine

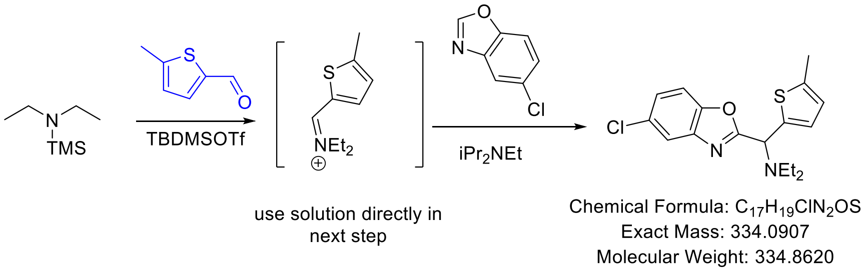

The title compound was prepared according to General Procedure 2 (reaction time: 18 h).

Yield: 120 mg (26 %); 82 LCAP before workup/ Pre-HPLC

^{1}H NMR (300 MHz, Chloroform-d, ppm) δ 7.73 (d, J = 2.1 Hz, 1H), 7.47 (d, J = 8.7 Hz, 1H), 7.32 (m, 2.1 Hz, 1H), 6.77 (m, 1H), 6.60 (m, 1H), 5.47 (s, 1H), 2.88 – 2.70 (m, 2H), 2.57 (dq, J = 13.8, 7.0 Hz, 2H), 2.47 (s, 3H), 1.10 (t, J = 7.1 Hz, 6H).

^{13}C NMR (75 MHz, Chloroform-d, ppm) δ 166.4, 149.2, 142.0, 140.4, 138.7, 129.9, 126.6, 125.4, 124.5, 120.2, 111.5, 59.7, 44.1, 15.3, 12.5.

HRMS: Calc. $C_{17}H_{19}ClN_2OS^+$ [M+H$^+$] 335.0979; found 335.0974.

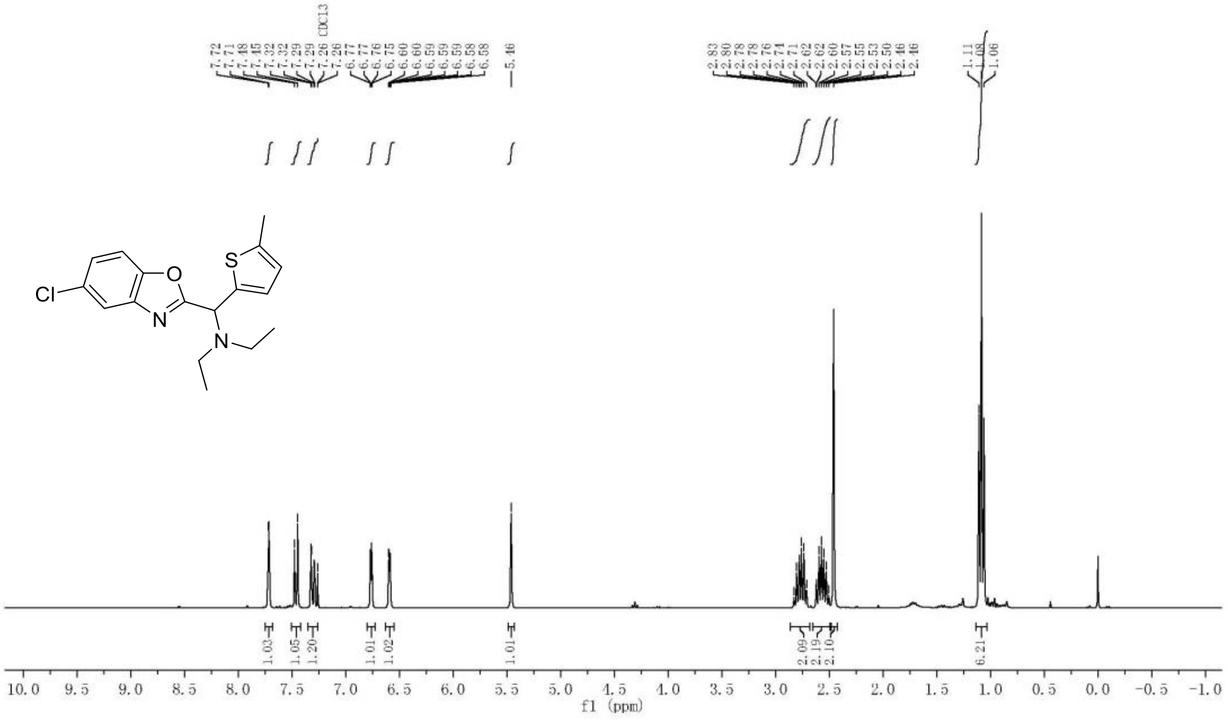

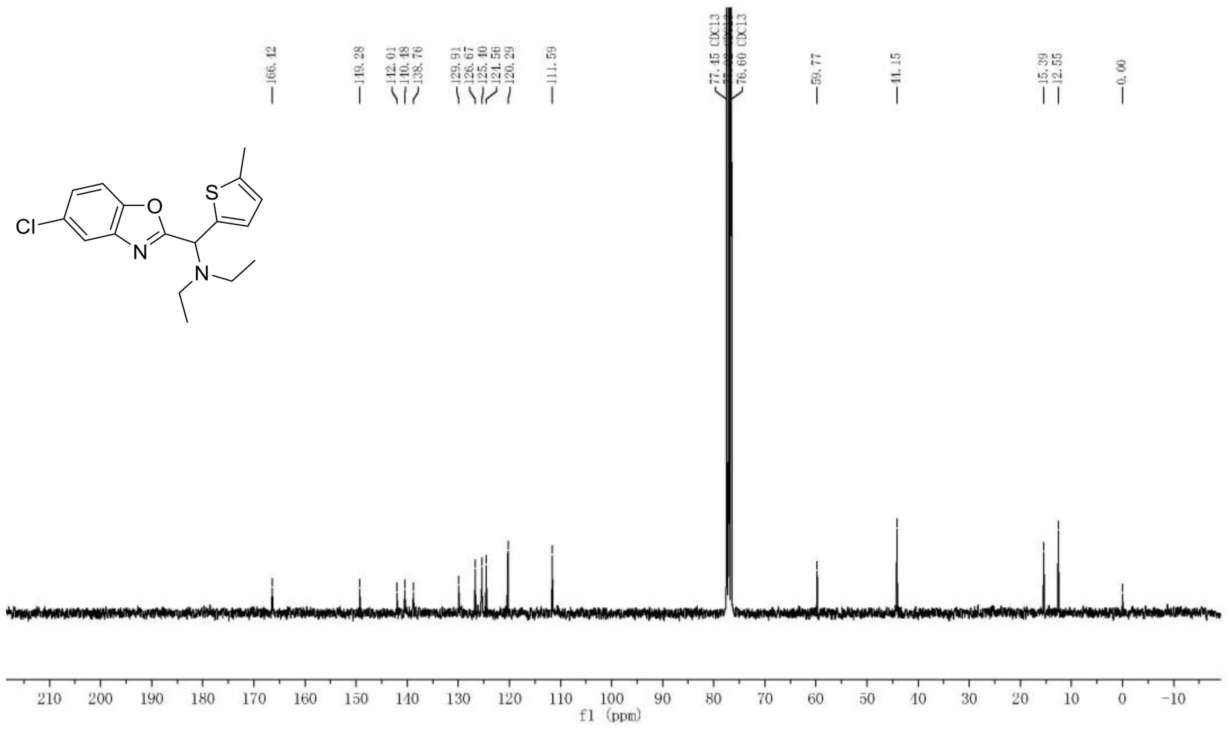

166.42
149.28
142.01
140.48
138.76
129.91
126.67
125.40
121.56
120.29
111.59
77.45 CDCl3
76.60 CDCl3
59.77
44.15
15.39
12.55
0.00
Cl
O
N
S
N
210 200 190 180 170 160 150 140 130 120 110 100 90 80 70 60 50 40 30 20 10 0 -10
f1 (ppm)

1-(5-Chlorobenzo[d]oxazol-2-yl)-*N,N*-diethyl-2,2-dimethylpropan-1-amine

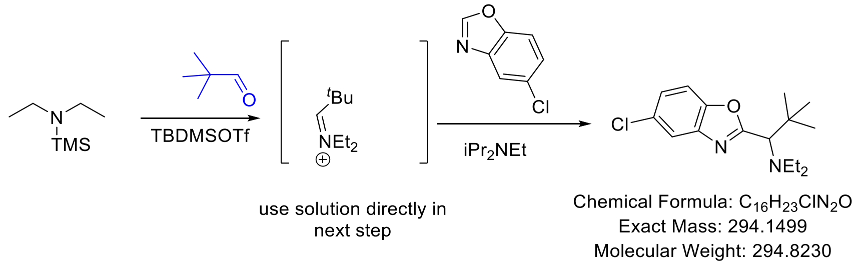

The title compound was prepared according to General Procedure 2 (reaction time: 18 h).

Yield: 53 mg (12 %); 37 LCAP before workup/ Pre-HPLC

^{1}H NMR (300 MHz, Chloroform-d, ppm) δ 7.70 (d, *J* = 2.1 Hz, 1H), 7.43 (d, *J* = 8.6 Hz, 1H), 7.31 – 7.26 (m, 1H), 3.70 (s, 1H), 2.83 (dq, *J* = 13.0, 7.4 Hz, 2H), 2.29 (dq, *J* = 13.5, 6.8 Hz, 2H), 1.09 (s, 9H), 1.04 (t, *J* = 7.1 Hz, 6H).

^{13}C NMR (75 MHz, Chloroform-d, ppm) δ 167.4, 148.7, 141.9, 129.5, 124.8, 120.0, 111.1, 67.5, 46.7, 35.4, 27.6, 14.0.

HRMS: Calc. $C_{15}H_{21}ClN_2O^+$ [M+H$^+$] 295.1572; found 295.1569.

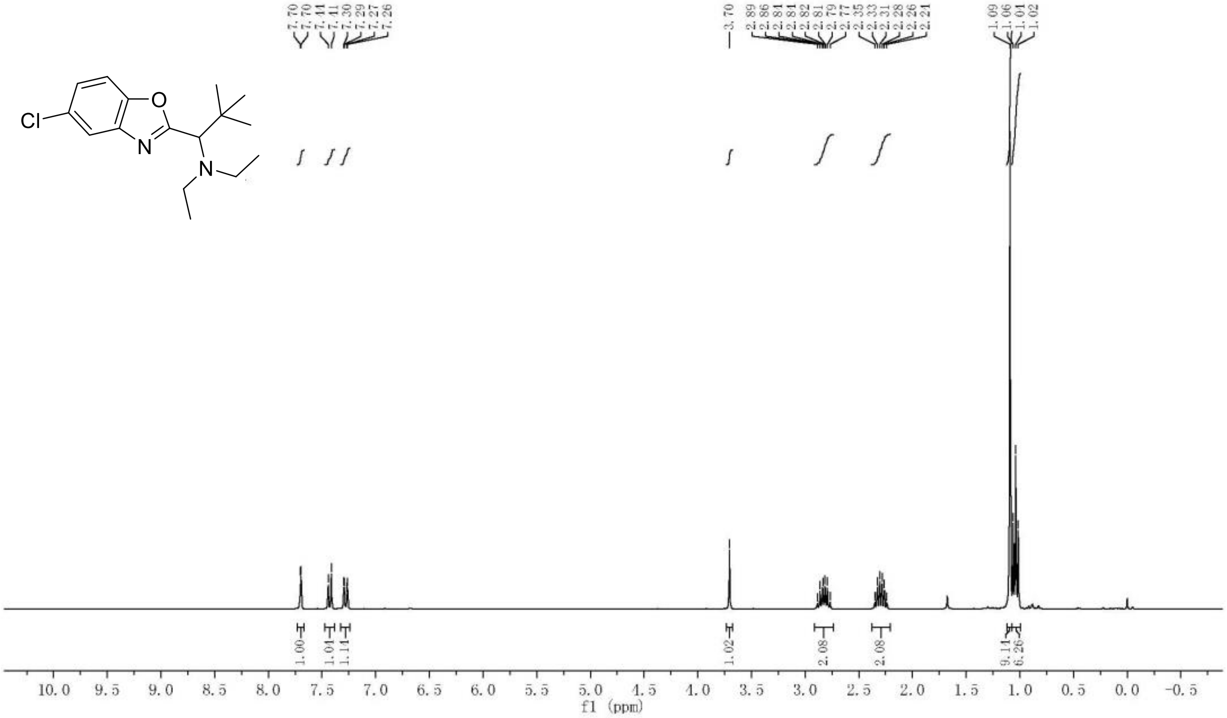

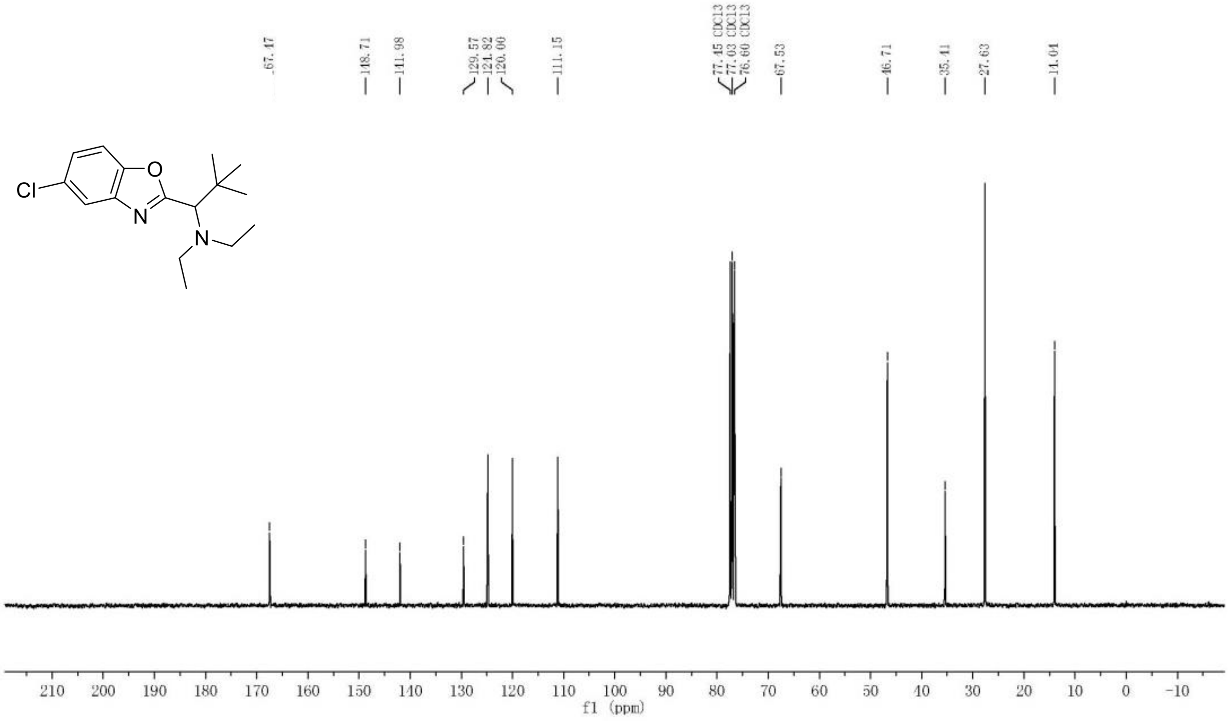

67.47
148.71
141.98
129.57
124.82
120.00
111.15
77.45 CDCl3
77.03 CDCl3
76.60 CDCl3
67.53
46.71
35.41
27.63
14.04
Cl
O
N
N
210
200
190
180
170
160
150
140
130
120
110
100
90
80
70
60
50
40
30
20
10
0
-10
f1 (ppm)

Aldehydes showing no product formation under conditions of General Procedure 2

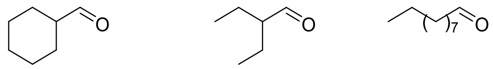

Experimental data supporting enamine formation for aliphatic aldehydes with acidic β-C-H bonds

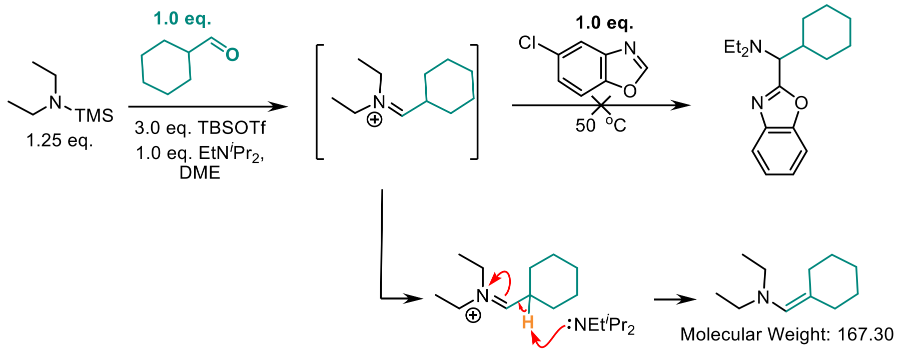

Scheme 3. Proposed Mechanism of Enamine Formation

In a glovebox, cyclohexanecarbaldehyde (0.121 mL, 1.00 mmol, 1.00 euqiv.) was reacted with *N,N*-diethyl-1,1,1-trimethylsilanamine (0.237 mL, 1.25 mmol, 1.25 equiv.), and *tert*-butyldimethylsilyl trifluoromethanesulfonate (0.69 mL, 3.00 mmol, 3.00 equiv.) in DME (1.5 mL) for 15 min. The resulting solution was added to a vial containing a mixture of 5-chlorobenzoxazole (0.154 g, 1.00 mmol, 1.00 equiv.) and *N*-ethyl-*N*-isopropylpropan-2-amine (0.174 mL, 1.00 mmol, 1.00 equiv.) in DME (1.5 mL). The reaction vial was sealed, removed from the glovebox, and heated on a hotplate to 50 °C for 48 h. After that time, the crude reaction mixture was cooled to room temperature and concentrated in vacuum (rotary evaporator). 0.6 mL $CDCl_3$ was added and the resulting mixture was analyzed by 1H NMR. 100 uL of the solution in CDCl3 was then dilution with 300 uL MeCN and analyzed by UPLC/MS. See below for data and analysis.

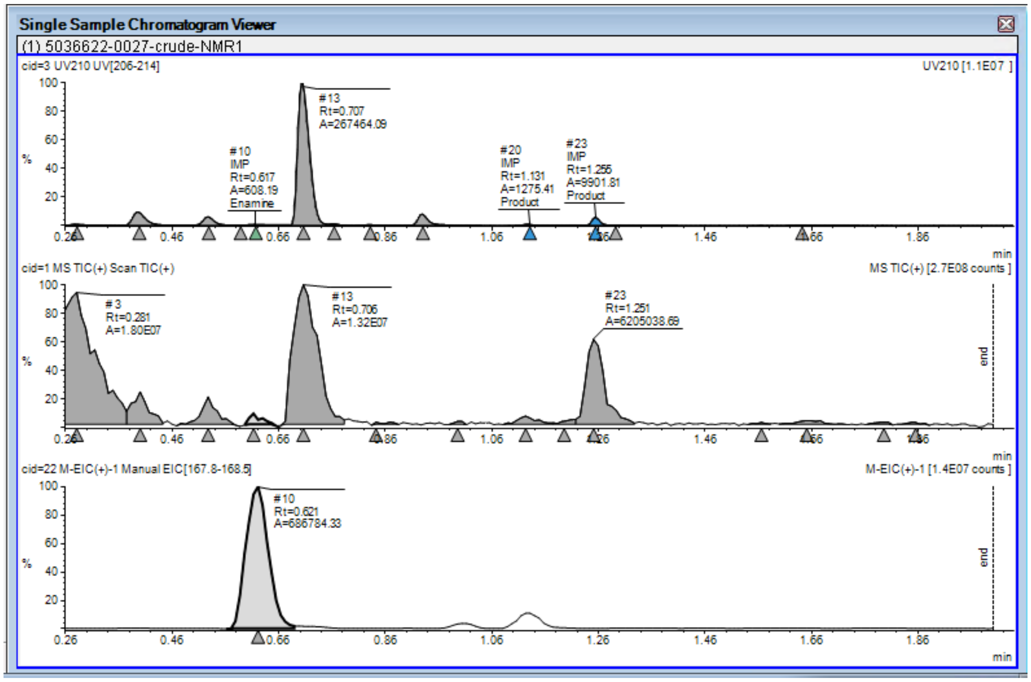

Figure 5. Analysis of crude reaction mixture. A peak at 0.617 min (0.2 LCAP in the 210 nm chromatogram) likely represents the proposed enamine product, as confirmed by massspectroscopic analysis (M+H 168; see TIC spectrum in the second row). The third row shows a mass extracted chromatogram for m/z 168. However, due to the lack of chromophore in the enamine structure, the peak intensity is low in the UV chromatogram.

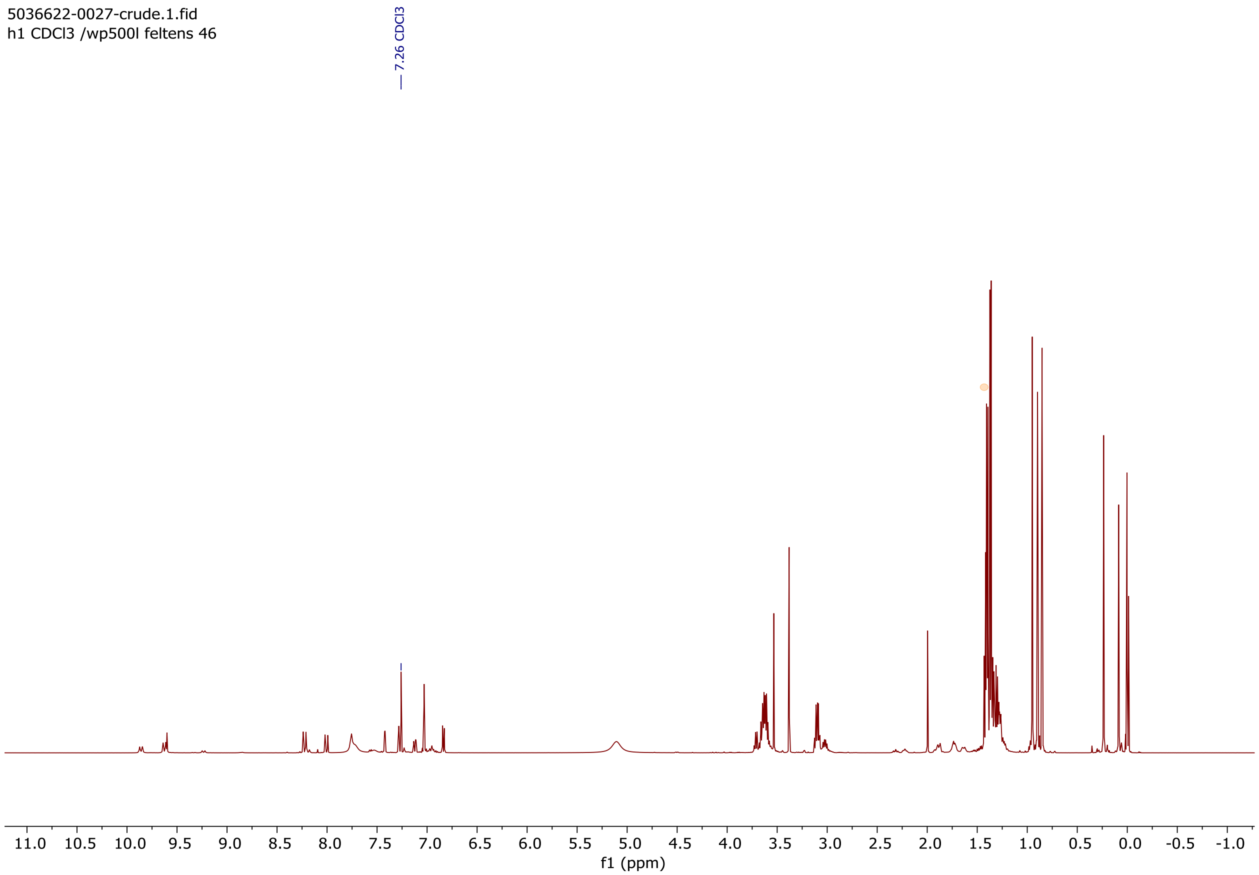

Figure 6. 1H NMR Spectrum of the crude reaction mixture. The broad peak at 5.11 ppm is characteristic for the enamine product;[1] all the other enamine peaks likely overlap with peaks of other reactants.

[1] A. D. Dilman, P. A. Belyakov, M. I. Struchkova, D. E. Arkhipov, A. A. Korlyukov and V. A. Tartakovsky, *The Journal of Organic Chemistry*, 2010, **75**, 5367-5370.

Experiments in support of proposed mechanism: Observation of azole-TBS adduct and H/D exchange

In the glovebox, an oven dried 4 mL vial equipped with a stirbar was charged with 5-chlorobenzoxazole (80 mg, 0.520 mmol) and DME (1.6 mL). 100 uL of this solution was diluted with 300 uL MeCN and then analyzed by UPLC/MS.

tert-Butyldimethylsilyl trifluoromethanesulfonate (0.120 mL, 0.520 mmol) was added to the solution, which was stirred for 5 min. 100 uL of the solution was diluted with 300 uL MeCN and analyzed by UPLC/MS.

Then, *N*-ethyl-*N*-isopropylpropan-2-amine (0.190 mL, 1.094 mmol) and trifluoromethanesulfonic acid-d_1 (0.046uL, 0.520 mmol) were added. The reaction was stirred at room temperature. After 2 h, 100 uL of the solution was diluted with 300 uL MeCN and analyzed by UPLC/MS.

KF (80 mg, 1.4 mmol) was added to convert any silylated azole back to the starting material and the mixture was heated to 50 C for 5 h. 100 uL of the resulting mixture was diluted with 300 uL MeCN and analyzed by UPLC/MS.

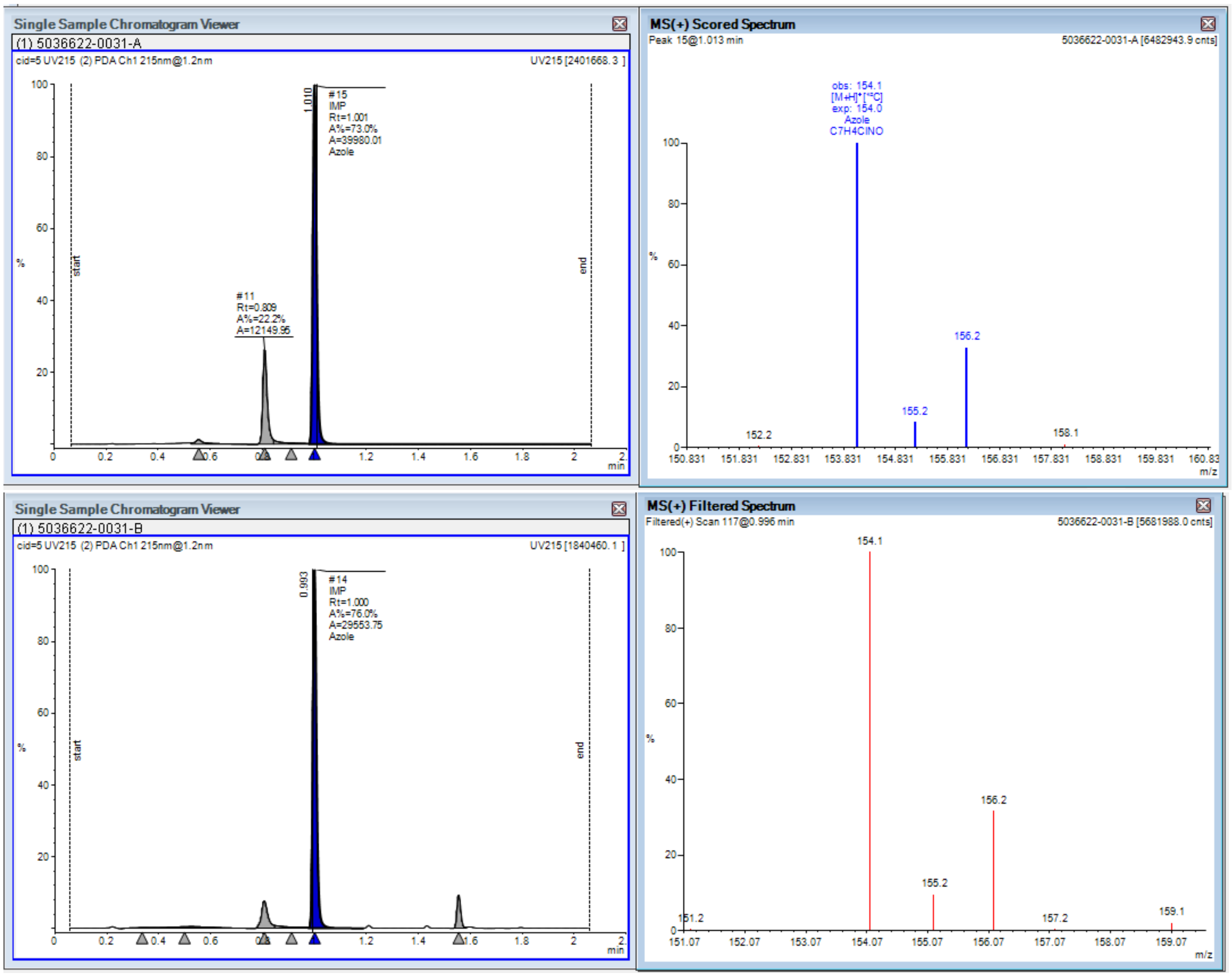

Figure 7. Left side: UPLCMS chromatograms of 4-chlorobenzoxazole in DME (top) and 4-chlorobenzoxazole + TBSOTf in DME. Right side: mass spectra of 4-chlorobenzoxazole peak at 1.0 min.

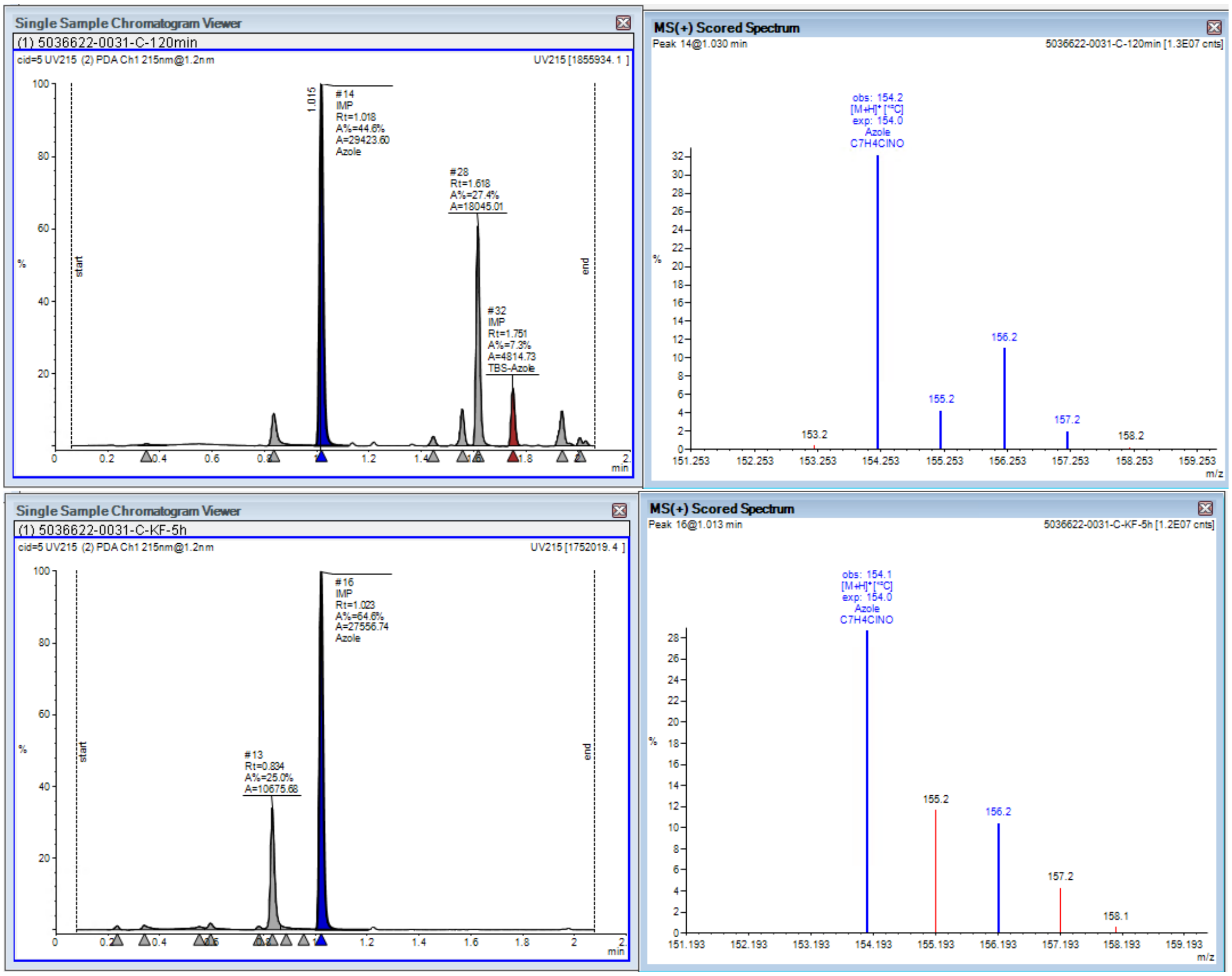

Figure 8. Left side: UPLCMS chromatograms of 4-chlorobenzoxazole + TBSOTf + TfOH-D1 + $^{i}Pr_2NEt$ in DME after 2 h (top) and after quenching with KF (bottom). Right side: mass spectra of 4-chlorobenzoxazole peak at 1.0 min showing an increase in the M+2 and M+4 peaks due to deuterium incorporation.

Table 1. Detailed H/D exchange results.

m/z	Calculated m/z abundance azole [M+H]	Measured azole starting material	Measured Azole + TBSOTf	Measured Azole + TBSOTf + TfOD + iPr2NEt (2 h, RT)	Measured reaction mixture + KF (5 h @ 50 °C)
154	100%	100%	100%	100%	100%
155	7.6%	8%	10%	13%	41%
156	32.0%	33%	39%	34%	36%
157	2.4%	0%	2%	6%	15%

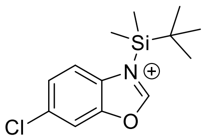

m/z: 268.09 (100.0%), 270.09 (32.0%),
269.10 (14.1%), 269.09 (5.1%), 271.09
(4.5%), 270.09 (3.3%), 271.09 (1.6%),
272.09 (1.1%)

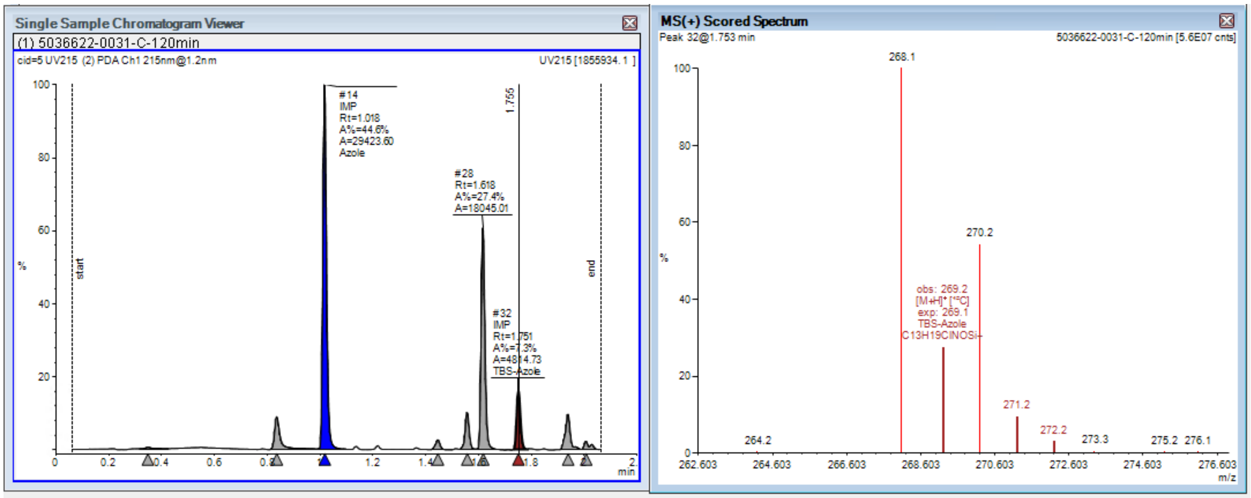

Figure 9. Mass Spectrum showing TBS-azole adduct m/z 268 at 1.75 min and predicted mass distribution for TBS-azole adduct.

DFT Results

Proposed Mechanism

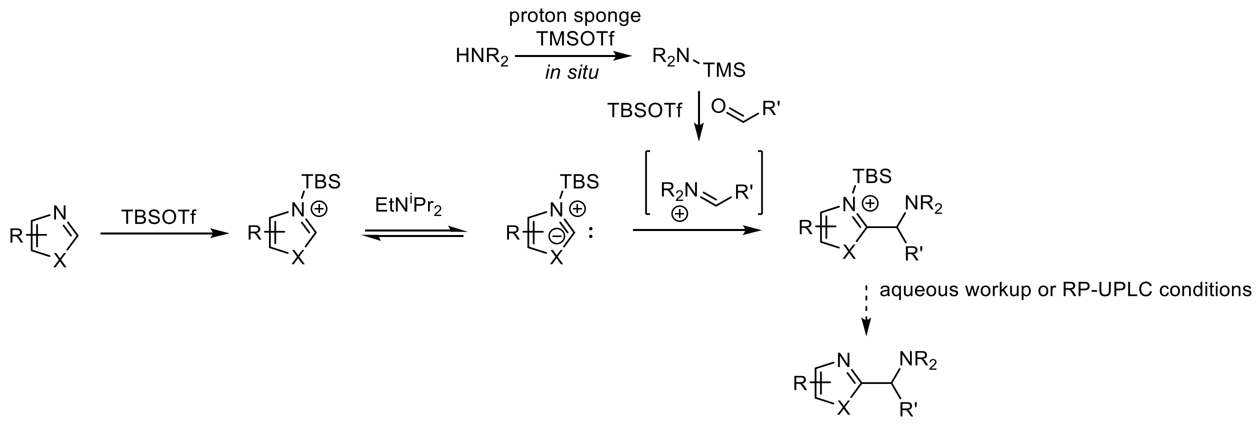

Scheme 4. Proposed Mechanism

Computational Methods

All density functional theory (DFT) calculations were performed using Gaussian 16[2]. Geometry optimizations and frequency calculations were performed at the M06-2X/6-31G(d) level of theory[3,4], with the SMD model[5] to account for solvation effects. Normal vibrational mode analysis confirmed the optimize structures are minima or transition structures. Transition structures are verified by intrinsic reaction coordinate (IRC) calculations. Truhlar's quasiharmonic correction was used to compute molecular entropies to reduce error caused by the breakdown of the harmonic oscillator approximation, by setting all positive frequencies that are less than 100 cm−1 to 100 cm−1.[6] M06-2X/def2TZVPP single-point energies were computed on the M06-2X-optimized structures. MacroModel[7] was used to perform conformational searches with the OPLS3e force field.[8] 3D renderings of stationary points were generated using CYLview 1.0[9] and PyMOL 2.3[10].

---

[2] Gaussian 16, Revision C.01, Frisch, M. J.; Trucks, G. W.; Schlegel, H. B.; Scuseria, G. E.; Robb, M. A.; Cheeseman, J. R.; Scalmani, G.; Barone, V.; Petersson, G. A.; Nakatsuji, H.; Li, X.; Caricato, M.; Marenich, A. V.; Bloino, J.; Janesko, B. G.; Gomperts, R.; Mennucci, B.; Hratchian, H. P.; Ortiz, J. V.; Izmaylov, A. F.; Sonnenberg, J. L.; Williams-Young, D.; Ding, F.; Lipparini, F.; Egidi, F.; Goings, J.; Peng, B.; Petrone, A.; Henderson, T.; Ranasinghe, D.; Zakrzewski, V. G.; Gao, J.; Rega, N.; Zheng, G.; Liang, W.; Hada, M.; Ehara, M.; Toyota, K.; Fukuda, R.; Hasegawa, J.; Ishida, M.; Nakajima, T.; Honda, Y.; Kitao, O.; Nakai, H.; Vreven, T.; Throssell, K.; Montgomery, J. A., Jr.; Peralta, J. E.; Ogliaro, F.; Bearpark, M. J.; Heyd, J. J.; Brothers, E. N.; Kudin, K. N.; Staroverov, V. N.; Keith, T. A.; Kobayashi, R.; Normand, J.; Raghavachari, K.; Rendell, A. P.; Burant, J. C.; Iyengar, S. S.; Tomasi, J.; Cossi, M.; Millam, J. M.; Klene, M.; Adamo, C.; Cammi, R.; Ochterski, J. W.; Martin, R. L.; Morokuma, K.; Farkas, O.; Foresman, J. B.; Fox, D. J. Gaussian, Inc., Wallingford CT, 2016.

[3] Zhao, Y.; Truhlar, D. G. Theor. Chem. Acc. 2008, 120, 215

[4] Zhao, Y.; Truhlar, D. G. Acc. Chem. Res. 2008, 41, 157

[5] Marenich, A. V.; Cramer, C. J.; Truhlar, D. G. J. Phys. Chem. B 2009, 113, 6378

[6] Zhao, Y.; Truhlar, D. G. Phys. Chem. Chem. Phys. 2008, 10, 2813

[7] Schrödinger Release 2019-3: MacroModel, Schrödinger, LLC, New York, NY, 2019.

[8] Roos, K.; Wu, C.; Damm, W.; Reboul, M.; Stevenson, J.M.; Lu, C.; Dahlgren, M.K.; Mondal, S.; Chen, W.; Wang, L.; Abel, R.; Friesner, R.A.; Harder, E.D., J. Chem. Theory Comput., 2019, 1863–1874

[9] Legault, C. Y. CYLview, version 1.0b; Université de Sherbrooke: Quebec, Canada, 2009; http://www.cylview.org.

[10] The PyMOL Molecular Graphics System, Version 2.0 Schrödinger, LLC.

Calculated Transition States for C-C Bond Formation Step

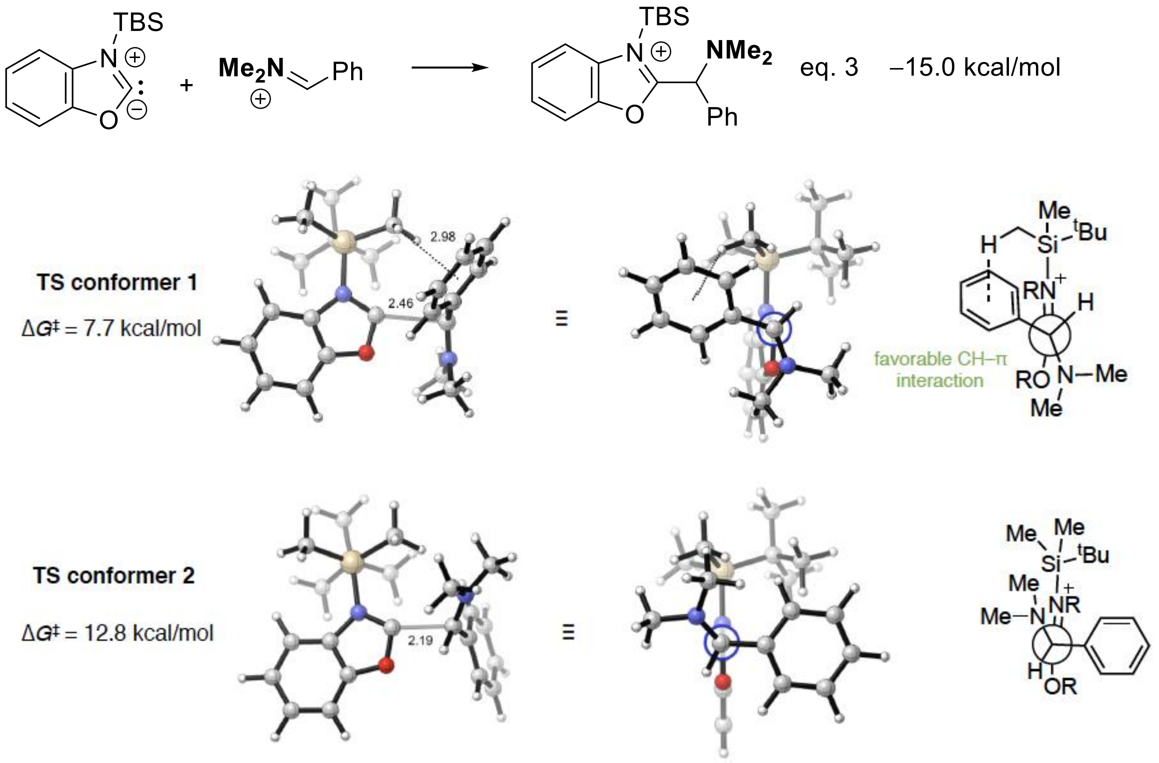

*Figure 10. Important TS conformations about the C-C forming bond between **3** and the truncated N-benzylidene-N-methylmethanaminium.*

*Table 2. Key C-C bond forming distances and angles of the lowest energy transition state structures for 9 iminium electrophiles reacting with **3**.*

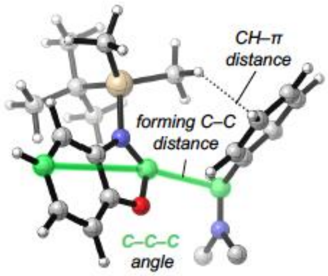

TS	ΔG‡ (kcal/mol)	C-C_distance (Å)	CH-π_distance (Å)	C-C-C_angle
**Cy2NH**	20.3	2.48	2.89	160.9
**CyNH2**	10.9	2.53	3.21	151.5
**pyrrolidine**	9.9	2.46	2.79	137.4
**piperazine**	9.8	2.47	2.87	159.6
**azetidine**	9.1	2.42	2.92	157.1
**azepane**	8.7	2.41	2.81	157.8
**morpholine**	8.5	2.48	2.91	156.3
**Me2NH**	7.7	2.46	2.98	156.3
**Et2NH**	7.6	2.45	2.79	159.4

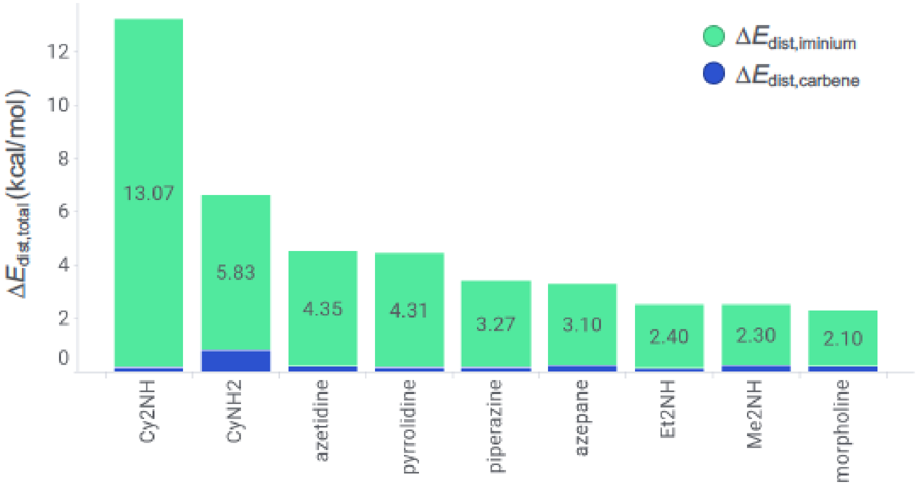

*Figure 11. Distortion energies of silylated carbene **3** and 9 iminium electrophiles going from reactant to transition state geometries.*

Insights into Azole Reactivity: Calculated Deprotonation Equilibria with Successful and Unsuccessful Substrates

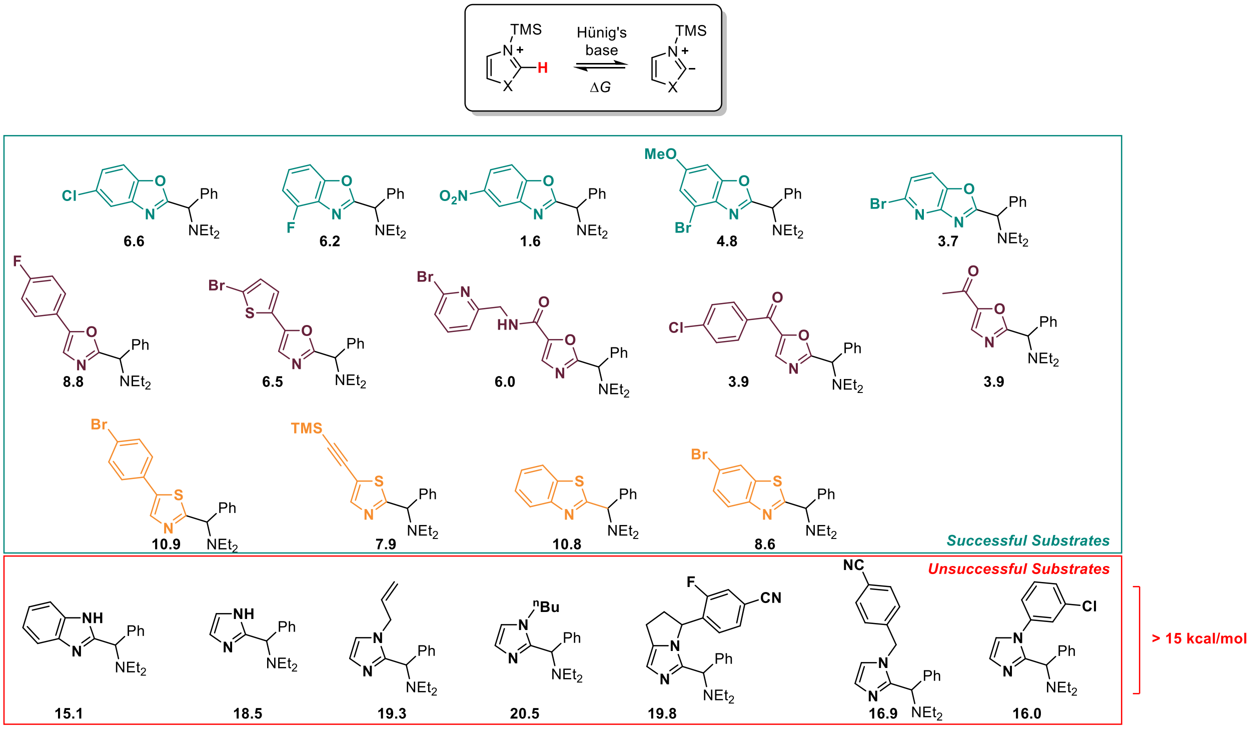

Figure 12. Calculated Free Enthalpies ΔG for Deprotonation of the Parent Azole Substrate for the Azole Substrate Scope. Desired products are shown. All numbers under the desired product are shown in kcal/mol.

Cartesian Coordinates for Transition State Structures in the Amine Scope

azepane_TS

C	2.14353	4.10748	1.82519
C	3.94835	-1.30921	1.07620
C	1.42316	-2.57741	-0.14392
C	3.29049	-0.83493	-1.95086
Si	2.60891	-1.13958	-0.21811
C	0.91163	3.62687	1.39041
C	3.30644	3.32694	1.73834
C	3.28842	2.03592	1.21875
O	-0.13534	1.61515	0.37140
C	0.91701	2.34072	0.87743
C	2.05385	1.55170	0.79097
N	1.62017	0.33737	0.21942
C	-1.49389	-1.31144	2.00749
C	-1.41216	-2.31093	2.96976
C	-1.63118	-3.64319	2.61641
C	-1.92304	-3.97834	1.29649
C	-1.98857	-2.98266	0.32649
C	0.30246	0.39756	-0.03146
C	-1.79002	-1.64509	0.68099
C	-1.83530	-0.65173	-0.40865
H	2.20664	5.10959	2.23612
H	4.24755	3.74198	2.08357
H	4.19495	1.44544	1.15124
H	-1.29751	-0.27740	2.27706
H	-1.17157	-2.05203	3.99585
H	-1.56569	-4.42058	3.37134
H	-2.08579	-5.01455	1.01838
H	-2.19271	-3.24220	-0.70928
H	-1.45834	-0.97664	-1.37397
H	4.26922	-2.35687	1.11114
H	3.57995	-1.04675	2.07392
H	4.83544	-0.70451	0.86498
H	0.99124	-2.67897	0.85847
H	0.60244	-2.47504	-0.85963
H	1.95740	-3.50716	-0.37296
C	2.14411	-0.81585	-2.97048
C	4.26045	-1.97785	-2.28970
C	4.04137	0.50234	-2.00512
H	0.00391	4.21735	1.44322
N	-2.56031	0.43030	-0.42737
C	-3.30939	0.94148	0.73367
C	-4.60629	1.64223	0.33253
C	-5.43345	0.89319	-0.72274
C	-5.06196	1.26749	-2.16201
C	-3.66845	0.82800	-2.61163
C	-2.54384	1.23725	-1.66175
H	-3.54004	0.08367	1.36741
H	-2.65480	1.62010	1.29152
H	-5.18145	1.74798	1.25788
H	-4.40134	2.66121	-0.01566
H	-5.33547	-0.19132	-0.57298
H	-6.49075	1.13229	-0.57190
H	-5.79772	0.83783	-2.85029
H	-5.14020	2.35794	-2.26359
H	-3.63294	-0.26262	-2.73059
H	-3.45067	1.26048	-3.59409
H	-1.56344	1.08846	-2.12298
H	-2.61821	2.28932	-1.37492

H	1.40612	-0.03685	-2.73925
H	2.53781	-0.60997	-3.97477
H	1.62167	-1.77869	-3.01224
H	5.11168	-2.00832	-1.59906
H	3.76733	-2.95727	-2.26438
H	4.66288	-1.83998	-3.30204
H	4.86787	0.54006	-1.28507
H	4.47299	0.64749	-3.00456
H	3.37799	1.35245	-1.80660

azetidine_TS

C	2.08816	4.01761	1.44154
C	0.35188	-2.64713	0.17864
C	2.88069	-1.60651	-1.34645
C	2.79892	-1.68793	1.78364
Si	1.81445	-1.49079	0.20572
C	0.89711	3.70644	0.79167
C	3.04680	3.03279	1.72419
C	2.85654	1.69926	1.37399
O	-0.32963	1.78162	-0.19309
C	0.72892	2.37434	0.45349
C	1.66163	1.38631	0.72911
N	1.10201	0.19551	0.22063
C	-0.09271	0.45323	-0.33536
H	2.28094	5.04486	1.73241
H	3.96375	3.31838	2.22906
H	3.60778	0.94957	1.59395
H	0.70055	-3.68662	0.18620
H	-0.26832	-2.50290	-0.71039
H	-0.27992	-2.49866	1.06192
C	1.99126	-1.57871	-2.59664
C	3.87182	-0.43594	-1.40102
C	3.65689	-2.93197	-1.29756
H	2.30196	-1.20435	2.63171
H	3.81821	-1.29668	1.71157
H	2.87549	-2.75689	2.01464
H	0.14739	4.45445	0.55956
C	-2.73532	-0.45973	1.37125
C	-3.13387	-1.26054	2.43508
C	-3.51222	-2.58503	2.21135
C	-3.48202	-3.11480	0.92331
C	-3.06981	-2.32196	-0.14306
C	-2.70795	-0.98974	0.07694
H	-2.40595	0.55900	1.55252
H	-3.14232	-0.85429	3.44140
H	-3.82255	-3.20647	3.04559
H	-3.76835	-4.14696	0.74970
H	-3.02926	-2.73280	-1.14825
C	-2.30182	-0.19475	-1.09332
N	-2.75354	0.99236	-1.33188
C	-2.55649	1.87766	-2.49164
C	-3.46067	2.92444	-1.80224
C	-3.50553	2.00328	-0.56118
H	-1.85306	-0.70802	-1.93965
H	-2.92334	1.43778	-3.42092
H	-1.50472	2.15970	-2.59056
H	-4.42735	3.05326	-2.28749
H	-2.99399	3.89370	-1.63427
H	-2.93449	2.37101	0.29321
H	-4.48252	1.63701	-0.23839
H	1.38327	-0.66620	-2.64414

H	2.61309	-1.60900	-3.50131
H	1.31455	-2.44045	-2.63214
H	4.52120	-0.40220	-0.51741
H	4.52265	-0.53848	-2.27972
H	3.35821	0.52938	-1.48209
H	4.33290	-2.97758	-0.43537
H	2.98692	-3.79929	-1.24985
H	4.26932	-3.04095	-2.20248

bzoxazole-1-

H	-0.86449	2.51811	0.00000
H	-0.85926	-2.50084	-0.00000
H	-3.00980	-1.22536	0.00000
H	-3.01028	1.24166	0.00000
N	1.66136	1.13483	0.00000
C	2.44890	0.07391	-0.00001
O	1.58692	-1.10786	-0.00000
C	-0.86687	1.43110	0.00000
C	0.33448	0.72101	0.00000
C	0.31301	-0.68218	-0.00000
C	-0.86723	-1.41470	-0.00000
C	-2.06331	-0.69133	0.00000
C	-2.06247	0.70978	0.00000

bzoxazole

C	2.10153	0.69585	-0.00000
C	2.08692	-0.71046	0.00000
C	0.89673	-1.43105	0.00000
C	-0.26065	-0.66778	0.00000
C	-0.27408	0.72742	-0.00000
C	0.92683	1.43804	-0.00000
O	-1.56038	-1.08818	0.00000
C	-2.27979	0.06259	-0.00000
N	-1.60538	1.15703	-0.00000
H	3.05682	1.21100	-0.00000
H	3.02923	-1.24899	0.00000
H	0.87227	-2.51498	0.00000
H	0.93313	2.52295	-0.00000
H	-3.35571	-0.05140	-0.00000

bzox-tms-imin-adduct

C	-2.90245	3.66851	0.06801
C	-3.68518	-1.69290	-0.90577
C	-0.97267	-2.79303	-1.56938
C	-1.95616	-2.65553	1.45904
Si	-1.94825	-1.91605	-0.24746
C	-1.55262	3.42944	-0.15408
C	-3.81214	2.61514	0.26014
C	-3.42485	1.28163	0.22127
O	0.06919	1.57155	-0.33747
C	-1.19330	2.09375	-0.18449
C	-2.07423	1.03268	-0.02660
N	-1.29113	-0.14300	-0.12083
C	2.75160	1.14135	0.71078
C	3.99389	1.76544	0.77058
C	4.98963	1.43745	-0.14802
C	4.73901	0.48328	-1.12989
C	3.49496	-0.13975	-1.19057
C	-0.04256	0.25886	-0.27406
C	2.49179	0.18174	-0.27425
C	1.19000	-0.60309	-0.33840
C	1.03973	-1.20087	2.04192
C	1.98434	-2.73793	0.46875

N	1.01186	-1.66502	0.65711
H	-3.25984	4.69168	0.10698
H	-4.85400	2.84894	0.45097
H	-4.14336	0.49184	0.39074
H	1.98482	1.41304	1.43093
H	4.18340	2.51098	1.53649
H	5.95709	1.92739	-0.09867
H	5.50840	0.22419	-1.85043
H	3.29932	-0.88355	-1.95958
H	1.15801	-1.09979	-1.31901
H	0.31047	-0.39717	2.19722
H	0.75785	-2.03619	2.68860
H	2.03237	-0.84494	2.35360
H	1.69415	-3.58798	1.09316
H	3.00548	-2.43411	0.74456
H	1.99228	-3.05728	-0.57761
H	-3.76341	-0.91165	-1.66911
H	-4.43135	-1.50747	-0.12902
H	-3.95949	-2.64049	-1.38584
H	-0.81042	-2.15521	-2.44569
H	-0.01548	-3.18308	-1.22185
H	-1.57711	-3.64379	-1.90644
H	-1.02085	-3.17529	1.67907
H	-2.78109	-3.37305	1.53417
H	-2.11758	-1.88752	2.22374
H	-0.82562	4.22191	-0.28665

bzox-TS

C	2.35364	3.99142	1.05625
C	2.38703	-1.69579	2.06222
C	-0.10962	-2.52283	0.45019
C	2.59660	-2.00540	-1.04478
Si	1.49486	-1.57006	0.42388
C	1.19111	3.73431	0.33474
C	3.15918	2.95018	1.54141
C	2.83722	1.61230	1.33147
O	-0.17505	1.84856	-0.53679
C	0.89079	2.39728	0.13693
C	1.66985	1.35461	0.61601
N	1.01166	0.17934	0.19966
C	-2.85975	0.34458	1.15000
C	-3.37585	-0.14186	2.34492
C	-3.96715	-1.40513	2.39059
C	-4.02959	-2.19204	1.24294
C	-3.49682	-1.71800	0.04846
C	-0.09205	0.49345	-0.49786
C	-2.92892	-0.44098	-0.00712
C	-2.36586	-0.01876	-1.29981
C	-3.31768	2.23843	-1.29787
C	-1.97673	1.39820	-3.18651
N	-2.59762	1.11363	-1.89389
H	2.64457	5.01954	1.24448
H	4.05980	3.19408	2.09524
H	3.47010	0.81791	1.70983
H	-2.37338	1.31567	1.12298
H	-3.31175	0.46188	3.24430
H	-4.36961	-1.77931	3.32667
H	-4.47915	-3.17894	1.27916
H	-3.52040	-2.33639	-0.84506
H	-1.91713	-0.79227	-1.91396
H	-3.84629	2.75719	-2.09912

H	-4.03559	1.88014	-0.56201
H	-2.60593	2.92553	-0.83160
H	-2.75286	1.67043	-3.90519
H	-1.43195	0.52122	-3.53657
H	-1.28336	2.23505	-3.06363
H	2.33886	-2.73453	2.40923
H	1.91370	-1.07242	2.82861
H	3.44480	-1.42237	1.99743
H	-0.74494	-2.18780	1.27812
H	-0.67349	-2.40285	-0.47984
H	0.09010	-3.59137	0.59228
C	3.19118	-3.40180	-0.80466
C	3.73325	-0.98208	-1.17579
C	1.76908	-2.01647	-2.33697
H	0.55896	4.52560	-0.05261
H	3.81899	-3.69384	-1.65700
H	3.82180	-3.42810	0.09202
H	2.41280	-4.16695	-0.69470
H	4.39888	-1.26663	-2.00167
H	3.35377	0.02392	-1.39041
H	4.34630	-0.92772	-0.26774
H	2.41748	-2.23206	-3.19676
H	0.98943	-2.78678	-2.31052
H	1.28605	-1.04797	-2.52082

cy1_TS

C	4.01688	0.21152	3.31421
C	-0.12393	-0.27817	-2.34375
C	2.96615	-0.31574	-2.35867
C	1.38204	-2.83761	-1.38902
Si	1.41432	-0.95433	-1.53489
C	2.68144	0.59794	3.37237
C	4.56397	-0.36647	2.15770
C	3.80190	-0.58725	1.01493
O	0.62504	0.66579	1.97908
C	1.94462	0.37295	2.22171
C	2.46339	-0.20359	1.07372
N	1.39185	-0.24260	0.15529
C	0.30246	0.30292	0.71875
H	4.65044	0.36271	4.18180
H	5.61125	-0.64961	2.15423
H	4.23476	-1.03670	0.12847
H	-0.05890	0.81130	-2.42901
H	-1.02616	-0.53737	-1.77947
H	-0.22699	-0.68591	-3.35574
H	3.17100	0.72056	-2.06857
H	3.85212	-0.91737	-2.13362
H	2.82640	-0.33155	-3.44570
C	2.56891	-3.37487	-0.57874
C	1.45947	-3.39703	-2.82002
C	0.07649	-3.29946	-0.72943
H	2.24108	1.04829	4.25456
C	-1.19305	3.04838	-1.65328
C	-0.53447	4.09062	-2.29670
C	0.46327	4.80464	-1.63519
C	0.79822	4.48193	-0.32113
C	0.14194	3.44211	0.32516
C	-0.85137	2.71102	-0.33856
H	-1.97482	2.51717	-2.18883
H	-0.80552	4.34888	-3.31519
H	0.97373	5.61765	-2.14178

H	1.56726	5.04204	0.20068
H	0.39712	3.19008	1.35114
C	-1.53063	1.63698	0.41239
N	-2.57648	1.00189	-0.05212
C	-3.32944	0.00464	0.71598
C	-4.83171	0.23969	0.57070
C	-5.61366	-0.82558	1.34533
C	-5.22474	-2.23864	0.90373
C	-3.71704	-2.46207	1.03981
C	-2.93502	-1.40161	0.26146
H	-1.44427	1.66143	1.49499
H	-2.76937	1.02591	-1.05134
H	-3.03719	0.14165	1.76429
H	-5.09389	0.18499	-0.49582
H	-5.08527	1.24580	0.92052
H	-6.68736	-0.66237	1.20911
H	-5.40817	-0.71151	2.41829
H	-5.51778	-2.38347	-0.14516
H	-5.77198	-2.98131	1.49344
H	-3.43839	-3.45915	0.68281
H	-3.43497	-2.41174	2.10012
H	-1.85546	-1.53197	0.38839
H	-3.15812	-1.49556	-0.81195
H	2.54674	-4.47293	-0.57897
H	3.53247	-3.06954	-1.00367
H	2.53485	-3.04702	0.46621
H	1.43233	-4.49426	-2.79111
H	0.61752	-3.06579	-3.43944
H	2.38884	-3.10535	-3.32408
H	0.05352	-4.39606	-0.67361
H	-0.01617	-2.91602	0.29443
H	-0.80548	-2.98011	-1.29754

cy2_TS

C	-2.32704	-3.51803	2.95934
C	-4.74697	1.31875	0.95153
C	-2.46028	2.43081	-0.78266
C	-4.20043	0.07854	-1.87419
Si	-3.46584	0.91882	-0.35189
C	-1.17752	-3.05114	2.32822
C	-3.57316	-2.90605	2.75549
C	-3.72372	-1.80385	1.91883
O	-0.41003	-1.29206	0.74979
C	-1.35124	-1.95476	1.50069
C	-2.57223	-1.33177	1.29174
N	-2.30111	-0.27852	0.39321
C	-0.99405	-0.27344	0.07480
H	-2.25906	-4.37530	3.62081
H	-4.44591	-3.30399	3.26272
H	-4.69465	-1.34638	1.76685
H	-5.55619	0.58420	1.00890
H	-5.20147	2.28696	0.71129
H	-4.29248	1.40850	1.94435
H	-2.02481	2.88668	0.11345
H	-1.64844	2.20679	-1.48065
H	-3.11247	3.17686	-1.25247
C	-3.11044	-0.11512	-2.93661
C	-5.30941	0.98320	-2.43404
C	-4.79814	-1.28576	-1.50461
H	-0.20612	-3.51267	2.46844
C	0.53121	2.15346	1.35832

C	0.43574	3.35619	2.05200
C	0.67411	4.56434	1.39856
C	0.99236	4.57183	0.04207
C	1.07863	3.37282	-0.65857
C	0.87404	2.15873	0.00464
H	0.31293	1.21714	1.86048
H	0.16566	3.34874	3.10318
H	0.59835	5.50008	1.94349
H	1.16365	5.51092	-0.47427
H	1.31708	3.37705	-1.71927
C	0.97810	0.93843	-0.82778
N	1.90834	0.03173	-0.78146
C	3.05415	-0.00293	0.17206
C	3.81706	1.32239	0.27144
C	5.12006	1.08089	1.04063
C	4.83034	0.50957	2.43038
C	4.01911	-0.78405	2.33580
C	2.71803	-0.57968	1.54978
C	1.85053	-1.02051	-1.84711
C	2.82197	-0.71477	-3.00036
C	4.25226	-1.22880	-2.80455
C	4.25190	-2.71917	-2.45369
C	3.42041	-3.01626	-1.20249
C	1.99414	-2.46391	-1.33025
H	0.37055	0.93687	-1.72677
H	3.74249	-0.70321	-0.29729
H	3.23071	2.07621	0.80432
H	4.01961	1.70685	-0.73572
H	5.67534	2.02086	1.11994
H	5.75460	0.37964	0.48061
H	4.26340	1.25054	3.01068
H	5.76630	0.32856	2.96904
H	3.78449	-1.16513	3.33493
H	4.62307	-1.55422	1.83511
H	2.17903	-1.52597	1.44314
H	2.06626	0.11292	2.09231
H	0.83096	-0.93165	-2.23046
H	2.80786	0.36088	-3.21111
H	2.40571	-1.21491	-3.88419
H	4.77913	-0.65465	-2.03190
H	4.80878	-1.06555	-3.73346
H	5.27719	-3.07840	-2.31872
H	3.82828	-3.27661	-3.30059
H	3.92566	-2.62618	-0.31115
H	3.35158	-4.09928	-1.05413
H	1.47698	-3.07381	-2.08204
H	1.42905	-2.58117	-0.40106
H	-2.27831	-0.72293	-2.55818
H	-3.52524	-0.63121	-3.81281
H	-2.70418	0.84351	-3.28004
H	-6.12556	1.11955	-1.71433
H	-4.93160	1.97485	-2.71106
H	-5.73995	0.53229	-3.33810
H	-5.58179	-1.19921	-0.74190
H	-5.25646	-1.74440	-2.39124
H	-4.03574	-1.97988	-1.13175

etnipr2_1

N	0.00232	0.27293	-0.21486
C	-1.03427	-0.77057	-0.19864
C	-0.30903	1.45644	0.57924

C	1.38475	-0.19731	-0.05867
C	-1.66415	-1.06642	1.17155
C	-2.12294	-0.42572	-1.21584
C	0.27438	2.73065	-0.02478
C	1.64864	-1.06440	1.17945
C	1.86230	-0.90546	-1.32680
H	-0.54760	-1.69129	-0.53984
H	0.01817	1.35455	1.63005
H	-1.39740	1.56793	0.60818
H	1.99188	0.70929	0.04566
H	-0.91094	-1.26321	1.93898
H	-2.28827	-0.23379	1.51412
H	-2.31019	-1.94816	1.09931
H	-2.88432	-1.21230	-1.26079
H	-2.62747	0.50990	-0.94622
H	-1.68678	-0.29996	-2.21117
H	-0.02444	3.60455	0.56369
H	1.36816	2.71318	-0.05511
H	-0.09015	2.85651	-1.04903
H	1.31901	-0.56728	2.09806
H	1.13435	-2.02950	1.10536
H	2.72082	-1.26740	1.27594
H	2.93281	-1.12751	-1.26186
H	1.34055	-1.85646	-1.48240
H	1.68752	-0.27270	-2.20201

Hetnipr2_1

N	0.02235	0.25951	-0.27235
C	-1.14288	-0.72524	-0.12683
C	-0.18870	1.55352	0.47240
C	1.40172	-0.35596	-0.04381

C	-1.75515	-0.72435	1.26789
C	-2.17286	-0.40266	-1.19951
C	0.61735	2.69386	-0.12250
C	1.50131	-1.10096	1.27681
C	1.75434	-1.23605	-1.23483
H	-0.70686	-1.70577	-0.32876
H	0.05860	1.37293	1.51991
H	-1.25335	1.78046	0.40418
H	2.07922	0.50050	-0.02838
H	-1.01507	-0.83092	2.06283
H	-2.34731	0.17633	1.45056
H	-2.43316	-1.58019	1.32652
H	-3.00117	-1.11177	-1.12457
H	-2.58104	0.60569	-1.07040
H	-1.74503	-0.48869	-2.20382
H	0.34033	3.61490	0.39663
H	1.69561	2.56416	-0.00964
H	0.38448	2.82817	-1.18396
H	1.23794	-0.47924	2.13659
H	0.88311	-2.00352	1.28101
H	2.54218	-1.41132	1.40226
H	2.78869	-1.57218	-1.12640
H	1.12051	-2.12594	-1.29174
H	1.67852	-0.68356	-2.17745
H	0.02656	0.52037	-1.26664

morpholine_TS

C	-1.71494	4.02119	2.05226
C	-1.30676	-2.58622	-0.18990
C	-3.22274	-0.75473	-1.86669
C	-3.75260	-1.29922	1.16888

Si	-2.46721	-1.12494	-0.17800
C	-0.54089	3.53956	1.47985
C	-2.89633	3.26445	2.04639
C	-2.95487	1.99719	1.47346
O	0.36141	1.55816	0.27873
C	-0.62214	2.27764	0.91591
C	-1.77821	1.51194	0.90680
N	-1.42627	0.31780	0.24458
C	-0.13805	0.36392	-0.13332
H	-1.71699	5.00491	2.50986
H	-3.79083	3.67880	2.49962
H	-3.87464	1.42395	1.47269
H	-1.86248	-3.50339	-0.41792
H	-0.51486	-2.47519	-0.93636
H	-0.83489	-2.71529	0.79114
C	-2.13533	-0.78426	-2.94894
C	-3.89143	0.62686	-1.85307
C	-4.27772	-1.83003	-2.16986
H	-3.32403	-1.11793	2.16062
H	-4.61196	-0.63488	1.03598
H	-4.13081	-2.32821	1.15904
H	0.37941	4.11259	1.46969
C	1.83328	-1.26720	1.79897
C	1.80182	-2.20277	2.82574
C	1.96419	-3.55960	2.54238
C	2.14453	-3.98435	1.22826
C	2.15696	-3.05378	0.19383
C	2.02139	-1.69102	0.47779
H	1.67762	-0.21420	2.01638
H	1.64306	-1.87447	3.84778
H	1.93925	-4.28665	3.34812
H	2.26037	-5.04002	1.00550
H	2.27296	-3.38439	-0.83491
C	2.00754	-0.76676	-0.66861
N	2.72940	0.30600	-0.79436
C	3.59152	0.89153	0.24376
C	4.91914	1.29921	-0.38914
C	3.99044	1.52973	-2.50095
C	2.60539	1.14027	-2.00084
H	1.52972	-1.11707	-1.57755
H	3.07614	1.76658	0.65335
H	3.75943	0.15988	1.03301
H	5.52035	1.82986	0.35206
H	5.46491	0.39994	-0.71155
H	3.89468	2.23475	-3.32908
H	4.52614	0.63553	-2.85315
H	2.04861	0.58486	-2.75878
H	2.03890	2.03336	-1.71522
O	4.72318	2.17159	-1.47965
H	-2.56738	-0.51893	-3.92309
H	-1.32922	-0.06954	-2.73820
H	-1.68928	-1.78099	-3.04657
H	-4.38255	0.81365	-2.81754
H	-4.66084	0.70514	-1.07505
H	-3.16329	1.43070	-1.69276
H	-4.71376	-1.65864	-3.16303
H	-3.84798	-2.83945	-2.17125
H	-5.09844	-1.81194	-1.44301

ph-iminium

H	3.96254	-0.65576	0.73714

H	3.34089	-1.60460	-0.65007
H	3.96026	0.05377	-0.89958
H	2.98436	1.41149	1.19772
H	2.14187	2.03853	-0.23933
H	1.20222	1.47037	1.17818
H	1.21374	-1.76770	-0.54013
H	-0.10391	1.76559	-0.66374
H	-2.50091	2.29085	-0.46728
H	-4.12172	0.52044	0.14826
H	-3.34672	-1.80658	0.52535
H	-0.93985	-2.35477	0.30141
N	2.09254	-0.04895	0.02322
C	3.42883	-0.61048	-0.21426
C	2.09494	1.31146	0.57510
C	1.03219	-0.74001	-0.23027
C	-0.35592	-0.32188	-0.12998
C	-0.80061	0.99004	-0.36622
C	-2.15431	1.28159	-0.27204
C	-3.06599	0.28118	0.06699
C	-2.63346	-1.02718	0.27994
C	-1.28574	-1.33454	0.15901

piperazine_TS

C	-0.59354	-4.05964	2.22860
C	-2.05373	2.32204	-0.25530
C	-3.46066	-0.03956	-1.75221
C	-3.99884	0.49506	1.28523
Si	-2.76852	0.60391	-0.11882
C	0.39415	-3.29265	1.61700
C	-1.93718	-3.65578	2.22977
C	-2.35063	-2.47248	1.62431
O	0.69874	-1.17880	0.34565
C	-0.04009	-2.12029	1.02220
C	-1.36194	-1.70169	1.01612
N	-1.35976	-0.47976	0.31136
C	-0.11119	-0.18428	-0.09061
H	-0.31890	-4.99091	2.71275
H	-2.67672	-4.28455	2.71454
H	-3.39252	-2.17492	1.63214
H	-2.85248	3.03979	-0.47647
H	-1.30467	2.39507	-1.04967
H	-1.58239	2.62514	0.68673
C	-3.74054	-1.54544	-1.65640
C	-4.77222	0.70571	-2.04576
C	-2.45904	0.22000	-2.88524
H	-4.63004	1.39130	1.26772
H	-3.49663	0.46708	2.25842
H	-4.66203	-0.37242	1.21277
H	1.43688	-3.59029	1.60176
C	1.36167	2.04895	1.59933
C	1.12603	3.00210	2.58315
C	0.89692	4.33220	2.22932
C	0.89436	4.70858	0.88858
C	1.11438	3.75507	-0.10141
C	1.36411	2.42624	0.25259
H	1.51638	1.00852	1.87213
H	1.11382	2.70553	3.62692
H	0.71243	5.07285	3.00103
H	0.70810	5.74088	0.61071
H	1.09074	4.04054	-1.14986
C	1.56351	1.46927	-0.85501

N	2.62137	0.73760	-1.03151
C	3.77279	0.64364	-0.11892
C	4.09969	-0.81404	0.17225
N	4.34863	-1.52631	-1.07336
C	4.76792	-2.89427	-0.81600
C	3.15660	-1.49954	-1.90974
C	2.78462	-0.06291	-2.25686
H	0.91326	1.56971	-1.71733
H	3.56751	1.20176	0.79171
H	4.61588	1.10643	-0.64309
H	3.27785	-1.27163	0.75145
H	5.00574	-0.84171	0.78516
H	3.99644	-3.47913	-0.28346
H	4.98565	-3.39495	-1.76377
H	5.67829	-2.89028	-0.20980
H	2.30239	-1.99737	-1.41956
H	3.36607	-2.02796	-2.84507
H	3.58827	0.39772	-2.84057
H	1.85025	-0.02500	-2.82016
H	-4.18794	-1.90227	-2.59382
H	-2.82254	-2.12124	-1.48915
H	-4.44393	-1.78341	-0.84923
H	-5.18578	0.37192	-3.00672
H	-5.53066	0.51436	-1.27730
H	-4.62326	1.79050	-2.11291
H	-2.84583	-0.18520	-3.82981
H	-2.28390	1.29208	-3.03378
H	-1.49113	-0.26072	-2.69155

pyrrolidine_TS

C	1.32445	4.12462	1.80539
C	1.04649	-2.59023	-0.15347
C	3.26637	-0.89576	-1.56567
C	3.26206	-1.28786	1.53933
Si	2.22677	-1.15265	-0.01306
C	0.22348	3.63631	1.10733
C	2.47551	3.34490	1.99396
C	2.57459	2.04799	1.49828
O	-0.56807	1.61117	-0.09703
C	0.34425	2.34464	0.62352
C	1.47060	1.55653	0.80479
N	1.17531	0.33491	0.16468
C	-0.05254	0.38665	-0.37890
H	1.29322	5.13124	2.20894
H	3.31368	3.76467	2.54029
H	3.47144	1.45835	1.64798
H	1.61069	-3.51884	-0.30076
H	0.35525	-2.47577	-0.99371
H	0.45640	-2.70091	0.76362
C	2.36947	-0.94808	-2.80933
C	3.98463	0.45930	-1.51097
C	4.30880	-2.02275	-1.63568
H	2.69029	-1.01050	2.43172
H	4.17275	-0.68172	1.50900
H	3.57077	-2.33291	1.66069
H	-0.67260	4.22530	0.94634
C	-2.05268	-1.13178	1.59089
C	-2.08692	-2.03226	2.64917
C	-2.31004	-3.38881	2.41065
C	-2.48434	-3.84947	1.10800
C	-2.43064	-2.95495	0.04367

C	-2.23378	-1.59047	0.28126
H	-1.84121	-0.08498	1.78462
H	-1.93320	-1.67512	3.66237
H	-2.33739	-4.08713	3.24137
H	-2.64752	-4.90525	0.91756
H	-2.54175	-3.31443	-0.97583
C	-2.18459	-0.72000	-0.90831
H	-1.74351	-1.13857	-1.80862
N	-2.88096	0.35786	-1.07765
C	-3.69716	1.06254	-0.06107
C	-4.17664	2.32307	-0.78901
C	-4.13925	1.93837	-2.27089
C	-2.87552	1.09261	-2.35846
H	-3.09244	1.29689	0.81379
H	-4.51703	0.40052	0.23264
H	-5.16638	2.63094	-0.44839
H	-3.47653	3.14295	-0.60098
H	-5.01199	1.33305	-2.53514
H	-4.09940	2.80267	-2.93559
H	-1.97036	1.71104	-2.38449
H	-2.85407	0.38241	-3.18640
H	1.57620	-0.19065	-2.77237
H	2.96634	-0.75927	-3.71166
H	1.89627	-1.93006	-2.92580
H	4.62812	0.54959	-0.62724
H	4.62642	0.57733	-2.39438
H	3.27721	1.29679	-1.50386
H	4.99820	-1.99401	-0.78341
H	3.84175	-3.01506	-1.66054
H	4.90993	-1.92100	-2.54898

tms-bzoxazole-1-

H	-1.30493	-2.01184	-1.57976
H	-1.99185	-0.62645	-2.44277
H	-3.05780	-1.77194	-1.61303
H	-1.99187	-0.62640	2.44278
H	-3.05777	-1.77194	1.61306
H	-1.30490	-2.01179	1.57981
H	-3.19526	1.78391	0.88462
H	-4.29339	0.70728	-0.00001
H	-3.19526	1.78389	-0.88466
H	0.53270	-2.22635	-0.00001
H	3.87647	1.53195	0.00001
H	4.61943	-0.86046	0.00001
H	2.97268	-2.69109	-0.00000
C	-2.09436	-1.25322	-1.55025
C	-2.09435	-1.25319	1.55028
C	-3.28910	1.14706	-0.00001
Si	-2.01514	-0.21113	0.00000
N	-0.38600	0.57556	-0.00001
C	-0.22485	1.92259	-0.00000
O	1.12348	2.13305	-0.00000
C	1.25211	-1.41363	-0.00001
C	0.85810	-0.07827	-0.00001
C	1.79776	0.94196	-0.00000
C	3.16415	0.71434	0.00000
C	3.56081	-0.62152	0.00000
C	2.62271	-1.66381	-0.00000

tms-bzoxazole

C	2.60523	1.68294	0.00000
C	3.55559	0.64520	0.00000

C	3.18447	-0.69366	-0.00000
C	1.81855	-0.91783	-0.00000
C	0.86541	0.09126	-0.00001
C	1.23948	1.43340	-0.00000
O	1.15238	-2.12400	0.00000
C	-0.13224	-1.83997	0.00000
N	-0.38612	-0.55642	-0.00000
Si	-2.06918	0.27545	0.00000
C	-2.06539	1.26742	-1.57064
C	-2.06538	1.26743	1.57063
C	-3.26854	-1.14221	0.00001
H	2.95163	2.71058	0.00000
H	4.61026	0.89811	0.00001
H	3.90153	-1.50620	0.00000
H	0.51205	2.23785	-0.00000
H	-0.84598	-2.65438	0.00000
H	-1.90719	0.62155	-2.44075
H	-3.03555	1.76212	-1.69357
H	-1.29373	2.04407	-1.57310
H	-1.90717	0.62157	2.44075
H	-3.03555	1.76213	1.69357
H	-1.29373	2.04409	1.57308
H	-4.28656	-0.73611	0.00001
H	-3.17043	-1.76931	-0.89211
H	-3.17042	-1.76931	0.89212

TBS_bzox-1-

C	4.07155	-0.81466	-0.21810
C	-1.24328	-1.17989	2.01607
C	-2.26708	1.66493	1.46046
C	-2.24064	-0.45137	-0.84512
Si	-1.40918	0.17589	0.73179
C	3.73443	0.51319	-0.46997
C	3.11491	-1.73784	0.22753
C	1.78791	-1.37273	0.44259
O	1.80263	2.06976	-0.42322
C	2.41037	0.85564	-0.25107
C	1.45216	-0.04321	0.19639
N	0.26525	0.70770	0.28037
C	0.48148	1.99187	-0.10295
H	5.09463	-1.14189	-0.37287
H	3.41482	-2.76503	0.40885
H	1.05817	-2.09765	0.78508
H	-2.18263	-1.25014	2.57725
H	-0.44805	-0.95495	2.73537
H	-1.04716	-2.16660	1.58469
H	-1.75876	1.98644	2.37616
H	-2.27665	2.51047	0.76806
H	-3.30206	1.41515	1.72214
C	-2.54092	0.72655	-1.78101
C	-3.55401	-1.14959	-0.46148
C	-1.32520	-1.45140	-1.56390
H	4.45812	1.24095	-0.81976
H	-1.63372	1.28829	-2.03436
H	-2.98124	0.36053	-2.71878
H	-3.25369	1.42793	-1.33210
H	-3.37982	-2.02737	0.17250
H	-4.23690	-0.47676	0.07207
H	-4.07528	-1.49423	-1.36505
H	-1.06678	-2.30655	-0.92665

H	-1.83039	-1.84973	-2.45461
H	-0.39196	-0.98220	-1.89645

TBS_Et2_TS_conf_1

C	-2.48444	4.00891	-1.24659
C	-3.07323	-1.71309	-1.70297
C	-0.45844	-2.64831	-0.36391
C	-2.84616	-1.70420	1.41956
Si	-1.94032	-1.52443	-0.22679
C	-1.22825	3.71363	-0.72443
C	-3.43850	3.00471	-1.47097
C	-3.17822	1.66672	-1.18940
O	0.13918	1.79668	0.07039
C	-0.99192	2.37700	-0.45085
C	-1.91812	1.36939	-0.67387
N	-1.27647	0.18036	-0.26835
C	3.02197	-2.32874	-0.06057
C	3.35287	-3.18228	-1.10894
C	3.21764	-2.75064	-2.42627
C	2.75538	-1.46241	-2.69786
C	2.43592	-0.60224	-1.65370
C	-0.03960	0.45995	0.17768
C	2.57691	-1.03148	-0.33029
C	2.22279	-0.18603	0.82540
C	3.66186	1.72151	0.21113
C	2.37076	1.63660	2.35499
N	2.72096	0.98903	1.07636
H	-2.73246	5.03872	-1.48138
H	-4.40789	3.27836	-1.87423
H	-3.92685	0.90217	-1.36199
H	3.10915	-2.66803	0.96835
H	3.70453	-4.18659	-0.89629
H	3.46392	-3.42047	-3.24417
H	2.63533	-1.13094	-3.72429
H	2.04069	0.38793	-1.86207
H	1.72902	-0.67495	1.65991
C	3.02944	2.88206	-0.54496
H	4.44542	2.08995	0.88105
H	4.11803	1.00592	-0.47365
C	3.31182	1.19996	3.46795
H	1.33242	1.37667	2.57860
H	2.41872	2.71577	2.19616
H	-3.15675	-2.77888	-1.94591
H	-2.67110	-1.20660	-2.58719
H	-4.08604	-1.33978	-1.52222
H	0.10600	-2.44953	-1.28223
H	0.22257	-2.53402	0.48402
H	-0.78957	-3.69301	-0.39461
C	-3.55688	-3.06686	1.42776
C	-3.88531	-0.58703	1.58486
C	-1.84546	-1.64202	2.58105
H	-0.48043	4.47648	-0.53902
H	-4.08139	-3.21060	2.38175
H	-4.30316	-3.14274	0.62786
H	-2.85126	-3.89885	1.31401
H	-4.43453	-0.72474	2.52593
H	-3.41858	0.40457	1.61802
H	-4.62396	-0.58851	0.77420
H	-2.37673	-1.71678	3.53929

H	-1.12342	-2.46594	2.54011
H	-1.28624	-0.69733	2.58691
H	2.29019	2.53778	-1.27090
H	3.81948	3.41583	-1.08048
H	2.54252	3.58975	0.13171
H	4.35049	1.44226	3.22481
H	3.04830	1.71609	4.39487
H	3.23626	0.12212	3.64039

bzox-TBS-Et2-imin-adduct

C	-2.54279	3.75229	-1.57985
C	-2.72649	-1.79972	-1.86034
C	-0.17465	-2.81950	-0.57014
C	-2.52732	-1.88839	1.28619
Si	-1.63169	-1.67741	-0.35271
C	-1.23482	3.59625	-1.13860
C	-3.43678	2.66939	-1.63143
C	-3.06657	1.38426	-1.25529
O	0.31551	1.86456	-0.28423
C	-0.89115	2.30725	-0.77102
C	-1.74949	1.21887	-0.82634
N	-1.00503	0.10754	-0.35856
C	3.40759	-1.43654	-0.10511
C	4.29306	-1.95478	-1.04286
C	4.10838	-1.69260	-2.40081
C	3.03611	-0.90900	-2.81372
C	2.14657	-0.38797	-1.87335
C	0.19205	0.57043	-0.04116
C	2.32581	-0.65362	-0.51650
C	1.39086	-0.13058	0.57202
C	2.88544	1.80857	1.07544
C	1.40222	0.86647	2.81831
N	2.09324	0.66790	1.54655
H	-2.88435	4.73568	-1.88441
H	-4.45250	2.84175	-1.97042
H	-3.77620	0.56716	-1.28320
H	3.55115	-1.62895	0.95478
H	5.12867	-2.56536	-0.71485
H	4.80123	-2.09672	-3.13226
H	2.88883	-0.69496	-3.86771
H	1.31374	0.22005	-2.22181
H	0.98088	-0.98955	1.11768
C	4.23838	1.86659	1.77402
H	3.04293	1.69857	-0.00115
H	2.34331	2.75422	1.21718
H	2.14577	1.23349	3.53296
H	1.08289	-0.11957	3.17863
C	0.20646	1.82418	2.81499
H	-2.36633	-1.17682	-2.68618
H	-3.77278	-1.54868	-1.66434
H	-2.69795	-2.84081	-2.20252
H	-0.59960	-3.81827	-0.73465
H	0.41365	-2.57097	-1.45887
H	0.49962	-2.89327	0.28672
C	-3.66436	-0.86841	1.42733
C	-1.54699	-1.73059	2.45534
C	-3.11164	-3.31191	1.29091
H	-0.52972	4.41693	-1.07861
H	-3.28917	0.16118	1.47296

H	-4.21586	-1.05744	2.35725
H	-4.38163	-0.93621	0.60073
H	-2.07497	-1.87132	3.40732
H	-0.73816	-2.47012	2.41622
H	-1.09998	-0.72846	2.47493
H	-3.65451	-3.48235	2.22940
H	-3.82037	-3.46865	0.46858
H	-2.32965	-4.07708	1.22064
H	4.80937	2.73147	1.42227
H	4.13042	1.95845	2.85924
H	4.81278	0.95981	1.56232
H	0.48091	2.81787	2.44756
H	-0.62759	1.45449	2.20565
H	-0.17197	1.93811	3.83561

OTf-bzox-TBS-Et2-imin-adduct

C	1.09005	-2.34172	3.92002
C	-1.00777	-0.52185	-2.65411
C	0.54508	-2.98497	-1.64177
C	-2.50074	-2.70331	-1.05578
Si	-0.85148	-1.79431	-1.29980
C	0.78435	-1.00489	3.69736
C	0.89941	-3.31510	2.92548
C	0.38796	-3.00162	1.67204
O	-0.08274	0.50382	1.94067
C	0.27713	-0.72002	2.44352
C	0.06390	-1.66372	1.45115
N	-0.44209	-0.95651	0.33690
C	-3.25531	1.15845	0.98760
C	-4.64438	1.27736	0.94921
C	-5.26574	1.82943	-0.16634
C	-4.49486	2.27834	-1.23985
C	-3.10954	2.16700	-1.19587
C	-0.47692	0.31763	0.69318
C	-2.48045	1.59340	-0.08684
C	-0.95672	1.53417	-0.08376
C	1.00882	3.00182	-0.08566
C	-0.69301	3.30328	1.65919
N	-0.38717	2.79844	0.31842
H	1.49768	-2.63898	4.88028
H	1.16688	-4.34462	3.13798
H	0.26064	-3.76322	0.91265
H	-2.78455	0.71230	1.86183
H	-5.23727	0.93200	1.79064
H	-6.34746	1.91574	-0.20009
H	-4.97417	2.71687	-2.10977
H	-2.50147	2.52858	-2.02222
H	-0.62429	1.35391	-1.10885
C	1.15362	3.19046	-1.59014
H	1.34343	3.91553	0.41560
H	1.65247	2.18646	0.27285
H	0.14151	3.10918	2.34876
H	-1.55820	2.76737	2.05775
C	-1.01451	4.79230	1.61391
H	-0.09088	0.06615	-2.74332
H	-1.87644	0.13622	-2.56060
H	-1.12329	-1.08653	-3.58789
H	0.74713	-2.95721	-2.71912
H	1.47591	-2.70449	-1.14092

H	0.29168	-4.01715	-1.38022
C	-2.83950	-3.29334	-2.43861
C	-3.62287	-1.75366	-0.62383
C	-2.42081	-3.85133	-0.04115
H	0.94242	-0.23153	4.43955
H	-3.02844	-2.51189	-3.18292
H	-3.75053	-3.90149	-2.36275
H	-2.04307	-3.94477	-2.82100
H	-4.58689	-2.28029	-0.65190
H	-3.46923	-1.40398	0.40168
H	-3.70881	-0.87164	-1.27100
H	-3.38326	-4.38017	-0.01405
H	-1.65319	-4.58921	-0.30154
H	-2.22021	-3.48939	0.97332
H	2.17689	3.48773	-1.83418
H	0.95255	2.26710	-2.14151
H	0.46929	3.97216	-1.93668
H	-0.18481	5.36740	1.18978
H	-1.89838	4.96492	0.99210
H	-1.21091	5.17734	2.61990
S	2.87705	-0.33968	-0.40752
O	2.61926	0.09019	0.97474
O	3.43492	-1.68680	-0.56140
C	4.25687	0.74530	-0.94060
O	1.79566	0.00270	-1.35342
F	3.93464	2.03520	-0.80283
F	5.34691	0.51385	-0.20836
F	4.56165	0.53115	-2.22088

OTf-et2_iminium

C	3.70485	-0.25392	-0.17756
C	4.61112	-1.28973	0.01710
C	4.16988	-2.61186	0.01636
C	2.82420	-2.90161	-0.20247
C	1.91367	-1.87407	-0.42086
C	2.34911	-0.54394	-0.38122
C	1.38466	0.51950	-0.69352
C	1.79273	1.89861	1.29026
C	0.34958	2.68514	-0.59711
N	1.23009	1.62480	-0.05213
H	4.05574	0.77366	-0.20921
H	5.66250	-1.06390	0.16310
H	4.87925	-3.41835	0.17477
H	2.48254	-3.93154	-0.21171
H	0.86182	-2.08230	-0.58526
H	0.74367	0.38958	-1.56539
C	0.81277	1.41574	2.35271
H	2.75219	1.39815	1.38959
H	1.95742	2.97645	1.34783
C	1.18640	3.75231	-1.28767
H	-0.35304	2.21019	-1.28343
H	-0.22004	3.08756	0.24257
H	1.20864	1.67506	3.33850
H	-0.17214	1.87265	2.22972
H	0.68546	0.33231	2.28720
H	1.72643	3.32860	-2.13938
H	1.90883	4.21029	-0.60537
H	0.52305	4.53906	-1.65636
S	-1.82000	0.02655	-0.06307

O	-1.63448	0.26038	-1.50626
O	-0.82425	-0.88515	0.52831
C	-3.37591	-0.93878	0.01321
O	-2.11410	1.22768	0.72797
F	-3.25965	-2.07471	-0.67639
F	-4.38942	-0.24131	-0.50052
F	-3.67856	-1.24660	1.27485
